# Health Misinformation about Toxic-Site Harm: The Case for Independent-Party Testing to Confirm Safety

**DOI:** 10.3390/ijerph18083882

**Published:** 2021-04-07

**Authors:** Kristin Shrader-Frechette, Andrew M. Biondo

**Affiliations:** 1 Department of Biological Sciences, University of Notre Dame, 100 Malloy Hall, Notre Dame, IN 46556, USA; 2 Department of Economics, University of Notre Dame, 3060 Jenkins Nanovic Hall, Notre Dame, IN 46556, USA; abiondo@nd.edu

**Keywords:** Coldwell Banker Real Estate/Trammell Crow (CBRE/TCC), hazardous waste, Method TO-17, passive sampling, pollution, sorbent tube sampling, toxin, trichloroethylene (TCE), vapor intrusion, volatile organic compound

## Abstract

Health misinformation can cause harm if regulators or private remediators falsely claim that a hazardous facility is safe. This misinformation especially threatens the health of children, minorities, and poor people, disproportionate numbers of whom live near toxic facilities. Yet, perhaps because of financial incentives, private remediators may use safety misinformation to justify reduced cleanup. Such incentives exist in nations like the United States, where most toxic-site testing/remediation is semi-privatized or voluntary, conducted by private parties, commercial redevelopers, who can increase profits by underestimating health harm, thus decreasing required testing/remediation. Our *objective* is to begin to determine whether or not interested parties misrepresent health harm (at hazardous facilities that they test/remediate/redevelop) when they use traditional and social media to claim that these sites are safe. Our *hypothesis* is that, contrary to the safety claims of the world’s largest commercial developer, Coldwell Banker Real Estate/Trammell Crow (CBRE/TCC), the authors’ screening assessment, especially its lab-certified, toxic-site, indoor-air tests, show violations of all three prominent government, cancer-safety benchmarks. If so, these facilities require additional testing/remediation, likely put site renters at risk, and may reveal problems with privatized hazardous cleanup. To our knowledge, we provide the first independent tests of privatized, toxic-site assessments before cancer reports occur. Our screening assessment of this hypothesis tests indoor air in rental units on a prominent former weapons-testing site (the US Naval Ordnance Testing Station, Pasadena, California (NOTSPA) that is subject to carcinogenic vapor intrusion by volatile organic compounds, VOCs), then compares test results to the redeveloper’s site-safety claims, made to government officials and citizens through traditional and social media. Although NOTSPA toxic soil-gas concentrations are up to nearly a million times above allowed levels, and indoor air was never tested until now, both the regulator and the remediator (CBRE/TCC) have repeatedly claimed on social media that “the site is safe at this time.” We used mainly *Method* TO-17 and two-week sampling with passive, sorbent tubes to assess indoor-air VOCs. Our *results* show that VOC levels at every location sampled—all in occupied site-rental units—violate all three government-mandated safety benchmarks: environmental screening levels (ESLs), No Significant Risk Levels (NSRLs), and inhalation risks based on the Inhalation Unit Risk (IUR); some violations are two orders of magnitude above multiple safety benchmarks. These results support our hypothesis and suggest a need for independent assessment of privatized cleanups and media-enhanced safety claims about them. If our results can be replicated at other sites, then preventing health misinformation and toxic-facility safety threats may require new strategies, one of which we outline.

## 1. Introduction

In the United States and many other nations, most hazardous-facility testing and remediation is semi-privatized or “voluntary”; that is, private parties (usually the commercial redevelopers of the toxic sites) conduct sampling and cleanup under varying degrees of government oversight [1,2,3]. In such semi-privatized assessment/remediation, private parties negotiate (with regulators) the levels and types of assessment/cleanup they must conduct. In exchange, these private parties receive government-liability protection plus regulatory, financial, and other benefits [4].

### 1.1. The Controversy over Toxic-Site Health-and-Safety Misinformation

Of course, as with any health-affecting activity that is at least partly motivated by private profits, semi-privatized toxic-site testing/remediation is the subject of controversy. Privatization proponents claim their assessments/cleanups are cheaper, faster, and safe, but critics say such testing and remediation is risky, typically driven by purely private-profit motives, and frequently lacks adequate government oversight, e.g., [4].

Who is right about toxic-site health and safety? Do private, commercial hazardous-site redevelopers sometimes conduct flawed assessments and cleanup, misrepresent site health and safety, then use traditional and social media to publicize their misinformation? This analysis begins to answer these questions and to address the data gap regarding the adequacy of semi-privatized toxic-site assessment, remediation, and its possible misrepresentation.

### 1.2. Research Objective, Hypothesis, Significance

Until human-health problems have appeared, to our knowledge, no one has conducted proactive, independent (non-government, non-redeveloper), sampling to test whether semi-privatized toxic-site testing/cleanup actually satisfies the health-and-safety claims that private interests sometimes make on its behalf. New Jersey and Massachusetts, for instance, have two of the best-established, semi-privatized assessment/remediation programs; yet their results suggest safety problems, despite the limited evaluations government gives them. Only about 2% of Massachusetts’ semi-privatized projects receives minimal, post-cleanup government evaluation. Although 10% of New Jersey toxic sites receives annual, post-cleanup audits, these typically involve neither sampling nor site visits, and none of these audits are truly independent. Yet, Massachusetts data show that an average of only 29% of “completed” privatized cleanups pass this evaluation and have no major safety problems; 71% of audited sites fails to pass this safety test, and these results presumably are applicable to other, non-audited, hazardous sites—that are never investigated [4].

One obstacle to independent post-testing or cleanup sampling is how to fund it. An additional obstacle is how scientists can secure permission for it, given that toxic-site owners know they could face personal-injury, trespass, and endangerment lawsuits because of testing results. Later paragraphs show this is a problem we face in our toxic-site sampling.

Our *objective* is to begin to determine whether or not interested parties misrepresent health harm (at hazardous facilities that they test/remediate/redevelop) when they use traditional and social media to claim that these sites are safe. To begin to remedy the data gap regarding the adequacy of semi-privatized testing/cleanup and the veracity of safety claims about it, we sample a toxic site, claimed to be safe by its private remediators. We provide a preliminary assessment (see the authors’ Section 2.2.1) of whether or not interested parties misrepresent health harm associated with the site. Our specific *hypothesis* is that, contrary to the safety claims of the world’s largest commercial developer, Coldwell Banker Real Estate/Trammell Crow (CBRE/TCC) [5,6], the authors’ screening assessment, especially its lab-certified, toxic-site, indoor-air samples, show violations of all three prominent government, cancer-safety benchmarks.

Our hypothesis, aided by our own 2021, university-based, toxic-site sampling results, is *significant* because, if it proves to be correct, then toxic facilities may put site renters at risk, require additional testing/remediation, and reveal problems with privatized hazardous cleanup. Our hypothesis also is important because CBRE/TCC is a $65-billion-plus corporation [7], a trend-setter for all other remediation-and-redevelopment companies. It claims to have pioneered privatized remediation [8], and it touts itself as “the industry leader in brownfields development” [9]. Yet, if CBRE/TCC misrepresents the health and safety of its testing/remediation, other companies may do so as well, as they have neither CBRE/TCC’s financial and PR resources nor its remediation/technical expertise.

Likewise, our hypothesis is significant because some data indicate that CBRE/TCC may have misrepresented the health and safety of its toxic-facility testing and remediation in official government reports, presented through both traditional and social media. That is, the literature shows that the corporation appears to have claimed site safety for at least four of its current hazardous facilities; however, independent, university testing shows that all of the CBRE/TCC facilities fail preliminary data-quality analyses [10], and at least some of them also fail scientific data audits [11] and data usability evaluations [12]. This article adds to that literature by presenting the first known independent testing of a CBRE/TCC hazardous-site redevelopment, before any cancers have been reported, in order to determine whether empirical data, independent sampling, tend to corroborate or to falsify the corporation’s safety claims.

Our hypothesis also is important because at least 200 million people face risks from thousands of hazardous-waste sites in poor- and middle-income nations [13]. In the United States, more than 120,000 toxic sites have not been adequately remediated, and thousands of “remediated” locations are being re-evaluated because of recently discovered health threats or flawed testing/cleanup [14]. The health of millions of people depends both on reliable assessment/cleanup and on accurate information about resulting toxic-site health and safety risks. Yet, if our hypothesis is proved correct, then toxic facilities may put site renters at risk, require additional testing/remediation, and suggest problems with privatized hazardous cleanup.

### 1.3. Background on the Toxic Site to Be Evaluated

To provide a preliminary evaluation (see the authors’ Section 2.2.1) of current CBRE/TCC toxic-site-health-and-safety claims, we located a hazardous facility whose current renters, typically one-person businesses, were able to give permission for noninvasive, completely passive, indoor-air testing. Legally, such renters can provide such permission. There is no need to rely on a large corporate owner for permission. However, only CBRE/TCC/the site owner can give permission for soil/soil-gas/groundwater sampling, as it is invasive, requiring drilling/boring into structures and the ground.

**Figure 1:**
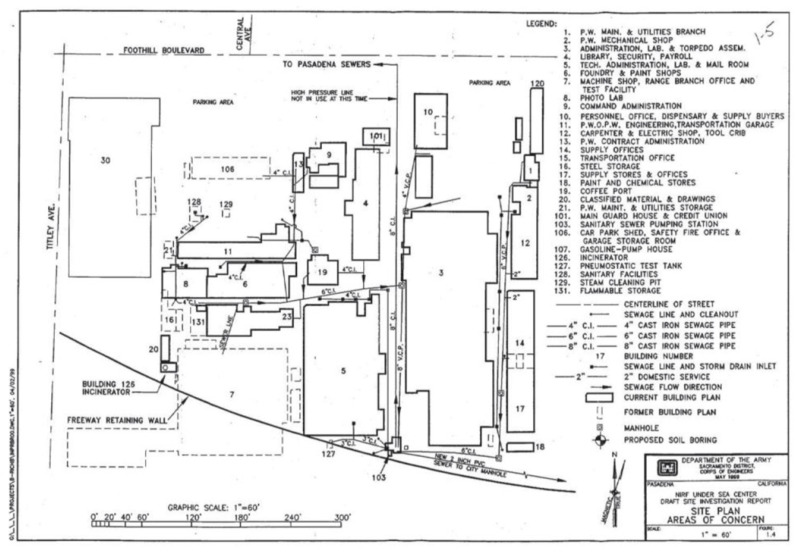
Map of the former US Naval Ordnance Test Station, Pasadena. (Map is from US Army Corps of Engineers, Draft Site Investigation Report, NIRF Under Sea Center Site Inspection, Figure 1.4).

The one such toxic site, on which it was possible to test indoor air because some current renters gave permission to do so, is the unremediated former US Naval Ordnance Testing Station, Pasadena, California (NOTPA), Envirostor ID 19970020; it is listed on the list of state of California hazardous-waste sites <https://www.envirostor.dtsc.ca.gov/public/profile_report.asp?global_id=19970020> (accessed on 28 March 2021) as the Naval Information Research Foundation [15]; see the authors’ Figure 1.

For at least seven reasons, the former NOTSPA may pose significant risks to health. *First*, it is a former US-military, classified (top-secret) weapons-manufacturing-and-testing facility that that is supposed to follow US CERCLA or Superfund cleanup rules. *Second*, NOTSPA abuts the heavily polluted, 10-lane Interstate 210, a main East-West, diesel-truck artery in Los Angeles County.

*Third*, the former NOTSPA facility is dangerous because it contains dioxins, furans, heavy metals, hexavalent chromium, PCBs, perchlorate, petroleum hydrocarbons, and radioactive materials [16]; however, the redeveloper admits that the main site “risk drivers” are chlorinated-solvent-VOC carcinogens in soil gas, such as carbon tetrachloride (CT), tetrachloroethylene (PCE), and trichloroethylene (TCE) [15] (p. 34). As the authors’ Table 1 and Table 2, show, site VOC concentrations are up to nearly a million times above safe (10^−6^ or one-in-a-million risk) levels [17] (Appendix D), that is, levels that cause no more than one cancer per million people exposed over a lifetime [15] (p. 33).

The authors’ Table 1 lists the results of 25% of CBRE/TCC’s former-NOTSPA perchloroethylene (PCE) soil-gas samples [17], 100% of which violate required environmental screening levels (ESLs). Yet, only two of these PCE locations will be removed, as they are in two of the 11 metals hotspots/drains, the only areas that the state regulator requires the redeveloper to remove, before building apartment residences onsite [15] (Appendix A, and Figure 7). Similarly, the authors’ Table 2 lists the results of 30% of CBRE/TCC’s former-NOTSPA carbon tetrachloride (CT) soil-gas samples [17], 60% of which violate required ESLs, and 40% of which used tests whose detection levels were 3000 times too lenient to find any effects higher than allowed levels. Again, only one of the many locations of disallowed levels of CT will be removed because it is in one of the 11 small, localized metals-hotspots/drains, the only areas that the state regulator requires the redeveloper to excavate [15] (Appendix A, and Figure 7).

*Fourth*, the former NOTSPA is risky because government admits that it poses vapor-intrusion threats to site renters and the general public, given its VOC carcinogens. Precisely because of these vapor-intrusion risks, in 2004 the state regulator, the California Department of Toxic Substances Control (DTSC) issued an Imminent and Substantial Endangerment (ISE) Order for the site [18]. Although this order mandated a schedule for removing and remedying potential and actual releases of NOTSPA hazardous substances, the NOTSPA responsible parties, including the site owner, instead legally battled the state regulator and conducted no additional cleanup. 

*Fifth*, despite this 2004 ISE Order, no one, including CBRE/TCC, has ever performed the required indoor-air and groundwater testing [17]. In addition, CBRE/TCC failed to conduct the required soil-gas testing under 86 % of (25 of 29) former NOTSPA buildings [15]; see later Section 3.2. *Sixth*, the former NOTSPA is dangerous because, despite the 2004 ISE Order by the DTSC and the lack of most required testing, in 2019 the state regulator (under a new site-project director), approved both CBRE/TCC’s limited 2007 NOTSPA testing, as well as its mitigation and redevelopment plans, including plans to leave most VOCs “in place” onsite and instead to use land-use controls [19,20]. *Seventh*, despite CBRE/TCC’s leaving most contaminants in place (see Table 1 and Table 2), CBRE/TCC has approval to build 550 small apartment units onsite [20,21] that will house disproportionate numbers of poor people, minorities, children, and families [12].

### 1.4. Toxic-Site Health-and-Safety Information from Regulators and from CBRE/TCC

Instead of cleaning up the former NOTSPA toxic site when the US Navy sold it in the middle 1970s to Space Bank, a storage-and-rental facility, the Navy merely paved the site with asphalt, except for what was covered by the World-War-II buildings; this pavement was supposed to ensure site safety [15]. Since then, Space Bank has rented units in the 29 old, unremodeled buildings. However, after CBRE/TCC negotiated to buy the site from Space Bank and went into escrow for the purchase, in 2007 CBRE/TCC and Space Bank conducted limited site-soil-gas testing. Their 2007 testing confirmed, as the 2004 Imminent and Substantial Endangerment Order had warned [18], that site renters face vapor-intrusion threats, because—as already mentioned—VOC soil-gas levels are nearly one million times above allowed levels (see the authors’ Table 1), and there is a complete exposure pathway from sub-slab soil gas into rental buildings [15,17].

As already mentioned, neither CBRE/TCC nor Space Bank has conducted additional sampling to address the vapor-intrusion threat to site renters. Yet, since 2007, CBRE/TCC has remained in escrow to buy the site and has negotiated with the state a Covenant Not to Sue, as part of its voluntary, semi-privatized testing and limited cleanup [22,23]. Although its own 2007 test results show dangerous VOC soil-gas levels—up to nearly one million times above what is allowed, beginning in 2016 CBRE/TCC began officially saying in its site-redevelopment documents that it found only “low-level VOCs” in site soil gas [15]. In 2019 the state regulator [24] (p. 50) and CBRE/TCC also repeatedly claimed and wrote to the public and to Pasadena City Council (who, as lead agency for the site, has authority to approve or disapprove CBRE/TCC’s site-mitigation/redevelopment plans) that “the site is safe at this time. Contamination is below paved surfaces and confined” [25]. In claiming safety for current site renters, yet in conducting neither indoor-air, nor groundwater, nor additional soil-gas testing—all required by toxic-site, regulatory guidance (see Section 2.2.3)—CBRE/TCC contradicted the 2004 ISE Order from California DTSC.

The preceding contradiction, the site’s high soil-gas levels, and the absence of indoor-air testing, to date, all suggest to the authors that indoor air should be tested. These three facts provided the motivation for our using indoor-air sampling to conduct a preliminary screening evaluation (see the authors’ Section 2.2.1) of our hypothesis. This hypothesis is that, contrary to the safety claims of one of the world’s largest redevelopers of hazardous-waste facilities (CBRE/TCC), the authors’ screening assessment, especially its university-based, lab-certified, toxic-site, indoor-air samples, show violations of all three prominent government, cancer-safety benchmarks.

## 2. Materials and Methods

### 2.1. Materials

Materials used in this study—to set up, collect, and analyze passive, noninvasive, VOC-sorbent-tube samplers in rental units on the former NOTSPA site—are listed below:

- Beacon Field Kit for indoor-air sampling and 12 Beacon Passive (Diffusion) Samplers; see the authors’ Chapter 1 in the online Supplemental Materials;
- Markes International thermal desorption system with auto recollection and mass-flow-controller module;
- Agilent 7890 Gas Chromatograph;
- Agilent 5975 Mass Spectrometer; and
- Beacon *Field Kit Guide for Air-Sampling Investigations*, including protocols; see the authors’ Chapter 1 in the online Supplemental Materials.

**Figure 2:**
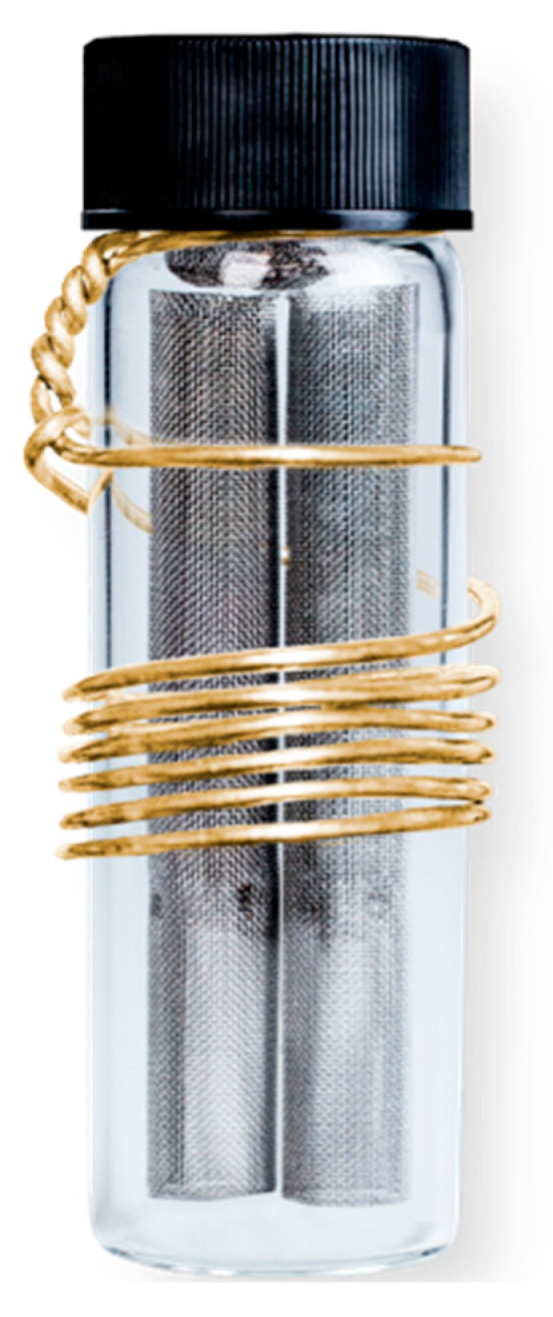
Beacon Passive Soil Gas Sampler.

Beacon Environmental Services, Inc, the undisputed US leader in passive soil-gas sampling and analysis <beacon-usa.com/> (accessed on 28 March 2021; see the authors’ Chapter 2 in the online Supplemental Materials)—supplied both the 12 sampler-tubes used for this study and analyzed them in its laboratory in Forest Hill, Maryland. Its 12 passive samplers are ¼-inch in diameter and 4.5-inch in length, packed with a Beacon proprietary sorbent that targets indoor-air, chlorinated VOCs over a two-week period (see the authors’ Figure 2).

### 2.2. Methods

Our hypothesis is that, contrary to the safety claims of the world’s largest commercial developer (CBRE/TCC), the authors’ screening assessment, especially its lab-certified, university-based, toxic-site, indoor-air sampling, shows violations of all three prominent government, cancer-safety benchmarks. Such misinformation by the redeveloper includes the claim that “the [former NOTSPA] site is safe at this time. Contamination is below paved surfaces and confined” [25].

To perform a preliminary test of this hypothesis (see the authors’ Section 2.2.1), we used 3 main methods. We (1) conducted noninvasive, passive, sorbent-tube sampling using US EPA Method TO-17 in all NOTSPA rental units in which tenants gave permission for such sampling; (2) instructed Beacon labs to analyze these indoor-air samples using US EPA Method TO-17, thermal desorption, and gas chromatography/mass spectrometry; and (3) used US EPA and DTSC screening-assessment methods to compare our sample results to safety benchmarks mandated by the California DTSC and the California Office of Environmental Health Hazard Assessment (OEHHA). We then compared test results to the redeveloper’s site-safety claims, made to government officials and citizens through traditional and social media. In addition, the authors provided an important plan for discovering a major way that the NOTSPA redevelopers use social media to claim that the toxic site is “safe.”

#### 2.2.1. Why Assessment of Our Hypothesis Is Preliminary

No scientific study can accomplish everything. Hence, this analysis is preliminary in at least seven senses.

##### 2.2.1.1. An Initial-Screening Assessment

First, for both scientific and practical reasons, this analysis is preliminary in providing an initial-screening assessment, including focused sampling of indoor air at the former NOTSPA; this screening was to determine mainly whether any samples violate any of three standard safety benchmarks, provided by federal and state government. The scientific reasons for conducting only initial screening are that the range of onsite indoor-air contaminants is unknown. No direct empirical data on current NOTSPA human-health risks have been available because there was no prior indoor-air sampling. Likewise no reliable inferences about current vapor-intrusion threats to building occupants have been possible because required [26,27] testing of subslab soil gases—what can travel to indoor air—has never been conducted for 25 of 29 (or 86% of) NOTSPA buildings [15]. Yet, results from the four buildings, that had required subslab sampling, are troubling; they reveal that only five feet below the rented structures, carcinogenic gases like PCE are up to 743,000 times above allowed levels (see the authors’ Table 1).

Another scientific reason for an initial screening assessment is that it requires focused sampling—conducted near likely indoor-air, high-contaminant locations such as floor drains, slab joints, and pipe penetrations [26,27]—not a statistically robust, full-site dataset for all former NOTSPA buildings. Government emphasizes that such datasets are rare in hydrogeochemical studies [27], and regulators do not require them for screening assessments. Unlike quantitative human health risk assessment or epidemiological assessment—the latter of which often requires thousands to tens of thousands of samples for an adequately powered, statistically robust dataset—guidance for vapor-intrusion screening requires merely using the maximum contaminant concentrations obtained after conducting the focused sampling, just described [26,27].

The main practical reason for performing an initial screening assessment, not a full-site, statistically robust dataset, is that full-site sampling is not an option. Why not? NOTSPA renters say they fear granting the required permission for our university-based, indoor-air testing of their units. This is because, for decades, site owners have not provided full access for NOTSPA testing [18,22,23]—that could be used to show owners’ liability for known harm to site occupants. As site renters all have month-to-month, below-market-rate leases for their units, they fear that their allowing testing could cause the owners to revoke the leases for their units (see authors’ Chapter 3 in the online Supplemental Materials). As a result, we tested only those units whose renters gave us written permission to do so.

Practically speaking, initial screening of indoor air in some NOTSPA rental units also makes sense because it may uncover conditions sufficient to induce the regulator to demand the full-site, indoor-air testing that its own technical documents require [26] but that has never been accomplished, despite the state’s ISE Order [18]; see the author’s Section 1.3. If even several indoor-air samples reveal contaminants above the ESL, NSRL, or the IUR-determined safety benchmarks, state regulatory guidance documents say this would be sufficient to trigger further testing [26], needed to help ensure the renters’ and site safety.

##### 2.2.1.2. Sampling Only of Indoor Air

In addition, this evaluation is preliminary in including only indoor-air sampling, not the DTSC-recommended testing of pairs of soil-gas and indoor-air samples at the same locations and times [26]. Pairwise, soil-gas sampling was impossible in our testing because such soil-gas testing is invasive, requires slab-boring, and therefore would require the permission of both the current Owner, Space Bank, and the incoming owner, CBRE/TCC.

Yet, neither Space Bank nor CBRE/TCC would give such permission, as they have been partners since 2007; both maintain that the hazardous facility is now safe; and both are adamant that it requires no additional testing, e.g., [25]. Besides, the results of any additional tests could show that both Space Bank and CBRE/TCC may have harmed site renters, just as the California ISE Order for the site suggested [18] (see the authors’ Section 1.3). Thus, Space Bank and CBRE/TCC could be legally liable for any harm revealed by further testing.

##### 2.2.1.3. Using Residential Safety Benchmarks

This assessment likewise is preliminary in focusing only on residential safety benchmarks for the former-NOTSPA toxic site. For three reasons, we spend little time assessing NOTSPA with respect to commercial benchmarks. First, the California regulator, DTSC, says that it “recommends that a residential scenario be assumed for site screening at all [toxic] facilities, both active and closing/closed.” Part of its reasoning is “that reuse of hazardous-waste sites could result in a change of ownership and land use, including potential residential reuse of the property.” [28] *Second*, the current Space Bank owner of the former NOTSPA guarantees that site rental tenants have 24/7 site access—which is equivalent to residential toxic exposures. *Third*, given the preceding guarantee, some rental tenants say they are onsite as many as 20 hours (not merely 8 hours) per day.

##### 2.2.1.4. A Six-Contaminant, Screening-Level Assessment

In addition, our assessment is preliminary regarding the types of samplers used. At each sampling location, for two weeks we deployed samplers A, capable of detecting all six high-soil-gas, site-risk compounds of interest, at an average Beacon detection limit of 0.24 ug/m^3^ above their current residential ESLs [29]. Instead, we could have deployed samplers B, capable of detecting only two of the six high-soil-gas-site-risk compounds (PCE and TCE), but detected by Beacon at their respective current residential ESLs [30] (see the authors’ Table 3).

As this analysis is an initial screening assessment, designed to begin to assess a range of site contaminants, not just two of them, the authors judged that it is more important, all things considered, to deploy samplers A for at least six reasons.

- Samplers A detect 300% more site-high-risk compounds than samplers B [29,30].
- The Sampler-A detection limits average only about 0.24 ug/m^3^ (roughly 80 parts per trillion) less sensitive than only one of three benchmarks, ESLs (see the authors’ Table 3).
- The general hypothesis to be tested is whether any toxic-site-contaminant concentrations violate any of the three prominent government indoor-air, cancer-safety benchmarks. Because government guidance requires calculating screening-assessment risk solely in terms of maximum-contaminant concentrations [26,27] (not lower concentrations, close to detection levels), and because of the other five reasons listed here, using samplers A is overall preferable to using samplers B.
- In 2019 state regulators approved CBRE/TCC’s completed site testing and planned contaminant mitigation [20], although its VOC sampling employed contaminant-detection levels (e.g., 20.0 ug/m^3^) that were as much as two orders of magnitude above the required screening levels (e.g., 0. 12 ug/m^3^), while the authors’ screening levels average only 0.24 ug/m^3^ above the ESLs; see the authors’ Table 3.
- Both samplers A and B employ the only US EPA “preferred” method (TO-17) for sampling/evaluating sorbent results, analyzed by the top US laboratory for such samplers [31], Beacon Environmental Services. Beacon wrote many USEPA sorbent-tube-sampler manuals, and Beacon is now conducting the largest sorbent-sampling-tube deployment projects in the United States, under contract with US EPA (see the authors’ Chapter 2 in the online Supplemental Materials).
- Method TO-17 has better detection limits and better reporting limits than the next-most-sensitive method (TO-15), and TO-15 is used with large stainless-steel canisters [32].

##### 2.2.1.5. Uncalibrated and Generally Calibrated Results for Sorbent Samples

In addition, these results are preliminary in that they provide only uncalibrated and generally calibrated sample results from the sorbent-tube manufacturer, not also individual-sample calibrated results. The latter would require having two sets of testing equipment, sorbent tubes and stainless-steel canisters, at every sampling location. Yet, having site-calibrated results was impossible because NOTSPA renters were unable to give permission (and had no space in their small workplaces) for testing with large canisters.

Nevertheless, as Section 3.4.1. explains, some calibration is essential because sorbent-tube results “consistently” underreport results; they show same-location contaminant concentrations that are two to eight times lower than the US-industry-standard, canister results [33,34]. This study attempted to mitigate the sorbent-tube/canister discrepancy by following US EPA recommendations for continuous sampling over 14 days, rather than point-in-time sampling that yields less reliable, not 14-day average, contaminant concentrations [32,35].

##### 2.2.1.6. Preliminary DCDFM and DBM Screening Levels

This assessment also is preliminary because of the dichlorodifluoromethane (DCDFM) screening levels used. As DCDFM has long been phased out, little/no testing continues on it, and it has no screening levels. However, the US National Academies of Science say DCDFM toxicity is comparable to that of trichloromethane (TCM); thus we employ TCM screening levels for DCDFM in our preliminary safety-benchmark studies [36]. Similarly, neither US EPA nor California DTSC provides screening levels for dibromomethane/methylene bromide (DBM). As the state of Indiana does provide them, we used Indiana ESLs for our preliminary assessment of NOTSPA DBM levels [37].

##### 2.2.1.7. A Partial Social-Media Evaluation

Finally, because the focus of this analysis is whether or not indoor-air sampling reveals that the former-NOTSPA toxic facility currently violates three safety benchmarks, we provide only a preliminary evaluation of NOTSPA assessors’/redeveloper’s social-media dissemination of their claims of hazardous-site safety. This preliminary assessment examines only one major source of evidence for the redeveloper’s safety assurances.

This major source of social-media evidence is the redeveloper’s written and spoken safety claims, made to Pasadena City Council, and transmitted to the entire community through internet/television streaming of city-council meetings <cityofpasadena.net/city-manager/pasadena-media/> (accessed on 28 March 2021), e.g., [25]. These social-media claims also are available through city audiovisual tapings, and they can be downloaded or viewed on the internet <cityofpasadena.net/city-clerk/audio-video-archives/> (accessed on 28 March 2021), e.g., [25]. Finally, these social-media claims are in the redeveloper’s documents, provided online by the city, along with the relevant council-meeting agenda <pasadena.granicus.com/ViewPublisherRSS.php?view_id=25&mode=agendas> (accessed on 28 March 2021), e.g., [25]. As the City Council is the government’s lead agency in approving the limited toxic-site assessment/cleanup proposed by the redeveloper, the council is the main target of the redeveloper’s claims regarding NOTSPA safety.

#### 2.2.2. Method 1: Sampling and Monitoring

Site indoor-air sampling followed US EPA Method TO-17, with further procedures outlined in the Beacon Technical Guide for Sampling (see the authors’ Chapter 1 in the online Supplemental Materials) and in the diffusive-sampling methods of the International Standards Organization (ISO) [38]. This first method involves installing 12 (including one duplicate) noninvasive, passive, indoor-air, thermally conditioned, sorbent-tube samplers (see authors’ Chapter 1 in the online Supplemental Materials) in order to conduct a preliminary assessment (see the authors’ Section 2.2.1) of 12 samplers targeting 6 site VOCs at 11 site locations, namely, all those in which site rental tenants gave written permission for testing (see Figure 1 and the authors’ Chapter 3 in the online Supplemental Materials). This sampling provides 72 different NOTSPA toxic-chemical measurements (6 contaminants in each of 12 samplers). As already mentioned, Maryland-based Beacon Environmental Services, Inc., the US industry leader in passive-sorbent-tube testing, supplied the samplers; see the authors’ Chapter 2 in the online Supplemental Materials.

Because earlier, limited soil-gas testing showed that carbon tetrachloride (CT), per(tetra)chloroethylene (PCE), and trichloroethylene (TCE) pose the three highest onsite cancer risks and non-cancer hazards (e.g., birth defects) [15] (Table 6), the three main target compounds sampled by our 2021 method 1 are CT, PCE, and TCE. In addition, this method targeted three additional compounds: Chloroform/Trichloromethane (TCM), Dibromomethane (DBM), and Dichlorodifluoromethane (DCDFM). Method 1 added DCDFM and TCM, as testing shows they pose, respectively, non-cancer hazards and cancer threats that are in the top-four and top-five onsite [15] (Table 6). Method 1 added DBM because it is a potent solvent, suspected to be onsite [17] (Appendix D); because it has acute inhalation toxicity, effects on the nervous system/heart/lungs after only short exposure, and effects on the kidney and liver with longer exposure [39]; and because its screening level is relatively low, only 4.2 µg/m^3^ [40].

#### 2.2.3. Method 2: Using Instrumentation for Laboratory Analysis of Samplers

Laboratory analysis of site indoor-air samples followed US EPA Method TO-15, modified by US EPA to fit the specific requirements of the more-sensitive US EPA Method TO-17, including BF3 tuning, calibration, and verification, as well as requirements for using internal standards, surrogates, duplicates, and lab-control spikes and associated compounds [32]. Procedures for implementing these requirements are outlined in the Beacon Analytical Report (see the authors’ Chapter 4 in the online Supplemental Materials) and in the methods of analysis of VOCs by diffusive sorbent tubes, thermal desorption, and gas chromatography (GC), prescribed by the International Standards Organization (ISO) [38]. These methods dictate how to desorb site-sample VOCS (collected in the sorbent tube) onto the GC column for separation, then how to analyze them by mass spectrometry [32]. Methods used for assuring laboratory quality control are also from the ISO [41].

Beacon’s method 2 analysis follows all US Environmental Protection Agency (EPA) Method TO-17 procedures. Beacon, the US leader in passive-sorbent-tube sampling, is the only US laboratory accredited for thermal desorption-gas chromatography/mass spectrometry (TD-GC/MS) instrumentation, following US EPA Method TO-17 procedures “without modifications,” in accordance with the US Department of Defense Environmental Laboratory Accreditation Program (DOD ELAP) <beacon-usa.com/> (accessed on 28 March 2021); see authors’ Chapter 2 in the online Supplemental Materials.

For method 2, Beacon reports concentration results as time-weighted averages over a two-week period. Beacon calculates these time-weighted-average concentrations by using the exposure period, target analyte mass, and the procedures detailed in ISO 16017-2 for analysis of VOCs by sorbent tubes <beacon-usa.com/services/perimeter-air-quality-monitoring/> (accessed on 28 March 2021).

For EPA Method TO-17, the limit of quantitation is 10 nanograms (ng) and the limit of detection/detection limit is 5 ng. However, method 2 reports analytical results in micrograms per cubic meter (µg/m^3^). All continuing calibration verification values must be within ±30% of the true values, as defined by the initial calibration and must meet the requirements specified in Beacon’s Quality Manual, including for surrogates, acceptance criteria, etc. All lab results must be fully certified (see the authors’ Chapter 4 in the online Supplemental Materials).

#### 2.2.4. Method 3: Comparing Beacon Results to Government Safety Benchmarks

Method 3 assesses the risk levels posed by the fully certified Beacon lab (sampling) results by comparing them to three government safety benchmarks for California indoor-air-VOC concentrations. These three benchmarks are the current health-protective, government Environmental Screening Levels (ESLs), No Significant Risk Levels (NSRLs), and site-specific inhalation risks based on the Inhalation Unit Risk (IURs) for hazardous chemicals; see the authors’ Table 3.

This comparative analysis of site indoor-air samples, relative to the three classic, toxic-site, safety benchmarks, follows methods outlined by US EPA technical guidance [35] and California DTSC technical guidance for “vapor intrusion screening evaluations” [32]. US EPA says this benchmark-comparative analysis must specify “where and how the data will be…compared to risk-based benchmarks [35] (p. 65), in order to determine potential for adverse effects, by comparing “measurements of indoor air levels of vapor-forming chemicals” to screening, “benchmark,” or “reference concentrations” [35] (pp. 126–127). Indeed, the main US EPA methodological/technical document on vapor-intrusion screening assessments calls benchmark-analysis methods one of the 14 main steps in evaluating indoor-air contamination [35] (p. 184).

##### 2.2.4.1. The First or ESL Safety Benchmark

Government designed the first (ESL) benchmark to protect against residential/workplace risks greater than 10^−6^, risks causing more than one cancer per million persons exposed over a lifetime [40,42]. Violations of these ESLs alert regulators that a safety problem could exist and therefore, at a minimum, to conduct additional testing [24,26]. The relevant ESLs include both some California-specific levels [42] and the default US EPA ESLs, otherwise mandated for assessing toxic-chemical sites [40]. As Section 2.2.1.3 explains, because of the explicit California DTSC recommendations regarding screening evaluations, we emphasize only residential, not commercial, screening levels or benchmarks [28].

##### 2.2.4.2. The Second or NSRL Safety Benchmark

The second, or NSRL, benchmark is the set of daily residential- and workplace-exposure levels that the California Office of Environmental Health Hazard Assessment (OEHHA) judges to be safe from “significant” risk; this is the 10^−5^ risk level causing no more than one cancer per 100,000 persons’ lifetime exposures [43]. For NOTSPA VOCs, the mandated NSRL is a residential daily airborne-exposure level [28] for each detected chemical [43].

##### 2.2.4.3. The Third or IUR Safety Benchmark

The third, or IUR-based, benchmark is the set of US EPA and California EPA continuous-inhalation-exposure levels that one calculates by multiplying the US EPA [45] or California EPA [43], contaminant-specific IUR (the upper-bound excess lifetime cancer risk estimated to result from continuous exposure to an agent at a concentration of 1 µg/m^3^ in air), as detected by the site-contaminant sample. For instance California EPA’s carbon tetrachloride IUR is 4.2 × 10^−5^ per µg/m^3^ (4.2 × 10^−5^) [46], while US EPA’s carbon tetrachloride IUR is 6 × 10^−6^ per µg/m^3^ (6 × 10^−6^) [47]; because the NOTSPA toxic site is located in California, one must follow California IURs.

Note that US EPA advises that when one uses the IUR for a specific contaminant “from the Integrated Risk Information System (IRIS) to characterize risk, it is not necessary to calculate the inhaled dose. This is because of the specific properties of the IUR. US EPA says it is not necessary to use its models to calculate inhalation dose–response relationships. Likewise, perhaps because its focus is regulatory, California DTSC appears to have a preference for “direct measurement of the VOCs,” rather than for modeled risks [28]. Instead of models, US EPA likewise seems to have a preference for comparison on the basis of established benchmarks, like IUR, given the many modeling assumptions that can be involved in the different potential, versus applied, versus internal, versus biologically effective doses [45]. Moreover, by definition, this study is an initial screening assessment whose general hypothesis is whether or not indoor-air samples violate three standard safety benchmarks. This study is not a dose assessment, not an exposure assessment, not an epidemiological assessment. Instead, it is an initial hydrogeochemical screening assessment for carcinogenic vapor intrusion, a preliminary screening accomplished by focused indoor-air sampling, so as to determine whether safety benchmarks are exceeded.

#### 2.2.5. Decision Criteria for the Benchmarks

Given the preceding ESL, NSRL, and IUR safety benchmarks, for toxic sites to be considered safe, California regulators require responsible parties to use the benchmarks to:

- (a) *Conduct indoor-air testing* of all “current buildings,” to fully characterize site risks (and potentially remediate/mitigate any excess risks)—if soil-gas levels exceed ESLs [26] (p. 17);
- (b) *Perform additional sampling*, to fully characterize site risks (and potentially remediate/mitigate excess risks)—if a building’s indoor-air-VOC concentrations exceed ESLs [24,26] (p. 27);
- (c) *Use the ESLs*, *NSRLs*, and IURs to ensure that each indoor-air level for each VOC is at/below its respective benchmark, e.g., [24,26,43].

Method 3 will determine whether former-NOTSPA site testing and management meet conditions (a)–(c). If all site conditions (a)–(c) are met, then CBRE/TCC safety claims to media (e.g., that “the [former NOTSPA] site is safe at this time. Contamination is below paved surfaces and confined” [25]) likely do not misrepresent site health harm, and our hypothesis is not confirmed. If any site conditions (a)–(c) are not met, then the regulator’s and CBRE/TCC’s safety claims may misrepresent site health harm, and our hypothesis may be confirmed.

All methods and procedures used in the sorbent-tube-sampling/instrumentation study are available for review, as provided by Beacon Environmental Services; see the authors’ Chapters 1 and 4 in the online Supplemental Materials. However, the US EPA and other government agencies require access agreements for any site sampling of private property; consequently, any data related to the rental tenants’ private, health-related information, or protected by the tenants’ signed Access Agreement, will not be available without the tenants’ written permission; see authors’ Chapter 3 in the online Supplemental Materials.

In addition, the authors provide a limited assessment of how the redeveloper used social media to disseminate its claims of site safety that contradict the results of our preliminary hypothesis confirmation. This limited social-media assessment covers the audio-visual streaming, internet exchanges, and internet postings of the redeveloper’s safety claims, made at Pasadena City Council meetings and transmitted to anyone with either television or internet access. This limited assessment (1) focuses only on whether the toxic-site redeveloper specifically makes claims that the toxic site is now “safe”; it (2) follows the constraints of this preliminary assessment, outlined in the authors’ Section 2.2.1.

## 3. Results

### 3.1. Results of Methods 1 and 2: Sampling and Laboratory Analysis of Samplers

For the two-week, passive, noninvasive, sorbent-tube sampler study of indoor air in rental units at the former NOTSPA, in method 1 we followed all the US EPA and California EPA methods outlined in the previous Section 2.2—especially US EPA Method TO-17, and all Beacon Environmental Services, Inc. protocols and directions for installation, monitoring, and retrieval of the samplers—then shipped them to Beacon for analysis. These procedures and the resulting deployed samplers met all Beacon protocols and requirements. All results are fully certified and from a fully certified lab.

Beacon’s method 2 analysis provides results (see authors’ Chapter 4 in the online Supplemental Materials) for 6 contaminants measured by each of 12 passive-VOC samplers at 11 locations, for a total of 72 results for different locations. The authors’ Table 3 presents the Beacon detection limits used for its analysis; the relevant benchmarks, e.g., [43], for comparison; and, also for comparison, the best detection limits used, to date, in site studies conducted earlier than this one [17,44]. As the authors’ Table 3 shows, Beacon’s detection limits are 2 orders of magnitude more sensitive than the best CBRE/TCC detection limits, used in its 2007 studies [17,44]. These Beacon detection limits are sometimes several nanograms less sensitive than the lowest current ESLs, but as explained in Section 2.2.1, beacon/we use the only US EPA-approved testing method for sorbent sampling, the method with the best-possible detection limits. As such, all Beacon detection limits are of the same order of magnitude as the lowest current ESLs; see authors’ Table 3. We focused on residential benchmarks, for reasons mandated by government and given in Section 2.2.1.3.

Of the 6 VOCs whose levels were sampled/analyzed, stricter California residential/commercial screening levels (ESLs) exist only for CT and PCE; their respective values are 0.47 and 2 µg/m^3^—and 0.46 and 2 µg/m^3^ [42]. The otherwise-required US EPA residential and commercial ESLs for TCE, and TCM, respectively, are 0.48 and 2 µg/m^3^, and 0.12 and 0.53 µg/m^3^ [40]. See Section 2.2.1.6 for an explanation of DCDFM and DBM ESLs used. The authors’ Table 4 presents results of Beacon’s Method 2, along with the relevant ESLs for comparison.

### 3.2. Results of Method 3: Beacon’s Lower, Uncalibrated Results and Safety Benchmarks

#### 3.2.1. Uncalibrated Results, Compared to the First or ESL Benchmark

Regarding the first or ESL safety benchmark, comparison of the 72 NOTSPA-site, fully laboratory certified, VOC-sampler results (12 samplers targeting 6 VOCs, each sampled at 11 locations)—from a fully certified lab—reveals that:

- all 12 PCE samples—at every location tested—violate the first benchmark, ESLs, with 7 locations above both the commercial and residential ESLs, and all 12 locations above the residential ESL. As already mentioned, California dictates use of the more protective residential ESL standards at all toxic sites [28];
- 12 of 72 VOC values had neither actual nor potential ESL violations (because of inadequately sensitive testing, despite the authors’ using the only US EPA-approved testing method for sorbent sampling; see authors’ Section 2.2.1);
- 9 of 72 VOC values violated both residential and commercial ESLs (namely, 7 PCE and 2 DCDFM results);
- 43 of 72 VOC values have possible violations of residential ESLs (namely, 11 CT, 12 TCM, 8 DCDFM, and 12 TCE values), because of inadequately sensitive test-detection limits, despite the authors’ using the only US EPA-approved testing method for sorbent sampling (see authors’ Section 2.2.1);
- Given inadequately sensitive test-detection limits in this initial screening, despite the authors’ using the only US EPA-approved testing method for sorbent sampling (see authors’ Section 2.2.1), all 12 locations should be soon retested for TCE. This is because they may require a government-mandated, accelerated-response action (within several days), owing to possible disallowed TCE levels; only a brief exposure to TCE, at roughly the screening level (ESL), can cause birth defects and other harm [48]; see authors’ Table 4.

The first or ESL benchmark also shows that for all sampled locations, even the lower, uncalibrated results violate the required PCE ESL. This residential ESL for PCE (0.46 µg/m^3^) is violated by uncalibrated results ranging between 13.4–1.43 µg/m^3^ at the 12 sampled locations. These lower, uncalibrated results correspond to a lifetime PCE risk from (2.9) 10^−5^ to (3.1) 10^−6^, roughly 29 to 3 times above the ESL. Most site samples also violate the less protective, commercial ESL; see the authors’ Table 4. However, as mentioned, California dictates use of only the more stringent residential ESL at toxic sites [28].

#### 3.2.2. Lower, Uncalibrated Results and the Second or NSRL Benchmark

To determine whether any lower, uncalibrated VOC risks are higher than the NSRLs, which (like ESLs) assume lifetime exposure, OEHHA dictates multiplying the average (Beacon) dose of a VOC (e.g., PCE at 13.4 µg/m^3^ at location B) times 10 hours (to obtain worker exposure/day), or times 20 hours (to obtain residential exposure/day). This second or NSRL benchmark of 14 shows that the lowest and highest exposure locations (respectively, samplers H and B) indicate *PCE worker uncalibrated exposures per day* are 14.3–134 µg/m^3^; this means even the lowest, uncalibrated, PCE worker risks are 102% to 957% higher than the PCE NSRL. Similarly, the second or NSRL benchmark shows that the lowest and highest exposure locations, respectively (samplers H and B), indicate that *PCE residential uncalibrated exposures per day* are 28.6–268 µg/m^3^; this means that even the lowest, uncalibrated PCE residential risks are 204% to 1,914% higher than the required NSRL. Thus, the NSRL benchmark shows that even the lower, uncalibrated PCE results, at all NOTSPA sampling locations, violate the NSRL and pose risks up to roughly 20 times higher than the 10^−5^ NSRL.

In fact, using the 10^−5^ NSRL, some site-sampling locations show that even the lower, uncalibrated, residential risks are above 10^−4^. At the *highest-exposure site-sampling location (B)*, the lower or uncalibrated residential risk is 1.9 (10^−4^); PCE at sample-location B = 13.4 µg/m^3^ and CT = 0.679 J. Similarly, at the *lowest-exposure site-sampling location (H*), the lower or uncalibrated PCE risk is 2.0 (10^−5^); PCE at sample-location H = 1.43 µg/m^3^.

#### 3.2.3. Lower or Uncalibrated Results and the Third, nor IUR-Based, Safety Benchmark

To determine whether any lower or uncalibrated VOC risks are higher than the IUR-derived inhalation risks (which, like ESLs and NSRLs, assume lifetime exposure) [49], recall from the author’s Section 2.2.4.3 that one must multiply the contaminant-specific IUR by the site-sample-detected contaminant level. For instance, because California EPA’s carbon tetrachloride (CT) IUR is 4.2 × 10^−5^ per µg/m^3^ (4.2 × 10^−5^) [46], and the highest detected site CT level (µg/m^3^) is 0.679 J (see authors’ Table 4), therefore the lower or uncalibrated CT inhalation risk is 2.9 × 10^−5^ or 29 times higher than the PCE NOTSPA acceptable cancer risk of 10^−6^ [15,19].

Using the same formula, at all locations sampled, even the lower or uncalibrated PCE inhalation-risk values violate the IUR-based benchmark because they range from 8.2 (10^−5^) to 8.7 (10^−6^). Note that the highest IUR-based risk (for the lower or uncalibrated PCE levels) is 82 times above the PCE NOTSPA acceptable cancer risk of 10^−6^ [15,19].

#### 3.2.4. Lower, Uncalibrated Results and All Three Government Safety Benchmarks

Based on the previous NOTSPA studies and the ESL, NSRL, and IUR safety benchmarks for toxic-site exposures, method 3 reveals likely safety threats to current site renters, as the former NOTSPA appears to meet none of the three government safety benchmarks for vapor-intrusion sites. That is (see preceding Section 2.2.3), method 3 shows that for toxic sites to be considered safe, government says responsible parties should (a) *conduct indoor-air tests* of all “current buildings,” if soil-gas levels exceed benchmarks [26] (p. 17). 

However, the authors’ Table 1 and Table 2 earlier showed that not only do the redeveloper’s latest, 2007 site soil-gas levels exceed ESLs, but both PCE and CT are up to hundreds of thousands of times above these ESLs. Yet, the responsible parties, including CBRE/TCC, conducted no cleanup since they learned in 2007 of these high soil-gas levels. Because they appear to have violated government safety criterion (a), they likely misrepresent site safety when they claim to media (that “the [former NOTSPA (Space Bank)] site is safe at this time. Contamination is below paved surfaces and confined” [25]. This claim ignores VOC vapor intrusion, which (as our test results show) clearly is not confined.

In addition, preceding Section 3.2.1 showed that 7 of 12 of our 2021 site PCE indoor-air lower, uncalibrated results even violate the worker ESL, part of the first safety benchmark; that no prior indoor-air sampling was done; and that these lower, worker-ESL violations range up to 7 times above what is allowed. Yet, Section 2.2.3, method 3, shows that for toxic sites to be considered safe, responsible parties also should (b) *perform additional sampling*, to fully characterize site risks (and potentially remediate/mitigate excess risks), if a building’s indoor-air-VOC concentrations exceed ESLs [24,26] (p. 27). 

However, because no one ever conducted prior onsite, indoor-air tests, no one can be sure the site is safe. At best, the site-safety situation is unknown; at worst, our sampling shows that even the lower, or uncalibrated, results pose vapor-intrusion risks and violate government safety guidelines. The only way that CBRE/TCC, or anyone else, can make site-safety claims is after having jointly sampled both subslab soil gas [27], and indoor air tests for all buildings, just as guidance requires [26] (p. 17). However, CBRE/TCC tested neither subslab soil gas under 86% of site buildings, nor any site indoor air [17]. Therefore, at best, CBRE/TCC has represented an unknown situation as a safe situation. As already shown, because the state requires additional testing—given even our lower, or uncalibrated, indoor-air test results, CBRE/TCC appears to misrepresent the site situation when it makes NOTSPA safety claims. 

Besides violation of safety criteria (a)–(b) above, the authors’ Section 3.2.2 and Section 3.2.3, respectively, also show that the uncalibrated results, from every NOTSPA location sampled, violate the required residential ESLs, NSRLs, and IUR-based benchmarks. Yet, according to preceding Section 2.2.3, method 3—and the ESL, NSRL, and IUR-based benchmarks—for toxic sites to be considered safe, responsible parties should (c) use the benchmarks to ensure that indoor-air-VOC levels are at/below all these benchmarks [43]. 

However, our sorbent-tube sampling shows that even these lower or uncalibrated levels are not at/below all benchmarks. That is, some CT and DCDFM, and all PCE, uncalibrated levels violate the ESL at all locations sampled; some CT, and all PCE, uncalibrated levels violate the NSRL at all locations sampled; and some CT, and all PCE, uncalibrated levels violate the IUR-based benchmark at all locations sampled. These violations are a result of the fact that not only did CBRE/TCC not ensure that, or test whether, site indoor-air VOC levels are at/below benchmarks, but instead CBRE/TCC repeatedly represented this unknown situation as a safe situation. In summary, CBRE/TCC appears to have violated government-safety criteria (a)–(b), yet to have misrepresented toxic-site safety at the former NOTSPA (Space Bank) site, especially the safety of current renters.

### 3.3. Preliminary Assessment of the Redeveloper’s Social-Media Claims of Site Safety

Recall that the hypothesis to be tested in this study is contrary to the safety claims of one of the world’s largest redevelopers of hazardous-waste facilities (CBRE/TCC Crow). This hypothesis is that the authors’ university-based, screening assessment, especially its lab-certified, toxic-site, indoor-air samples, show violations of all three prominent government, cancer-safety benchmarks, at all NOTSPA locations sampled. Regarding this hypothesis, how did the authors find/select safety claims by CBRE/TCC about a prominent toxic site that it is assessing/redeveloping? As the authors’ Section 2.2.1.7 and Section 2.2.3 indicate, their preliminary assessment of social-media evidence for the redeveloper’s safety assurances is limited to only one major social-media source.

This major social-media source is the audio-visual streaming, internet exchanges, and internet postings of the redeveloper’s safety claims, made available online and through television, by the City of Pasadena. The authors’ limited assessment (1) focuses only on whether the toxic-site redeveloper specifically makes claims that the toxic site is now “safe,” and (2) follows the constraints of this preliminary social-media assessment, outlined in the authors’ Section 2.2.1.7.

Using the preceding procedures, the authors found a CBRE/TCC claim of toxic-site safety that appears to be the most direct, explicit, and often-repeated assertion: “The site is safe at this time. Contamination is below paved surfaces and confined” [25]. This social-media claim was made in at least three different circumstances: during internet/television streaming of (Pasadena, California) city-council meetings <cityofpasadena.net/city-manager/pasadena-media/> (accessed on 28 March 2021); on city audiovisual tapings, that can be downloaded or viewed on the internet <cityofpasadena.net/city-clerk/audio-video-archives/> (accessed on 28 March 2021); and within the redeveloper’s official documents, provided online by the city as part of each council-meeting agenda <pasadena.granicus.com/ViewPublisherRSS.php?view_id=25&mode=agendas> (accessed on 28 March 2021), e.g., for 9 July 2019. [25]. Because the Pasadena City Council is the government’s lead agency in approving the limited NOTSPA toxic-site assessment/cleanup proposed by the redeveloper, the council is the main target of the redeveloper’s claims regarding NOTSPA safety.

For five reasons, the authors focused only on City of Pasadena, social-media sources. (1) This initial social-media assessment is limited and preliminary (see the authors’ Section 2.2.1) and is not part of assessing the main hypothesis of this paper, and (2) the authors are not social-media experts. In addition, (3) the city is the lead agency for the toxic site, and the city is the government entity that the redeveloper most needs to convince that the toxic site is safe; and (4) this city internet site, including its postings/updates and streamed city-council meetings/exchanges, is the main place where city residents go for quick, up-to-date information about what city government is doing regarding the toxic site and other critical city issues. Finally, the authors focused on city-sponsored websites to discover toxic-site, social-media claims because (5) it is very difficult to find non-city-related, social-media claims by redeveloper CBRE/TCC, given a specific corporate policy. This corporate policy is that of creating individual, different, limited liability corporations (LLCs) at each of the many toxic sites that CBRE/TCC assesses/remediates/redevelops. Thus, the LLC for the Pasadena toxic site is “Pasadena Gateway.” Attorneys confirmed that unless one knows, ahead of time, the names of all LLCs, controlled by CBRE/TCC, it is impossible to find them all, because each name is different. Using LLCs keeps the major company from having its $billions of assets at risk at every toxic site; it also makes it difficult to track, by name, the overall corporate behavior of the parent company.

### 3.4. Interpretation of Passive Sorbent-Tube Results from Indoor-Air Sampling

Previous sections show that because—at all sampled locations—even the lower, uncalibrated results of our passive-sorbent-tube samplers violate ESL, NSRL, and IUR-based safety benchmarks, they raise questions both about whether CBRE/TCC and the state regulators accurately represent former NOTSPA safety in their claims about the site. Our sampling results also heighten the controversy over whether CBRE/TCC should have followed state guidance that mandates indoor-air testing. However, for at least six different reasons, discussed in subsequent paragraphs, our lower or uncalibrated, passive-sorbent-tube samples likely under-represent or underestimate the real site VOC levels and risks.

#### 3.4.1. Interpretation of Results Using Beacon Sorbent-Tube General Calibration

A *first reason* that our uncalibrated results likely underestimate site risks is that Beacon says its standard samplers—that we used—provide “at a minimum, *biased low results*” [50] [italics/underline theirs]. This Beacon warning is consistent with its own published studies showing that when Beacon calibrated its Passive Sorbent Samplers, in terms of the much-more-expensive, industry standard for soil-gas sampling (stainless-steel Summa Canisters), all 36 Beacon sorbent-tube samples showed “consistently lower” VOC concentrations than the reference-standard canister results; all canister concentrations were 155–231% above the Beacon passive-tube results [33]. Other, 2016, non-Beacon calibration publications show canister results up to 816% higher than passive, sorbent (Tenax TA, Carbopack)-tube results (120 samples) for the same locations/times [34].

What happens if we apply Beacon’s general-calibration numbers (a canister increase of 1.55–2.31 over sorbent-tube VOC concentrations) to the 12-sampler Beacon results for PCE at the former NOTSPA (1.43–13.4 µg/m^3^)? The Beacon general-calibration interpretation of results for the same 12 samplers at 11 locations/times are (2.22–3.3 µg/m^3^) to (20.77–30.95 µg/m^3^), or 2.22-30.95 µg/m^3^, lowest to highest; see the authors’ Table 5.

As Table 6 shows, if one employs the residential ESLs, as recommended by the California regulator for all toxic-site screenings (see Section 2.2.1.3) [28], then ESL residential PCE risks are up to 67 times higher than the allowed 10^−6^ risk. Even the lowest or most lenient average NOTSPA residential risks (generally calibrated) violate the residential ESLs at every NOTSPA sampling location that was sampled. For instance, using the first or ESL safety benchmark, the PCE residential ESL (0.46 µg/m^3^ = 10^−6^ risk), and the highest (at location B) and lowest (at location H) generally calibrated PCE concentrations, Table 6 indicates the *highest to lowest average PCE residential risks* are 6.7 (10^−5^) to 4.8 (10^−6^) µg/m^3^, respectively.

In fact, although the California regulator does not recommend employing the workplace or commercial ESLs, because they are inadequately protective (see Section 2.2.1.3) [28], even the generally calibrated NOTSPA commercial PCE levels are up to 15 times higher than the allowed 10^−6^ risk. In fact, even the lowest or most lenient average NOTSPA workplace risks (generally calibrated) violate the commercial ESLs at every NOTSPA location that was sampled. For instance, using the first or ESL safety benchmark, the PCE workplace ESL (2 µg/m^3^ = 10^−6^ risk), and the highest (at location B) and lowest (at location H) PCE generally calibrated PCE concentrations, then the *highest to lowest average PCE commercial risks* are 1.5 (10^−5^) to 1.1 (10^−6^) µg/m^3^, respectively. 

Thus, in summary, as the authors’ Table 6 shows, both all uncalibrated PCE samples, at every location sampled, and all generally calibrated PCE samples, at every location sampled, violate ESL safety benchmarks. In addition, some uncalibrated CT and DCDFM samples and some generally calibrated CT and DCDFM samples also violate ESL safety benchmarks. Indeed, even the lower or uncalibrated risks of the highest PCE samples are up to 29 times higher—while the corresponding generally calibrated samples are up to 67 times higher than this ESL safety benchmark; see authors’ Table 6.

As the authors’ Table 7 shows, when one checks the preceding Beacon-general-calibration data against the required residential NSRL or second benchmark, the highest PCE risks at NOTSPA are up to 44 times above the NSRL—a significant threat. Using the PCE NSRL (14 µg/m^3^/day = 10^−5^ risk), the highest and lowest ranges of PCE *average residential exposures, respectively,* are (31.0–20.8 µg/m^3^) (20) or 620–416 µg/m^3^/day for highest exposures and (3.3–2.2 µg/m^3^) (20) or 66–44 µg/m^3^/day for lowest exposures. That is, 620 µg/m^3^/day and 44 µg/m^3^/day, respectively, at NOTSPA-sampling-locations B and H, represent the highest and lowest ends of the range of generally calibrated PCE exposures, respectively. This OEHHA PCE residential-exposure range corresponds to a risk range between 4.4 (10^−4^) µg/day and 3.1 (10^−5^) µg/day. Indeed, even the uncalibrated risks of the highest PCE samples are 19 times higher than this second or NSRL safety benchmark, and the corresponding generally calibrated samples are more than double that uncalibrated risk.

Likewise, as already mentioned (Section 2.2.1.3), the State of California does not recommend use of commercial or workplace safety benchmarks for toxic sites because they are inadequately protective [28]. However, even when one uses the PCE NSRL (14 µg/m^3^/day = 10^−5^ risk) and the commercial (10-h/day) NSRL, the NOTSPA generally calibrated PCE exposures reveal a risk up to 22 times above the commercial NSRL.

That is, the highest to lowest PCE *average workplace exposures* per day are 310 µg/m^3^ and 22.0 µg/m^3^, respectively, at NOTSPA-sampling-locations B and H. This commercial/workplace-exposure range corresponds to a risk between 2.2 (10^−4^) and 1.6 (10^−5^). Thus samples, at all locations tested, violated even the more lenient commercial NSREL safety benchmark

Similarly, as the author’s Table 7 shows, when one checks the Beacon-general-calibration data against the residential NSRLs, as recommended by the state [28], both the lower or uncalibrated CT result of 0.679 J µg/m^3^ and the more realistic or generally calibrated CT results of 1.1–1.6 µg/m^3^ violate both the commercial and the residential NSRL of 5 µg/m^3^ per day (=10^−5^ risk). The highest to lowest range of generally calibrated CT *average residential exposures* are (1.6–1.1 µg/m^3^) (20) or 32.0–22 µg/m^3^ per day. This CT residential-exposure range corresponds to a risk range between 6.4 (10^−5^) µg/day and 4.4 (10^−5^) µg/day. Even the risk from the lower or uncalibrated CT residential (13.6 µg/m^3^ per day) concentration at NOTSPA is roughly three times higher than the NSRL for CT. 

Finally, as Table 8 shows, when one assesses generally calibrated sampling results against the third or IUR-based safety benchmark and the CT IUR, namely, (4.2) 10^−5^ risk, CT cancer-inhalation risks from NOTSPA are as high 67 times above the allowed level. The highest and lowest CT *average inhalation risks*, respectively, are (6.7) 10^−5^ and (4.6) 10^−5^, given Beacon’s general calibration. 

For PCE, Table 8 reveals that even the lower or uncalibrated inhalation cancer risks of the highest PCE samples are 82 times higher [(8.2) 10^−5^] than the 10^−6^ NOTSPA safety benchmark. The inhalation cancer risk of corresponding generally calibrated PCE sample is roughly 190 times higher than this benchmark. Thus, as the authors’ Table 8 shows, all the uncalibrated and generally calibrated PCE samples, at all locations sampled—and all the generally calibrated CT samples with detections—violate IUR-based safety benchmarks.

Of course, the preceding results and interpretations are based on two main assumptions. The first assumption is that the renter has lifetime exposure at the level indicated. The second main assumption is that it is reasonable to apply Beacon’s own general calibration interpretation, given the absence of site-specific calibration. However, because the sorbent-tube results are preliminary (see the authors’ Section 2.2.1), meant merely to assess the need for additional site indoor-air and soil-gas testing (and potential mitigation/remediation), they succeed because at every location sampled, even the uncalibrated results violate all three safety benchmarks. 

To summarize, even the uncalibrated results—at every location sampled—show clear violations of the ESL, NSRL, and IUR-based safety benchmark. (see, respectively, the authors’ Table 6, Table 7 and Table 8). As such, this sampling shows that both uncalibrated and calibrated sorbent-tube results, at every location tested, show the need for full-site, indoor-air and soil-gas testing, both because testing guidance mandates it [26], and because existing rental tenants deserve protection.

#### 3.4.2. Interpreting Results, Given Average Exposures and Barometric Pressure

A *second reason* that our sorbent-tube results may underestimate site-toxin exposures is that they represent only two-week-average exposures for each of 12 samplers, targeting 6 different VOCs, for each of 11 sampling locations. As averages, these Beacon-reported concentrations miss the highest or peak exposures—that could be an order of magnitude above average. Yet, as already mentioned in Section 3.2.1, for some VOCs, even very short-term exposures, e.g., at only 0.5 µg/m^3^ TCE, could cause serious harm, including birth defects in unborn children; that’s why government mandates accelerated (within several days) reduction of VOC exposures in situations where TCE concentrations, for instance, are as low as 0.5 µg/m^3^ [48]. Thus, because averages do not reveal the entire story, only continuous, indoor-air and sub-slab testing, for all onsite buildings, could provide exposure data that clearly are not underestimates [48].

A *third reason* that our sorbent-tube results may underestimate site exposures is that during the two weeks of site testing, there were great fluctuations in barometric pressure. Given these fluctuations, the highest or peak levels of VOCs could be much higher, even urgent-action, levels [48]. As the latest draft vapor-intrusion guidance from US EPA [35] (pp. 24–25) and California DTSC [26] (p. 3) warn, whenever interior-building air pressure is, respectively, lower (or not) than that of the subsurface environment, including because of fluctuations in barometric pressure (as occurred during our testing), advection (transport from high to low pressure areas) is more (or less) likely to transport carcinogenic vapors from the soil or groundwater to indoor air via cracks or other openings [26,35].

#### 3.4.3. Interpreting Results, Given No Building HVAC, Wind, and Toxin Sources 

A *fourth* reason that our sorbent-tube results may underestimate gaseous VOC exposures is that former NOTSPA (Space Bank) buildings have no central HVAC. Yet, heating systems promote vapor intrusion into buildings through the wintertime “stack effect”; heating reduces internal air pressure and creates a vacuum effect that enhances advective flow from underlying soils and/or groundwater into buildings [26,35]. Due to this stack effect, and given no NOTSPA heating systems, therefore any NOTSPA renter who uses space heaters (to keep his unit warm during winter) may have higher indoor-air-VOC exposures that are higher than what we measured in our 2021 university-based sampling.

*Fifth*, our results also may underestimate site-exposure risks because strong winds reduce internal air pressure, cause a vacuum, and promote vapor intrusion through advection [35] (p. 28). However, during our 2 weeks of indoor-air sampling, there were no typical strong winds. Although average site wind speed is 12.7 mph (usa.com/rank/california-state--average-wind-speed--city-rank.htm; accessed on 20 March 2021), during our testing, average wind speed was only 3.3 mph. As a result, during our 2 weeks of sampling, wind-induced advective vapor intrusion likely was less than is normal/typical/average onsite.

*Sixth*, our results likewise may underestimate rental tenants’ exposures because indoor-air, vapor-intrusion risks are highest, the closer they are to contaminant sources. Yet, most NOTSPA contaminant sources are unknown [15] (Appendix A), [18]. Given limited soil-gas sampling, CBRE/TCC assessors were able to locate sources and related isoconcentration maps for only 2 (PCE and CT) of roughly 35 site contaminants of concern; for those 2 toxins they located CT and PCE sources only at 5 and 15 feet [15] (Tables 9–12). Moreover, none of the sorbent-tube samples was located above the known 5-foot and 15-foot PCE and CT sources; as a result, indoor-air sampling, elsewhere onsite, likely would identify higher VOC levels and renter exposures [15].

For all the preceding reasons, we draw two *experimental conclusions.* First, both our uncalibrated and generally calibrated results, for all sampling locations, reveal indoor-air-VOC risks that violate all three government safety benchmarks (namely, ESLs, NSRLs, and IUR-based levels). Second, for the preceding reasons, this sampling likely underestimates site indoor-air exposures.

## 4. Discussion

Preceding results show that our NOTSPA indoor-air sampling not only reveals violations of three major government-safety benchmarks that we considered in this analysis, namely, ESLs, NSRLs, and IUR-based levels (Section 3.2.1, Section 3.2.2 and Section 3.2.3), but also likely underestimates indoor-air exposures of site renters because, among six key factors, passive, sorbent-tube samplers are “biased low,” and weather during the two-week testing period was not conducive to the highest vapor intrusion (Section 3.4). Evaluation of our sampling in terms of three government-mandated, safety-guidance criteria (Section 3.2.4) also shows that the former NOTSPA site fails to satisfy these three criteria. That is, NOTSPA responsible parties, including CBRE/TCC, (a) *failed to conduct* *required indoor-air testing*, (b) *failed to perform* *additional sampling* of soil gas, to characterize full site risk, including tenant risks, and (c) *failed to use the ESLs, NSRLs and* IUR-based levels to ensure that indoor-air-VOC levels were not causing harm. For all these reasons, the former NOTSPA site cannot be considered safe for renters.

In addition, as already mentioned, both CBRE/TCC and the state regulator, DTSC, (1) failed to conduct either required groundwater or indoor air testing [26,27]; (2) failed to perform required sub-slab-soil-gas testing under 86% of site buildings [26,27]; and (3) failed both to take account of the indoor-floor-concrete cold joints and broken slabs, and to take account of the many open floor drains, sewer lines, and utility penetrations into the indoor concrete slabs—all of which allow vapor intrusion [26,27]. Instead, contrary to CBRE/TCC’s own site documents (clearly showing site diagrams of building floor drains and sewer lines that allow subsurface vapor intrusion into above-ground buildings) [15], CBRE/TCC and the regulator merely repeatedly claimed: “The site is safe at this time. Contamination is below paved surfaces and confined” [25]. Due to CBRE/TCC’s repeated, questionable reaffirmation of site safety, it arguably misrepresents health harm at some toxic sites that it remediates/redevelops. Thus, at least at the former NOTSPA site, our preliminary sampling and analysis (see the authors’ Section 2.2.1) appears to confirm our hypothesis that—contrary to the safety claims of the world’s largest commercial developer (CBRE/TCC)—the authors’ screening assessment, especially its lab-certified, toxic-site, indoor-air samples show violations of all three prominent government, cancer-safety benchmarks.

Given the preceding results and our preliminary hypothesis confirmation of our hypothesis for the former NOTSPA site, at least five questions come to mind:

4.1 What are the limits of this analysis?
4.2 How do our results compare to the results of previous studies?
4.3 How persuasive are the main objections to the apparent failure to provide a safe site for NOTSPA rental tenants?
4.4 What future research directions do these preliminary results suggest?
4.5 What future policy directions do these preliminary results suggest?

### 4.1. First Question: Limits of This Analysis

This analysis has at least 12 limitations, most of which are a result of following the California EPA’s methodological dictates for an initial screening assessment [26,27], rather than a full-scale study that is possible only after such screenings have been completed (see earlier Section 2.2.1 in this paper.) *First*, this analysis is limited in that, as an initial-screening assessment of a facility whose range of onsite indoor-air contaminants is unknown, it conducts a focused sampling of only toxic-site indoor air (partly because such sampling has never been done and is directly critical to human health). As such, it is not a full-scale, final study that includes all data from all exposure pathways.

*Second*, the study is limited in that it assesses sampling results only in terms of three major cancer benchmarks, namely, ESLs, NRSLs, and IURs, that appear to be the most prominent for the state of California. However, one also could assess results in terms of less well known, non-cancer benchmarks. *Third*, as required in initial screening assessments [26,27], the indoor-air sampling is focused, rather than grid-based/random, thus provides no statistically robust, full-site dataset; no quantitative human health risk assessment; and no epidemiological assessment. This is partly because the authors have no full-site-access permission for testing (see the authors’ Chapter 3 in the online Supplemental Materials). Nevertheless, even this limited sampling reveals that every location sampled violates all three site-safety benchmarks, a fact that guidance says is more than sufficient to confirm our hypothesis and to require further site testing [26,27].

*Fourth*, we conducted no guidance-recommended [26,27], pairwise, soil-gas sampling, given no permission from the site owner for such invasive tests. *Fifth*, our study is limited in emphasizing only residential-site benchmarks, as dictated by the California regulator, DTSC [28]. *Sixth*, the indoor-air analysis is limited to five of six already-known contaminants, recognized to have site concentrations that make them among the worst five toxins at the facility [15]. *Seventh*, owing to the working-area constraints of the site renters and the recommendations of the sample manufacturer, the continuous sampling lasted only two weeks. However, our university-based tests were longer than any other tests onsite, all of which (says government technical guidance) are much less reliable because they were single, grab-sample tests that are not representative [32,35]. 

*Eighth*, the testing was limited in that, (1) although it uses the only sampling/analysis method approved by US EPA for sorbent tubes, a method with the highest reporting and detection limits of all methods [32], the testing is not perfect. That is, (2) although our study’s lab analysis is provided by the top US sorbent-tube manufacturer and laboratory [32] (see the authors’ Chapter 2 in the online Supplemental Materials); and although our detection and reporting limits are two orders of magnitude better than all previous site studies [15], nevertheless some of our study’s detection/reporting limits are several parts per trillion above the current ESLs (see the authors’ Table 3).

*Ninth*, our study is limited in providing only uncalibrated and generally calibrated (by the sampler manufacturer), not site-calibrated detections. This is because of lack of permission for onsite testing using the large, stainless-steel canisters; as a result, sample detections underreport site-contaminant concentrations [33,34]. *Tenth*, our study employs dichlorodifluoromethane (DCDFM) screening levels based on US National Academies of Science recommendations, as government provides none [36].

*Eleventh*, our study uses state of Indiana ESLs for dibromomethane/methylene bromide because neither US EPA nor California DTSC provides them [37]. *Twelfth*, our study’s social-media assessment (of the redeveloper’s safety claims) is limited to what is found on City of Pasadena websites, television streaming, and online exchanges, for the reasons already given in the authors’ Section 2.2.1.7 and Section 3.3. Again, despite these limitations, even this preliminary testing reveals that all site samples violated all three site-safety benchmarks, a fact that is more than sufficient to confirm our hypothesis and require additional site testing and perhaps mitigation/remediation to protect current site renters [26,27].

### 4.2. Second Question: Comparison with Previous Results

Given our sampling that challenges former NOTSPA safety and suggests that interested parties, site redevelopers, likely misrepresent toxic-facility safety, how do these results compare with those of earlier relevant studies? As already mentioned in Section 1.2, to our knowledge, no one has conducted proactive, independent (from government/special interests) sampling to test whether semi-privatized hazardous-site testing/remediation actually satisfies the health-and-safety claims that private interests often make on its behalf. There likely has been little independent sampling because of two main obstacles.

As noted earlier, the first obstacle is how to fund independent sampling. We addressed this problem by using much less expensive (than large steel canisters), sorbent-tube samplers. The second obstacle to independent testing is how to obtain permission for sampling, given that its results could open the toxic-site owner to personal-injury, trespass, and endangerment lawsuits. We addressed this second problem by obtaining permission for noninvasive, indoor-air sampling from site rental tenants, the only permission necessary—and also by using small, inconspicuous, passive, indoor-air, sorbent-tube samplers that are much more easily handled and accommodated than large steel canisters.

Despite the absence of independent toxic-site testing, however, our preliminary (see the authors’ Section 2.2.1) conclusion (that CBRE/TCC appears to disseminate health misinformation about the safety of its semi-privatized, toxic-site testing/remediation) is consistent with some government data on semi-privatized assessments and cleanups. For instance, as already mentioned, although the state of Massachusetts partially evaluates only a small percentage of its toxic sites that have undergone “completed,” semi-privatized testing/remediation, an average of only 29% of “completed” privatized cleanups pass this evaluation and have no major safety problems [4]. During many years, this “passing-grade” rate drops to only about 13% of all “completed” privatized sites. As the Massachusetts assessment/remediation standards and requirements have a long history of being clearly articulated, the state’s apparent, average, annual, toxic-site failure rates of 71% suggest that special interests may have financial conflicts of interest and may attempt to use flawed/incomplete testing and remediation to reduce their own costs [4,51].

Flawed/incomplete assessment and cleanup, like that in Massachusetts, likewise characterizes the CBRE/TCC case and may drive the apparent NOTSPA misinformation. As already noted in Section 1.2, the literature shows that CBRE/TCC appears to have claimed site safety, yet conducted studies on least four of its current hazardous facilities that failed preliminary data-quality analysis [10]. The scientific literature also shows that, besides the problems outlined in this analysis, NOTSPA toxic-site testing failed a scientific data audit [11], as well as a data usability evaluation [12]. This article adds to that existing literature and supports it.

### 4.3. Third Question: Assessment of Main Objections to Our Results

As our investigation assesses only one toxic site, the former NOTSPA, at least two main objections to our results may arise. First, are these results important if they concern only one site and may not reveal a multi-site pattern? Second, why should CBRE/TCC clean up the toxic facility now, to protect renters, when it claims that it will do so in several years, after it purchases the site?

#### 4.3.1. Is There a Pattern of Misinformation?

Perhaps the most basic question about these results is that because they concern only one case, the former NOTSPA, why are these results significant? To show deliberate harm, does one need to show a pattern of misinformation at multiple sites?

Although these empirical sampling results address only one site, they are significant and illustrate a consistent pattern of harm for several reasons. *First*, our hypothesis testing shows that toxic facilities may put site renters at risk, require additional testing/remediation, and reveal problems with privatized hazardous cleanup. After all, the site we sampled is a former US military, weapons-testing facility, among the most dangerous of all toxic sites. Yet the responsible parties, including CBRE/TCC, repeatedly misrepresented its safety. 

*Second*, as already mentioned, CBRE/TCC claims to be the “industry leader” in toxic-site redevelopment, the world’s largest/wealthiest commercial developer. As such, CBRE/TCC is both a trend-setter for other remediators/redevelopers and also a company with a “standard way” of handling its many toxic-site projects. If so, NOTSPA may be a typical CBRE/TCC project.

*Third*, these results are significant, both because the main victims of toxic sites include disproportionate numbers of children, minorities, and poor people [12], and because of the great potential for harm. At least one quarter of the known US inventory of hazardous facilities (nearly 400,000) has caused or could cause public exposure to carcinogenic vapor intrusion to thousands of people [52]. Indeed, there are many reported victims because of misinformation about flawed toxic-site testing and cleanup, from the US Navy shipyard at Hunter’s Point in San Francisco [53,54,55]; to the Amphenol industrial property in Franklin, Indiana [56,57] to the W.R. Grace site in Woburn, Massachusetts [58,59,60]; to the US Marine Corps base at Camp LeJeune, North Carolina [61]; to the Jordan Downs project in Los Angeles [62,63].

*Fourth*, the apparent pattern of misrepresentation at the former NOTSPA also is significant because it is consistent with a pattern of CBRE/TCC misinformation and misrepresentation that has occurred both at other CBRE/TCC sites and with respect to other NOTSPA scientific methods and results, besides those discussed here. For instance, earlier research shows that at four different, current CBRE/TCC hazardous-cleanup and redevelopment facilities—namely in Canoga Park, Boyle Heights, Monrovia, and Pasadena, all in California—there have been misrepresentations about the quality of the toxic-site testing, as already mentioned. At three of these sites, CBRE/TCC violates 9 of 10 data-representativeness standards promulgated by the United States government; at the fourth site, the company violates all 10 data-representativeness standards [10]. In addition, earlier research shows that CBRE/TCC’s published conclusions about NOTSPA contamination contradict its own source data [11]; moreover, CBRE/TCC’s testing data for NOTSPA fail to include all the required data specified in CBRE/TCC’s own project description [12].

*Fifth*, results, for even this one NOTSPA site, also are significant because they show that a repeated pattern of CBRE/TCC misinformation has continued at NOTSPA, not merely a single instance of misrepresentation. In addition to material presented earlier in Section 1.2, Section 1.3 and Section 1.4, consider additional examples of CBRE/TCC NOTSPA safety misinformation, one about contaminant concentrations, and the other about site-cleanup levels.

*Regarding misinformation about NOTSPA contaminant levels, as already mentioned,* CBRE/TCC’s own 2007 testing shows carcinogenic-solvent VOCs, that cause vapor intrusion, are nearly a million times above allowed levels [17]; see the authors’ Table 1 and Table 2. NOTSPA VOC contaminants are also above the level at which government allows merely site mitigation. Instead, for such high levels of contaminants, government requires excavation/remediation of these VOCs [11,27]. However, instead of admitting the severity of NOTSPA VOC levels, in repeated official submissions to government, briefings to the press, news reports, and electronic communications, CBRE/TCC has maintained that the former NOTSPA has “low level” carcinogenic VOCs in soil [15] (p. 41).

*Regarding misinformation about its NOTSPA cleanup levels*, beginning at least in 2009, CBRE/TCC began privately negotiating with the state to obtain both reduced NOTSPA cleanup levels and toxic-site-liability protection. As a result, in 2011 (amended 2017) the state regulator, DTSC, signed a “Covenant Not to Sue” with CBRE/TCC. Under this DTSC agreement, CBRE/TCC received site-liability protection; was required to remove only 11 NOTSPA localized metals hotspots/drains, only as deep as 20 feet; was not required to conduct any indoor-air testing or any pre-remediation or pre-construction groundwater testing; was allowed to leave most site VOC contaminants “in place” onsite (including high VOC levels that, DTSC says, require excavation/remediation) [27]; and was allowed to employ land-use controls, given CBRE/TCC’s leaving most toxins onsite [22,23].

Consistent with its preceding 2009–2017 negotiations/agreements with the state regulator, CBRE/TCC’s public documents show that it will not fully clean up the former NOTSPA. Instead, it will leave most site VOCs “in place” onsite, [15] (Appendix A), above regulatory levels [19]. Instead of full cleanup, site documents also show that CBRE/TCC can use land-use controls and put under buildings a thin plastic liner to “limit…vapor intrusion” [15] (Appendix A), [19] (p. 48). CBRE/TCC’s site documents likewise explain that it is not cleaning up most site VOCs because doing so would be “costly and time-intensive” [19] (p. 47). 

**Figure 3:**
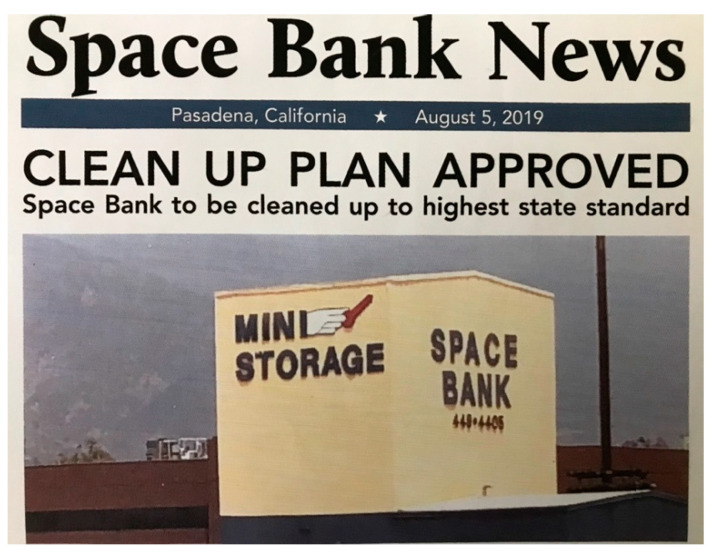
Photo of the first page of a CBRE/TCC brochure, mass mailed to thousands of Pasadena, California residents, that contradicts the cleanup standards contained both in the official CBRE/TCC site documents and in the Covenant Not to Sue that CBRE/TCC negotiated with the state regulator.

Yet, two years later, in 2019, CBRE/TCC admitted none of these preceding facts from its own documents. Instead it claimed, in an expensive, eight-page, color brochure that the Space Bank toxic site would “be cleaned up to highest state standards” (see the authors’ Figure 3). Obviously the mass-mailed brochure seriously misrepresents the contract-based level of cleanup and its safety.

Clearly CBRE/TCC is not cleaning up the site to highest state standards. Instead, consistent with its own approved site documents and its preceding contracts with DTSC, CBRE/TCC received DTSC approval, for instance, to leave building-subslab tricholorethylene (TCE) at levels up to 12,400 µg/m^3^ [19] (p. 37). However, the highest or most protective state standard allows only 0.48 µg/m^3^ TCE [17]. Thus, CBRE/TCC’s planned cleanup level is 25,833 times (that is, 12,400/0.48) less protective than the highest or most protective state standard.

Yet, instead of admitting these already documented/approved, weakened-cleanup levels in its own official technical documents, CBRE/TCC misrepresents the situation. It mailed its expensive color brochure to thousands of Pasadena residents, as shown in the authors’ Figure 3.

CBRE/TCC also repeatedly made this highest-cleanup-standards claim to the public, at multiple Pasadena City Council meetings and on electronic media. For instance, at the audio-recorded and TV-broadcast Pasadena City Council meeting of 9 July 2018, before the city approved CBRE/TCC’s site cleanup/redevelopment, CBRE/TCC officials said “The project will also safely clean up the site to the highest applicable regulatory standards…. [and] will not allow any development to occur on the site unless it’s cleaned up to the highest residential standards” [64]. Although there are many other examples, similar to those in the preceding paragraphs, the instances above give a sense of the way CBRE/TCC appears to spread health misinformation through both traditional and social media.

#### 4.3.2. Why Should the Site Be Tested Now, Given Later Scheduled Cleanup?

Another question in response to our results (that show health risks to current toxic-site renters) is why CBRE/TCC should test and clean up the former NOTSPA, to protect current renters, when it says it will do so after it purchases the site? While this question appears reasonable, there are several difficulties. *First*, one problem is that site renters deserve to know what risks they face, to know whether they are being protected, as well as what protections government guidance and regulations require, and to be protected by government at those required levels. Yet, as previous paragraphs show, site renters have been deprived of both information and protection. Although the responsible parties (including CBRE/TCC, Space Bank, and the state regulator) have known about high site risks since CBRE/TCC’s 2007 site testing, no one has conducted the required indoor-air, groundwater, or additional soil-gas testing. 

*Second*, in addition, site renters especially deserve this information and protection for another reason. This that, although both the regulators and the responsible parties have said that the NOTSPA is now safe, our sampling results suggests the opposite. In particular, current site renters may not be safe.

*Third*, site renters deserve risk information and protection now because otherwise, they may never get it. CBRE/TCC has not yet purchased the site; it has been in escrow to buy the site for decades, and many prior contracts to purchase the site have failed, once prospective buyers discovered NOTSPA toxicity. Hence, the CBRE/TCC purchase may never be accomplished. If there is no purchase, then at a minimum, the site should made safe for current renters and future people. One cannot merely assume that protection will come in the future, including because, as already noted, CBRE/TCC will leave “in place” most site VOCs, at levels above those at which the state requires remediation.

*Fourth*, site renters especially deserve immediate protection because they appear not to have been protected in the past. Instead, regulators and the responsible parties failed to act in response to the already-mentioned 2004 Imminent and Substantial Endangerment Order. Because our 2021 sampling appears at least partly to confirm the very risks that the 2004 Order was meant to investigate and mitigate, arguably the regulators and the responsible parties may have failed because since 2007, they have neither given adequate risk information to site renters nor tested site indoor air. As result, they likely failed to provide adequate protection to NOTSPA renters.

Finally, California government vapor-intrusion standards clearly say that “points of departure [include]…10^−6^ for cancer risk…. For any exceedance of the points of departure for risk or hazard, based on soil-gas data, proceed to Step 3 for an indoor air investigation at current buildings” [26] (p. 17). However, at the former NOTSPA, the 2007 studies conducted by Space Bank and CBRE/TCC showed a soil-gas cancer risk as high as 5 orders of magnitude above the preceding 10^−6^ level, a level that requires indoor-air testing; for instance, sample NMSV10-5 reveals PCE at 443,480 µg/m^3^, posing a risk of 7.4 (10^−1^), given that the screening level, 0.46 µg/m^3^, poses a risk of 10^−6^ [17]. Clearly concurrent, same-location, full-site indoor air and soil gas tests should have been conducted in 2007, but they were not. Therefore, justice for site renters already has been delayed, thus denied. Justice ought not again be delayed and denied.

### 4.4. Fourth Question: Future Research Directions?

Our results suggest that a trend-setting, major commercial redeveloper of toxic sites, CBRE/TCC, appears to have spread misinformation about both the current safety and the future risks associated with the dangerous, inadequately tested, former NOTSPA—most of whose contaminants CBRE/TCC will leave onsite. Yet as already mentioned, CBRE/TCC will redevelop the site for 550 small residential apartments, disproportionate numbers of which are reserved for affordable, low-income, and moderate-income families. Given these facts, future research might attempt to replicate our results. That is, scientists might attempt to discover the prevalence of flawed or incomplete toxic-site testing and cleanup, as well as the prevalence of assessors’/remediators’ spreading apparent misinformation about hazardous-facility risks, testing, cleanup, and the degree to which some toxic-site redevelopments put people at risk. 

If our results can be replicated, then as the recent Hunter’s Point, California, residential redevelopment of another prominent former US military site suggests [53,54,55], additional research needs to be done at other toxic sites where semi-privatized testing and remediation supposedly were conducted. This research could determine what government programs appear to, or fail to, provide the greatest independent checks on the adequacy of privatized toxic-site assessment/remediation. What semi-privatized, toxic-site programs achieve the best and worst results? What toxic-site programs and practices provide reliable or unreliable testing/cleanup? What toxic-site programs provide reliable or unreliable health and safety information about testing/cleanup?

### 4.5. Fifth Question: Future Policy Directions?

If our results can be replicated, they also suggest that government leaders may need to re-assess the reliability and performance of toxic-site regulators, regulatory-oversight programs, and semi-privatized testing/assessment. After all, in 2019 California banned private prisons, saying they are “driven to maximize shareholder profits” and “lack proper oversight” [65]. However, these same comments about prisons might also apply to semi-privatized toxic-site testing and cleanup. In both situations, financial conflicts of interest might be compromising safety, public health, oversight, and even rights to information itself.

## 5. Conclusions

In the United States and several other nations of the world, most toxic-site testing/remediation/redevelopment is conducted by private parties, usually redevelopers seeking commercial profits. However, because few studies have analyzed the effectiveness of such semi-privatized hazardous-site assessment and remediation programs, this analysis conducted a preliminary assessment of an important hypothesis. The *hypothesis* is that, contrary to the safety claims of the world’s largest commercial developer, and a major redeveloper of hazardous-waste facilities (CBRE/TCC), the authors’ screening assessment, especially its lab-certified, toxic-site, indoor-air samples show violations of all three prominent government, cancer-safety benchmarks—at every NOTSPA location tested. The results of this hypothesis are important because these findings suggest that toxic facilities may put site renters at risk, require additional testing/remediation, and reveal both scientific, public-health, and environmental-justice problems with privatized hazardous cleanup.

To begin to test this hypothesis, the study uses mainly GC/MS and TO-17 *methods* to sample, then analyze two weeks of continuous, indoor-air testing with passive sorbent tubes; these *results* provide a preliminary assessment (see the authors’ Section 2.2.1) of vapor intrusion and levels of volatile organic compounds in occupied rental units on the former US Naval Ordnance Testing Station, Pasadena, California. Indoor-air sampling results also reveal violations of all three government-safety benchmarks that we considered, namely, the California health-protective, Environmental Screening Levels (ESLs), the California No Significant Risk Levels (NSRLs), and the US EPA inhalation risks based on the inhalation unit risk (IUR).

Both the ESL, NSRL, and the IUR-based safety benchmarks and results reveal indoor-air carcinogens at nearly, and above, two orders of magnitude less protective than the standard one-in-a-million acceptable cancer risk. This suggests that site renters could face cancer risks more than 100 times higher than normal. Specifically, Table 6 shows that some indoor-air carbon tetrachloride and dichlorodifluoromethane concentrations, and all per/tetrachloroethylene levels—at all locations sampled—violate the ESLs. Table 7 shows that some indoor-air, carbon tetrachloride concentrations, and all perchloroethylene levels—at all locations sampled—violate NSRLs. Table 8 shows that some indoor-air, carbon tetrachloride concentrations, and all perchloroethylene levels—at all locations sampled—violate the IUR-based safety benchmark

On one hand, the prominent site assessor/remediator/redeveloper has repeatedly used traditional and social media to claim that the former NOTSPA toxic site is “safe at this time.” On the other hand, despite such safety claims, CBRE/TCC’s own studies show NOTSPA soil-gas-VOC levels pose a lifetime risk as high as 7.4 (10^−1^). Moreover, although state regulatory-guidance documents require indoor-air testing whenever soil-gas-VOC levels indicate a lifetime risk higher than 10^−6^, neither CBRE/TCC nor the regulator ever tested site indoor air. As a result of the preceding facts, the misleading safety claims of CBRE/TCC, and our indoor-air sampling results, this study draws the *preliminary conclusion* (see the authors’ Section 2.2.1) that our *hypothesis* is likely confirmed. That is, contrary to the safety claims of one of the world’s largest redevelopers of hazardous-waste facilities (CBRE/TCC), the authors’ lab-certified, toxic-site, indoor-air samples show violations of all three prominent government, cancer-safety benchmarks.

If these CBRE/TCC results can be replicated at other brownfields-redevelopment sites, they suggest that government may need to reconsider the public-health consequences of semi-privatized toxic-site testing/cleanup and the adequacy of government oversight of such facilities. Scientists and policymakers also may need to assess whether the financial incentives for reduced testing/cleanup, reduced public safety, and increased private profits may jeopardize the economic benefits of semi-privatized toxic-site assessment/remediation. 

The warning of social reformer and author Upton Sinclair seems appropriate to the CBRE/TCC situation. He said “It is difficult to get a man to understand something [such as the health threats caused by health misinformation about poor toxic-site testing/cleanup], when his salary depends on his not understanding it.” [66].

## Figures and Tables

**Table 1:** Onsite Soil-Gas Perchloroethylene (PCE): Up to 5 Orders of Magnitude above the Health-Protective, Environmental Screening Levels (ESLs) [15,17].

Sample Location Identifier	PCE (µg/m^3^) Concentration	/0.46 µg/m^3^ (Screening Level) = Times above Screening Level	Is the Regulator Requiring the Re- developer to Remove This Contamination? ^1^
NMSV10-5	342,000	743,480	yes
V9-15	137,000	298,000	no
VD2-30	122,000	265,217	no
V-5-15	79,000	172,000	no
V9-10	39,100	85,000	no
V10-5	36,300	79,000	no
NMSD3-60	22,300	48,480	no
V6-15	20,500	45,000	no
VD1-20	20,400	44,347	no
NASD3-113	17,900	38,913	no
V2-15	16,700	36,304	no
NMSV12-15	14,500	31,522	no
NMSV15-15	14,200	30,870	no
NMSV11-15	13,500	29,348	no
V18-15	13,500	29,348	yes
NMSV14-15	11,600	25,217	no
VD1-30	10,800	23,500	no
V8-15	10,500	23,000	no
NMSV2-15	10,200	22,174	no
V2-5	9470	21,090	no
V18-5	8320	18,090	no
NMSV13-5	5510	11,978	no
NMSV4-15	1290	2804	no

^1^ Only two of these PCE locations will be removed, as they are in metals hotspots/drains, the only areas that the regulator requires the redeveloper to remove [15] (Appendix A, and Figure 7).

**Table 2:** Soil-Gas Carbon Tetrachloride (CT): Up to 5 Orders of Magnitude above the Health-Protective, Environmental Screening Levels (ESLs) [15,17].

Sample Location Identifier	CT (µg/m^3^) Concentration	/0.067 µg/m^3^ (Screening Level) = Times above Screening Level	Is the Regulator Requiring the Re- developer to Remove This Contamination? ^1^
NMSD3-113	28,400	424,000	no
NMSD3-84	24,300	363,000	no
NMSD3-150	20,600	307,463	no
NMSD3-150	18,500	276,119	no
NMSD2-150	13,200	197,015	no
NMSD2-130	12,900	193,000	no
NMSD2-150	9830	146,700	no
NMSD3-60	8390	125,224	no
NMSO1-85	7530	112,388	no
NMSD1-99	5950	90,806	no
NMSD2-63	2670	40,000	no
VD1-30	2270	34,000	no
NMSD2-130	2270	33,881	no
NMSV7-5	1820	27,164	no
VD3-20	1450	21,642	no
VD3-30	1420	21,200	no
V2-5	1390	21,000	no
NMSV6-5	1380	20,600	no
V8-15	1360	20,300	yes
VO12-15	1190	18,000	no

^1^ Only two of these PCE locations will be removed, as they are in metals hotspots/drains, the only areas that the regulator requires the redeveloper to remove [15] (Appendix A, and Figure 7).

**Table 3:** Safety Benchmarks and Detection Limits, Former Naval Ordnance Testing Station, Pasadena (NOTSPA).

VOCs Sampled	Government-Mandated, Airborne-VOC Residential Screening Levels (ESLs), µg/m^3^	No Significant Risk Levels (NSRLs)/ µg/m^3^ per Day [43]	Inhalation Unit Risks (IURs), µg/m^3^ [43]	Beacon Environmental Services, Airborne-VOC Detection Limits,^1^ µg/m^3^	CBRC/Trammell Crow, Soil-Gas VOC Detection Limits, 2019 Site Studies, µg/m^3^,
Carbon Tetrachloride	0.47 ^2^	5	4.2 × 10^−5^	0.58	20.0 ^3^
Dibromomethane	4.2 ^4^	nd	nd	0.62	20.0 ^5^
Dichlorodifluoromethane/ Freon 12	0.12 ^6^	nd	nd	0.43	20.0 ^5^
Per/Tetrachloroethylene	0.46 ^2^	14	6.1 × 10^−6^	0.60	20.0 ^3^
Trichloroethylene	0.48 ^7^	14	2.0 × 10^−6^	0.75	20.0 ^3^
Trichloromethane/ Chloroform	0.12 ^7^	20	5.3 × 10^−6^	0.71	20.0 ^3^

^1^ Authors’ Chapter 4 in the online Supplemental Materials. ^2^ State of California screening level [42]. ^3^ [17]. ^4^ Neither US EPA nor California DTSC provides screening levels for dibromomethane/methylene bromide, but this ESL is from the state of Indiana; see Section 2.2.1.6 in this text. ^5^ [44]. ^6^ Partly because DCDFM has been phased out, it has no screening levels. However, its toxicity is comparable to TCM, according to the US National Academies of Science [36]. ^7^ US EPA screening level [40]. No data exist = nd.

**Table 4:** 12 Indoor-Air, Lower or Uncalibrated Passive-Sorbent-Sampler Results: 6 Toxic Chemicals at 11 Locations, Former Naval Ordnance Testing Station, Pasadena, California, NOTSPA (Authors’ Chapter 4 in the online Supplemental Materials). Bold = Violations of the ESLs.

SampleLocation	Residential ESL ^1^	A	B	C	D	E	F	G	H	I	J	J-Dup- licate	K
Units		µg/m^3^	µg/m^3^	µg/m^3^	µg/m^3^	µg/m^3^	µg/m^3^	µg/m^3^	µg/m^3^	µg/m^3^	µg/m^3^	µg/m^3^	µg/m^3^
Carbon Tetrachloride	0.47	<0.576	**0.679 ^2^**	<0.576	<0.576	<0.577	<0.577	<0.577	<0.577	<0.577	<0.577	<0.577	<0.577
Trichloromethane	0.12	<0.708	<0.708	<0.708	<0.708	<0.709	<0.709	<0.709	<0.709	<0.709	<0.709	<0.709	<0.709
Dibromomethane	4.3	<0.619	<0.619	<0.619	<0.619	<0.621	<0.621	<0.620	<0.620	<0.620	<0.620	<0.620	<0.620
Dichlorodifluoro-methane ^3^	0.12	<0.427	**0.484 ^4^**	<0.427	<0.427	<0.428	1.83	<0.428	<0.428	**1.79**	<0.428	<0.428	**0.464 ^4^**
**Per/Tetrachloro-** **ethylene**	**0.46**	**7.97**	**13.4**	**7.02**	**12.8**	**1.74**	**4.44**	**2.61**	**1.43**	**2.92**	**1.5**	**1.63**	**1.71**
Trichloroethylene	0.48	<0.751	<0.751	<0.751	<0.751	<0.752	<0.752	<0.752	<0.752	<0.752	<0.752	<0.752	<0.752

^1^ [17,26,27,40,42]. ^2^ J Value reported is above the CT residential screening level, 0.47 µg/m^3^ (see authors’ Table 3); above the Beacon limit of detection, 0.577 µg/m^3^; but below the Beacon limit of quantitation; further testing could address this problem. ^3^ See Section 2.2.1.6, this paper. ^4^ J Value reported is above the DCDFM residential screening level, 0.12 µg/m^3^ (see authors’ Table 3); above the Beacon limit of detection, 0.428 µg/m^3^; but below the Beacon limit of quantitation; further testing could address this problem.

**Table 5:** PCE Indoor-Air, Passive-Sorbent-Sampler Interpretation of Results, Calibrated Generally per Beacon Canister-Comparison Study, ^1^ Former Naval Ordnance Testing Station, Pasadena, California (NOTSPA). Bold = Violations.

Sample Location	Residential, Commercial ESL, µg/m^3^ ^1^	Detected-Contaminant Levels, µg/m^3^											
		A	B	C	D	E	F	G	H	I	J	J-Dup-Licate	K
Per/Tetrachloroethylene, Beacon Sorbent Tubes	0.46, 2.0	**7.97**	**13.4**	**7.02**	**12.8**	**1.74**	**4.44**	**2.61**	**1.43**	**2.92**	**1.5**	**1.63**	**1.71**
Per/Tetrachloroethylene, Applying Beacon General Calibration (row 1) (1.55–2.31) ^2^	0.46, 2.0	**12.35–18.41**	**20.77–30.95**	**10.88–16.22**	**19.84–29.59**	**2.67–4.02**	**6.88–10.26**	**4.05–6.03**	**2.22–3.30**	**4.53–6.75**	**2.33–3.47**	**2.53–3.77**	**2.65–3.95**

^1^ [17,26,27,40,42]. See Section 2.2.1.3, this paper. ^2^ See [33] Section 3.4 and Section 3.4.1, this paper.

**Table 6:** Violations of the First Safety Benchmark, No “Response-Action” Levels or Environmental Screening Level (ESLs), Indoor Air, Former Naval Ordnance Testing Station, Pasadena, California (NOTSPA). Bold = Violations.

Contaminant	“Safe,” No- Response- Action Level ^1^ = ESL ^2^ µg/m^3^	Detected-Contaminant Levels, µg/m^3^				Risks of Detected-Contaminant Levels, Based on the 10^−6^ Risk ESLs				
		Generally Calibrated ^3^ (Uncalibrated Results) X (1.55–2.31)		Uncalibrated ^4^		ESL ^2^	Generally Calibrated ^3^		Uncalibrated ^4^	
		Highest Results	Lowest Results	Highest Results	Lowest Results		Highest Risks^5^	Lowest Risks	Highest Risks	Lowest Risks
Carbon tetrachloride	0.47 ^6^	1.1–1.6	1.1–1.6	0.679 J ^7^	0.679 J ^7^	10^−6^	**(3.4) 10^−6^**	**(2.3) 10^−6^**	**(1.4) 10^−6^**	**(1.4) 10^−6^**
Dichlorodifluoromethane	0.12 ^8^	2.8–4.2	0.7–1.1	1.83	0.464 J ^7^	10^−6^	**(3.5) 10^−5^**	**(5.8) 10^−6^**	**(1.5)10^−5^**	**(3.9) 10^−6^**
Perchloroethylene	0.46 ^6^	20.8–31.0	2.2–3.3	13.4	1.43	10^−6^	**(6.7) 10^−5^**	**(4.8) 10^−6^**	**(2.9) 10^−5^**	**(3.1) 10^−6^**

^1^ [17,26,27,40,42]. ^2^ See Section 2.2.1.3, this paper. ^3^ See Section 2.2.1.5, this paper. ^4^ See Section 3.4 and Section 3.4.1, this paper. ^5^ Given the perchloroethylene (PCE) ESL = 0.46 ug/m^3^ (column 2), and the PCE ESL risk of 10^−6^, one extrapolates to obtain the risk of various detected PCE levels. ^6^ State of California screening level [42]. ^7^ J Value is estimated because it is above the Beacon detection limit, thus clearly detected, but below the Beacon quantitation limit; as a result, it is somewhere between the detection and quantitation limits, but clearly above the residential ESL. ^8^ See Section 2.2.1.6, this paper.

**Table 7:** Violations of the Second Safety Benchmark, No Significant Risk Level (NSRL), Indoor-Air Passive Samplers, Former Naval Ordnance Testing Station, Pasadena, California (NOTSPA). Bold = Violations.

Contaminant	“Safe,” No- Response- Action Level ^1^ = ESL ^2^ 10^−6^ Risk, µg/m^3^	Detected-Contaminant Levels, µg/m^3^				Risks of Detected-Contaminant Levels, Based On The NSRL Or “Safe Harbor” Level, µg/m^3^ Per Day				
		Generally Calibrated ^3^		Uncalibrated ^4^		NSRL ^5^	Generally Calibrated ^3^		Uncalibrated ^4^	
		Highest Results	Lowest Results	Highest Results	Lowest Results		Highest Risks ^6^	Lowest Risks	Highest Risks	Lowest Risks
Carbon tetrachloride	0.47 ^7^	1.1–1.6	1.1–1.6	0.679 J ^8^	0.679 J ^8^	5	**(6.4) 10^−5^**	**(4.4) 10^−5^**	**(2.7) 10^−5^**	**(2.7) 10^−5^**
Dichlorodifluoromethane	0.12 ^9^	2.8–4.2	0.7–1.1	1.83	0.464 J ^8^	not given	-	-	-	-
Perchloroethylene	0.46 ^7^	20.8–31.0	2.2–3.3	13.4	1.43	14	**(4.4) 10^−4^**	**(3.1) 10^−5^**	**(1.9) 10^−4^**	** (2.0) 10^−5^**

^1^ [17,26,27,40,42]. ^2^ See Section 2.2.1.3, this paper and [28]. ^3^ See Section 2.2.1.5, this paper. ^4^ See Section 3.4 and Section 3.4.1, this paper. ^5^ The NSRL of California DTSC is defined in regulations as the daily contaminant-intake level calculated to result in one excess case of cancer in a population of 100,000 exposed people [43]. Per note 2 above, we use the residential NSRL [28]. ^6^ Given a detected contaminant level/day, associated with the NSRL, this is the generally calibrated risk range represented by (detected level) (20 h) for residential risk and (detected level) (10 h) for commercial risk. ^7^ State of California screening level [42]. ^8^ J Value is estimated because it is above the Beacon detection limit, but below the Beacon quantitation limit; as a result, it is somewhere between the detection and quantitation limits, but clearly detected above the residential ESL. ^9^ See Section 2.2.1.6, this paper.

**Table 8:** Violations of the Third Safety Benchmark, Based on Inhalation Unit Risk (IUR), Indoor-Air Passive Samplers, Former Naval Ordnance Testing Station, Pasadena, California (NOTSPA). Bold = Violations.

Contaminant	“Safe,” No-Response-Action Level ^1^ = ESL ^2^ 10^−6^ Risk, µg/m^3^	Detected-Contaminant Levels, µg/m^3^				Risks of Contaminant Levels, Based on the IUR µg/m^3^				
		Generally Calibrated ^3^		Uncalibrated ^4^		IUR ^5^	Generally Calibrated ^3^		Uncalibrated ^4^	
		Highest Results	Lowest Results	Highest Results	Lowest Results		Highest Risks ^6^	Lowest Risks	Highest Risks	Lowest Risks
Carbon tetrachloride	0.47 ^7^	1.1–1.6	1.1–1.6	0.679 J ^8^	0.679 J ^8^	(4.2) 10^−5^	**(6.7) 10^−5^**	**(4.6) 10^−5^**	**(2.9) 10^−5^**	**(2.9) 10^−5^**
Dichlorodifluoromethane	0.12 ^9^	2.8–4.2	0.7–1.1	1.83	0.464 J ^8^	not given	-	-	-	-
Perchloroethylene	0.46 ^7^	20.8–31.0	2.2–3.3	13.4	1.43	(6.1) 10^−6^	**(1.9) 10^−4^**	**(1.3) 10^−5^**	**(8.2) 10^−5^**	**(8.7) 10^−6^**

^1^ [17,26,27,40,42]. ^2^ See Section 2.2.1.3, this paper. ^3^ See Section 2.2.1.5, this paper. ^4^ See Section 3.4 and Section 3.4.1, this paper. ^5^ The Inhalation Unit Risk (IUR) of US EPA “is an estimate of the increased cancer risk from inhalation exposure to a [contaminant] concentration of 1 µg/m^3^ for a lifetime” [49]. ^6^ Given the perchloroethylene (PCE) IUR = 6.1 × 10^−6^ (column 7), the PCE inhalation risk for the value of the highest generally calibrated PCE detection of 31 µg/m^3^ (column 3) = (PCE IUR) (PCE detected value whose risk we want to know) = (0.0000061) (31) = 0.00019 or 1.9 × 10^−4^ inhalation cancer risk. ^7^ State of California screening level [42]. ^8^ J Value is estimated because it is above the Beacon detection limit, but below the Beacon quantitation limit; as a result, it is somewhere between the detection and quantitation limits, but above the residential ESL. ^9^ See Section 2.2.1.6 of this analysis.

## Data Availability

Except for the authors’ sorbent-tube results, available as Chapters 1 and 4, data presented in this study are openly available in the online repository, Envirostor, of the California Department of Toxic Substances Control, at <envirostor.dtsc.ca.gov/public/profile_report?global_id=19970020> (accessed on 28 March 2021). Restrictions apply to the availability of some information about the authors’ sorbent-tube-sampling data. Due to legal and ethical constraints, some data (on the rental-unit numbers of the areas sampled, the precise rental-unit locations of the samples, and the names of the toxic-site tenants whose rental units were sampled) may obtained only from the toxic-site renters and are available from kshrader@nd.edu, upon the written permission of these toxic-site renters.

## Supplementary Materials

The following are available online at https://www.mdpi.com/article/10.3390/ijerph18083882/s1. Supplementary Materials: the authors’ Chapters 1, 2, 3, and 4. Chapter 1: Chapter 1 contains the protocols and instructions from the laboratories at Beacon Environmental Services, Inc., located in the state of Maryland, who provided the samplers and analyzed the sorbent-tube results for this study. Beacon is the top US passive-sorbent-tube sample provider and the top US analytical laboratory assessor of sorbent-tube results. Chapter 2: Chapter 2 contains information about Beacon Environmental Services, Inc., the top US passive-sorbent-tube sample provider and the top US analytical laboratory assessor of sorbent-tube results; it is located in the state of Maryland, and it currently provides the samplers and analysis for the largest US passive-sampler project, run by the US EPA. Chapter 3: Chapter 3 contains additional information about data availability, owing to legal and ethical constraints. Chapter 4: Chapter 4 contains the full Beacon Analytical Report. It does not contain instrumentation raw data, e.g., preliminary gas chromatograph data, for reasons outlined in Chapter 3.

## Abbreviations

Acronyms Used in the Article

Acronym	Phrase That the ACRONYM Represents
ATSDR	US Agency for Toxic Substances and Disease Registry
CERCLA	Comprehensive Environmental Response, Compensation, and Liability Act
CT	Carbon Tetrachloride
DCDFM	Dichlorodifluoromethane
DBM	Dibromomethane
DOD ELAP	US Department of Defense Environmental Laboratory Accreditation Program
DTSC	California Department of Toxic Substances Control
EASI	Environmental Asset Services, Inc.
EPA	US Environmental Protection Agency
ESL	Environmental Screening Level
HVAC	Heating, Ventilation, and Air Conditioning
NOTSPA	US Naval Ordnance Testing Station, Pasadena, California
NSRL	No Significant Risk Levels
OEHHA	California Office of Environmental Health Hazard Assessment
PCE	Perchloroethylene (Tetrachloroethylene)
TCC	Trammell Crow Company
TCE	Trichloroethylene
TCFM	Trichlorofluoromethane
TCM	Chloroform/Trichloromethane
TD-GC/MS	Thermal-Desorption Gas Chromatography/Mass Spectrometry
US	United States
VI	Vapor Intrusion
VOC	Volatile Organic Compound
