# Correction: Shrader-Frechette, K.; Biondo, A.M. Health Misinformation about Toxic-Site Harm: The Case for Independent-Party Testing to Confirm Safety. *Int. J. Environ. Res. Public Health* 2021, *18*, 3882

**DOI:** 10.3390/ijerph18189796

**Published:** 2021-09-17

**Authors:** Kristin Shrader-Frechette, Andrew M. Biondo

**Affiliations:** 1 Department of Biological Sciences, University of Notre Dame, 100 Malloy Hall, Notre Dame, IN 46556, USA; 2 Department of Economics, University of Notre Dame, 3060 Jenkins Nanovic Hall, Notre Dame, IN 46556, USA; abiondo@nd.edu

Because part of the text was unintentionally omitted, the first paragraph under Section 2.2.4.3. on p. 13 was jumbled and incomplete when it was published [1]:

“The third, or IUR-based, benchmark is the set of US EPA and California EPA continuous inhalation-exposure levels that one calculates by multiplying the US EPA [45] or California EPA [43], contaminant-specific IUR (the upper-bound excess lifetime cancer risk estimated to result from continuous exposure to an agent at a concentration of 1 µg/m^3^ in air), as detected by the site-contaminant sample.”

The preceding published sentence should be corrected to the following sentence:

“The third, or IUR-based benchmark is the excess-lifetime cancer risk from continuous exposure to an airborne contaminant, calculated by multiplying the contaminant’s IUR by one’s lifetime airborne exposure to that contaminant (expressed in µg/m^3^), as detected by site-contaminant testing; the IUR is the excess-lifetime cancer risk from continuous exposure to an airborne contaminant at a concentration of 1 µg/m^3^ [43,45].”

In addition, the authors discovered that their statement on p. 30 might cause misperceptions:

“The authors declare no conflict of interest.”

The preceding published sentence should be corrected to the following sentences to avoid any misperceptions of conflicts of interest:

“The authors declare they have no financial conflicts of interest. In fact, the authors believe they have no actual conflicts of interest of any kind. However, to avoid the perception of conflicts of interest, the authors share the following information. For decades Shrader-Frechette has directed and been a member of a University of Notre Dame pro-bono faculty/student group, the Center for Environmental Justice and Children’s Health. It responds to worldwide requests for scientific assistance from poor people, minorities, and children who are threatened by environmental injustice. No members of the Notre Dame group have ever received payment for these requested services. In Spring 2018, Pasadena, California residents living near the former Naval Ordnance Test Station Pasadena (NOTSPA) toxic site requested pro-bono Notre Dame scientific help. As a result, during August–December 2018, the Notre Dame group assessed developer Trammell Crow’s NOTSPA studies. Because they discovered major site-safety violations, serious risk underestimates, testing and cleanup errors, violations of data-quality standards and data-usability evaluation, and failure to pass a scientific-data audit, the Notre Dame scientists concluded in December 2018 that publishing their findings was necessary to protect public health and to ensure safe, complete testing/cleanup. Thus, in December 2018, the Notre Dame group began writing up/documenting their extensive results for four different scientific publications. They submitted the first publication on 15 October 2019. However, as the Notre Dame publications reveal, in November 2019, the much-criticized state regulator, California Department of Toxic Substances Control (DTSC), issued a final refusal to correct the toxic-site scientific/safety/testing/legal violations, despite the formal, written, May 2019 requests made by hundreds of citizens, community groups, scientists, including Dr. Shrader-Frechette, and the nonprofit charity, Stop Toxic Housing in Pasadena. As a result of the DTSC refusal, this nonprofit, all-volunteer charity (of which Dr. Shrader-Frechette was elected director) sued DTSC alone. Developer Trammell Crow was not sued but is an indirect “interested party” in this lawsuit because, as the Notre Dame publications also reveal, DTSC’s regulatory failures allow inexpensive, incomplete, illegal testing and cleanup that benefit Trammell Crow financially. This lawsuit against DTSC thus was *the result* of DTSC’s failure to correct serious site testing/cleanup/safety failures, documented by more than a year of Notre Dame scientific studies. Without the uncorrected violations discovered by Notre Dame scientists, there would be no lawsuit. Because state-required, indoor-air testing had not been done, and toxic-site renters were not protected, despite site carcinogens up to nearly a million times above allowed levels, beginning in 2020, the Notre Dame group conducted site-indoor-air testing to provide empirical support for their 2018 research conclusions and three earlier publications. In summary, the authors declare that although Dr. Shrader-Frechette’s and Notre Dame’s pro-bono scientific assistance to environmental-injustice victims is a potential, non-financial conflict of interest, this pro-bono work is part of Shrader-Frechette’s/university scientists’ typical job description: to perform (1) *research*, (2) teaching, and (3) pro-bono professional *service* that helps to protect the public good.”

The authors would like to apologize for any inconvenience caused to the readers by these changes.
