# Supplementary material for: Health Misinformation about Toxic-Site Harm: The Case for Independent-Party Testing to Confirm Safety
Source: Int J Environ Res Public Health. 2021 Apr 7;18(8):3882. doi: 10.3390/ijerph18083882 (PMC8067841; doi:10.3390/ijerph18083882)
Supplement: Supplementary file 1 [file ijerph-18-03882-s001.pdf]

Contents of Supplemental File

Page

Chapter 1 2-7

Chapter 2 8-9

Chapter 3 10-11

Chapter 4 12-140

Chapter 1

BEACON ENVIRONMENTAL

DoD ELAP & ISO/IEC 17025 Accredited Laboratory
TNI NEFAP Accredited Field Sampling Organization
TNI NELAP State Accredited Laboratory

FIELD KIT GUIDE
FOR
AIR SAMPLING INVESTIGATIONS

[READ ENTIRE GUIDE BEFORE STARTING SURVEY]

I. General Information

A. BEACON assembled this kit for **University of Notre Dame** to perform sampling on the **Former DoD Facility Site in California.** To meet the project objectives, retrieve Samplers **approximately 14 days after installation**. Contact BEACON following completion of sampling at Ryan.Schneider@beacon-usa.com or 1-410-838-8780 to schedule analysis in BEACON's laboratory.

B. Inventory the contents of the package ***before going to the field***, and compare items with the contents page to verify all items are provided. Please conduct the inventory without opening the plastic bags because components are thoroughly cleaned prior to shipment.

C. Prior to returning the Samplers to BEACON, verify that the caps are completely secured on the Samplers, the Samplers are sealed individually in the small Sampler Bags and packed together in the larger Return Shipment Bag, containing an adsorbent pak.

D. Upon completion of the survey, fill in the Chain-of-Custody Form with the following information: (i) Field Sample IDs, (ii) the name and contact phone number of the person submitting the samples, and (iii) signature and date of person relinquishing samples. Return the Chain-of-Custody Form with the Field Kit to BEACON. Retain photocopies or photographs for your record.

Please use the provided return label on a Fed Ex Box, and relinquish the package to the nearest Fedex pickup location.

NOTE: **DO NOT USE STYRENE PEANUTS, NEWSPAPER, OR OTHER PACKING MATERIALS THAT MAY CONTAMINATE THE SAMPLES. AVOID SMOKING WHILE HANDLING SAMPLERS.**

II. Contents

A. This project contains the components needed for a **11**-point survey. **Do not open bags until deployment.**

Code/Item		Quantity
**(1)**	BEACON PASSIVE SAMPLERS	**11**
**(2)**	VIAL HOLDERS	**11**
**(3)**	SAMPLING CAPS (in container)	**11**
**(4)**	CAP STORAGE CONTAINERS	**1**
**(5)**	3” x 4” PLASTIC SAMPLER BAGS (for return shipment of samples)	**11**
**(6)**	12” x 12” PLASTIC RETURN SHIPMENT BAG	**2**
**(7)**	FEDEX RETURN LABEL	**1**

B. In addition to the materials provided, field teams will need:

- NITRILE GLOVES
- BALL-POINT PEN and CLIPBOARD
- WIRE OR HEAVY STRING
- FED EX BOX (for return shipping)

III. Instructions

A. GENERAL:

Deployment and retrieval of Samplers requires only one person.

B. SAMPLER DEPLOYMENT:

Duplicates: Duplicate analysis can be performed on any field sample by analyzing the second set of sorbents from the vial. To select field sample duplicates, note them on the CoC; **DO NOT** install a second sampler (co-located) to collect a field sample duplicate. Add a second entry to the CoC with the field sample ID followed by "D" or "Dup" (i.e., PSG-08-Dup is the duplicate for PSG-08). There is an additional per sample charge for analysis of any duplicates.

1. Cut a piece of wire or string long enough to hang the sampler at the desired height and place within easy reach. Remove one of the Samplers (a glass vial containing two sets of hydrophobic adsorbent cartridges) and replace the solid cap on the Sampler Vial with a Sampling Cap (a one-hole cap with a screen meshing insert). Place the solid cap in the Cap Storage Container. Slide the sampler into the vial holder until it "clicks" into place, with the sampling cap facing out from the holder. Secure the previously cut piece of wire or string to the loop on the back of the holder. If using string, you can double the string and thread the looped end through the back of the holder, then thread the other ends of the string through the string's loop, and pull tight to ensure the string will not untie.

Note: At each sampling location, verify that the (black) sampling cap is on the vial before installing the Sampler.

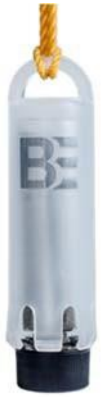

2. Secure the other end of the wire/string to any nearby structure (tubing, posts, railing, hooks, etc.). Suspend the sampler at the desired height with the sampling cap facing downward. Place the solid cap in the Cap Storage Container.

3. Record on the Chain-of-Custody: (a) sample location ID; (b) date/time of emplacement (to nearest minute); and (c) other relevant information.

4. Move to next location.

C. SAMPLER RETRIEVAL:

Duplicates: Duplicate analysis can be performed on any field sample by analyzing the second set of sorbents from the vial. To select field sample duplicates, note them on the CoC; **DO NOT** install a second sampler (co-located) to collect a field sample duplicate. Add a second entry to the CoC with the field sample ID followed by "D" or "Dup" (i.e., PSG-08-Dup is the duplicate for PSG-08). There is an additional per sample charge for analysis of any duplicates.

Indoor/Ambient Air Sampling:

1. At each sample location remove a solid cap from the Cap Storage Container and place it in easy reach

2. Remove the vial from the vial holder.

3. Remove the sampling cap and replace it with a solid cap. Use a ballpoint pen (not a Sharpie marker) to record the sample number, corresponding to the sample location, on the cap's label.

4. Place the sealed and labeled Sampler Vial in the 3" x 4" plastic Sampler Bag and record the sample number on the white block using a ballpoint pen. Then place the individually bagged and labeled sampler into the larger bag labeled "Return Shipment Bag."

Note: **Every sample must be individually bagged and placed in a Return Shipment Bag. If you know or suspect some sample(s) collected unusually high levels of contaminants, separately place these sample(s) in the extra bag provided.**

5. Record on the Chain-of-Custody: (a) date and time of retrieval (to nearest minute); and (b) any other relevant information.

6. After all samples have been retrieved, verify that the caps on each Sampler are sealed tightly and that the seals on the Sampler Bags are closed. Verify that all Samplers are stored in the Return Shipment Bag, which contains an adsorbent pak.

7. Pack samples in a Fed Ex Box, attach the provided return label, and relinquish the package to the nearest Fedex pickup location.

Note: **Please do not return the sampling caps or the wire as they could bias the samplers.**

Please, remember:

- ☐ Label, seal, and individually bag Passive Samplers in 3"x4" bags provided, with all the samples in the larger bag marked "Return Shipment" with a Trip Blank in each bag
- ☐ Include the signed and dated Chain-of-Custody Form
- ☐ Only use approved packaging materials (*i.e.*, no Styrofoam peanuts, etc.)
- ☐ Notify BEACON's laboratory that samples are being returned

---

THANK YOU!

Chapter 2

Chapter 2

Beacon is the top Passive Sorbent Sampling laboratory in the United States, as the text reveals. For instance, it is supplying both the passive samplers and the laboratory analyses for the largest passive-sample, VOC-monitoring project in the United States, run by the US EPA to assess vapor intrusion in thousands of offsite buildings contaminated by Hill Air Force base in Northern Utah. Beacon also was the first (National Environmental Field Activities Program) NEFAP-accredited field sampling and measurement organization in the United States; and its President was lead author on the (American Society for Testing and Materials) ASTM Standard D7758-2011, for Passive Soil Gas Sampling. The US EPA, US Department of Defense, US Department of Energy, state agencies, and commercial clients in every US state and 25 foreign countries, from across 7 continents, have used Beacon's passive soil-gas samplers and laboratory analyses of them <beacon-usa.com/>.

Chapter 3

Chapter 3

Two types of data used in this study are not yet publicly available. These data are (L) the 12 indoor-air sampling locations and (R) the raw data in terms of which Beacon Environmental Services, Inc, conducted its analyses.

Because of the Site Access Agreement (signed by renters at the former US Naval Ordnance Testing Station, Pasadena, California (NOTSPA), as condition of their allowing indoor-air sampling in their units), data L will not be available until these former-NOTSPA rental tenants give written permission for release of L. Current renters fear the toxic-site owners will evict them (they have month-to-month leases), on very short notice, if the owners are able to identify which tenants allowed indoor-air testing. These tenants are now in the process of securing alternative rental space, a difficult task because, compared to current-market-rental rates, those for the toxic-site units are heavily discounted, owing to their health risks. Site renters sought our passive-sorbent-tube testing mainly because they wanted to know what potential health risks they faced because of their renting units on a toxic site. Although these tenants now want to move, because of the serious risks, do not want to face eviction--before they have made alternative arrangements---merely because they exercised their legal rights to protect themselves.

Raw data L likewise will not be available until site renters have finished their analyses of it, needed to help reveal what potential health risks the tenants face because of the toxic site. Once site tenants have finished analyses of their private health risks, the Project Manager will both deposit the raw data in a publicly available database and provide the accession numbers for data L. Note that US EPA says in its OLRS (Open Literature Review Summaries) that typically raw data are not available for review, only the data in the published study [50]. Note also that top journals such as *Archives of Toxicology*, *PLOS*, and *Toxicological Sciences* encourage, but do not require, submission of raw data unless one presents genome-wide data [51]. Finally, note that, for at least 3 reasons, there should be no or little question about the reliability of the study results, based on the raw data collected in Beacon Passive Sorbent Samplers, supplied by Beacon Environmental Services, Inc, and analyzed by Beacon Environmental Services, Inc. These reasons are listed below:

- Beacon Environmental Services, Inc. is the *industry leader* in Passive Sorbent Sampling; see Authors' Appendix B and the text of the article, Methods and Materials.
- Beacon is a top laboratory, completely *independent of the authors*, and Beacon supplied the main materials and the only laboratory analyses for this study.
- Beacon certified that the returned samplers *passed all tests*, required by Beacon for quality-control, calibration, etc.

Chapter 4

Beacon Environmental
2203A Commerce Road, Suite 1
Forest Hill, MD 21050 USA
1.410.838.8780

CERTIFICATE OF ANALYSIS

Beacon Proposal No.: 201201H01

Laboratory Work Order: 0005542

Project Description:

So-Cal Military Toxic Site

Notspa, CA

Prepared for:

Kristin Shrader-Frechette

University of Notre Dame

Department of Biological Sciences

South Bend, IN 46556

Ryan W. Schneider

Senior Project Manager

January 25, 2021

All data meet requirements as specified in the Beacon Environmental Quality Assurance Project Plan and the results relate only to the samples reported. The work performed was in accordance with ISO/IEC 17025:2017 and DoD ELAP requirements. This report shall not be reproduced, except in full, without written approval of the laboratory. Release of the data contained in this data package has been authorized by the Laboratory Director or his signee, as verified by the following signatures:

Steven C. Thornley
Laboratory Director

Peter B. Kelly
Interim Quality Manager

Table of Contents

Cover Page	1
Sample Summary	5
Case Narrative	6
Analytical Results	7
Summary of Compound Detections	8
Data Summary Table-Concentration	10
Detailed Analytical Results	11
0005542-01 - A	12
0005542-02 - B	13
0005542-03 - C	14
0005542-04 - D	15
0005542-05 - E	16
0005542-06 - F	17
0005542-07 - G	18
0005542-08 - H	19
0005542-09 - I	20
0005542-10 - J	21
0005542-11 - J-DUP	22
0005542-12 - K	23
QC Summaries	24
QC/CLP Tables	38
CLP Form 1: Sequence Summary	39
CLP Table 2: SURR Summary	41
CLP Table 3A: LCS Summary	43
CLP Table 3B: LCSD Summary	45

Table of Contents (continued)

CLP Table 3C: Sample Duplicate Summary 47

CLP Table 4: Method Blank Summary 48

CLP Table 5: Tune Summary 51

CLP Table 6: Initial Calibration 60

CLP Table 7: RRF Continuing Calibration Summary 62

CLP Table 8: ISTD & RT Summary 70

Additional QC Information 73

Holding Time Report 74

Sample Analysis & Prep Summary 75

Sample Result Calculations 76

Equation 78

MDL/MRL Calculation Summary Table 79

Certifications 82

Notes and Definitions 83

Standard Traceability 84

Stock Standard Certificates of Analysis 85

Sample Preparation/Purge Log 124

Sample Raw Data 129

0005542-01 / A / K21010505.D 130

0005542-02 / B / K21010506.D 137

0005542-03 / C / K21010507.D 145

0005542-04 / D / K21010605.D 152

0005542-05 / E / K21010509.D 159

0005542-06 / F / K21010510.D 166

0005542-07 / G / K21010511.D 173

Table of Contents (continued)

0005542-08 / H / K21010606.D	180
0005542-09 / I / K21010513.D	187
0005542-10 / J / K21010514.D	194
0005542-11 / J-DUP / K21010515.D	201
0005542-12 / K / K21010516.D	208
Batch & Sequence Raw Data	215
TO-17 (Passive) / 21A0006-BS1	216
TO-17 (Passive) / 21A0006-BLK1	225
TO-17 (Passive) / B21A005-ICV1	231
TO-17 (Passive) / B21A005-CCV1	240
TO-17 (Passive) / 21A0007-BS1	249
TO-17 (Passive) / 21A0007-BLK1	258
TO-17 (Passive) / B21A006-ICV1	264
TO-17 (Passive) / B21A006-CCV1	273
Calibration Data/Chromatograms	282
Sample Management Records	326
Sample Condition & Receipt Log	327
Chain of Custody	329

BEACON ENVIRONMENTAL

2203A Commerce Road, Suite 1
Forest Hill, MD 21050 USA
1.410.838.8780

CERTIFICATE OF ANALYSIS

**University of Notre Dame**	**Site Name:** So-Cal Military Toxic Site	**Beacon Proposal:** 201201H01
Department of Biological Sciences	**Site Location:** Notspa, CA	**Lab Work Order:** 0005542
South Bend, IN 46556	**Project Manager:** Kristin Shrader-Frechette	**Reported:** 01/25/2021

Sample Summary

Lab Sample ID	Client Sample ID	Received	Analysis	Matrix
0005542-01	A	01/04/2021	TO-17 (Passive)	Indoor Air
Sampler Type:	Beacon Passive Sampler			
0005542-02	B	01/04/2021	TO-17 (Passive)	Indoor Air
Sampler Type:	Beacon Passive Sampler			
0005542-03	C	01/04/2021	TO-17 (Passive)	Indoor Air
Sampler Type:	Beacon Passive Sampler			
0005542-04	D	01/04/2021	TO-17 (Passive)	Indoor Air
Sampler Type:	Beacon Passive Sampler			
0005542-05	E	01/04/2021	TO-17 (Passive)	Indoor Air
Sampler Type:	Beacon Passive Sampler			
0005542-06	F	01/04/2021	TO-17 (Passive)	Indoor Air
Sampler Type:	Beacon Passive Sampler			
0005542-07	G	01/04/2021	TO-17 (Passive)	Indoor Air
Sampler Type:	Beacon Passive Sampler			
0005542-08	H	01/04/2021	TO-17 (Passive)	Indoor Air
Sampler Type:	Beacon Passive Sampler			
0005542-09	I	01/04/2021	TO-17 (Passive)	Indoor Air
Sampler Type:	Beacon Passive Sampler			
0005542-10	J	01/04/2021	TO-17 (Passive)	Indoor Air
Sampler Type:	Beacon Passive Sampler			
0005542-11	J-DUP	01/04/2021	TO-17 (Passive)	Indoor Air
Sampler Type:	Beacon Passive Sampler			
0005542-12	K	01/04/2021	TO-17 (Passive)	Indoor Air
Sampler Type:	Beacon Passive Sampler			

Project Completeness

Samples Received: **12**
Samples Analyzed: **12**

2203A Commerce Road, Suite 1
Forest Hill, MD 21050 USA
1.410.838.8780

BEACON ENVIRONMENTAL

CERTIFICATE OF ANALYSIS

**University of Notre Dame**	**Site Name:** So-Cal Military Toxic Site	**Beacon Proposal:** 201201H01
Department of Biological Sciences	**Site Location:** Notspa, CA	**Lab Work Order:** 0005542
South Bend, IN 46556	**Project Manager:** Kristin Shrader-Frechette	**Reported:** 01/25/2021

Case Narrative

Beacon Environmental provided thermally conditioned Beacon Samplers for sampling, with analyses following U.S. EPA Method TO-17, with analytical results reported in µg/m3. Beacon calculated concentration results using the exposure period, target analyte mass, and the following procedures detailed in ISO 16017-2, *Indoor, ambient and workplace air-Sampling and analysis of volatile organic compounds by sorbent tube/thermal desorption/capillary gas chromatography-Part 2: Diffusive sampling.*

Beacon reports results and reporting limits to three significant digits.

Reporting Limits (RLs) for EPA Method TO-17
The limit of quantitation (LOQ) is 10 nanograms (ng), the limit of detection (LOD) is 5 ng and the detection limit (DL) is 2.5 ng; however, when reporting concentration data in **Analytical Results** section, the values are provided in micrograms per meter cubed (µg/m^3).

Calibration Verification
All continuing calibration verification (CCV) values are within ±30% of the true values as defined by the initial calibration and met the requirements specified in BEACON's Quality Manual.

Internal Standards and Surrogates
Internal standards and surrogates are spiked on all blanks (ICB, BLK), field samples and laboratory control samples (ICV/CALV, BS, ICV and CCV). Acceptance criteria for internal standards are 60 to 140 percent and surrogate recoveries are 70 to 130 percent; all internal standards and surrogates are within the acceptance criteria unless noted in the **Case Narrative**.

Blank Contamination
No targeted compounds above the limit of detection (LOD) for each compound were observed in the Laboratory Method Blanks.

Laboratory Control Samples
Acceptance criteria for surrogate and analytes recoveries are 70 to 130 percent; all recoveries are within the acceptance criteria unless noted in the **Case Narrative** section.

Discussion
BEACON received twelve (12) air samples on 01/04/2021. Samples were received in proper condition and laboratory control parameters were met unless otherwise noted below. Analyses of these samples were completed 01/06/2021. The work performed was in accordance with ISO/IEC 17025:2017. Sample chromatograms are included in the Raw Data section.

End of Case Narrative

2203A Commerce Road, Suite 1
Forest Hill, MD 21050 USA
1.410.838.8780

CERTIFICATE OF ANALYSIS

**University of Notre Dame**		**Site Name:**	So-Cal Military Toxic Site	**Beacon Proposal:**	201201H01
Department of Biological Sciences		**Site Location:**	Notspa, CA	**Lab Work Order:**	0005542
South Bend, IN 46556		**Project Manager:**	Kristin Shrader-Frechette	**Reported:**	01/25/2021

Analytical Results

BEACON ENVIRONMENTAL
2203A Commerce Road, Suite 1
Forest Hill, MD 21050 USA
1.410.838.8780

CERTIFICATE OF ANALYSIS

**University of Notre Dame**	**Site Name:** So-Cal Military Toxic Site	**Beacon Proposal:** 201201H01
Department of Biological Sciences	**Site Location:** Notspa, CA	**Lab Work Order:** 0005542
South Bend, IN 46556	**Project Manager:** Kristin Shrader-Frechette	**Reported:** 01/25/2021

Summary of Compound Detections- Concentration

A (0005542-01)
TO-17 (Passive)

Analyte	CAS#	Result	Q	RT	LOQ	LOD	DL	Units	DF	Analyzed
**Tetrachloroethene**	127-18-4	**7.97**		8.30	1.21	0.604	0.604	µg/m³	1	01/05/2021 15:38

B (0005542-02)
TO-17 (Passive)

Analyte	CAS#	Result	Q	RT	LOQ	LOD	DL	Units	DF	Analyzed
**Dichlorodifluoromethane (Freon 12)**	75-71-8	**0.484**	J	1.53	0.854	0.427	0.427	µg/m³	1	01/05/2021 16:07
**Carbon Tetrachloride**	56-23-5	**0.679**	J	4.67	1.15	0.576	0.576	µg/m³	1	01/05/2021 16:07
**Tetrachloroethene**	127-18-4	**13.4**		8.30	1.21	0.604	0.604	µg/m³	1	01/05/2021 16:07

C (0005542-03)
TO-17 (Passive)

Analyte	CAS#	Result	Q	RT	LOQ	LOD	DL	Units	DF	Analyzed
**Tetrachloroethene**	127-18-4	**7.02**		8.30	1.21	0.604	0.604	µg/m³	1	01/05/2021 16:37

D (0005542-04)
TO-17 (Passive)

Analyte	CAS#	Result	Q	RT	LOQ	LOD	DL	Units	DF	Analyzed
**Tetrachloroethene**	127-18-4	**12.8**		8.31	1.21	0.604	0.604	µg/m³	1	01/06/2021 09:35

E (0005542-05)
TO-17 (Passive)

Analyte	CAS#	Result	Q	RT	LOQ	LOD	DL	Units	DF	Analyzed
**Tetrachloroethene**	127-18-4	**1.74**		8.31	1.21	0.605	0.605	µg/m³	1	01/05/2021 17:36

F (0005542-06)
TO-17 (Passive)

Analyte	CAS#	Result	Q	RT	LOQ	LOD	DL	Units	DF	Analyzed
**Dichlorodifluoromethane (Freon 12)**	75-71-8	**1.83**		1.53	0.856	0.428	0.428	µg/m³	1	01/05/2021 18:06
**Tetrachloroethene**	127-18-4	**4.44**		8.31	1.21	0.605	0.605	µg/m³	1	01/05/2021 18:06

G (0005542-07)
TO-17 (Passive)

Analyte	CAS#	Result	Q	RT	LOQ	LOD	DL	Units	DF	Analyzed
**Tetrachloroethene**	127-18-4	**2.61**		8.30	1.21	0.605	0.605	µg/m³	1	01/05/2021 18:36

BEACON ENVIRONMENTAL

2203A Commerce Road, Suite 1
Forest Hill, MD 21050 USA
1.410.838.8780

CERTIFICATE OF ANALYSIS

**University of Notre Dame**	**Site Name:** So-Cal Military Toxic Site	**Beacon Proposal:** 201201H01
Department of Biological Sciences	**Site Location:** Notspa, CA	**Lab Work Order:** 0005542
South Bend, IN 46556	**Project Manager:** Kristin Shrader-Frechette	**Reported:** 01/25/2021

Summary of Compound Detections- Concentration

H (0005542-08)
TO-17 (Passive)

Analyte	CAS#	Result	Q	RT	LOQ	LOD	DL	Units	DF	Analyzed
**Tetrachloroethene**	127-18-4	**1.43**		8.30	1.21	0.605	0.605	µg/m³	1	01/06/2021 10:05

I (0005542-09)
TO-17 (Passive)

Analyte	CAS#	Result	Q	RT	LOQ	LOD	DL	Units	DF	Analyzed
**Dichlorodifluoromethane (Freon 12)**	75-71-8	**1.79**		1.53	0.856	0.428	0.428	µg/m³	1	01/05/2021 19:37
**Tetrachloroethene**	127-18-4	**2.92**		8.31	1.21	0.605	0.605	µg/m³	1	01/05/2021 19:37

J (0005542-10)
TO-17 (Passive)

Analyte	CAS#	Result	Q	RT	LOQ	LOD	DL	Units	DF	Analyzed
**Tetrachloroethene**	127-18-4	**1.50**		8.31	1.21	0.605	0.605	µg/m³	1	01/05/2021 20:06

J-DUP / J (0005542-11)
TO-17 (Passive)

Analyte	CAS#	Result	Q	RT	LOQ	LOD	DL	Units	DF	Analyzed
**Tetrachloroethene**	127-18-4	**1.63**		8.31	1.21	0.605	0.605	µg/m³	1	01/05/2021 20:37

K (0005542-12)
TO-17 (Passive)

Analyte	CAS#	Result	Q	RT	LOQ	LOD	DL	Units	DF	Analyzed
**Dichlorodifluoromethane (Freon 12)**	75-71-8	**0.464**	J	1.54	0.855	0.428	0.428	µg/m³	1	01/05/2021 21:06
**Tetrachloroethene**	127-18-4	**1.71**		8.30	1.21	0.605	0.605	µg/m³	1	01/05/2021 21:06

BEACON ENVIRONMENTAL
2203A Commerce Road, Suite 1
Forest Hill, MD 21050 USA
1.410.838.8780

CERTIFICATE OF ANALYSIS

**University of Notre Dame**	**Site Name:** So-Cal Military Toxic Site	**Beacon Proposal:** 201201H01
Department of Biological Sciences	**Site Location:** Notspa, CA	**Lab Work Order:** 0005542
South Bend, IN 46556	**Project Manager:** Kristin Shrader-Frechette	**Reported:** 01/25/2021

Data Summary Table- Concentration

Compound	Frequency	LOD (µg/m³)	Max Value (µg/m³)
Dichlorodifluoromethane (Freon 12)	4	0.427	1.83
Carbon Tetrachloride	1	0.576	0.679
Tetrachloroethene	11	0.604	13.4

2203A Commerce Road, Suite 1
Forest Hill, MD 21050 USA
1.410.838.8780

CERTIFICATE OF ANALYSIS

**University of Notre Dame**	**Site Name:**	So-Cal Military Toxic Site	**Beacon Proposal:**	201201H01
Department of Biological Sciences	**Site Location:**	Notspa, CA	**Lab Work Order:**	0005542
South Bend, IN 46556	**Project Manager:**	Kristin Shrader-Frechette	**Reported:**	01/25/2021

Detailed Analytical Results

BEACON ENVIRONMENTAL

2203A Commerce Road, Suite 1
Forest Hill, MD 21050 USA
1.410.838.8780

CERTIFICATE OF ANALYSIS

**University of Notre Dame**	**Site Name:** So-Cal Military Toxic Site	**Beacon Proposal:** 201201H01
Department of Biological Sciences	**Site Location:** Notspa, CA	**Lab Work Order:** 0005542
South Bend, IN 46556	**Project Manager:** Kristin Shrader-Frechette	**Reported:** 01/25/2021

Lab ID: 0005542-01 **Sample ID:** A **Matrix:** Indoor Air

Method: TO-17 (Passive)

Analyte	CAS#	Result (µg/m³)	Q	LOQ (µg/m³)	LOD (µg/m³)	DL (µg/m³)	DF	RRT Eval	Analyzed	File ID
Dichlorodifluoromethane (Freon 12)	75-71-8	<0.427	U	0.854	0.427	0.427	1	0.00	01/05/2021 15:38	K21010505.D
Chloroform	67-66-3	<0.708	U	1.42	0.708	0.708	1	0.00	01/05/2021 15:38	K21010505.D
Carbon Tetrachloride	56-23-5	<0.576	U	1.15	0.576	0.576	1	0.00	01/05/2021 15:38	K21010505.D
Dibromomethane	74-95-3	<0.619	U	1.24	0.619	0.619	1	0.00	01/05/2021 15:38	K21010505.D
Trichloroethene	79-01-6	<0.751	U	1.50	0.751	0.751	1	0.00	01/05/2021 15:38	K21010505.D
**Tetrachloroethene**	127-18-4	**7.97**		1.21	0.604	0.604	1	0.00	01/05/2021 15:38	K21010505.D

Analyte	*CAS#*	*% Recovery*	*Recovery Limits*	*Q*	*RRT Eval*	*Analyzed*	*File ID*
Surrogate: 1,2-DCA-d4	17060-07-0	*103%*	*70-130*		*0.00*	*01/05/2021 15:38*	K21010505.D
Surrogate: Toluene-d8	2037-26-5	*98.3%*	*70-130*		*0.00*	*01/05/2021 15:38*	K21010505.D
Surrogate: Bromofluorobenzene	460-00-4	*92.3%*	*70-130*		*0.00*	*01/05/2021 15:38*	K21010505.D

BEACON ENVIRONMENTAL
2203A Commerce Road, Suite 1
Forest Hill, MD 21050 USA
1.410.838.8780

CERTIFICATE OF ANALYSIS

**University of Notre Dame**	**Site Name:** So-Cal Military Toxic Site	**Beacon Proposal:** 201201H01
Department of Biological Sciences	**Site Location:** Notspa, CA	**Lab Work Order:** 0005542
South Bend, IN 46556	**Project Manager:** Kristin Shrader-Frechette	**Reported:** 01/25/2021

Lab ID: 0005542-02 **Sample ID:** B **Matrix:** Indoor Air
Method: TO-17 (Passive)

Analyte	CAS#	Result (µg/m³)	Q	LOQ (µg/m³)	LOD (µg/m³)	DL (µg/m³)	DF	RRT Eval	Analyzed	File ID
**Dichlorodifluoromethane (Freon 12)**	75-71-8	**0.484**	J	0.854	0.427	0.427	1	0.00	01/05/2021 16:07	K21010506.D
Chloroform	67-66-3	<0.708	U	1.42	0.708	0.708	1	0.00	01/05/2021 16:07	K21010506.D
**Carbon Tetrachloride**	56-23-5	**0.679**	J	1.15	0.576	0.576	1	0.00	01/05/2021 16:07	K21010506.D
Dibromomethane	74-95-3	<0.619	U	1.24	0.619	0.619	1	0.00	01/05/2021 16:07	K21010506.D
Trichloroethene	79-01-6	<0.751	U	1.50	0.751	0.751	1	0.00	01/05/2021 16:07	K21010506.D
**Tetrachloroethene**	127-18-4	**13.4**		1.21	0.604	0.604	1	0.00	01/05/2021 16:07	K21010506.D

Analyte	*CAS#*	*% Recovery*	*Recovery Limits*	*Q*	*RRT Eval*	*Analyzed*	*File ID*
Surrogate: 1,2-DCA-d4	17060-07-0	*101%*	*70-130*		*0.00*	*01/05/2021 16:07*	K21010506.D
Surrogate: Toluene-d8	2037-26-5	*96.4%*	*70-130*		*0.00*	*01/05/2021 16:07*	K21010506.D
Surrogate: Bromofluorobenzene	460-00-4	*95.3%*	*70-130*		*0.00*	*01/05/2021 16:07*	K21010506.D

BEACON ENVIRONMENTAL

2203A Commerce Road, Suite 1
Forest Hill, MD 21050 USA
1.410.838.8780

CERTIFICATE OF ANALYSIS

**University of Notre Dame**	**Site Name:** So-Cal Military Toxic Site	**Beacon Proposal:** 201201H01
Department of Biological Sciences	**Site Location:** Notspa, CA	**Lab Work Order:** 0005542
South Bend, IN 46556	**Project Manager:** Kristin Shrader-Frechette	**Reported:** 01/25/2021

Lab ID: 0005542-03 **Sample ID:** C **Matrix:** Indoor Air

Method: TO-17 (Passive)

Analyte	CAS#	Result (µg/m³)	Q	LOQ (µg/m³)	LOD (µg/m³)	DL (µg/m³)	DF	RRT Eval	Analyzed	File ID
Dichlorodifluoromethane (Freon 12)	75-71-8	<0.427	U	0.854	0.427	0.427	1	0.00	01/05/2021 16:37	K21010507.D
Chloroform	67-66-3	<0.708	U	1.42	0.708	0.708	1	0.00	01/05/2021 16:37	K21010507.D
Carbon Tetrachloride	56-23-5	<0.576	U	1.15	0.576	0.576	1	0.00	01/05/2021 16:37	K21010507.D
Dibromomethane	74-95-3	<0.619	U	1.24	0.619	0.619	1	0.00	01/05/2021 16:37	K21010507.D
Trichloroethene	79-01-6	<0.751	U	1.50	0.751	0.751	1	0.00	01/05/2021 16:37	K21010507.D
**Tetrachloroethene**	127-18-4	**7.02**		1.21	0.604	0.604	1	0.00	01/05/2021 16:37	K21010507.D

Analyte	*CAS#*	*% Recovery*	*Recovery Limits*	*Q*	*RRT Eval*	*Analyzed*	*File ID*
Surrogate: 1,2-DCA-d4	17060-07-0	*100%*	*70-130*		*0.00*	*01/05/2021 16:37*	K21010507.D
Surrogate: Toluene-d8	2037-26-5	*95.6%*	*70-130*		*0.00*	*01/05/2021 16:37*	K21010507.D
Surrogate: Bromofluorobenzene	460-00-4	*92.7%*	*70-130*		*0.00*	*01/05/2021 16:37*	K21010507.D

BEACON ENVIRONMENTAL

2203A Commerce Road, Suite 1
Forest Hill, MD 21050 USA
1.410.838.8780

CERTIFICATE OF ANALYSIS

**University of Notre Dame**	**Site Name:** So-Cal Military Toxic Site	**Beacon Proposal:** 201201H01
Department of Biological Sciences	**Site Location:** Notspa, CA	**Lab Work Order:** 0005542
South Bend, IN 46556	**Project Manager:** Kristin Shrader-Frechette	**Reported:** 01/25/2021

Lab ID: 0005542-04 **Sample ID:** D **Matrix:** Indoor Air
Method: TO-17 (Passive)

Analyte	CAS#	Result (µg/m³)	Q	LOQ (µg/m³)	LOD (µg/m³)	DL (µg/m³)	DF	RRT Eval	Analyzed	File ID
Dichlorodifluoromethane (Freon 12)	75-71-8	<0.427	U	0.854	0.427	0.427	1	0.00	01/06/2021 09:35	K21010605.D
Chloroform	67-66-3	<0.708	U	1.42	0.708	0.708	1	0.00	01/06/2021 09:35	K21010605.D
Carbon Tetrachloride	56-23-5	<0.576	U	1.15	0.576	0.576	1	0.00	01/06/2021 09:35	K21010605.D
Dibromomethane	74-95-3	<0.619	U	1.24	0.619	0.619	1	0.00	01/06/2021 09:35	K21010605.D
Trichloroethene	79-01-6	<0.751	U	1.50	0.751	0.751	1	0.00	01/06/2021 09:35	K21010605.D
**Tetrachloroethene**	127-18-4	**12.8**		1.21	0.604	0.604	1	0.00	01/06/2021 09:35	K21010605.D

Analyte	*CAS#*	*% Recovery*	*Recovery Limits*	*Q*	*RRT Eval*	*Analyzed*	*File ID*
Surrogate: 1,2-DCA-d4	17060-07-0	*103%*	*70-130*		*0.00*	*01/06/2021 09:35*	K21010605.D
Surrogate: Toluene-d8	2037-26-5	*95.4%*	*70-130*		*0.00*	*01/06/2021 09:35*	K21010605.D
Surrogate: Bromofluorobenzene	460-00-4	*90.6%*	*70-130*		*0.00*	*01/06/2021 09:35*	K21010605.D

BEACON ENVIRONMENTAL
2203A Commerce Road, Suite 1
Forest Hill, MD 21050 USA
1.410.838.8780

CERTIFICATE OF ANALYSIS

**University of Notre Dame**	**Site Name:** So-Cal Military Toxic Site	**Beacon Proposal:** 201201H01
Department of Biological Sciences	**Site Location:** Notspa, CA	**Lab Work Order:** 0005542
South Bend, IN 46556	**Project Manager:** Kristin Shrader-Frechette	**Reported:** 01/25/2021

Lab ID: 0005542-05 **Sample ID:** E **Matrix:** Indoor Air
Method: TO-17 (Passive)

Analyte	CAS#	Result (µg/m³)	Q	LOQ (µg/m³)	LOD (µg/m³)	DL (µg/m³)	DF	RRT Eval	Analyzed	File ID
Dichlorodifluoromethane (Freon 12)	75-71-8	<0.428	U	0.856	0.428	0.428	1	0.00	01/05/2021 17:36	K21010509.D
Chloroform	67-66-3	<0.709	U	1.42	0.709	0.709	1	0.00	01/05/2021 17:36	K21010509.D
Carbon Tetrachloride	56-23-5	<0.577	U	1.15	0.577	0.577	1	0.00	01/05/2021 17:36	K21010509.D
Dibromomethane	74-95-3	<0.621	U	1.24	0.621	0.621	1	0.00	01/05/2021 17:36	K21010509.D
Trichloroethene	79-01-6	<0.752	U	1.50	0.752	0.752	1	0.00	01/05/2021 17:36	K21010509.D
**Tetrachloroethene**	127-18-4	**1.74**		1.21	0.605	0.605	1	0.00	01/05/2021 17:36	K21010509.D

Analyte	*CAS#*	*% Recovery*	*Recovery Limits*	*Q*	*RRT Eval*	*Analyzed*	*File ID*
Surrogate: 1,2-DCA-d4	17060-07-0	*95.7%*	*70-130*		*0.00*	*01/05/2021 17:36*	K21010509.D
Surrogate: Toluene-d8	2037-26-5	*97.0%*	*70-130*		*0.00*	*01/05/2021 17:36*	K21010509.D
Surrogate: Bromofluorobenzene	460-00-4	*97.2%*	*70-130*		*0.00*	*01/05/2021 17:36*	K21010509.D

BEACON ENVIRONMENTAL
2203A Commerce Road, Suite 1
Forest Hill, MD 21050 USA
1.410.838.8780

CERTIFICATE OF ANALYSIS

**University of Notre Dame**	**Site Name:** So-Cal Military Toxic Site	**Beacon Proposal:** 201201H01
Department of Biological Sciences	**Site Location:** Notspa, CA	**Lab Work Order:** 0005542
South Bend, IN 46556	**Project Manager:** Kristin Shrader-Frechette	**Reported:** 01/25/2021

Lab ID: 0005542-06 **Sample ID:** F **Matrix:** Indoor Air
Method: TO-17 (Passive)

Analyte	CAS#	Result (µg/m³)	Q	LOQ (µg/m³)	LOD (µg/m³)	DL (µg/m³)	DF	RRT Eval	Analyzed	File ID
**Dichlorodifluoromethane (Freon 12)**	75-71-8	**1.83**		0.856	0.428	0.428	1	0.00	01/05/2021 18:06	K21010510.D
Chloroform	67-66-3	<0.709	U	1.42	0.709	0.709	1	0.00	01/05/2021 18:06	K21010510.D
Carbon Tetrachloride	56-23-5	<0.577	U	1.15	0.577	0.577	1	0.00	01/05/2021 18:06	K21010510.D
Dibromomethane	74-95-3	<0.621	U	1.24	0.621	0.621	1	0.00	01/05/2021 18:06	K21010510.D
Trichloroethene	79-01-6	<0.752	U	1.50	0.752	0.752	1	0.00	01/05/2021 18:06	K21010510.D
**Tetrachloroethene**	127-18-4	**4.44**		1.21	0.605	0.605	1	0.00	01/05/2021 18:06	K21010510.D

Analyte	*CAS#*	*% Recovery*	*Recovery Limits*	*Q*	*RRT Eval*	*Analyzed*	*File ID*
Surrogate: 1,2-DCA-d4	17060-07-0	*103%*	*70-130*		*0.00*	*01/05/2021 18:06*	K21010510.D
Surrogate: Toluene-d8	2037-26-5	*96.7%*	*70-130*		*0.00*	*01/05/2021 18:06*	K21010510.D
Surrogate: Bromofluorobenzene	460-00-4	*92.4%*	*70-130*		*0.00*	*01/05/2021 18:06*	K21010510.D

BEACON ENVIRONMENTAL

2203A Commerce Road, Suite 1
Forest Hill, MD 21050 USA
1.410.838.8780

CERTIFICATE OF ANALYSIS

**University of Notre Dame**	**Site Name:** So-Cal Military Toxic Site	**Beacon Proposal:** 201201H01
Department of Biological Sciences	**Site Location:** Notspa, CA	**Lab Work Order:** 0005542
South Bend, IN 46556	**Project Manager:** Kristin Shrader-Frechette	**Reported:** 01/25/2021

Lab ID: 0005542-07 **Sample ID:** G **Matrix:** Indoor Air

Method: TO-17 (Passive)

Analyte	CAS#	Result (µg/m³)	Q	LOQ (µg/m³)	LOD (µg/m³)	DL (µg/m³)	DF	RRT Eval	Analyzed	File ID
Dichlorodifluoromethane (Freon 12)	75-71-8	<0.428	U	0.855	0.428	0.428	1	0.00	01/05/2021 18:36	K21010511.D
Chloroform	67-66-3	<0.709	U	1.42	0.709	0.709	1	0.00	01/05/2021 18:36	K21010511.D
Carbon Tetrachloride	56-23-5	<0.577	U	1.15	0.577	0.577	1	0.00	01/05/2021 18:36	K21010511.D
Dibromomethane	74-95-3	<0.620	U	1.24	0.620	0.620	1	0.00	01/05/2021 18:36	K21010511.D
Trichloroethene	79-01-6	<0.752	U	1.50	0.752	0.752	1	0.00	01/05/2021 18:36	K21010511.D
**Tetrachloroethene**	127-18-4	**2.61**		1.21	0.605	0.605	1	0.00	01/05/2021 18:36	K21010511.D

Analyte	*CAS#*	*% Recovery*	*Recovery Limits*	*Q*	*RRT Eval*	*Analyzed*	*File ID*
Surrogate: 1,2-DCA-d4	17060-07-0	*100%*	*70-130*		*0.00*	*01/05/2021 18:36*	K21010511.D
Surrogate: Toluene-d8	2037-26-5	*95.9%*	*70-130*		*0.00*	*01/05/2021 18:36*	K21010511.D
Surrogate: Bromofluorobenzene	460-00-4	*94.0%*	*70-130*		*0.00*	*01/05/2021 18:36*	K21010511.D

BEACON ENVIRONMENTAL
2203A Commerce Road, Suite 1
Forest Hill, MD 21050 USA
1.410.838.8780

CERTIFICATE OF ANALYSIS

**University of Notre Dame**	**Site Name:** So-Cal Military Toxic Site	**Beacon Proposal:** 201201H01
Department of Biological Sciences	**Site Location:** Notspa, CA	**Lab Work Order:** 0005542
South Bend, IN 46556	**Project Manager:** Kristin Shrader-Frechette	**Reported:** 01/25/2021

Lab ID: 0005542-08 **Sample ID:** H **Matrix:** Indoor Air

Method: TO-17 (Passive)

Analyte	CAS#	Result (µg/m³)	Q	LOQ (µg/m³)	LOD (µg/m³)	DL (µg/m³)	DF	RRT Eval	Analyzed	File ID
Dichlorodifluoromethane (Freon 12)	75-71-8	<0.428	U	0.855	0.428	0.428	1	0.00	01/06/2021 10:05	K21010606.D
Chloroform	67-66-3	<0.709	U	1.42	0.709	0.709	1	0.00	01/06/2021 10:05	K21010606.D
Carbon Tetrachloride	56-23-5	<0.577	U	1.15	0.577	0.577	1	0.00	01/06/2021 10:05	K21010606.D
Dibromomethane	74-95-3	<0.620	U	1.24	0.620	0.620	1	0.00	01/06/2021 10:05	K21010606.D
Trichloroethene	79-01-6	<0.752	U	1.50	0.752	0.752	1	0.00	01/06/2021 10:05	K21010606.D
**Tetrachloroethene**	127-18-4	**1.43**		1.21	0.605	0.605	1	0.00	01/06/2021 10:05	K21010606.D

Analyte	*CAS#*	*% Recovery*	*Recovery Limits*	*Q*	*RRT Eval*	*Analyzed*	*File ID*
Surrogate: 1,2-DCA-d4	17060-07-0	*102%*	*70-130*		*0.00*	*01/06/2021 10:05*	K21010606.D
Surrogate: Toluene-d8	2037-26-5	*100%*	*70-130*		*0.00*	*01/06/2021 10:05*	K21010606.D
Surrogate: Bromofluorobenzene	460-00-4	*95.5%*	*70-130*		*0.00*	*01/06/2021 10:05*	K21010606.D

2203A Commerce Road, Suite 1
Forest Hill, MD 21050 USA
1.410.838.8780

CERTIFICATE OF ANALYSIS

**University of Notre Dame**	**Site Name:** So-Cal Military Toxic Site	**Beacon Proposal:** 201201H01
Department of Biological Sciences	**Site Location:** Notspa, CA	**Lab Work Order:** 0005542
South Bend, IN 46556	**Project Manager:** Kristin Shrader-Frechette	**Reported:** 01/25/2021

Lab ID: 0005542-09 **Sample ID:** I **Matrix:** Indoor Air

Method: TO-17 (Passive)

Analyte	CAS#	Result (µg/m³)	Q	LOQ (µg/m³)	LOD (µg/m³)	DL (µg/m³)	DF	RRT Eval	Analyzed	File ID
**Dichlorodifluoromethane (Freon 12)**	75-71-8	**1.79**		0.856	0.428	0.428	1	0.00	01/05/2021 19:37	K21010513.D
Chloroform	67-66-3	<0.709	U	1.42	0.709	0.709	1	0.00	01/05/2021 19:37	K21010513.D
Carbon Tetrachloride	56-23-5	<0.577	U	1.15	0.577	0.577	1	0.00	01/05/2021 19:37	K21010513.D
Dibromomethane	74-95-3	<0.620	U	1.24	0.620	0.620	1	0.00	01/05/2021 19:37	K21010513.D
Trichloroethene	79-01-6	<0.752	U	1.50	0.752	0.752	1	0.00	01/05/2021 19:37	K21010513.D
**Tetrachloroethene**	127-18-4	**2.92**		1.21	0.605	0.605	1	0.00	01/05/2021 19:37	K21010513.D

Analyte	*CAS#*	*% Recovery*	*Recovery Limits*	*Q*	*RRT Eval*	*Analyzed*	*File ID*
Surrogate: 1,2-DCA-d4	17060-07-0	*98.2%*	*70-130*		*0.00*	*01/05/2021 19:37*	K21010513.D
Surrogate: Toluene-d8	2037-26-5	*95.4%*	*70-130*		*0.00*	*01/05/2021 19:37*	K21010513.D
Surrogate: Bromofluorobenzene	460-00-4	*95.3%*	*70-130*		*0.00*	*01/05/2021 19:37*	K21010513.D

BEACON ENVIRONMENTAL

2203A Commerce Road, Suite 1
Forest Hill, MD 21050 USA
1.410.838.8780

CERTIFICATE OF ANALYSIS

**University of Notre Dame**	**Site Name:** So-Cal Military Toxic Site	**Beacon Proposal:** 201201H01
Department of Biological Sciences	**Site Location:** Notspa, CA	**Lab Work Order:** 0005542
South Bend, IN 46556	**Project Manager:** Kristin Shrader-Frechette	**Reported:** 01/25/2021

Lab ID: 0005542-10 **Sample ID:** J **Matrix:** Indoor Air

Method: TO-17 (Passive)

Analyte	CAS#	Result (µg/m³)	Q	LOQ (µg/m³)	LOD (µg/m³)	DL (µg/m³)	DF	RRT Eval	Analyzed	File ID
Dichlorodifluoromethane (Freon 12)	75-71-8	<0.428	U	0.856	0.428	0.428	1	0.00	01/05/2021 20:06	K21010514.D
Chloroform	67-66-3	<0.709	U	1.42	0.709	0.709	1	0.00	01/05/2021 20:06	K21010514.D
Carbon Tetrachloride	56-23-5	<0.577	U	1.15	0.577	0.577	1	0.00	01/05/2021 20:06	K21010514.D
Dibromomethane	74-95-3	<0.620	U	1.24	0.620	0.620	1	0.00	01/05/2021 20:06	K21010514.D
Trichloroethene	79-01-6	<0.752	U	1.50	0.752	0.752	1	0.00	01/05/2021 20:06	K21010514.D
**Tetrachloroethene**	127-18-4	**1.50**		1.21	0.605	0.605	1	0.00	01/05/2021 20:06	K21010514.D

Analyte	*CAS#*	*% Recovery*	*Recovery Limits*	*Q*	*RRT Eval*	*Analyzed*	*File ID*
Surrogate: 1,2-DCA-d4	17060-07-0	*89.0%*	*70-130*		*0.00*	*01/05/2021 20:06*	K21010514.D
Surrogate: Toluene-d8	2037-26-5	*96.3%*	*70-130*		*0.00*	*01/05/2021 20:06*	K21010514.D
Surrogate: Bromofluorobenzene	460-00-4	*95.7%*	*70-130*		*0.00*	*01/05/2021 20:06*	K21010514.D

BEACON ENVIRONMENTAL
2203A Commerce Road, Suite 1
Forest Hill, MD 21050 USA
1.410.838.8780

CERTIFICATE OF ANALYSIS

**University of Notre Dame**	**Site Name:** So-Cal Military Toxic Site	**Beacon Proposal:** 201201H01
Department of Biological Sciences	**Site Location:** Notspa, CA	**Lab Work Order:** 0005542
South Bend, IN 46556	**Project Manager:** Kristin Shrader-Frechette	**Reported:** 01/25/2021

Lab ID: 0005542-11 **Sample ID:** J-DUP **Matrix:** Indoor Air

Method: TO-17 (Passive)

Analyte	CAS#	Result (µg/m³)	Q	LOQ (µg/m³)	LOD (µg/m³)	DL (µg/m³)	DF	RRT Eval	Analyzed	File ID
Dichlorodifluoromethane (Freon 12)	75-71-8	<0.428	U	0.856	0.428	0.428	1	0.00	01/05/2021 20:37	K21010515.D
Chloroform	67-66-3	<0.709	U	1.42	0.709	0.709	1	0.00	01/05/2021 20:37	K21010515.D
Carbon Tetrachloride	56-23-5	<0.577	U	1.15	0.577	0.577	1	0.00	01/05/2021 20:37	K21010515.D
Dibromomethane	74-95-3	<0.620	U	1.24	0.620	0.620	1	0.00	01/05/2021 20:37	K21010515.D
Trichloroethene	79-01-6	<0.752	U	1.50	0.752	0.752	1	0.00	01/05/2021 20:37	K21010515.D
**Tetrachloroethene**	127-18-4	**1.63**		1.21	0.605	0.605	1	0.00	01/05/2021 20:37	K21010515.D

Analyte	*CAS#*	*% Recovery*	*Recovery Limits*	*Q*	*RRT Eval*	*Analyzed*	*File ID*
Surrogate: 1,2-DCA-d4	17060-07-0	*91.8%*	*70-130*		*0.00*	*01/05/2021 20:37*	K21010515.D
Surrogate: Toluene-d8	2037-26-5	*91.9%*	*70-130*		*0.00*	*01/05/2021 20:37*	K21010515.D
Surrogate: Bromofluorobenzene	460-00-4	*95.5%*	*70-130*		*0.00*	*01/05/2021 20:37*	K21010515.D

BEACON ENVIRONMENTAL

2203A Commerce Road, Suite 1
Forest Hill, MD 21050 USA
1.410.838.8780

CERTIFICATE OF ANALYSIS

**University of Notre Dame**	**Site Name:** So-Cal Military Toxic Site	**Beacon Proposal:** 201201H01
Department of Biological Sciences	**Site Location:** Notspa, CA	**Lab Work Order:** 0005542
South Bend, IN 46556	**Project Manager:** Kristin Shrader-Frechette	**Reported:** 01/25/2021

Lab ID: 0005542-12 **Sample ID:** K **Matrix:** Indoor Air

Method: TO-17 (Passive)

Analyte	CAS#	Result (μg/m³)	Q	LOQ (μg/m³)	LOD (μg/m³)	DL (μg/m³)	DF	RRT Eval	Analyzed	File ID
**Dichlorodifluoromethane (Freon 12)**	75-71-8	**0.464**	J	0.855	0.428	0.428	1	0.00	01/05/2021 21:06	K21010516.D
Chloroform	67-66-3	<0.709	U	1.42	0.709	0.709	1	0.00	01/05/2021 21:06	K21010516.D
Carbon Tetrachloride	56-23-5	<0.577	U	1.15	0.577	0.577	1	0.00	01/05/2021 21:06	K21010516.D
Dibromomethane	74-95-3	<0.620	U	1.24	0.620	0.620	1	0.00	01/05/2021 21:06	K21010516.D
Trichloroethene	79-01-6	<0.752	U	1.50	0.752	0.752	1	0.00	01/05/2021 21:06	K21010516.D
**Tetrachloroethene**	127-18-4	**1.71**		1.21	0.605	0.605	1	0.00	01/05/2021 21:06	K21010516.D

Analyte	*CAS#*	*% Recovery*	*Recovery Limits*	*Q*	*RRT Eval*	*Analyzed*	*File ID*
Surrogate: 1,2-DCA-d4	17060-07-0	*92.1%*	*70-130*		*0.00*	*01/05/2021 21:06*	K21010516.D
Surrogate: Toluene-d8	2037-26-5	*92.4%*	*70-130*		*0.00*	*01/05/2021 21:06*	K21010516.D
Surrogate: Bromofluorobenzene	460-00-4	*93.0%*	*70-130*		*0.00*	*01/05/2021 21:06*	K21010516.D

BEACON ENVIRONMENTAL

2203A Commerce Road, Suite 1
Forest Hill, MD 21050 USA
1.410.838.8780

CERTIFICATE OF ANALYSIS

**University of Notre Dame**	**Site Name:**	So-Cal Military Toxic Site	**Beacon Proposal:**	201201H01
Department of Biological Sciences	**Site Location:**	Notspa, CA	**Lab Work Order:**	0005542
South Bend, IN 46556	**Project Manager:**	Kristin Shrader-Frechette	**Reported:**	01/25/2021

QC Information/Summary

BEACON ENVIRONMENTAL

2203A Commerce Road, Suite 1
Forest Hill, MD 21050 USA
1.410.838.8780

CERTIFICATE OF ANALYSIS

**University of Notre Dame**	**Site Name:** So-Cal Military Toxic Site	**Beacon Proposal:** 201201H01
Department of Biological Sciences	**Site Location:** Notspa, CA	**Lab Work Order:** 0005542
South Bend, IN 46556	**Project Manager:** Kristin Shrader-Frechette	**Reported:** 01/25/2021

Organics in Air by EPA TO-17 Using Beacon Sampler - Quality Control Summary

Analyte	Result	LOQ	LOD	DL	Units	Spike Level	Source Result	%REC	%REC Limits	RPD	RPD Limit	Notes
**Sequence: B20L066 - Instrument: K System - File ID: Kd20122316.D**												
B20L066-ICV1 (LCSD/Second Source Verification/CALV)												
Dichlorodifluoromethane (Freon 12)	57.9	10	5	5	ng	50.0		116	70-130			
Chloroform	47.0	10	5	5	ng	50.0		94.0	70-130			
Carbon Tetrachloride	48.2	10	5	5	ng	50.0		96.4	70-130			
Dibromomethane	50.4	10	5	5	ng	50.0		101	70-130			
Trichloroethene	48.7	10	5	5	ng	50.0		97.4	70-130			
Tetrachloroethene	48.2	10	5	5	ng	50.0		96.3	70-130			
Surrogate: 1,2-DCA-d4	*48.1*				*ng*	50.0		*96.1*	*70-130*			
Surrogate: Toluene-d8	*48.8*				*ng*	50.0		*97.5*	*70-130*			
Surrogate: Bromofluorobenzene	*48.3*				*ng*	50.0		*96.6*	*70-130*			

BEACON ENVIRONMENTAL

2203A Commerce Road, Suite 1
Forest Hill, MD 21050 USA
1.410.838.8780

CERTIFICATE OF ANALYSIS

**University of Notre Dame**	**Site Name:** So-Cal Military Toxic Site	**Beacon Proposal:** 201201H01
Department of Biological Sciences	**Site Location:** Notspa, CA	**Lab Work Order:** 0005542
South Bend, IN 46556	**Project Manager:** Kristin Shrader-Frechette	**Reported:** 01/25/2021

Organics in Air by EPA TO-17 Using Beacon Sampler - Quality Control Summary

Analyte	Result	LOQ	LOD	DL	Units	Spike Level	Source Result	%REC	%REC Limits	RPD	RPD Limit	Notes
**Sequence: B20L066 - Instrument: K System - File ID: Kd20122318.D**												
B20L066-ICB1 (Lab Blank/Initial Calibration Blank)												
Dichlorodifluoromethane (Freon 12)	<5	10	5	5	ng							U
Chloroform	<5	10	5	5	ng							U
Carbon Tetrachloride	<5	10	5	5	ng							U
Dibromomethane	<5	10	5	5	ng							U
Trichloroethene	<5	10	5	5	ng							U
Tetrachloroethene	<5	10	5	5	ng							U
Surrogate: 1,2-DCA-d4	*95.0*				*ng*	100		*95.0*	*70-130*			
Surrogate: Toluene-d8	*97.0*				*ng*	100		*97.0*	*70-130*			
Surrogate: Bromofluorobenzene	*93.5*				*ng*	100		*93.5*	*70-130*			

2203A Commerce Road, Suite 1
Forest Hill, MD 21050 USA
1.410.838.8780

CERTIFICATE OF ANALYSIS

**University of Notre Dame**	**Site Name:** So-Cal Military Toxic Site	**Beacon Proposal:** 201201H01
Department of Biological Sciences	**Site Location:** Notspa, CA	**Lab Work Order:** 0005542
South Bend, IN 46556	**Project Manager:** Kristin Shrader-Frechette	**Reported:** 01/25/2021

Organics in Air by EPA TO-17 Using Beacon Sampler - Quality Control Summary

Analyte	Result	LOQ	LOD	DL	Units	Spike Level	Source Result	%REC	%REC Limits	RPD	RPD Limit	Notes
**Sequence: B21A005 - Batch: 21A0006 - Instrument: K System - File ID: K21010502.D**												
21A0006-BS1 (LCS, Calibration Source Verification)												
Dichlorodifluoromethane (Freon 12)	50.5	10	5	5	ng	50.0		101	70-130			
Chloroform	50.8	10	5	5	ng	50.0		102	70-130			
Carbon Tetrachloride	47.5	10	5	5	ng	50.0		95.1	70-130			
Dibromomethane	53.8	10	5	5	ng	50.0		108	70-130			
Trichloroethene	51.7	10	5	5	ng	50.0		103	70-130			
Tetrachloroethene	52.2	10	5	5	ng	50.0		104	70-130			
Surrogate: 1,2-DCA-d4	*54.5*				*ng*	50.0		*109*	*70-130*			
Surrogate: Toluene-d8	*51.2*				*ng*	50.0		*102*	*70-130*			
Surrogate: Bromofluorobenzene	*45.7*				*ng*	50.0		*91.3*	*70-130*			

2203A Commerce Road, Suite 1
Forest Hill, MD 21050 USA
1.410.838.8780

BEACON ENVIRONMENTAL

CERTIFICATE OF ANALYSIS

**University of Notre Dame**	**Site Name:** So-Cal Military Toxic Site	**Beacon Proposal:** 201201H01
Department of Biological Sciences	**Site Location:** Notspa, CA	**Lab Work Order:** 0005542
South Bend, IN 46556	**Project Manager:** Kristin Shrader-Frechette	**Reported:** 01/25/2021

Organics in Air by EPA TO-17 Using Beacon Sampler - Quality Control Summary

Analyte	Result	LOQ	LOD	DL	Units	Spike Level	Source Result	%REC	%REC Limits	RPD	RPD Limit	Notes
**Sequence: B21A005 - Batch: 21A0006 - Instrument: K System - File ID: K21010503.D**												
21A0006-BLK1 (Lab Blank)												
Dichlorodifluoromethane (Freon 12)	<0.427	0.854	0.427	0.427	µg/m³							U
Chloroform	<0.708	1.42	0.708	0.708	µg/m³							U
Carbon Tetrachloride	<0.576	1.15	0.576	0.576	µg/m³							U
Dibromomethane	<0.619	1.24	0.619	0.619	µg/m³							U
Trichloroethene	<0.751	1.50	0.751	0.751	µg/m³							U
Tetrachloroethene	<0.604	1.21	0.604	0.604	µg/m³							U
Surrogate: 1,2-DCA-d4	*101*				*ng*	100		*101*	*70-130*			
Surrogate: Toluene-d8	*101*				*ng*	100		*101*	*70-130*			
Surrogate: Bromofluorobenzene	*89.6*				*ng*	100		*89.6*	*70-130*			

2203A Commerce Road, Suite 1
Forest Hill, MD 21050 USA
1.410.838.8780

BEACON ENVIRONMENTAL

CERTIFICATE OF ANALYSIS

**University of Notre Dame**	**Site Name:** So-Cal Military Toxic Site	**Beacon Proposal:** 201201H01
Department of Biological Sciences	**Site Location:** Notspa, CA	**Lab Work Order:** 0005542
South Bend, IN 46556	**Project Manager:** Kristin Shrader-Frechette	**Reported:** 01/25/2021

Organics in Air by EPA TO-17 Using Beacon Sampler - Quality Control Summary

Analyte	Result	LOQ	LOD	DL	Units	Spike Level	Source Result	%REC	%REC Limits	RPD	RPD Limit	Notes
**Sequence: B21A005 - Instrument: K System - File ID: K21010504.D**												
B21A005-ICV1 (LCSD/Second Source Verification/CALV)												
Dichlorodifluoromethane (Freon 12)	57.7	10	5	5	ng	50.0		115	70-130			
Chloroform	47.5	10	5	5	ng	50.0		95.0	70-130			
Carbon Tetrachloride	46.6	10	5	5	ng	50.0		93.1	70-130			
Dibromomethane	54.4	10	5	5	ng	50.0		109	70-130			
Trichloroethene	50.9	10	5	5	ng	50.0		102	70-130			
Tetrachloroethene	49.6	10	5	5	ng	50.0		99.2	70-130			
Surrogate: 1,2-DCA-d4	*50.2*				*ng*	50.0		*100*	*70-130*			
Surrogate: Toluene-d8	*48.0*				*ng*	50.0		*96.1*	*70-130*			
Surrogate: Bromofluorobenzene	*44.6*				*ng*	50.0		*89.2*	*70-130*			

BEACON ENVIRONMENTAL

2203A Commerce Road, Suite 1
Forest Hill, MD 21050 USA
1.410.838.8780

CERTIFICATE OF ANALYSIS

**University of Notre Dame**	**Site Name:** So-Cal Military Toxic Site	**Beacon Proposal:** 201201H01
Department of Biological Sciences	**Site Location:** Notspa, CA	**Lab Work Order:** 0005542
South Bend, IN 46556	**Project Manager:** Kristin Shrader-Frechette	**Reported:** 01/25/2021

Organics in Air by EPA TO-17 Using Beacon Sampler - Quality Control Summary

Analyte	Result	LOQ	LOD	DL	Units	Spike Level	Source Result	%REC	%REC Limits	RPD	RPD Limit	Notes
**Sequence: B21A005 - Instrument: K System - File ID: K21010517.D**												
B21A005-CCV1 (LCS, Closing Calibration Verification)												
Dichlorodifluoromethane (Freon 12)	82.3	10	5	5	ng	50.0		165	50-150			L, L
Chloroform	50.6	10	5	5	ng	50.0		101	50-150			
Carbon Tetrachloride	50.2	10	5	5	ng	50.0		100	50-150			
Dibromomethane	51.4	10	5	5	ng	50.0		103	50-150			
Trichloroethene	49.5	10	5	5	ng	50.0		99.0	50-150			
Tetrachloroethene	51.5	10	5	5	ng	50.0		103	50-150			
Surrogate: 1,2-DCA-d4	*49.9*				*ng*	50.0		*99.9*	*50-150*			
Surrogate: Toluene-d8	*49.5*				*ng*	50.0		*98.9*	*70-130*			
Surrogate: Bromofluorobenzene	*48.0*				*ng*	50.0		*95.9*	*70-130*			

2203A Commerce Road, Suite 1
Forest Hill, MD 21050 USA
1.410.838.8780

BEACON ENVIRONMENTAL

CERTIFICATE OF ANALYSIS

**University of Notre Dame**	**Site Name:** So-Cal Military Toxic Site	**Beacon Proposal:** 201201H01
Department of Biological Sciences	**Site Location:** Notspa, CA	**Lab Work Order:** 0005542
South Bend, IN 46556	**Project Manager:** Kristin Shrader-Frechette	**Reported:** 01/25/2021

Organics in Air by EPA TO-17 Using Beacon Sampler - Quality Control Summary

Analyte	Result	LOQ	LOD	DL	Units	Spike Level	Source Result	%REC	%REC Limits	RPD	RPD Limit	Notes
**Sequence: B21A006 - Batch: 21A0007 - Instrument: K System - File ID: K21010602.D**												
21A0007-BS1 (LCS, Calibration Source Verification)												
Dichlorodifluoromethane (Freon 12)	42.1	10	5	5	ng	50.0		84.1	70-130			
Chloroform	50.7	10	5	5	ng	50.0		101	70-130			
Carbon Tetrachloride	47.5	10	5	5	ng	50.0		94.9	70-130			
Dibromomethane	52.6	10	5	5	ng	50.0		105	70-130			
Trichloroethene	51.4	10	5	5	ng	50.0		103	70-130			
Tetrachloroethene	50.9	10	5	5	ng	50.0		102	70-130			
Surrogate: 1,2-DCA-d4	*53.9*				*ng*	50.0		*108*	*70-130*			
Surrogate: Toluene-d8	*50.0*				*ng*	50.0		*100*	*70-130*			
Surrogate: Bromofluorobenzene	*46.0*				*ng*	50.0		*92.0*	*70-130*			

2203A Commerce Road, Suite 1
Forest Hill, MD 21050 USA
1.410.838.8780

BEACON ENVIRONMENTAL

CERTIFICATE OF ANALYSIS

**University of Notre Dame**	**Site Name:** So-Cal Military Toxic Site	**Beacon Proposal:** 201201H01
Department of Biological Sciences	**Site Location:** Notspa, CA	**Lab Work Order:** 0005542
South Bend, IN 46556	**Project Manager:** Kristin Shrader-Frechette	**Reported:** 01/25/2021

Organics in Air by EPA TO-17 Using Beacon Sampler - Quality Control Summary

Analyte	Result	LOQ	LOD	DL	Units	Spike Level	Source Result	%REC	%REC Limits	RPD	RPD Limit	Notes
**Sequence: B21A006 - Batch: 21A0007 - Instrument: K System - File ID: K21010603.D**												
21A0007-BLK1 (Lab Blank)												
Dichlorodifluoromethane (Freon 12)	<0.427	0.854	0.427	0.427	µg/m³							U
Chloroform	<0.708	1.42	0.708	0.708	µg/m³							U
Carbon Tetrachloride	<0.576	1.15	0.576	0.576	µg/m³							U
Dibromomethane	<0.619	1.24	0.619	0.619	µg/m³							U
Trichloroethene	<0.751	1.50	0.751	0.751	µg/m³							U
Tetrachloroethene	<0.604	1.21	0.604	0.604	µg/m³							U
Surrogate: 1,2-DCA-d4	*98.7*				*ng*	100		*98.7*	*70-130*			
Surrogate: Toluene-d8	*99.3*				*ng*	100		*99.3*	*70-130*			
Surrogate: Bromofluorobenzene	*90.0*				*ng*	100		*90.0*	*70-130*			

2203A Commerce Road, Suite 1
Forest Hill, MD 21050 USA
1.410.838.8780

BEACON ENVIRONMENTAL

CERTIFICATE OF ANALYSIS

**University of Notre Dame**	**Site Name:** So-Cal Military Toxic Site	**Beacon Proposal:** 201201H01
Department of Biological Sciences	**Site Location:** Notspa, CA	**Lab Work Order:** 0005542
South Bend, IN 46556	**Project Manager:** Kristin Shrader-Frechette	**Reported:** 01/25/2021

Organics in Air by EPA TO-17 Using Beacon Sampler - Quality Control Summary

Sequence: B21A006 - Instrument: K System - File ID: K21010604.D

B21A006-ICV1 (LCSD/Second Source Verification/CALV)

Analyte	Result	LOQ	LOD	DL	Units	Spike Level	Source Result	%REC	%REC Limits	RPD	RPD Limit	Notes
Dichlorodifluoromethane (Freon 12)	43.7	10	5	5	ng	50.0		87.3	70-130			
Chloroform	51.5	10	5	5	ng	50.0		103	70-130			
Carbon Tetrachloride	48.0	10	5	5	ng	50.0		96.0	70-130			
Dibromomethane	52.9	10	5	5	ng	50.0		106	70-130			
Trichloroethene	52.2	10	5	5	ng	50.0		104	70-130			
Tetrachloroethene	50.5	10	5	5	ng	50.0		101	70-130			
Surrogate: 1,2-DCA-d4	*52.3*				*ng*	50.0		*105*	*70-130*			
Surrogate: Toluene-d8	*50.0*				*ng*	50.0		*100*	*70-130*			
Surrogate: Bromofluorobenzene	*46.3*				*ng*	50.0		*92.5*	*70-130*			

BEACON ENVIRONMENTAL

2203A Commerce Road, Suite 1
Forest Hill, MD 21050 USA
1.410.838.8780

CERTIFICATE OF ANALYSIS

**University of Notre Dame**	**Site Name:** So-Cal Military Toxic Site	**Beacon Proposal:** 201201H01
Department of Biological Sciences	**Site Location:** Notspa, CA	**Lab Work Order:** 0005542
South Bend, IN 46556	**Project Manager:** Kristin Shrader-Frechette	**Reported:** 01/25/2021

Organics in Air by EPA TO-17 Using Beacon Sampler - Quality Control Summary

Analyte	Result	LOQ	LOD	DL	Units	Spike Level	Source Result	%REC	%REC Limits	RPD	RPD Limit	Notes
**Sequence: B21A006 - Instrument: K System - File ID: K21010607.D**												
B21A006-CCV1 (LCS, Closing Calibration Verification)												
Dichlorodifluoromethane (Freon 12)	52.0	10	5	5	ng	50.0		104	50-150			
Chloroform	50.3	10	5	5	ng	50.0		101	50-150			
Carbon Tetrachloride	48.1	10	5	5	ng	50.0		96.1	50-150			
Dibromomethane	51.4	10	5	5	ng	50.0		103	50-150			
Trichloroethene	50.4	10	5	5	ng	50.0		101	50-150			
Tetrachloroethene	50.4	10	5	5	ng	50.0		101	50-150			
Surrogate: 1,2-DCA-d4	*50.2*				*ng*	50.0		*100*	*50-150*			
Surrogate: Toluene-d8	*50.5*				*ng*	50.0		*101*	*70-130*			
Surrogate: Bromofluorobenzene	*45.9*				*ng*	50.0		*91.8*	*70-130*			

BEACON ENVIRONMENTAL
2203A Commerce Road, Suite 1
Forest Hill, MD 21050 USA
1.410.838.8780

CERTIFICATE OF ANALYSIS

**University of Notre Dame**	**Site Name:** So-Cal Military Toxic Site	**Beacon Proposal:** 201201H01
Department of Biological Sciences	**Site Location:** Notspa, CA	**Lab Work Order:** 0005542
South Bend, IN 46556	**Project Manager:** Kristin Shrader-Frechette	**Reported:** 01/25/2021

TO-17 (Passive) - LCS/LCSD Quality Control Summary

LCS: 21A0006-BS1 File ID: K21010502.D Analyzed: 1/5/21 15:08
LCSD: B21A005-ICV1 File ID: K21010504.D Analyzed: 1/5/21 14:16

Analyte	CAS#	LCS Result (ng)	%REC	Q	Spike Level (ng)	LCSD Result (ng)	%REC	%REC Limits	RPD	RPD Limit	Q
Dichlorodifluoromethane (Freon 12)	75-71-8	50.51	101.02		50	57.73	115.00	70-130	13.34	30	
Chloroform	67-66-3	50.75	101.5		50	47.51	95.00	70-130	6.59	30	
Carbon Tetrachloride	56-23-5	47.54	95.08		50	46.57	93.10	70-130	2.06	30	
Dibromomethane	74-95-3	53.83	107.66		50	54.42	109.00	70-130	1.09	30	
Trichloroethene	79-01-6	51.67	103.34		50	50.92	102.00	70-130	1.46	30	
Tetrachloroethene	127-18-4	52.16	104.32		50	49.62	99.20	70-130	4.99	30	

2203A Commerce Road, Suite 1
Forest Hill, MD 21050 USA
1.410.838.8780

CERTIFICATE OF ANALYSIS

**University of Notre Dame**	**Site Name:** So-Cal Military Toxic Site	**Beacon Proposal:** 201201H01
Department of Biological Sciences	**Site Location:** Notspa, CA	**Lab Work Order:** 0005542
South Bend, IN 46556	**Project Manager:** Kristin Shrader-Frechette	**Reported:** 01/25/2021

TO-17 (Passive) - LCS/LCSD Quality Control Summary

LCS: 21A0007-BS1 File ID: K21010602.D Analyzed: 1/6/21 9:05
LCSD: B21A006-ICV1 File ID: K21010604.D Analyzed: 1/6/21 8:13

Analyte	CAS#	LCS Result (ng)	%REC	Q	Spike Level (ng)	LCSD Result (ng)	%REC	%REC Limits	RPD	RPD Limit	Q
Dichlorodifluoromethane (Freon 12)	75-71-8	42.06	84.12		50	43.67	87.30	70-130	3.76	30	
Chloroform	67-66-3	50.72	101.44		50	51.46	103.00	70-130	1.45	30	
Carbon Tetrachloride	56-23-5	47.46	94.92		50	48.01	96.00	70-130	1.15	30	
Dibromomethane	74-95-3	52.63	105.26		50	52.91	106.00	70-130	0.53	30	
Trichloroethene	79-01-6	51.38	102.76		50	52.18	104.00	70-130	1.54	30	
Tetrachloroethene	127-18-4	50.91	101.82		50	50.45	101.00	70-130	0.91	30	

BEACON ENVIRONMENTAL

2203A Commerce Road, Suite 1
Forest Hill, MD 21050 USA
1.410.838.8780

CERTIFICATE OF ANALYSIS

**University of Notre Dame**	**Site Name:** So-Cal Military Toxic Site	**Beacon Proposal:** 201201H01
Department of Biological Sciences	**Site Location:** Notspa, CA	**Lab Work Order:** 0005542
South Bend, IN 46556	**Project Manager:** Kristin Shrader-Frechette	**Reported:** 01/25/2021

Sample Duplicate RPD Summary

Organics in Air by EPA TO-17 Using Beacon Sampler

Duplicate Sample: J-DUP (0005542-11) ***Sample: J (0005542-10)*** ***Average RPD:*** 1.4%

Analyte	CAS#	Duplicate Result (µg/m³)	LOD (µg/m³)	Q	Sample Result (µg/m³)	RPD (%)	RPD Limit	Q
Dichlorodifluoromethane (Freon 12)	75-71-8	<0.428	0.428	U	<0.428	0.0	25	U
Chloroform	67-66-3	<0.709	0.709	U	<0.709	0.0	25	U
Carbon Tetrachloride	56-23-5	<0.577	0.577	U	<0.577	0.0	25	U
Dibromomethane	74-95-3	<0.620	0.620	U	<0.620	0.0	25	U
Trichloroethene	79-01-6	<0.752	0.752	U	<0.752	0.0	25	U
Tetrachloroethene	127-18-4	1.63	0.605		1.50	8.3	25	

Notes: + Field Duplicate RPD out of laboratory acceptance limits.

2203A Commerce Road, Suite 1
Forest Hill, MD 21050 USA
1.410.838.8780

CERTIFICATE OF ANALYSIS

**University of Notre Dame**		**Site Name:**	So-Cal Military Toxic Site	**Beacon Proposal:**	201201H01
Department of Biological Sciences		**Site Location:**	Notspa, CA	**Lab Work Order:**	0005542
South Bend, IN 46556		**Project Manager:**	Kristin Shrader-Frechette	**Reported:**	01/25/2021

QC/CLP Tables

BEACON ENVIRONMENTAL

2203A Commerce Road, Suite 1
Forest Hill, MD 21050 USA
1.410.838.8780

CERTIFICATE OF ANALYSIS

**University of Notre Dame**	**Site Name:** So-Cal Military Toxic Site	**Beacon Proposal:** 201201H01
Department of Biological Sciences	**Site Location:** Notspa, CA	**Lab Work Order:** 0005542
South Bend, IN 46556	**Project Manager:** Kristin Shrader-Frechette	**Reported:** 01/25/2021

Form 1
Volatile Analysis Data Package Sequence Summary

Method: TO-17 (Passive)

Sequence: B21A005 **Instrument:** K System

Lab Sample ID	Client Sample ID	DF	File ID	QC Description
B21A005-TUN1	MS Tune	1.00	K21010501.D	MS Tune
21A0006-BS1	LCS	1.00	K21010502.D	LCS, Calibration Source Verification
21A0006-BLK1	Blank	1.00	K21010503.D	Method Blank
B21A005-ICV1	Initial Cal Check	1.00	K21010504.D	LCSD, Second Source Verification/ICV
0005542-01	A	1.00	K21010505.D	
0005542-02	B	1.00	K21010506.D	
0005542-03	C	1.00	K21010507.D	
0005542-05	E	1.00	K21010509.D	
0005542-06	F	1.00	K21010510.D	
0005542-07	G	1.00	K21010511.D	
0005542-09	I	1.00	K21010513.D	
0005542-10	J	1.00	K21010514.D	
0005542-11	J-DUP	1.00	K21010515.D	
0005542-12	K	1.00	K21010516.D	
B21A005-CCV1	Calibration Check	1.00	K21010517.D	Closing Calibration Verification

2203A Commerce Road, Suite 1
Forest Hill, MD 21050 USA
1.410.838.8780

BEACON ENVIRONMENTAL

CERTIFICATE OF ANALYSIS

**University of Notre Dame**	**Site Name:** So-Cal Military Toxic Site	**Beacon Proposal:** 201201H01
Department of Biological Sciences	**Site Location:** Notspa, CA	**Lab Work Order:** 0005542
South Bend, IN 46556	**Project Manager:** Kristin Shrader-Frechette	**Reported:** 01/25/2021

Form 1
Volatile Analysis Data Package Sequence Summary

Method: TO-17 (Passive)

Sequence: B21A006 **Instrument:** K System

Lab Sample ID	Client Sample ID	DF	File ID	QC Description
B21A006-TUN1	MS Tune	1.00	K21010601.D	MS Tune
21A0007-BS1	LCS	1.00	K21010602.D	LCS, Calibration Source Verification
21A0007-BLK1	Blank	1.00	K21010603.D	Method Blank
B21A006-ICV1	Initial Cal Check	1.00	K21010604.D	LCSD, Second Source Verification/ICV
0005542-04	D	1.00	K21010605.D	
0005542-08	H	1.00	K21010606.D	
B21A006-CCV1	Calibration Check	1.00	K21010607.D	Closing Calibration Verification

BEACON ENVIRONMENTAL

2203A Commerce Road, Suite 1
Forest Hill, MD 21050 USA
1.410.838.8780

CERTIFICATE OF ANALYSIS

**University of Notre Dame**	**Site Name:** So-Cal Military Toxic Site	**Beacon Proposal:** 201201H01
Department of Biological Sciences	**Site Location:** Notspa, CA	**Lab Work Order:** 0005542
South Bend, IN 46556	**Project Manager:** Kristin Shrader-Frechette	**Reported:** 01/25/2021

Table 2 - Form II A VOA
Volatile Deuterated Monitoring Compound Recovery Summary

Method: TO-17 (Passive)

Sequence: B21A005

Instrument: K System

QC Limits: 70 - 130%

+ values are outside method/contract required QC limits

			% Recovery		
**Lab Number**	**Client Sample Name**	**File ID**	**Toluene-d8**	**BFB**	**1,2-DCA-d4**
21A0006-BS1	LCS, Primary Calibration Source	K21010502.D	102	91	109
21A0006-BLK1	Method Blank	K21010503.D	101	90	101
B21A005-ICV1	LCSD, Second Source	K21010504.D	96	89	100
0005542-01	A	K21010505.D	98	92	103
0005542-02	B	K21010506.D	96	95	101
0005542-03	C	K21010507.D	96	93	100
0005542-05	E	K21010509.D	97	97	96
0005542-06	F	K21010510.D	97	92	103
0005542-07	G	K21010511.D	96	94	100
0005542-09	I	K21010513.D	95	95	98
0005542-10	J	K21010514.D	96	96	89
0005542-11	J-DUP	K21010515.D	92	95	92
0005542-12	K	K21010516.D	92	93	92
B21A005-CCV1	Closing Calibration Verification	K21010517.D	99	96	100

BEACON ENVIRONMENTAL
2203A Commerce Road, Suite 1
Forest Hill, MD 21050 USA
1.410.838.8780

CERTIFICATE OF ANALYSIS

**University of Notre Dame**	**Site Name:** So-Cal Military Toxic Site	**Beacon Proposal:** 201201H01
Department of Biological Sciences	**Site Location:** Notspa, CA	**Lab Work Order:** 0005542
South Bend, IN 46556	**Project Manager:** Kristin Shrader-Frechette	**Reported:** 01/25/2021

Table 2 - Form II A VOA
Volatile Deuterated Monitoring Compound Recovery Summary

Method: TO-17 (Passive) **Instrument:** K System

Sequence: B21A006 **QC Limits:** 70 - 130%

+ values are outside method/contract required QC limits

			% Recovery		
**Lab Number**	**Client Sample Name**	**File ID**	**Toluene-d8**	**BFB**	**1,2-DCA-d4**
21A0007-BS1	LCS, Primary Calibration Source	K21010602.D	100	92	108
21A0007-BLK1	Method Blank	K21010603.D	99	90	99
B21A006-ICV1	LCSD, Second Source	K21010604.D	100	93	105
0005542-04	D	K21010605.D	95	91	103
0005542-08	H	K21010606.D	100	96	102
B21A006-CCV1	Closing Calibration Verification	K21010607.D	101	92	100

2203A Commerce Road, Suite 1
Forest Hill, MD 21050 USA
1.410.838.8780

BEACON ENVIRONMENTAL

CERTIFICATE OF ANALYSIS

**University of Notre Dame**	**Site Name:** So-Cal Military Toxic Site	**Beacon Proposal:** 201201H01
Department of Biological Sciences	**Site Location:** Notspa, CA	**Lab Work Order:** 0005542
South Bend, IN 46556	**Project Manager:** Kristin Shrader-Frechette	**Reported:** 01/25/2021

Table 3 - Form III B VOA
Volatile Laboratory Control Sample Recoveries

Lab Sample No.: 21A0006-BS1 | **QC Description:** LCS

Instrument: K System | **Sequence:** B21A005

Method: TO-17 (Passive) | **File ID:** K21010502.D

+ values are outside method/contract required QC limits

Compound	Spike Added (ng)	Spike Result (ng)	% Recovery	Q	QC Limits	Notes
Dichlorodifluoromethane (Freon 12)	50.0	50.5	101.0		70 - 130	
Chloroform	50.0	50.8	101.5		70 - 130	
Carbon Tetrachloride	50.0	47.5	95.1		70 - 130	
Dibromomethane	50.0	53.8	107.7		70 - 130	
Trichloroethene	50.0	51.7	103.3		70 - 130	
Tetrachloroethene	50.0	52.2	104.3		70 - 130	

BEACON ENVIRONMENTAL

2203A Commerce Road, Suite 1
Forest Hill, MD 21050 USA
1.410.838.8780

CERTIFICATE OF ANALYSIS

**University of Notre Dame**	**Site Name:** So-Cal Military Toxic Site	**Beacon Proposal:** 201201H01
Department of Biological Sciences	**Site Location:** Notspa, CA	**Lab Work Order:** 0005542
South Bend, IN 46556	**Project Manager:** Kristin Shrader-Frechette	**Reported:** 01/25/2021

Table 3 - Form III B VOA
Volatile Laboratory Control Sample Recoveries

Lab Sample No.: 21A0007-BS1 **QC Description:** LCS

Instrument: K System **Sequence:** B21A006

Method: TO-17 (Passive) **File ID:** K21010602.D

+ values are outside method/contract required QC limits

Compound	Spike Added (ng)	Spike Result (ng)	% Recovery	Q	QC Limits	Notes
Dichlorodifluoromethane (Freon 12)	50.0	42.1	84.1		70 - 130	
Chloroform	50.0	50.7	101.4		70 - 130	
Carbon Tetrachloride	50.0	47.5	94.9		70 - 130	
Dibromomethane	50.0	52.6	105.3		70 - 130	
Trichloroethene	50.0	51.4	102.8		70 - 130	
Tetrachloroethene	50.0	50.9	101.8		70 - 130	

BEACON ENVIRONMENTAL

2203A Commerce Road, Suite 1
Forest Hill, MD 21050 USA
1.410.838.8780

CERTIFICATE OF ANALYSIS

**University of Notre Dame**	**Site Name:** So-Cal Military Toxic Site	**Beacon Proposal:** 201201H01
Department of Biological Sciences	**Site Location:** Notspa, CA	**Lab Work Order:** 0005542
South Bend, IN 46556	**Project Manager:** Kristin Shrader-Frechette	**Reported:** 01/25/2021

Table 3 - Form III B VOA
Volatile LCS/LCSD Recovery/RPD

Lab Sample No.: B21A005-ICV1
QC Description: LCSD, Second Source Standard
Method: TO-17 (Passive)
Sequence: B21A005
Batch: B21A005
Instrument: K System
LCSD FileID: K21010504.D
LCS FileID: K21010502.D

+ values are outside method/contract required QC limits

Compound	Spike Added (ng)	LCS Result (ng)	LCSD Result (ng)	LCSD Recovery (%)	LCSD RPD (%)	RPD Limit (%)	LCSD Recovery Limits (%)
Dichlorodifluoromethane (Freon 12)	50	50.51	57.73	115.00	13.34	30	70 - 130
Chloroform	50	50.75	47.51	95.00	6.59	30	70 - 130
Carbon Tetrachloride	50	47.54	46.57	93.10	2.06	30	70 - 130
Dibromomethane	50	53.83	54.42	109.00	1.09	30	70 - 130
Trichloroethene	50	51.67	50.92	102.00	1.46	30	70 - 130
Tetrachloroethene	50	52.16	49.62	99.20	4.99	30	70 - 130

BEACON ENVIRONMENTAL

2203A Commerce Road, Suite 1
Forest Hill, MD 21050 USA
1.410.838.8780

CERTIFICATE OF ANALYSIS

**University of Notre Dame**	**Site Name:** So-Cal Military Toxic Site	**Beacon Proposal:** 201201H01
Department of Biological Sciences	**Site Location:** Notspa, CA	**Lab Work Order:** 0005542
South Bend, IN 46556	**Project Manager:** Kristin Shrader-Frechette	**Reported:** 01/25/2021

Table 3 - Form III B VOA
Volatile LCS/LCSD Recovery/RPD

Lab Sample No.: B21A006-ICV1 **QC Description:** LCSD, Second Source Standard
Method: TO-17 (Passive) **Sequence:** B21A006
Batch: B21A006 **Instrument:** K System
LCSD FileID: K21010604.D **LCS FileID:** K21010602.D

+ values are outside method/contract required QC limits

Compound	Spike Added (ng)	LCS Result (ng)	LCSD Result (ng)	LCSD Recovery (%)	LCSD RPD (%)	RPD Limit (%)	LCSD Recovery Limits (%)
Dichlorodifluoromethane (Freon 12)	50	42.06	43.67	87.30	3.76	30	70 - 130
Chloroform	50	50.72	51.46	103.00	1.45	30	70 - 130
Carbon Tetrachloride	50	47.46	48.01	96.00	1.15	30	70 - 130
Dibromomethane	50	52.63	52.91	106.00	0.53	30	70 - 130
Trichloroethene	50	51.38	52.18	104.00	1.54	30	70 - 130
Tetrachloroethene	50	50.91	50.45	101.00	0.91	30	70 - 130

2203A Commerce Road, Suite 1
Forest Hill, MD 21050 USA
1.410.838.8780

BEACON ENVIRONMENTAL

CERTIFICATE OF ANALYSIS

**University of Notre Dame**	**Site Name:** So-Cal Military Toxic Site	**Beacon Proposal:** 201201H01
Department of Biological Sciences	**Site Location:** Notspa, CA	**Lab Work Order:** 0005542
South Bend, IN 46556	**Project Manager:** Kristin Shrader-Frechette	**Reported:** 01/25/2021

Table 3 - Form III C VOA

Volatile Laboratory Sample Duplicate Data Sheet

+ values are outside method/contract required QC limits

	Field Duplicate	Initial Sample			
**Lab Number:**	0005542-11	0005542-10			
**Sample Name:**	J-DUP	J			
**Analyte**	**Result (µg/m³)**	**Result (µg/m³)**	**RPD**	**RPD Limit**	**Q**
Dichlorodifluoromethane (Freon 12)	0.428 U	0.428 U		25	
Chloroform	0.709 U	0.709 U		25	
Carbon Tetrachloride	0.577 U	0.577 U		25	
Dibromomethane	0.620 U	0.620 U		25	
Trichloroethene	0.752 U	0.752 U		25	
Tetrachloroethene	1.63	1.50	8.3	25	

2203A Commerce Road, Suite 1
Forest Hill, MD 21050 USA
1.410.838.8780

BEACON ENVIRONMENTAL

CERTIFICATE OF ANALYSIS

**University of Notre Dame**	**Site Name:** So-Cal Military Toxic Site	**Beacon Proposal:** 201201H01
Department of Biological Sciences	**Site Location:** Notspa, CA	**Lab Work Order:** 0005542
South Bend, IN 46556	**Project Manager:** Kristin Shrader-Frechette	**Reported:** 01/25/2021

Table 4 - Form IV VOA

Volatile Method Blank Summary

Sequence: B20L066 **Batch:** B20L066 **Matrix:** **Analysis:**

EPA Sample No.: B20L066-ICB1 **Instrument:** K System **Date Analyzed**: 12/23/2020

Sample Name	Lab Sample Number	Lab File ID	Time Analyzed
MS Tune	B20L066-TUN1	Kd20122301.D	17:56:00
Cal Standard	B20L066-CAL1	Kd20122303.D	18:48:00
Cal Standard	B20L066-CAL2	Kd20122304.D	19:14:00
Cal Standard	B20L066-CAL3	Kd20122305.D	19:39:00
Cal Standard	B20L066-CAL4	Kd20122306.D	20:05:00
Cal Standard	B20L066-CAL5	Kd20122307.D	20:31:00
Cal Standard	B20L066-CAL6	Kd20122308.D	20:57:00
Cal Standard	B20L066-CAL7	Kd20122309.D	21:23:00
Cal Standard	B20L066-CAL8	Kd20122310.D	21:48:00
Cal Standard	B20L066-CAL9	Kd20122311.D	22:15:00
Cal Standard	B20L066-CALA	Kd20122312.D	22:40:00
Cal Standard	B20L066-CALB	Kd20122313.D	23:06:00
LCSD/Second Source Verification/CALV	B20L066-ICV1	Kd20122316.D	0:23:00
Lab Blank/Initial Calibration Blank	B20L066-ICB1	Kd20122318.D	1:14:00

BEACON ENVIRONMENTAL

2203A Commerce Road, Suite 1
Forest Hill, MD 21050 USA
1.410.838.8780

CERTIFICATE OF ANALYSIS

**University of Notre Dame**	**Site Name:** So-Cal Military Toxic Site	**Beacon Proposal:** 201201H01
Department of Biological Sciences	**Site Location:** Notspa, CA	**Lab Work Order:** 0005542
South Bend, IN 46556	**Project Manager:** Kristin Shrader-Frechette	**Reported:** 01/25/2021

Table 4 - Form IV VOA

Volatile Method Blank Summary

Sequence: B21A005 **Batch:** B21A005 **Matrix:** Indoor Air **Analysis:** TO-17 (Passive)

EPA Sample No.: 21A0006-BLK1 **Instrument:** K System **Date Analyzed:** 01/05/2021

Sample Name	Lab Sample Number	Lab File ID	Time Analyzed
MS Tune	B21A005-TUN1	K21010501.D	13:50:00
LCS, Calibration Source Verification	21A0006-BS1	K21010502.D	14:16:00
Lab Blank	21A0006-BLK1	K21010503.D	14:42:00
LCSD/Second Source Verification/CALV	B21A005-ICV1	K21010504.D	15:08:00
A	0005542-01	K21010505.D	15:38:00
B	0005542-02	K21010506.D	16:07:00
C	0005542-03	K21010507.D	16:37:00
E	0005542-05	K21010509.D	17:36:00
F	0005542-06	K21010510.D	18:06:00
G	0005542-07	K21010511.D	18:36:00
I	0005542-09	K21010513.D	19:37:00
J	0005542-10	K21010514.D	20:06:00
J-DUP	0005542-11	K21010515.D	20:37:00
K	0005542-12	K21010516.D	21:06:00
LCS, Closing Calibration Verification	B21A005-CCV1	K21010517.D	21:32:00

BEACON ENVIRONMENTAL

2203A Commerce Road, Suite 1
Forest Hill, MD 21050 USA
1.410.838.8780

CERTIFICATE OF ANALYSIS

**University of Notre Dame**	**Site Name:** So-Cal Military Toxic Site	**Beacon Proposal:** 201201H01
Department of Biological Sciences	**Site Location:** Notspa, CA	**Lab Work Order:** 0005542
South Bend, IN 46556	**Project Manager:** Kristin Shrader-Frechette	**Reported:** 01/25/2021

Table 4 - Form IV VOA

Volatile Method Blank Summary

Sequence: B21A006 **Batch:** B21A006 **Matrix:** Indoor Air **Analysis:** TO-17 (Passive)

EPA Sample No.: 21A0007-BLK1 **Instrument:** K System **Date Analyzed:** 01/06/2021

Sample Name	Lab Sample Number	Lab File ID	Time Analyzed
MS Tune	B21A006-TUN1	K21010601.D	7:48:00
LCS, Calibration Source Verification	21A0007-BS1	K21010602.D	8:13:00
Lab Blank	21A0007-BLK1	K21010603.D	8:39:00
LCSD/Second Source Verification/CALV	B21A006-ICV1	K21010604.D	9:05:00
D	0005542-04	K21010605.D	9:35:00
H	0005542-08	K21010606.D	10:05:00
LCS, Closing Calibration Verification	B21A006-CCV1	K21010607.D	10:30:00

BEACON ENVIRONMENTAL

2203A Commerce Road, Suite 1
Forest Hill, MD 21050 USA
1.410.838.8780

CERTIFICATE OF ANALYSIS

**University of Notre Dame**	**Site Name:** So-Cal Military Toxic Site	**Beacon Proposal:** 201201H01
Department of Biological Sciences	**Site Location:** Notspa, CA	**Lab Work Order:** 0005542
South Bend, IN 46556	**Project Manager:** Kristin Shrader-Frechette	**Reported:** 01/25/2021

Table 5 - Form V VOA

Volatile Organic Instrument Performance Check (BFB)

TO-17 (Passive)

Laboratory:	Beacon Environmental	SDG:	
Client:	University of Notre Dame	Project Site:	Notspa, CA
Lab File ID:	Kd20122301.D	Injection Date:	12/23/20
Instrument ID:	K System	Injection Time:	17:56
Sequence:	B20L066	Lab Sample ID:	B20L066-TUN1

m/z	ION ABUNDANCE CRITERIA	% RELATIVE ABUNDANCE	
50	8 - 40% of 95	11.7	PASS
75	30 - 66% of 95	41.0	PASS
95	Base peak, 100% relative abundance	100.0	PASS
96	5 - 9% of 95	6.8	PASS
173	Less than 2% of 174	0.2	PASS
174	50 - 120% of 95	90.7	PASS
175	4 - 9% of 174	7.4	PASS
176	93 - 101% of 174	98.0	PASS
177	5 - 9% of 176	6.3	PASS

Data Path : Z:\GCMS\data\20\12\K201223d\
Data File : Kd20122301.D
Acq On : 23 Dec 2020 5:56 pm
Operator : PBK
Sample : SEQ-TUN1
Misc : Dual Bed, BFB
ALS Vial : 31 Sample Multiplier: 1

Integration File: ALI_OOP.P

Method : Z:\msdchem\K_system\5975K_201223_Dual_Bed_TO17_8260C_High.M
Title : SOURCE AREA VOA ANALYSIS
Last Update : Wed Dec 23 11:21:43 2020

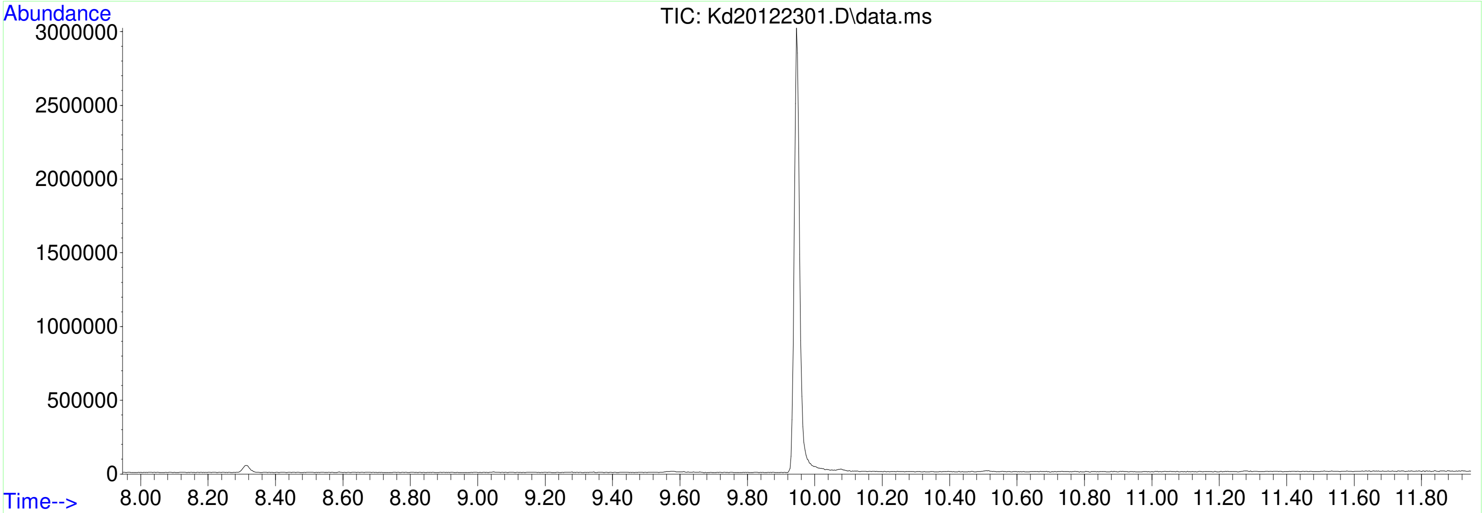

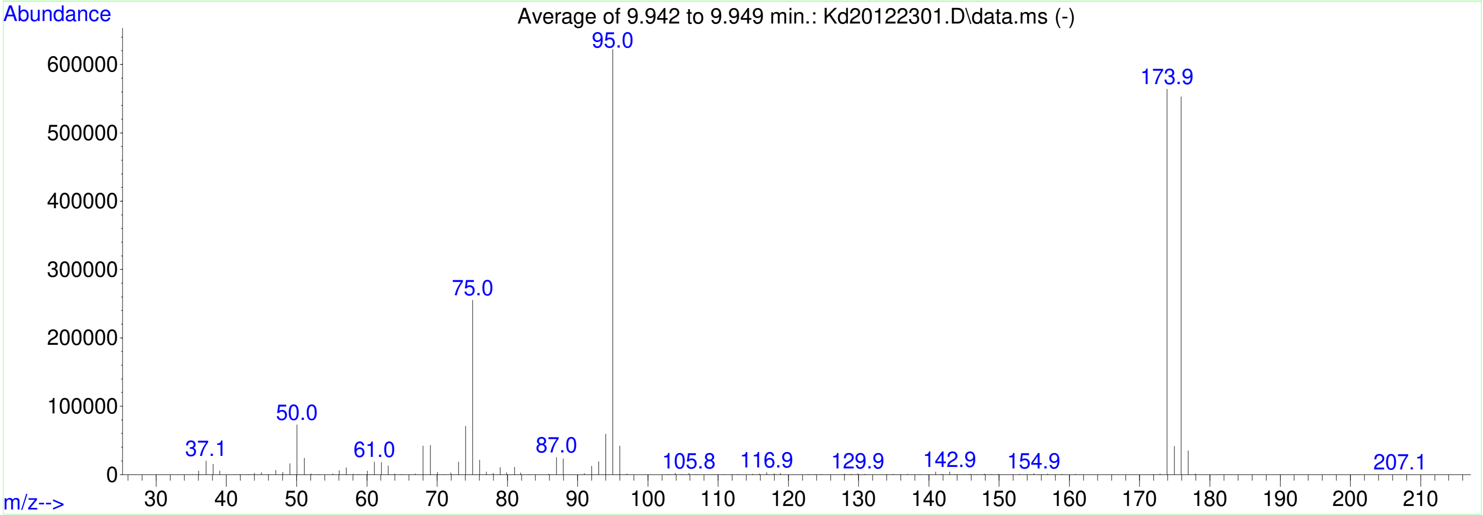

AutoFind: Scans 3086, 3087, 3088; Background Corrected with Scan 3077

Target Mass	Rel. to Mass	Lower Limit%	Upper Limit%	Rel. Abn%	Raw Abn	Result Pass/Fail
50	95	8	40	11.7	72992	PASS
75	95	30	66	41.0	255275	PASS
95	95	100	100	100.0	622144	PASS
96	95	5	9	6.8	42085	PASS
173	174	0.00	2	0.2	1355	PASS
174	95	50	120	90.7	563989	PASS
175	174	4	9	7.4	41531	PASS
176	174	93	101	98.0	552533	PASS
177	176	5	9	6.3	34941	PASS

BEACON ENVIRONMENTAL

2203A Commerce Road, Suite 1
Forest Hill, MD 21050 USA
1.410.838.8780

CERTIFICATE OF ANALYSIS

**University of Notre Dame**	**Site Name:** So-Cal Military Toxic Site	**Beacon Proposal:** 201201H01
Department of Biological Sciences	**Site Location:** Notspa, CA	**Lab Work Order:** 0005542
South Bend, IN 46556	**Project Manager:** Kristin Shrader-Frechette	**Reported:** 01/25/2021

Table 5 - Form V VOA

Volatile Organic Instrument Performance Check (BFB)

TO-17 (Passive)

Laboratory:	Beacon Environmental	SDG:	
Client:	University of Notre Dame	Project Site:	Notspa, CA
Lab File ID:	K21010501.D	Injection Date:	01/05/21
Instrument ID:	K System	Injection Time:	13:50
Sequence:	B21A005	Lab Sample ID:	B21A005-TUN1

m/z	ION ABUNDANCE CRITERIA	% RELATIVE ABUNDANCE	
50	8 - 40% of 95	11.8	PASS
75	30 - 66% of 95	41.5	PASS
95	Base peak, 100% relative abundance	100.0	PASS
96	5 - 9% of 95	6.6	PASS
173	Less than 2% of 174	0.5	PASS
174	50 - 120% of 95	92.8	PASS
175	4 - 9% of 174	7.0	PASS
176	93 - 101% of 174	95.3	PASS
177	5 - 9% of 176	6.5	PASS

Data Path : Z:\GCMS\data\21\01\K210105\
Data File : K21010501.D
Acq On : 5 Jan 2021 1:50 pm
Operator : sct
Sample : B21A005-TUN1
Misc : Dual Bed, BFB
ALS Vial : 61 Sample Multiplier: 1

Integration File: ALI_OOP.P

Method : Z:\msdchem\K_system\5975K_201223_Dual_Bed_TO17_8260C_High.M
Title : SOURCE AREA VOA ANALYSIS
Last Update : Thu Dec 24 06:58:47 2020

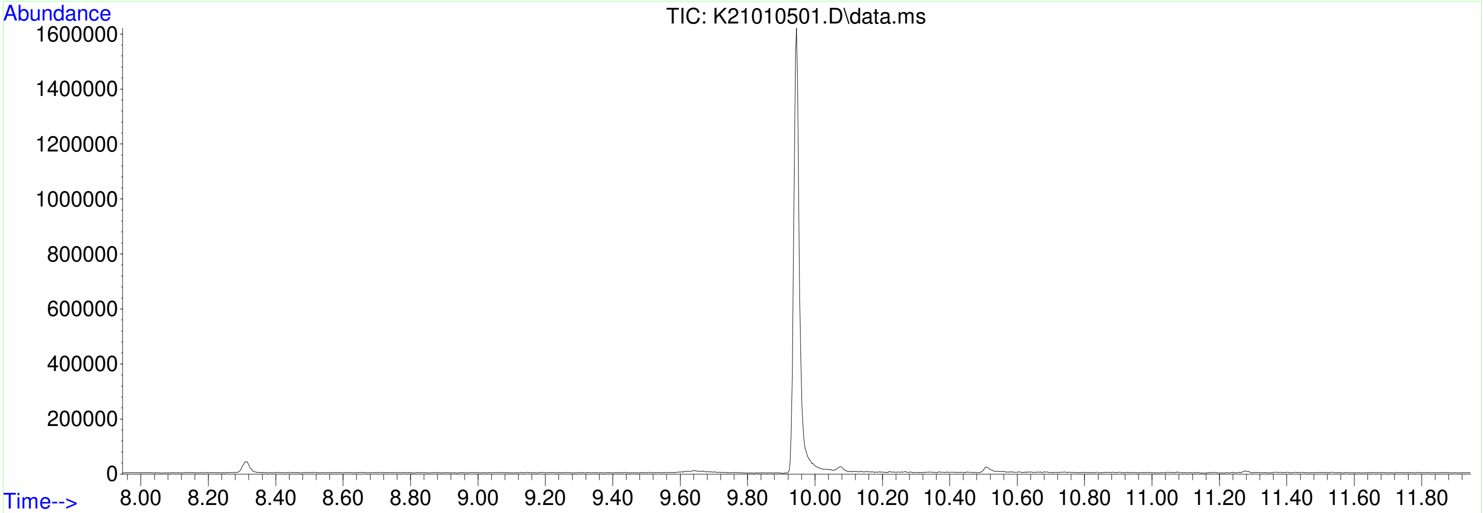

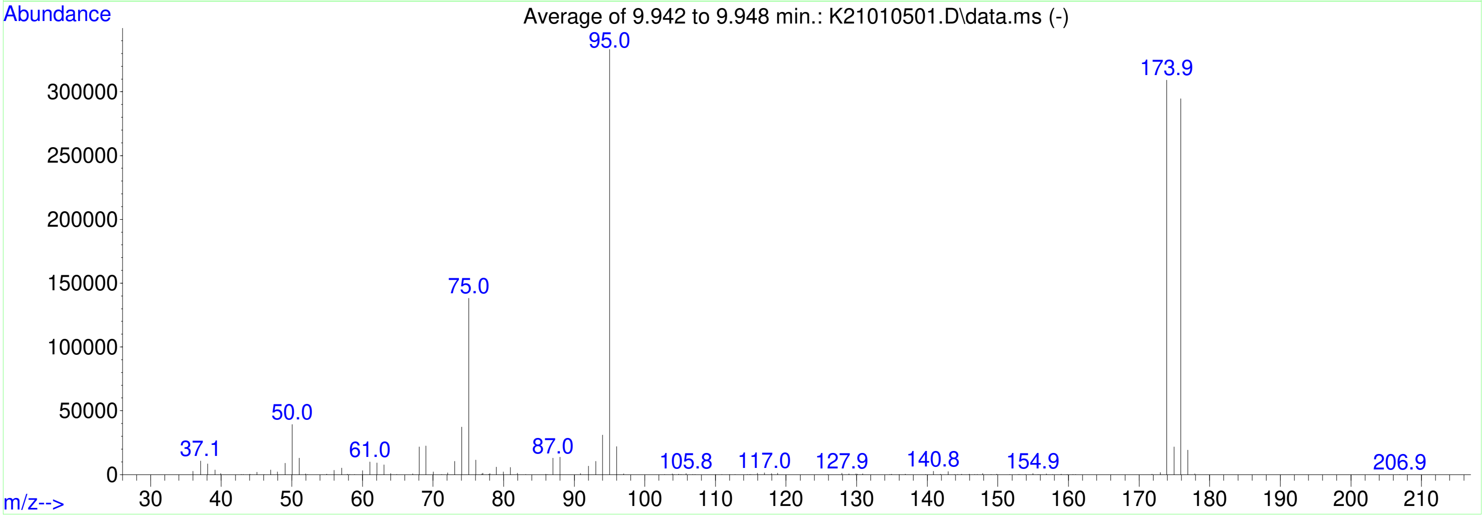

AutoFind: Scans 3086, 3087, 3088; Background Corrected with Scan 3076

Target Mass	Rel. to Mass	Lower Limit%	Upper Limit%	Rel. Abn%	Raw Abn	Result Pass/Fail
50	95	8	40	11.8	39221	PASS
75	95	30	66	41.5	138253	PASS
95	95	100	100	100.0	333248	PASS
96	95	5	9	6.6	21944	PASS
173	174	0.00	2	0.5	1532	PASS
174	95	50	120	92.8	309205	PASS
175	174	4	9	7.0	21789	PASS
176	174	93	101	95.3	294613	PASS
177	176	5	9	6.5	19288	PASS

BEACON ENVIRONMENTAL

2203A Commerce Road, Suite 1
Forest Hill, MD 21050 USA
1.410.838.8780

CERTIFICATE OF ANALYSIS

**University of Notre Dame**	**Site Name:** So-Cal Military Toxic Site	**Beacon Proposal:** 201201H01
Department of Biological Sciences	**Site Location:** Notspa, CA	**Lab Work Order:** 0005542
South Bend, IN 46556	**Project Manager:** Kristin Shrader-Frechette	**Reported:** 01/25/2021

Table 5 - Form V VOA

Volatile Organic Instrument Performance Check (BFB)

TO-17 (Passive)

Laboratory:	Beacon Environmental	SDG:	
Client:	University of Notre Dame	Project Site:	Notspa, CA
Lab File ID:	K21010601.D	Injection Date:	01/06/21
Instrument ID:	K System	Injection Time:	07:48
Sequence:	B21A006	Lab Sample ID:	B21A006-TUN1

m/z	ION ABUNDANCE CRITERIA	% RELATIVE ABUNDANCE	
50	8 - 40% of 95	12.4	PASS
75	30 - 66% of 95	41.3	PASS
95	Base peak, 100% relative abundance	100.0	PASS
96	5 - 9% of 95	6.5	PASS
173	Less than 2% of 174	0.7	PASS
174	50 - 120% of 95	91.0	PASS
175	4 - 9% of 174	7.4	PASS
176	93 - 101% of 174	94.3	PASS
177	5 - 9% of 176	6.5	PASS

```
Data Path : Z:\GCMS\data\21\01\K210106\
Data File : K21010601.D
Acq On    :  6 Jan 2021   7:48 am
Operator  : sct
Sample    : B21A006-TUN1
Misc      : Dual Bed, BFB
ALS Vial  : 1   Sample Multiplier: 1

Integration File: ALI_OOP.P

Method    : Z:\msdchem\K_system\5975K_201223_Dual_Bed_TO17_8260C_High.M
Title     : SOURCE AREA  VOA ANALYSIS
Last Update  : Thu Dec 24 06:58:47 2020
```

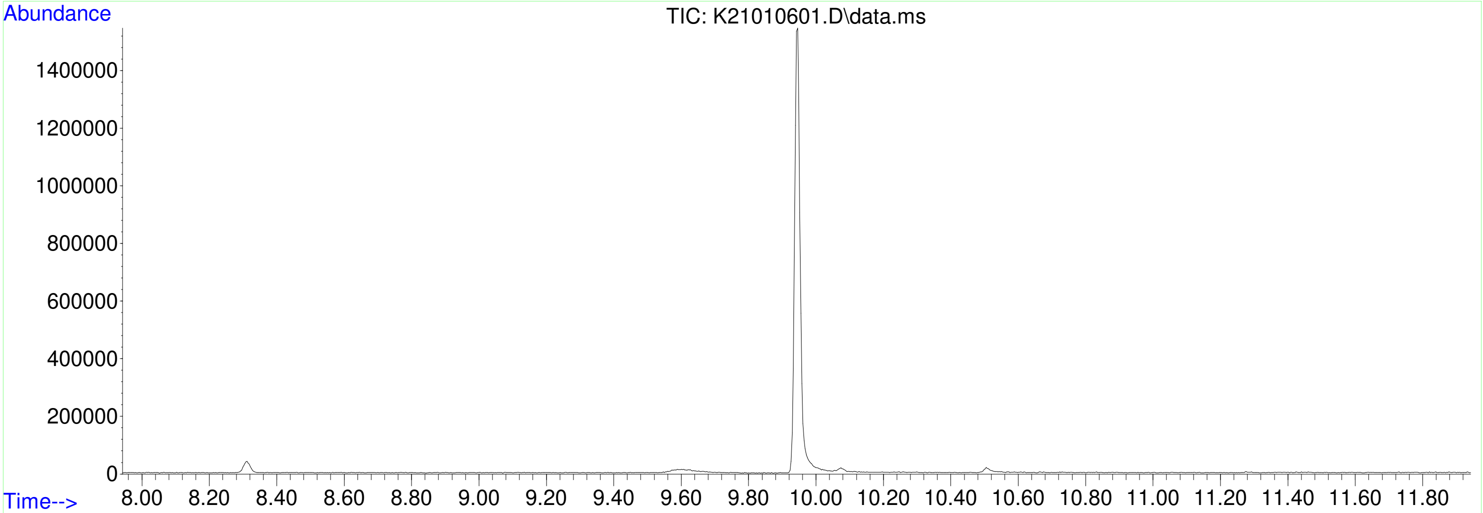

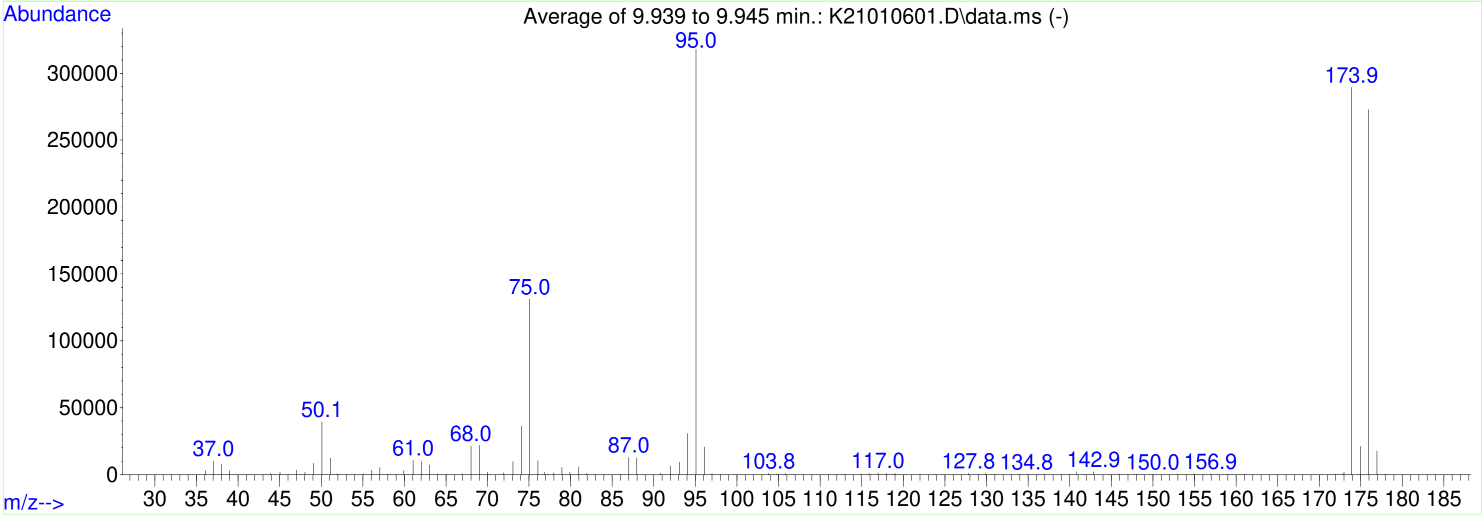

AutoFind: Scans 3085, 3086, 3087; Background Corrected with Scan 3077

Target Mass	Rel. to Mass	Lower Limit%	Upper Limit%	Rel. Abn%	Raw Abn	Result Pass/Fail
50	95	8	40	12.4	39339	PASS
75	95	30	66	41.3	131400	PASS
95	95	100	100	100.0	317888	PASS
96	95	5	9	6.5	20760	PASS
173	174	0.00	2	0.7	1967	PASS
174	95	50	120	91.0	289323	PASS
175	174	4	9	7.4	21307	PASS
176	174	93	101	94.3	272832	PASS
177	176	5	9	6.5	17601	PASS

BEACON ENVIRONMENTAL

2203A Commerce Road, Suite 1
Forest Hill, MD 21050 USA
1.410.838.8780

CERTIFICATE OF ANALYSIS

**University of Notre Dame**	**Site Name:** So-Cal Military Toxic Site	**Beacon Proposal:** 201201H01
Department of Biological Sciences	**Site Location:** Notspa, CA	**Lab Work Order:** 0005542
South Bend, IN 46556	**Project Manager:** Kristin Shrader-Frechette	**Reported:** 01/25/2021

Table 5 - Form IV VOA
Volatile Organic Instrument Performance Check (BFB) Sequence Summary

Sequence: B20L066 **Batch:** B20L066 **Matrix:** Indoor Air

EPA Sample No.: B20L066-TUN1 **Instrument:** K System **Date Analyzed:** 12/23/2020

+ values are outside method/contract required QC limits

	Lab Sample Number	Lab File ID	Time Analyzed	Tune to Analysis (hr)	Q
B20L066-TUN1	B20L066-TUN1	Kd20122301.D	17:56:00	0.00	
B20L066-CAL1	B20L066-CAL1	Kd20122303.D	18:48:00	0.87	
B20L066-CAL2	B20L066-CAL2	Kd20122304.D	19:14:00	1.30	
B20L066-CAL3	B20L066-CAL3	Kd20122305.D	19:39:00	1.72	
B20L066-CAL4	B20L066-CAL4	Kd20122306.D	20:05:00	2.15	
B20L066-CAL5	B20L066-CAL5	Kd20122307.D	20:31:00	2.58	
B20L066-CAL6	B20L066-CAL6	Kd20122308.D	20:57:00	3.02	
B20L066-CAL7	B20L066-CAL7	Kd20122309.D	21:23:00	3.45	
B20L066-CAL8	B20L066-CAL8	Kd20122310.D	21:48:00	3.87	
B20L066-CAL9	B20L066-CAL9	Kd20122311.D	22:15:00	4.32	
B20L066-CALA	B20L066-CALA	Kd20122312.D	22:40:00	4.73	
B20L066-CALB	B20L066-CALB	Kd20122313.D	23:06:00	5.17	
LCSD, Calibration Verification, Second Source	B20L066-ICV1	Kd20122316.D	0:23:00	6.45	
Lab Blank	B20L066-ICB1	Kd20122318.D	1:14:00	7.30	

2203A Commerce Road, Suite 1
Forest Hill, MD 21050 USA
1.410.838.8780

BEACON ENVIRONMENTAL

CERTIFICATE OF ANALYSIS

**University of Notre Dame**	**Site Name:** So-Cal Military Toxic Site	**Beacon Proposal:** 201201H01
Department of Biological Sciences	**Site Location:** Notspa, CA	**Lab Work Order:** 0005542
South Bend, IN 46556	**Project Manager:** Kristin Shrader-Frechette	**Reported:** 01/25/2021

Table 5 - Form IV VOA
Volatile Organic Instrument Performance Check (BFB) Sequence Summary

Sequence: B21A005 **Batch:** B21A005 **Matrix:** Indoor Air

EPA Sample No.: B21A005-TUN1 **Instrument:** K System **Date Analyzed**: 01/05/2021

+ values are outside method/contract required QC limits

	Lab Sample Number	Lab File ID	Time Analyzed	Tune to Analysis (hr)	Q
B21A005-TUN1	B21A005-TUN1	K21010501.D	13:50:00	0.00	
LCS, Primary Calibration Source	21A0006-BS1	K21010502.D	14:16:00	0.43	
Method Blank	21A0006-BLK1	K21010503.D	14:42:00	0.87	
LCSD, Calibration Verification, Second Source	B21A005-ICV1	K21010504.D	15:08:00	1.30	
0005542-01	0005542-01	K21010505.D	15:38:00	1.80	
0005542-02	0005542-02	K21010506.D	16:07:00	2.28	
0005542-03	0005542-03	K21010507.D	16:37:00	2.78	
0005542-05	0005542-05	K21010509.D	17:36:00	3.77	
0005542-06	0005542-06	K21010510.D	18:06:00	4.27	
0005542-07	0005542-07	K21010511.D	18:36:00	4.77	
0005542-09	0005542-09	K21010513.D	19:37:00	5.78	
0005542-10	0005542-10	K21010514.D	20:06:00	6.27	
0005542-11	0005542-11	K21010515.D	20:37:00	6.78	
0005542-12	0005542-12	K21010516.D	21:06:00	7.27	
Closing Calibration Verification	B21A005-CCV1	K21010517.D	21:32:00	7.70	

BEACON ENVIRONMENTAL
2203A Commerce Road, Suite 1
Forest Hill, MD 21050 USA
1.410.838.8780

CERTIFICATE OF ANALYSIS

**University of Notre Dame**	**Site Name:** So-Cal Military Toxic Site	**Beacon Proposal:** 201201H01
Department of Biological Sciences	**Site Location:** Notspa, CA	**Lab Work Order:** 0005542
South Bend, IN 46556	**Project Manager:** Kristin Shrader-Frechette	**Reported:** 01/25/2021

Table 5 - Form IV VOA
Volatile Organic Instrument Performance Check (BFB) Sequence Summary

Sequence: B21A006 **Batch:** B21A006 **Matrix:** Indoor Air

EPA Sample No.: B21A006-TUN1 **Instrument:** K System **Date Analyzed**: 01/06/2021

+ values are outside method/contract required QC limits

	Lab Sample Number	Lab File ID	Time Analyzed	Tune to Analysis (hr)	Q
B21A006-TUN1	B21A006-TUN1	K21010601.D	7:48:00	0.00	
LCS, Primary Calibration Source	21A0007-BS1	K21010602.D	8:13:00	0.42	
Method Blank	21A0007-BLK1	K21010603.D	8:39:00	0.85	
LCSD, Calibration Verification, Second Source	B21A006-ICV1	K21010604.D	9:05:00	1.28	
0005542-04	0005542-04	K21010605.D	9:35:00	1.78	
0005542-08	0005542-08	K21010606.D	10:05:00	2.28	
Closing Calibration Verification	B21A006-CCV1	K21010607.D	10:30:00	2.70	

2203A Commerce Road, Suite 1
Forest Hill, MD 21050 USA
1.410.838.8780

BEACON ENVIRONMENTAL

CERTIFICATE OF ANALYSIS

**University of Notre Dame**	**Site Name:** So-Cal Military Toxic Site	**Beacon Proposal:** 201201H01
Department of Biological Sciences	**Site Location:** Notspa, CA	**Lab Work Order:** 0005542
South Bend, IN 46556	**Project Manager:** Kristin Shrader-Frechette	**Reported:** 01/25/2021

Table 6 - Form VI A VOA
Volatile Organics Initial Calibration Data

Calibration: **BL00006** Calibration Start: 12/23/2020 6:48:00PM

Instrument: K System Calibration End: 12/23/2020 9:23:00PM

**Lab File ID:**	Kd20122303.D	Kd20122304.D	Kd20122305.D	Kd20122306.D	Kd20122307.D	Kd20122308.D	Kd20122309.D	
	Response Factors							
**Analyte**	**2.5 ng**	**5 ng**	**10 ng**	**25 ng**	**50 ng**	**100 ng**	**200 ng**	
Dichlorodifluoromethane (Freon 12)	0.28	0.22	0.22	0.13	0.28	0.07	0.16	
Chloroform	0.35	0.31	0.31	0.31	0.30	0.30	0.30	
1,2-DCA-d4	0.30	0.28	0.26	0.26	0.27	0.26	0.25	
Carbon Tetrachloride	0.25	0.32	0.31	0.29	0.29	0.29	0.29	
Dibromomethane	0.15	0.17	0.17	0.17	0.17	0.17	0.17	
Trichloroethene	0.27	0.28	0.30	0.27	0.27	0.28	0.28	
Toluene-d8	1.55	1.41	1.42	1.37	1.38	1.40	1.38	
Tetrachloroethene	0.55	0.59	0.54	0.53	0.51	0.51	0.52	
Bromofluorobenzene	0.65	0.56	0.50	0.47	0.46	0.45	0.48	

2203A Commerce Road, Suite 1
Forest Hill, MD 21050 USA
1.410.838.8780

CERTIFICATE OF ANALYSIS

**University of Notre Dame**	**Site Name:**	So-Cal Military Toxic Site	**Beacon Proposal:**	201201H01
Department of Biological Sciences	**Site Location:**	Notspa, CA	**Lab Work Order:**	0005542
South Bend, IN 46556	**Project Manager:**	Kristin Shrader-Frechette	**Reported:**	01/25/2021

Table 6 - Form VI A VOA
Volatile Organics Initial Calibration Data

Calibration: BL00006 **Calibration Start:** 12/23/2020 6:48:00PM

Instrument: K System **Calibration End:** 12/23/2020 9:23:00PM

+ values are outside method/contract required QC limits

**Lab File ID:**	Kd20122310.D	Kd20122311.D	Kd20122312.D	Kd20122313.D	Acceptance Limits	
	Response Factors				≤ 20	≥ 0.99
**Analyte**	**ng**	**ng**	**ng**	**ng**	**RSD**	R^2
Dichlorodifluoromethane (Freon 12)	--	--	--	--	+ 39.20	
Chloroform	0.28	0.29	0.28	0.29	6.56	
1,2-DCA-d4	0.25	0.26	0.25	0.26	5.15	
Carbon Tetrachloride	0.28	0.29	0.28	0.30	6.37	
Dibromomethane	--	--	--	--	5.96	
Trichloroethene	0.26	0.27	0.27	0.27	3.47	
Toluene-d8	1.37	1.37	1.39	1.37	3.76	
Tetrachloroethene	0.48	0.48	0.48	0.48	6.90	
Bromofluorobenzene	0.47	0.48	0.48	0.48	11.91	

2203A Commerce Road, Suite 1
Forest Hill, MD 21050 USA
1.410.838.8780

BEACON ENVIRONMENTAL

CERTIFICATE OF ANALYSIS

**University of Notre Dame**	**Site Name:** So-Cal Military Toxic Site	**Beacon Proposal:** 201201H01
Department of Biological Sciences	**Site Location:** Notspa, CA	**Lab Work Order:** 0005542
South Bend, IN 46556	**Project Manager:** Kristin Shrader-Frechette	**Reported:** 01/25/2021

Table 7 - Form VII A VOA
Volatile Organics Continuing Calibration Data: Calibration Mid-Point

Sample No.: 50ng Cal Std (B20L066-CAL5) **Calibration:** BL00006 **Calibration Start Date:** 12/23/2020 6:48:00PM

Sequence: B20L066 **Instrument:** K System **Lab File ID:** K21010605.D **Calibration End Date:** 12/23/2020 9:23:00PM

+ values are outside method/contract required QC limits

Compound	Mean RRF	RRF	Minimum RF	%D		%D Limit
Dichlorodifluoromethane (Freon 12)	0.19	0.28	0.1	42.8	+	30
Chloroform	0.30	0.30	0.2	0.3		30
1,2-DCA-d4	0.27	0.27	0.1	2.6		30
Carbon Tetrachloride	0.29	0.29	0.1	0.0		30
Dibromomethane	0.17	0.17	0.1	3.0		30
Trichloroethene	0.27	0.27	0.2	-0.4		30
Toluene-d8	1.40	1.38	0.4	-1.6		30
Tetrachloroethene	0.52	0.52	0.2	-0.4		30
Bromofluorobenzene	0.50	0.46	0.1	-7.6		30

BEACON ENVIRONMENTAL

2203A Commerce Road, Suite 1
Forest Hill, MD 21050 USA
1.410.838.8780

CERTIFICATE OF ANALYSIS

**University of Notre Dame**	**Site Name:** So-Cal Military Toxic Site	**Beacon Proposal:** 201201H01
Department of Biological Sciences	**Site Location:** Notspa, CA	**Lab Work Order:** 0005542
South Bend, IN 46556	**Project Manager:** Kristin Shrader-Frechette	**Reported:** 01/25/2021

Table 7 - Form VII A VOA

Volatile Organics Continuing Calibration Data

Sample No.: **B20L066-ICV1** **Sample Name:** LCSD/Second Source Verification/CALV

Analysis: A_TO-17 BES PSV (ug/m3) **Calibration:** BL00006 **Calibration Start Date:** 12/23/2020 6:48:00PM

Sequence: B20L066 **Instrument:** K System **Lab File ID:** Kd20122316.D **Calibration End Date:** 12/23/2020 11:06:00PM

+ values are outside method/contract required QC limits

Compound	Mean RRF	RRF	Min_RF	%D	Max %D
Dichlorodifluoromethane (Freon 12)	0.19	0.22	0.1	13.4	30
Chloroform	0.30	0.28	0.2	7.1	30
1,2-DCA-d4	0.26	0.25	0.1	5.6	30
Carbon Tetrachloride	0.29	0.28	0.1	3.6	30
Dibromomethane	0.17	0.17	0.1	1.7	30
Trichloroethene	0.27	0.27	0.2	1.3	30
Toluene-d8	1.40	1.37	0.4	2.2	30
Tetrachloroethene	0.52	0.50	0.2	3.2	30
Bromofluorobenzene	0.50	0.48	0.1	3.7	30

BEACON ENVIRONMENTAL
2203A Commerce Road, Suite 1
Forest Hill, MD 21050 USA
1.410.838.8780

CERTIFICATE OF ANALYSIS

**University of Notre Dame**	**Site Name:** So-Cal Military Toxic Site	**Beacon Proposal:** 201201H01
Department of Biological Sciences	**Site Location:** Notspa, CA	**Lab Work Order:** 0005542
South Bend, IN 46556	**Project Manager:** Kristin Shrader-Frechette	**Reported:** 01/25/2021

Table 7 - Form VII A VOA

Volatile Organics Continuing Calibration Data

Sample No.: **21A0006-BS1**

Sample Name: LCS, Calibration Source Verification

Analysis: A_TO-17 BES PSV (ug/m3)

Calibration: BL00006

Calibration Start Date: 12/23/2020 6:48:00PM

Sequence: B21A005 **Instrument:** K System

Lab File ID: K21010502.D

Calibration End Date: 12/23/2020 11:06:00PM

+ values are outside method/contract required QC limits

Compound	Mean RRF	RRF	Min_RF	%D	Max %D
Dichlorodifluoromethane (Freon 12)	0.19	0.23	0.1	18.6	30
Chloroform	0.30	0.31	0.2	2.9	30
1,2-DCA-d4	0.26	0.29	0.1	9.5	30
Carbon Tetrachloride	0.29	0.28	0.1	3.6	30
Dibromomethane	0.17	0.18	0.1	7.7	30
Trichloroethene	0.27	0.28	0.2	2.4	30
Toluene-d8	1.40	1.43	0.4	2.1	30
Tetrachloroethene	0.52	0.54	0.2	4.5	30
Bromofluorobenzene	0.50	0.46	0.1	7.7	30

2203A Commerce Road, Suite 1
Forest Hill, MD 21050 USA
1.410.838.8780

BEACON ENVIRONMENTAL

CERTIFICATE OF ANALYSIS

**University of Notre Dame**	**Site Name:** So-Cal Military Toxic Site	**Beacon Proposal:** 201201H01
Department of Biological Sciences	**Site Location:** Notspa, CA	**Lab Work Order:** 0005542
South Bend, IN 46556	**Project Manager:** Kristin Shrader-Frechette	**Reported:** 01/25/2021

Table 7 - Form VII A VOA

Volatile Organics Continuing Calibration Data

Sample No.: **B21A005-CCV1**
Sample Name: LCS, Closing Calibration Verification
Analysis: A_TO-17 BES PSV (ug/m3)
Calibration: BL00006
Calibration Start Date: 12/23/2020 6:48:00PM
Sequence: B21A005 **Instrument:** K System
Lab File ID: K21010517.D
Calibration End Date: 12/23/2020 11:06:00PM

+ values are outside method/contract required QC limits

Compound	Mean RRF	RRF	Min_RF	%D	Max %D
Dichlorodifluoromethane (Freon 12)	0.19	0.32	0.1	65.0 +	50
Chloroform	0.30	0.30	0.2	0.4	50
1,2-DCA-d4	0.26	0.26	0.1	1.8	50
Carbon Tetrachloride	0.29	0.29	0.1	0.2	50
Dibromomethane	0.17	0.17	0.1	1.7	50
Trichloroethene	0.27	0.27	0.2	1.3	50
Toluene-d8	1.40	1.39	0.4	0.7	50
Tetrachloroethene	0.52	0.53	0.2	2.6	50
Bromofluorobenzene	0.50	0.48	0.1	3.7	50

BEACON ENVIRONMENTAL
2203A Commerce Road, Suite 1
Forest Hill, MD 21050 USA
1.410.838.8780

CERTIFICATE OF ANALYSIS

**University of Notre Dame**	**Site Name:** So-Cal Military Toxic Site	**Beacon Proposal:** 201201H01
Department of Biological Sciences	**Site Location:** Notspa, CA	**Lab Work Order:** 0005542
South Bend, IN 46556	**Project Manager:** Kristin Shrader-Frechette	**Reported:** 01/25/2021

Table 7 - Form VII A VOA

Volatile Organics Continuing Calibration Data

Sample No.: **B21A005-ICV1**
Sample Name: LCSD/Second Source Verification/CALV
Analysis: A_TO-17 BES PSV (ug/m3)
Calibration: BL00006
Calibration Start Date: 12/23/2020 6:48:00PM
Sequence: B21A005 **Instrument:** K System
Lab File ID: K21010504.D
Calibration End Date: 12/23/2020 11:06:00PM

+ values are outside method/contract required QC limits

Compound	Mean RRF	RRF	Min_RF	%D	Max %D
Dichlorodifluoromethane (Freon 12)	0.19	0.22	0.1	13.4	30
Chloroform	0.30	0.29	0.2	3.8	30
1,2-DCA-d4	0.26	0.27	0.1	1.9	30
Carbon Tetrachloride	0.29	0.27	0.1	7.0	30
Dibromomethane	0.17	0.18	0.1	7.7	30
Trichloroethene	0.27	0.28	0.2	2.4	30
Toluene-d8	1.40	1.35	0.4	3.6	30
Tetrachloroethene	0.52	0.51	0.2	1.3	30
Bromofluorobenzene	0.50	0.44	0.1	11.7	30

BEACON ENVIRONMENTAL

2203A Commerce Road, Suite 1
Forest Hill, MD 21050 USA
1.410.838.8780

CERTIFICATE OF ANALYSIS

**University of Notre Dame**	**Site Name:** So-Cal Military Toxic Site	**Beacon Proposal:** 201201H01
Department of Biological Sciences	**Site Location:** Notspa, CA	**Lab Work Order:** 0005542
South Bend, IN 46556	**Project Manager:** Kristin Shrader-Frechette	**Reported:** 01/25/2021

Table 7 - Form VII A VOA

Volatile Organics Continuing Calibration Data

Sample No.: **21A0007-BS1**

Sample Name: LCS, Calibration Source Verification

Analysis: A_TO-17 BES PSV (ug/m3)

Calibration: BL00006

Calibration Start Date: 12/23/2020 6:48:00PM

Sequence: B21A006

Instrument: K System

Lab File ID: K21010602.D

Calibration End Date: 12/23/2020 11:06:00PM

+ values are outside method/contract required QC limits

Compound	Mean RRF	RRF	Min_RF	%D	Max %D
Dichlorodifluoromethane (Freon 12)	0.19	0.19	0.1	2.1	30
Chloroform	0.30	0.31	0.2	2.9	30
1,2-DCA-d4	0.26	0.29	0.1	9.5	30
Carbon Tetrachloride	0.29	0.28	0.1	3.6	30
Dibromomethane	0.17	0.18	0.1	7.7	30
Trichloroethene	0.27	0.28	0.2	2.4	30
Toluene-d8	1.40	1.40	0.4	0.0	30
Tetrachloroethene	0.52	0.53	0.2	2.6	30
Bromofluorobenzene	0.50	0.46	0.1	7.7	30

BEACON ENVIRONMENTAL
2203A Commerce Road, Suite 1
Forest Hill, MD 21050 USA
1.410.838.8780

CERTIFICATE OF ANALYSIS

**University of Notre Dame**	**Site Name:** So-Cal Military Toxic Site	**Beacon Proposal:** 201201H01
Department of Biological Sciences	**Site Location:** Notspa, CA	**Lab Work Order:** 0005542
South Bend, IN 46556	**Project Manager:** Kristin Shrader-Frechette	**Reported:** 01/25/2021

Table 7 - Form VII A VOA

Volatile Organics Continuing Calibration Data

Sample No.: **B21A006-CCV1** **Sample Name:** LCS, Closing Calibration Verification

Analysis: A_TO-17 BES PSV (ug/m3) **Calibration:** BL00006 **Calibration Start Date:** 12/23/2020 6:48:00PM

Sequence: B21A006 **Instrument:** K System **Lab File ID:** K21010607.D **Calibration End Date:** 12/23/2020 11:06:00PM

+ values are outside method/contract reauired OC limits

Compound	Mean RRF	RRF	Min_RF	%D	Max %D
Dichlorodifluoromethane (Freon 12)	0.19	0.23	0.1	18.6	50
Chloroform	0.30	0.30	0.2	0.4	50
1,2-DCA-d4	0.26	0.27	0.1	1.9	50
Carbon Tetrachloride	0.29	0.28	0.1	3.6	50
Dibromomethane	0.17	0.17	0.1	1.7	50
Trichloroethene	0.27	0.28	0.2	2.4	50
Toluene-d8	1.40	1.41	0.4	0.7	50
Tetrachloroethene	0.52	0.52	0.2	0.7	50
Bromofluorobenzene	0.50	0.46	0.1	7.7	50

BEACON ENVIRONMENTAL
2203A Commerce Road, Suite 1
Forest Hill, MD 21050 USA
1.410.838.8780

CERTIFICATE OF ANALYSIS

**University of Notre Dame**	**Site Name:** So-Cal Military Toxic Site	**Beacon Proposal:** 201201H01
Department of Biological Sciences	**Site Location:** Notspa, CA	**Lab Work Order:** 0005542
South Bend, IN 46556	**Project Manager:** Kristin Shrader-Frechette	**Reported:** 01/25/2021

Table 7 - Form VII A VOA

Volatile Organics Continuing Calibration Data

Sample No.: B21A006-ICV1 **Sample Name:** LCSD/Second Source Verification/CALV

Analysis: A_TO-17 BES PSV (ug/m3) **Calibration:** BL00006 **Calibration Start Date:** 12/23/2020 6:48:00PM

Sequence: B21A006 **Instrument:** K System **Lab File ID:** K21010604.D **Calibration End Date:** 12/23/2020 11:06:00PM

+ values are outside method/contract reauired OC limits

Compound	Mean RRF	RRF	Min_RF	%D	Max %D
Dichlorodifluoromethane (Freon 12)	0.19	0.20	0.1	3.1	30
Chloroform	0.30	0.31	0.2	2.9	30
1,2-DCA-d4	0.26	0.28	0.1	5.7	30
Carbon Tetrachloride	0.29	0.28	0.1	3.6	30
Dibromomethane	0.17	0.18	0.1	7.7	30
Trichloroethene	0.27	0.29	0.2	6.0	30
Toluene-d8	1.40	1.40	0.4	0.0	30
Tetrachloroethene	0.52	0.52	0.2	0.7	30
Bromofluorobenzene	0.50	0.46	0.1	7.7	30

BEACON ENVIRONMENTAL

2203A Commerce Road, Suite 1
Forest Hill, MD 21050 USA
1.410.838.8780

CERTIFICATE OF ANALYSIS

**University of Notre Dame**	**Site Name:** So-Cal Military Toxic Site	**Beacon Proposal:** 201201H01
Department of Biological Sciences	**Site Location:** Notspa, CA	**Lab Work Order:** 0005542
South Bend, IN 46556	**Project Manager:** Kristin Shrader-Frechette	**Reported:** 01/25/2021

Table 8 - Form VIII A VOA
Volatile Internal Standard Area and Retention Time Summary

Sequence: B20L066 **Instrument:** K System **Calibration:** BL00006

Matrix: Air **Calibration Date:** 12/23/2020

+ values are outside method/contract required QC limits

Initial Calibration Reference Values

**Reference Value (RV) File ID:** Kd20122307.D	**Fluorobenzene**		**Chlorobenzene-d5**		**1,4-Dichlorobenzene-d4**	
	**Response**	**RT**	**Response**	**RT**	**Response**	**RT**
**Reference Value**	343638	5.566	219120	9.05	114663	10.74
**Upper Limit**	481093	5.69	306768	9.17	160528	10.86
**Lower Limit**	206183	5.45	131472	8.93	68798	10.62

			**Fluorobenzene**		**Chlorobenzene-d5**		**1,4-Dichlorobenzene-d4**	
**LabNumber**	**RV**	**FileID**	**Response**	**RT**	**Response**	**RT**	**Response**	**RT**
B20L066-CAL1		Kd20122303.D	381328	5.57	223052	9.05	103514	10.74
B20L066-CAL2		Kd20122304.D	368162	5.56	216664	9.05	103906	10.74
B20L066-CAL3		Kd20122305.D	329400	5.57	203352	9.05	95591	10.74
B20L066-CAL4		Kd20122306.D	358346	5.57	219414	9.05	114208	10.74
B20L066-CAL5	RV	Kd20122307.D	343638	5.57	219120	9.05	114663	10.74
B20L066-CAL6		Kd20122308.D	355146	5.56	223974	9.05	120980	10.73
B20L066-CAL7		Kd20122309.D	365361	5.56	234115	9.05	126411	10.73
B20L066-CAL8		Kd20122310.D	370262	5.57	232223	9.05	126762	10.73
B20L066-CAL9		Kd20122311.D	358222	5.57	234704	9.05	127830	10.74
B20L066-CALA		Kd20122312.D	379293	5.57	240640	9.05	131877	10.73
B20L066-CALB		Kd20122313.D	354356	5.56	234891	9.05	131477	10.73
B20L066-ICV1		Kd20122316.D	323148	5.57	214690	9.05	110480	10.74
B20L066-ICB1		Kd20122318.D	303557	5.57	203377	9.05	99551	10.73

BEACON ENVIRONMENTAL

2203A Commerce Road, Suite 1
Forest Hill, MD 21050 USA
1.410.838.8780

CERTIFICATE OF ANALYSIS

**University of Notre Dame**	**Site Name:** So-Cal Military Toxic Site	**Beacon Proposal:** 201201H01
Department of Biological Sciences	**Site Location:** Notspa, CA	**Lab Work Order:** 0005542
South Bend, IN 46556	**Project Manager:** Kristin Shrader-Frechette	**Reported:** 01/25/2021

Table 8 - Form VIII A VOA
Volatile Internal Standard Area and Retention Time Summary

Sequence: B21A005 **Instrument:** K System **Calibration:** BL00006
Matrix: Air **Calibration Date:** 12/23/2020

+ values are outside method/contract required QC limits

Initial Calibration Reference Values						
**Reference Value (RV) File ID:** Kd20122307.D	**Fluorobenzene**		**Chlorobenzene-d5**		**1,4-Dichlorobenzene-d4**	
	**Response**	**RT**	**Response**	**RT**	**Response**	**RT**
**Reference Value**	343638	5.566	219120	9.05	114663	10.74
**Upper Limit**	481093	5.69	306768	9.17	160528	10.86
**Lower Limit**	206183	5.45	131472	8.93	68798	10.62

			**Fluorobenzene**		**Chlorobenzene-d5**		**1,4-Dichlorobenzene-d4**	
**LabNumber**	**RV**	**FileID**	**Response**	**RT**	**Response**	**RT**	**Response**	**RT**
21A0006-BS1		K21010502.D	354631	5.57	207902	9.05	106802	10.74
21A0006-BLK1		K21010503.D	347077	5.57	205249	9.05	94877	10.74
B21A005-ICV1		K21010504.D	346591	5.56	232529	9.05	113623	10.74
0005542-01		K21010505.D	281914	5.56	174322	9.05	90307	10.73
0005542-02		K21010506.D	268910	5.57	177723	9.05	92125	10.73
0005542-03		K21010507.D	270357	5.57	173704	9.05	91916	10.74
0005542-05		K21010509.D	267448	5.57	170012	9.05	91744	10.73
0005542-06		K21010510.D	232805	5.57	154911	9.05	81154	10.74
0005542-07		K21010511.D	257657	5.57	166288	9.05	88467	10.73
0005542-09		K21010513.D	259219	5.56	171858	9.05	92920	10.73
0005542-10		K21010514.D	244272	5.57	163285	9.05	85687	10.74
0005542-11		K21010515.D	209371	5.57	151442	9.05	77282	10.73
0005542-12		K21010516.D	241225	5.57	172094	9.05	91300	10.73
B21A005-CCV1		K21010517.D	293986	5.57	192875	9.05	104444	10.73

BEACON ENVIRONMENTAL

2203A Commerce Road, Suite 1
Forest Hill, MD 21050 USA
1.410.838.8780

CERTIFICATE OF ANALYSIS

**University of Notre Dame**	**Site Name:** So-Cal Military Toxic Site	**Beacon Proposal:** 201201H01
Department of Biological Sciences	**Site Location:** Notspa, CA	**Lab Work Order:** 0005542
South Bend, IN 46556	**Project Manager:** Kristin Shrader-Frechette	**Reported:** 01/25/2021

Table 8 - Form VIII A VOA
Volatile Internal Standard Area and Retention Time Summary

Sequence: B21A006 **Instrument:** K System **Calibration:** BL00006
Matrix: Air **Calibration Date:** 12/23/2020

+ values are outside method/contract required QC limits

Initial Calibration Reference Values

**Reference Value (RV) File ID:** Kd20122307.D	**Fluorobenzene**		**Chlorobenzene-d5**		**1,4-Dichlorobenzene-d4**	
	**Response**	**RT**	**Response**	**RT**	**Response**	**RT**
**Reference Value**	343638	5.566	219120	9.05	114663	10.74
**Upper Limit**	481093	5.69	306768	9.17	160528	10.86
**Lower Limit**	206183	5.45	131472	8.93	68798	10.62

			**Fluorobenzene**		**Chlorobenzene-d5**		**1,4-Dichlorobenzene-d4**	
**LabNumber**	**RV**	**FileID**	**Response**	**RT**	**Response**	**RT**	**Response**	**RT**
21A0007-BS1		K21010602.D	346645	5.56	208363	9.05	105954	10.74
21A0007-BLK1		K21010603.D	321818	5.57	196467	9.05	94490	10.74
B21A006-ICV1		K21010604.D	326425	5.57	200493	9.05	102568	10.73
0005542-04		K21010605.D	250824	5.57	165248	9.05	80491	10.74
0005542-08		K21010606.D	277937	5.56	168222	9.05	91431	10.73
B21A006-CCV1		K21010607.D	311164	5.57	192507	9.05	102020	10.74

2203A Commerce Road, Suite 1
Forest Hill, MD 21050 USA
1.410.838.8780

BEACON ENVIRONMENTAL

CERTIFICATE OF ANALYSIS

**University of Notre Dame**	**Site Name:** So-Cal Military Toxic Site	**Beacon Proposal:** 201201H01
Department of Biological Sciences	**Site Location:** Notspa, CA	**Lab Work Order:** 0005542
South Bend, IN 46556	**Project Manager:** Kristin Shrader-Frechette	**Reported:** 01/25/2021

Additional QC Information

BEACON ENVIRONMENTAL
2203A Commerce Road, Suite 1
Forest Hill, MD 21050 USA
1.410.838.8780

CERTIFICATE OF ANALYSIS

**University of Notre Dame**<br>Department of Biological Sciences<br>South Bend, IN 46556	**Site Name:** So-Cal Military Toxic Site<br>**Site Location:** Notspa, CA<br>**Project Manager:** Kristin Shrader-Frechette	**Beacon Proposal:** 201201H01<br>**Lab Work Order:** 0005542<br>**Reported:** 01/25/2021

TO-17 (Passive) Holding Time Report

Analysis: TO-17 (Passive)- Concentration

SampleName	Sampled	Received	Prepared	Days to Prep	Analyzed	Days to Analysis	Expiration Date	Qualifier
A	01/02/2021 09:24	01/04/2021 13:30	01/05/2021 15:38	3.26	01/05/2021 15:38	3.26	02/02/2021	
B	01/02/2021 09:15	01/04/2021 13:30	01/05/2021 16:07	3.29	01/05/2021 16:07	3.29	02/02/2021	
C	01/02/2021 09:19	01/04/2021 13:30	01/05/2021 16:37	3.30	01/05/2021 16:37	3.30	02/02/2021	
D	01/02/2021 09:09	01/04/2021 13:30	01/06/2021 09:35	4.02	01/06/2021 09:35	4.02	02/02/2021	
E	01/02/2021 09:46	01/04/2021 13:30	01/05/2021 17:36	3.33	01/05/2021 17:36	3.33	02/02/2021	
F	01/02/2021 09:44	01/04/2021 13:30	01/05/2021 18:06	3.35	01/05/2021 18:06	3.35	02/02/2021	
G	01/02/2021 09:36	01/04/2021 13:30	01/05/2021 18:36	3.38	01/05/2021 18:36	3.38	02/02/2021	
H	01/02/2021 09:38	01/04/2021 13:30	01/06/2021 10:05	4.02	01/06/2021 10:05	4.02	02/02/2021	
I	01/02/2021 09:40	01/04/2021 13:30	01/05/2021 19:37	3.41	01/05/2021 19:37	3.41	02/02/2021	
J	01/02/2021 09:42	01/04/2021 13:30	01/05/2021 20:06	3.43	01/05/2021 20:06	3.43	02/02/2021	
J-DUP	01/02/2021 09:42	01/04/2021 13:30	01/05/2021 20:37	3.45	01/05/2021 20:37	3.45	02/02/2021	
K	01/02/2021 09:32	01/04/2021 13:30	01/05/2021 21:06	3.48	01/05/2021 21:06	3.48	02/02/2021	

H = Sample analysis exceeded holding time.

2203A Commerce Road, Suite 1
Forest Hill, MD 21050 USA
1.410.838.8780

BEACON ENVIRONMENTAL

CERTIFICATE OF ANALYSIS

**University of Notre Dame**	**Site Name:** So-Cal Military Toxic Site	**Beacon Proposal:** 201201H01
Department of Biological Sciences	**Site Location:** Notspa, CA	**Lab Work Order:** 0005542
South Bend, IN 46556	**Project Manager:** Kristin Shrader-Frechette	**Reported:** 01/25/2021

Sample Preparation and Analysis Summary Table

Batch	Sequence	Instrument	FileID	Analyzed Date/Time	Dilution	Analysis
**Lab ID:** 0005542-01	**Method:** TO-17 (Passive)		**Sample:** A			**Matrix:** Air
21A0006	B21A005	K System	K21010505.D	1/5/21 15:38	1.00	A_TO-17 BES PSV (ug/m3)
**Lab ID:** 0005542-02	**Method:** TO-17 (Passive)		**Sample:** B			**Matrix:** Air
21A0006	B21A005	K System	K21010506.D	1/5/21 16:07	1.00	A_TO-17 BES PSV (ug/m3)
**Lab ID:** 0005542-03	**Method:** TO-17 (Passive)		**Sample:** C			**Matrix:** Air
21A0006	B21A005	K System	K21010507.D	1/5/21 16:37	1.00	A_TO-17 BES PSV (ug/m3)
**Lab ID:** 0005542-04	**Method:** TO-17 (Passive)		**Sample:** D			**Matrix:** Air
21A0007	B21A006	K System	K21010605.D	1/6/21 9:35	1.00	A_TO-17 BES PSV (ug/m3)
**Lab ID:** 0005542-05	**Method:** TO-17 (Passive)		**Sample:** E			**Matrix:** Air
21A0006	B21A005	K System	K21010509.D	1/5/21 17:36	1.00	A_TO-17 BES PSV (ug/m3)
**Lab ID:** 0005542-06	**Method:** TO-17 (Passive)		**Sample:** F			**Matrix:** Air
21A0006	B21A005	K System	K21010510.D	1/5/21 18:06	1.00	A_TO-17 BES PSV (ug/m3)
**Lab ID:** 0005542-07	**Method:** TO-17 (Passive)		**Sample:** G			**Matrix:** Air
21A0006	B21A005	K System	K21010511.D	1/5/21 18:36	1.00	A_TO-17 BES PSV (ug/m3)
**Lab ID:** 0005542-08	**Method:** TO-17 (Passive)		**Sample:** H			**Matrix:** Air
21A0007	B21A006	K System	K21010606.D	1/6/21 10:05	1.00	A_TO-17 BES PSV (ug/m3)
**Lab ID:** 0005542-09	**Method:** TO-17 (Passive)		**Sample:** I			**Matrix:** Air
21A0006	B21A005	K System	K21010513.D	1/5/21 19:37	1.00	A_TO-17 BES PSV (ug/m3)
**Lab ID:** 0005542-10	**Method:** TO-17 (Passive)		**Sample:** J			**Matrix:** Air
21A0006	B21A005	K System	K21010514.D	1/5/21 20:06	1.00	A_TO-17 BES PSV (ug/m3)
**Lab ID:** 0005542-11	**Method:** TO-17 (Passive)		**Sample:** J-DUP			**Matrix:** Air
21A0006	B21A005	K System	K21010515.D	1/5/21 20:37	1.00	A_TO-17 BES PSV (ug/m3)
**Lab ID:** 0005542-12	**Method:** TO-17 (Passive)		**Sample:** K			**Matrix:** Air
21A0006	B21A005	K System	K21010516.D	1/5/21 21:06	1.00	A_TO-17 BES PSV (ug/m3)

BEACON ENVIRONMENTAL
2203A Commerce Road, Suite 1
Forest Hill, MD 21050 USA
1.410.838.8780

CERTIFICATE OF ANALYSIS

**University of Notre Dame**	**Site Name:** So-Cal Military Toxic Site	**Beacon Proposal:** 201201H01
Department of Biological Sciences	**Site Location:** Notspa, CA	**Lab Work Order:** 0005542
South Bend, IN 46556	**Project Manager:** Kristin Shrader-Frechette	**Reported:** 01/25/2021

Sample Result Calculation Summary (Concentration)

TO-17 (Passive)

Analyte	t<br>Sampling Time<br>minutes	DF<br>Dilution<br>Factor	Uc<br>Uptake<br>Rate	M<br>Initial Result<br>ng	C<br>Calculated Result<br>µg/m³	File ID
**Lab ID:** 0005542-01 **Sample Name:** A					**X̄ Temp (°C):** 16.11	
Dichlorodifluoromethane (Freon 12)	20,199	1.00	0.579 g	U	U	K21010505.D
Chloroform	20,199	1.00	0.350 g	U	U	K21010505.D
Carbon Tetrachloride	20,199	1.00	0.430 g	U	U	K21010505.D
Dibromomethane	20,199	1.00	0.400 g	U	U	K21010505.D
Trichloroethene	20,199	1.00	0.330	U	U	K21010505.D
Tetrachloroethene	20,199	1.00	0.410	65.95	7.97	K21010505.D
**Lab ID:** 0005542-02 **Sample Name:** B					**X̄ Temp (°C):** 16.11	
Dichlorodifluoromethane (Freon 12)	20,206	1.00	0.579 g	5.67	0.484	K21010506.D
Chloroform	20,206	1.00	0.350 g	U	U	K21010506.D
Carbon Tetrachloride	20,206	1.00	0.430 g	5.89	0.679	K21010506.D
Dibromomethane	20,206	1.00	0.400 g	U	U	K21010506.D
Trichloroethene	20,206	1.00	0.330	U	U	K21010506.D
Tetrachloroethene	20,206	1.00	0.410	110.64	13.4	K21010506.D
**Lab ID:** 0005542-03 **Sample Name:** C					**X̄ Temp (°C):** 16.11	
Dichlorodifluoromethane (Freon 12)	20,199	1.00	0.579 g	U	U	K21010507.D
Chloroform	20,199	1.00	0.350 g	U	U	K21010507.D
Carbon Tetrachloride	20,199	1.00	0.430 g	U	U	K21010507.D
Dibromomethane	20,199	1.00	0.400 g	U	U	K21010507.D
Trichloroethene	20,199	1.00	0.330	U	U	K21010507.D
Tetrachloroethene	20,199	1.00	0.410	58.05	7.02	K21010507.D
**Lab ID:** 0005542-04 **Sample Name:** D					**X̄ Temp (°C):** 16.11	
Dichlorodifluoromethane (Freon 12)	20,204	1.00	0.579 g	U	U	K21010605.D
Chloroform	20,204	1.00	0.350 g	U	U	K21010605.D
Carbon Tetrachloride	20,204	1.00	0.430 g	U	U	K21010605.D
Dibromomethane	20,204	1.00	0.400 g	U	U	K21010605.D
Trichloroethene	20,204	1.00	0.330	U	U	K21010605.D
Tetrachloroethene	20,204	1.00	0.410	106.10	12.8	K21010605.D
**Lab ID:** 0005542-05 **Sample Name:** E					**X̄ Temp (°C):** 15.56	
Dichlorodifluoromethane (Freon 12)	20,180	1.00	0.579 g	U	U	K21010509.D
Chloroform	20,180	1.00	0.349 g	U	U	K21010509.D
Carbon Tetrachloride	20,180	1.00	0.429 g	U	U	K21010509.D
Dibromomethane	20,180	1.00	0.399 g	U	U	K21010509.D
Trichloroethene	20,180	1.00	0.329	U	U	K21010509.D
Tetrachloroethene	20,180	1.00	0.409	14.40	1.74	K21010509.D

BEACON ENVIRONMENTAL

2203A Commerce Road, Suite 1
Forest Hill, MD 21050 USA
1.410.838.8780

CERTIFICATE OF ANALYSIS

**University of Notre Dame**	**Site Name:** So-Cal Military Toxic Site	**Beacon Proposal:** 201201H01
Department of Biological Sciences	**Site Location:** Notspa, CA	**Lab Work Order:** 0005542
South Bend, IN 46556	**Project Manager:** Kristin Shrader-Frechette	**Reported:** 01/25/2021

Sample Result Calculation Summary (Concentration)

TO-17 (Passive)

Analyte	t<br>Sampling Time<br>minutes	DF<br>Dilution<br>Factor	Uc<br>Uptake<br>Rate	M<br>Initial Result<br>ng	C<br>Calculated Result<br>µg/m³	File ID
**Lab ID:** 0005542-06 **Sample Name:** F **X̄ Temp (°C):** 15.56						
Dichlorodifluoromethane (Freon 12)	20,181	1.00	0.579 g	21.40	1.83	K21010510.D
Chloroform	20,181	1.00	0.349 g	U	U	K21010510.D
Carbon Tetrachloride	20,181	1.00	0.429 g	U	U	K21010510.D
Dibromomethane	20,181	1.00	0.399 g	U	U	K21010510.D
Trichloroethene	20,181	1.00	0.329	U	U	K21010510.D
Tetrachloroethene	20,181	1.00	0.409	36.67	4.44	K21010510.D
**Lab ID:** 0005542-07 **Sample Name:** G **X̄ Temp (°C):** 15.56						
Dichlorodifluoromethane (Freon 12)	20,195	1.00	0.579 g	U	U	K21010511.D
Chloroform	20,195	1.00	0.349 g	U	U	K21010511.D
Carbon Tetrachloride	20,195	1.00	0.429 g	U	U	K21010511.D
Dibromomethane	20,195	1.00	0.399 g	U	U	K21010511.D
Trichloroethene	20,195	1.00	0.329	U	U	K21010511.D
Tetrachloroethene	20,195	1.00	0.409	21.57	2.61	K21010511.D
**Lab ID:** 0005542-08 **Sample Name:** H **X̄ Temp (°C):** 15.56						
Dichlorodifluoromethane (Freon 12)	20,192	1.00	0.579 g	U	U	K21010606.D
Chloroform	20,192	1.00	0.349 g	U	U	K21010606.D
Carbon Tetrachloride	20,192	1.00	0.429 g	U	U	K21010606.D
Dibromomethane	20,192	1.00	0.399 g	U	U	K21010606.D
Trichloroethene	20,192	1.00	0.329	U	U	K21010606.D
Tetrachloroethene	20,192	1.00	0.409	11.80	1.43	K21010606.D
**Lab ID:** 0005542-09 **Sample Name:** I **X̄ Temp (°C):** 15.56						
Dichlorodifluoromethane (Freon 12)	20,189	1.00	0.579 g	20.88	1.79	K21010513.D
Chloroform	20,189	1.00	0.349 g	U	U	K21010513.D
Carbon Tetrachloride	20,189	1.00	0.429 g	U	U	K21010513.D
Dibromomethane	20,189	1.00	0.399 g	U	U	K21010513.D
Trichloroethene	20,189	1.00	0.329	U	U	K21010513.D
Tetrachloroethene	20,189	1.00	0.409	24.10	2.92	K21010513.D
**Lab ID:** 0005542-10 **Sample Name:** J **X̄ Temp (°C):** 15.56						
Dichlorodifluoromethane (Freon 12)	20,186	1.00	0.579 g	U	U	K21010514.D
Chloroform	20,186	1.00	0.349 g	U	U	K21010514.D
Carbon Tetrachloride	20,186	1.00	0.429 g	U	U	K21010514.D
Dibromomethane	20,186	1.00	0.399 g	U	U	K21010514.D
Trichloroethene	20,186	1.00	0.329	U	U	K21010514.D
Tetrachloroethene	20,186	1.00	0.409	12.41	1.50	K21010514.D

BEACON ENVIRONMENTAL

2203A Commerce Road, Suite 1
Forest Hill, MD 21050 USA
1.410.838.8780

CERTIFICATE OF ANALYSIS

**University of Notre Dame**	**Site Name:** So-Cal Military Toxic Site	**Beacon Proposal:** 201201H01
Department of Biological Sciences	**Site Location:** Notspa, CA	**Lab Work Order:** 0005542
South Bend, IN 46556	**Project Manager:** Kristin Shrader-Frechette	**Reported:** 01/25/2021

Sample Result Calculation Summary (Concentration)

TO-17 (Passive)

Analyte	**t** Sampling Time minutes	**DF** Dilution Factor	**Uc** Uptake Rate	**M** Initial Result ng	**C** Calculated Result µg/m³	File ID
**Lab ID:** 0005542-11 **Sample Name:** J-DUP **x̄ Temp (°C):** 15.56						
Dichlorodifluoromethane (Freon 12)	20,186	1.00	0.579 g	U	U	K21010515.D
Chloroform	20,186	1.00	0.349 g	U	U	K21010515.D
Carbon Tetrachloride	20,186	1.00	0.429 g	U	U	K21010515.D
Dibromomethane	20,186	1.00	0.399 g	U	U	K21010515.D
Trichloroethene	20,186	1.00	0.329	U	U	K21010515.D
Tetrachloroethene	20,186	1.00	0.409	13.49	1.63	K21010515.D
**Lab ID:** 0005542-12 **Sample Name:** K **x̄ Temp (°C):** 15.56						
Dichlorodifluoromethane (Freon 12)	20,194	1.00	0.579 g	5.43	0.464	K21010516.D
Chloroform	20,194	1.00	0.349 g	U	U	K21010516.D
Carbon Tetrachloride	20,194	1.00	0.429 g	U	U	K21010516.D
Dibromomethane	20,194	1.00	0.399 g	U	U	K21010516.D
Trichloroethene	20,194	1.00	0.329	U	U	K21010516.D
Tetrachloroethene	20,194	1.00	0.409	14.13	1.71	K21010516.D

Calculations:

$$C = \frac{1000 \times M \times DF}{Uc \times t}$$

$$Uc = U * \left(\left(\frac{Ts + 273.15}{Tu + 273.15}\right)^{1/2}\right)$$

where:
- C = concentration (µg/m³)
- M = mass (ng)
- DF = dilution factor
- Uc = uptake rate (ml/min), corrected
- t = sampling time (minutes)
- U = compound specific uptake rate
- Tu = uptake rate study temperature
- Ts = sample average temperature

Note: Tu is 16.65°C

g Uptake rate determined using Graham's Law of Diffusion.

Reference: Federal Register/Vol. 79, No. 125/June 30, 2014

BEACON ENVIRONMENTAL

2203A Commerce Road, Suite 1
Forest Hill, MD 21050 USA
1.410.838.8780

CERTIFICATE OF ANALYSIS

**University of Notre Dame**	**Site Name:** So-Cal Military Toxic Site	**Beacon Proposal:** 201201H01
Department of Biological Sciences	**Site Location:** Notspa, CA	**Lab Work Order:** 0005542
South Bend, IN 46556	**Project Manager:** Kristin Shrader-Frechette	**Reported:** 01/25/2021

Method Detection and Reporting Limit Calculations (Concentration)

TO-17 (Passive)

Analyte	**t** Sampling Time minutes	**DF** Dilution Factor	**Uc** Uptake Rate	**M** Initial (ng) LOQ	LOD	DL	**C** Calculated (µg/m³) LOQ	LOD	DL
**Lab ID:** 0005542-01 **Sample Name:** A **x̄ Temp (°C):** 16.11									
Dichlorodifluoromethane (Freon 12)	20,199	1.00	0.579 [g]	10.0	5.00	5.00	0.854	0.427	0.427
Chloroform	20,199	1.00	0.350 [g]	10.0	5.00	5.00	1.42	0.708	0.708
Carbon Tetrachloride	20,199	1.00	0.430 [g]	10.0	5.00	5.00	1.15	0.576	0.576
Dibromomethane	20,199	1.00	0.400 [g]	10.0	5.00	5.00	1.24	0.619	0.619
Trichloroethene	20,199	1.00	0.330	10.0	5.00	5.00	1.50	0.751	0.751
Tetrachloroethene	20,199	1.00	0.410	10.0	5.00	5.00	1.21	0.604	0.604
**Lab ID:** 0005542-02 **Sample Name:** B **x̄ Temp (°C):** 16.11									
Dichlorodifluoromethane (Freon 12)	20,206	1.00	0.579 [g]	10.0	5.00	5.00	0.854	0.427	0.427
Chloroform	20,206	1.00	0.350 [g]	10.0	5.00	5.00	1.42	0.708	0.708
Carbon Tetrachloride	20,206	1.00	0.430 [g]	10.0	5.00	5.00	1.15	0.576	0.576
Dibromomethane	20,206	1.00	0.400 [g]	10.0	5.00	5.00	1.24	0.619	0.619
Trichloroethene	20,206	1.00	0.330	10.0	5.00	5.00	1.50	0.751	0.751
Tetrachloroethene	20,206	1.00	0.410	10.0	5.00	5.00	1.21	0.604	0.604
**Lab ID:** 0005542-03 **Sample Name:** C **x̄ Temp (°C):** 16.11									
Dichlorodifluoromethane (Freon 12)	20,199	1.00	0.579 [g]	10.0	5.00	5.00	0.854	0.427	0.427
Chloroform	20,199	1.00	0.350 [g]	10.0	5.00	5.00	1.42	0.708	0.708
Carbon Tetrachloride	20,199	1.00	0.430 [g]	10.0	5.00	5.00	1.15	0.576	0.576
Dibromomethane	20,199	1.00	0.400 [g]	10.0	5.00	5.00	1.24	0.619	0.619
Trichloroethene	20,199	1.00	0.330	10.0	5.00	5.00	1.50	0.751	0.751
Tetrachloroethene	20,199	1.00	0.410	10.0	5.00	5.00	1.21	0.604	0.604
**Lab ID:** 0005542-04 **Sample Name:** D **x̄ Temp (°C):** 16.11									
Dichlorodifluoromethane (Freon 12)	20,204	1.00	0.579 [g]	10.0	5.00	5.00	0.854	0.427	0.427
Chloroform	20,204	1.00	0.350 [g]	10.0	5.00	5.00	1.42	0.708	0.708
Carbon Tetrachloride	20,204	1.00	0.430 [g]	10.0	5.00	5.00	1.15	0.576	0.576
Dibromomethane	20,204	1.00	0.400 [g]	10.0	5.00	5.00	1.24	0.619	0.619
Trichloroethene	20,204	1.00	0.330	10.0	5.00	5.00	1.50	0.751	0.751
Tetrachloroethene	20,204	1.00	0.410	10.0	5.00	5.00	1.21	0.604	0.604

BEACON ENVIRONMENTAL

2203A Commerce Road, Suite 1
Forest Hill, MD 21050 USA
1.410.838.8780

CERTIFICATE OF ANALYSIS

**University of Notre Dame**	**Site Name:** So-Cal Military Toxic Site	**Beacon Proposal:** 201201H01
Department of Biological Sciences	**Site Location:** Notspa, CA	**Lab Work Order:** 0005542
South Bend, IN 46556	**Project Manager:** Kristin Shrader-Frechette	**Reported:** 01/25/2021

Method Detection and Reporting Limit Calculations (Concentration)

TO-17 (Passive)

Lab ID: 0005542-05 **Sample Name:** E **x̄ Temp (°C):** 15.56

Analyte	t Sampling Time minutes	DF Dilution Factor	Uc Uptake Rate	M Initial (ng) LOQ	M LOD	M DL	C Calculated (µg/m³) LOQ	C LOD	C DL
Dichlorodifluoromethane (Freon 12)	20,180	1.00	0.579 g	10.0	5.00	5.00	0.856	0.428	0.428
Chloroform	20,180	1.00	0.349 g	10.0	5.00	5.00	1.42	0.709	0.709
Carbon Tetrachloride	20,180	1.00	0.429 g	10.0	5.00	5.00	1.15	0.577	0.577
Dibromomethane	20,180	1.00	0.399 g	10.0	5.00	5.00	1.24	0.621	0.621
Trichloroethene	20,180	1.00	0.329	10.0	5.00	5.00	1.50	0.752	0.752
Tetrachloroethene	20,180	1.00	0.409	10.0	5.00	5.00	1.21	0.605	0.605

Lab ID: 0005542-06 **Sample Name:** F **x̄ Temp (°C):** 15.56

Analyte	t Sampling Time minutes	DF Dilution Factor	Uc Uptake Rate	M Initial (ng) LOQ	M LOD	M DL	C Calculated (µg/m³) LOQ	C LOD	C DL
Dichlorodifluoromethane (Freon 12)	20,181	1.00	0.579 g	10.0	5.00	5.00	0.856	0.428	0.428
Chloroform	20,181	1.00	0.349 g	10.0	5.00	5.00	1.42	0.709	0.709
Carbon Tetrachloride	20,181	1.00	0.429 g	10.0	5.00	5.00	1.15	0.577	0.577
Dibromomethane	20,181	1.00	0.399 g	10.0	5.00	5.00	1.24	0.621	0.621
Trichloroethene	20,181	1.00	0.329	10.0	5.00	5.00	1.50	0.752	0.752
Tetrachloroethene	20,181	1.00	0.409	10.0	5.00	5.00	1.21	0.605	0.605

Lab ID: 0005542-07 **Sample Name:** G **x̄ Temp (°C):** 15.56

Analyte	t Sampling Time minutes	DF Dilution Factor	Uc Uptake Rate	M Initial (ng) LOQ	M LOD	M DL	C Calculated (µg/m³) LOQ	C LOD	C DL
Dichlorodifluoromethane (Freon 12)	20,195	1.00	0.579 g	10.0	5.00	5.00	0.855	0.428	0.428
Chloroform	20,195	1.00	0.349 g	10.0	5.00	5.00	1.42	0.709	0.709
Carbon Tetrachloride	20,195	1.00	0.429 g	10.0	5.00	5.00	1.15	0.577	0.577
Dibromomethane	20,195	1.00	0.399 g	10.0	5.00	5.00	1.24	0.620	0.620
Trichloroethene	20,195	1.00	0.329	10.0	5.00	5.00	1.50	0.752	0.752
Tetrachloroethene	20,195	1.00	0.409	10.0	5.00	5.00	1.21	0.605	0.605

Lab ID: 0005542-08 **Sample Name:** H **x̄ Temp (°C):** 15.56

Analyte	t Sampling Time minutes	DF Dilution Factor	Uc Uptake Rate	M Initial (ng) LOQ	M LOD	M DL	C Calculated (µg/m³) LOQ	C LOD	C DL
Dichlorodifluoromethane (Freon 12)	20,192	1.00	0.579 g	10.0	5.00	5.00	0.855	0.428	0.428
Chloroform	20,192	1.00	0.349 g	10.0	5.00	5.00	1.42	0.709	0.709
Carbon Tetrachloride	20,192	1.00	0.429 g	10.0	5.00	5.00	1.15	0.577	0.577
Dibromomethane	20,192	1.00	0.399 g	10.0	5.00	5.00	1.24	0.620	0.620
Trichloroethene	20,192	1.00	0.329	10.0	5.00	5.00	1.50	0.752	0.752
Tetrachloroethene	20,192	1.00	0.409	10.0	5.00	5.00	1.21	0.605	0.605

BEACON ENVIRONMENTAL

2203A Commerce Road, Suite 1
Forest Hill, MD 21050 USA
1.410.838.8780

CERTIFICATE OF ANALYSIS

**University of Notre Dame**	**Site Name:** So-Cal Military Toxic Site	**Beacon Proposal:** 201201H01
Department of Biological Sciences	**Site Location:** Notspa, CA	**Lab Work Order:** 0005542
South Bend, IN 46556	**Project Manager:** Kristin Shrader-Frechette	**Reported:** 01/25/2021

Method Detection and Reporting Limit Calculations (Concentration)

TO-17 (Passive)

Analyte	**t** Sampling Time minutes	**DF** Dilution Factor	**Uc** Uptake Rate	**M** Initial (ng)			**C** Calculated (µg/m³)		
				LOQ	LOD	DL	LOQ	LOD	DL
**Lab ID:** 0005542-09 **Sample Name:** I **X̄ Temp (°C):** 15.56									
Dichlorodifluoromethane (Freon 12)	20,189	1.00	0.579 [g]	10.0	5.00	5.00	0.856	0.428	0.428
Chloroform	20,189	1.00	0.349 [g]	10.0	5.00	5.00	1.42	0.709	0.709
Carbon Tetrachloride	20,189	1.00	0.429 [g]	10.0	5.00	5.00	1.15	0.577	0.577
Dibromomethane	20,189	1.00	0.399 [g]	10.0	5.00	5.00	1.24	0.620	0.620
Trichloroethene	20,189	1.00	0.329	10.0	5.00	5.00	1.50	0.752	0.752
Tetrachloroethene	20,189	1.00	0.409	10.0	5.00	5.00	1.21	0.605	0.605
**Lab ID:** 0005542-10 **Sample Name:** J **X̄ Temp (°C):** 15.56									
Dichlorodifluoromethane (Freon 12)	20,186	1.00	0.579 [g]	10.0	5.00	5.00	0.856	0.428	0.428
Chloroform	20,186	1.00	0.349 [g]	10.0	5.00	5.00	1.42	0.709	0.709
Carbon Tetrachloride	20,186	1.00	0.429 [g]	10.0	5.00	5.00	1.15	0.577	0.577
Dibromomethane	20,186	1.00	0.399 [g]	10.0	5.00	5.00	1.24	0.620	0.620
Trichloroethene	20,186	1.00	0.329	10.0	5.00	5.00	1.50	0.752	0.752
Tetrachloroethene	20,186	1.00	0.409	10.0	5.00	5.00	1.21	0.605	0.605
**Lab ID:** 0005542-11 **Sample Name:** J-DUP **X̄ Temp (°C):** 15.56									
Dichlorodifluoromethane (Freon 12)	20,186	1.00	0.579 [g]	10.0	5.00	5.00	0.856	0.428	0.428
Chloroform	20,186	1.00	0.349 [g]	10.0	5.00	5.00	1.42	0.709	0.709
Carbon Tetrachloride	20,186	1.00	0.429 [g]	10.0	5.00	5.00	1.15	0.577	0.577
Dibromomethane	20,186	1.00	0.399 [g]	10.0	5.00	5.00	1.24	0.620	0.620
Trichloroethene	20,186	1.00	0.329	10.0	5.00	5.00	1.50	0.752	0.752
Tetrachloroethene	20,186	1.00	0.409	10.0	5.00	5.00	1.21	0.605	0.605
**Lab ID:** 0005542-12 **Sample Name:** K **X̄ Temp (°C):** 15.56									
Dichlorodifluoromethane (Freon 12)	20,194	1.00	0.579 [g]	10.0	5.00	5.00	0.855	0.428	0.428
Chloroform	20,194	1.00	0.349 [g]	10.0	5.00	5.00	1.42	0.709	0.709
Carbon Tetrachloride	20,194	1.00	0.429 [g]	10.0	5.00	5.00	1.15	0.577	0.577
Dibromomethane	20,194	1.00	0.399 [g]	10.0	5.00	5.00	1.24	0.620	0.620
Trichloroethene	20,194	1.00	0.329	10.0	5.00	5.00	1.50	0.752	0.752
Tetrachloroethene	20,194	1.00	0.409	10.0	5.00	5.00	1.21	0.605	0.605

2203A Commerce Road, Suite 1
Forest Hill, MD 21050 USA
1.410.838.8780

CERTIFICATE OF ANALYSIS

**University of Notre Dame**	**Site Name:** So-Cal Military Toxic Site	**Beacon Proposal:** 201201H01
Department of Biological Sciences	**Site Location:** Notspa, CA	**Lab Work Order:** 0005542
South Bend, IN 46556	**Project Manager:** Kristin Shrader-Frechette	**Reported:** 01/25/2021

Laboratory Certification List

Certification ID	Certification No.	Description	Expires	Project Required
Alaska CS-LAP	19-002	Alaska Department of Environmental Conservation	01/31/2023	
DoD-ELAP	L20-532	United States Department of Defense Environmental Laboratory Accreditation	12/31/2022	
ISO/IEC 17025:2017	L20-532	General Requirements for the competence of Testing and Calibration Laboratories	12/31/2022	
NY-NELAC	12097	New York Department of Health	04/01/2021	
Utah-NELAC	MD01091	Utah Department of Health	12/31/2021	

BEACON ENVIRONMENTAL

2203A Commerce Road, Suite 1
Forest Hill, MD 21050 USA
1.410.838.8780

CERTIFICATE OF ANALYSIS

**University of Notre Dame**	**Site Name:** So-Cal Military Toxic Site	**Beacon Proposal:** 201201H01
Department of Biological Sciences	**Site Location:** Notspa, CA	**Lab Work Order:** 0005542
South Bend, IN 46556	**Project Manager:** Kristin Shrader-Frechette	**Reported:** 01/25/2021

Qualifiers/Notes and Definitions

General Definitions:

DF	Dilution Factor
DL	Detection Limit
LOD	Limit of Detection
LOQ	Limit of Quantitation
NA	Not Applicable
Q	Qualifier
RPD	Relative Percent Difference
RT	Retention Times in Minutes
RRT	Evaluation of Relative Retention Times in RRT Units (qualified if outside $\pm$0.06 control limits)
3σ	Uncertainty
∉	Compound not on scope of accreditation
+	values are outside method/contract required QC limits
∅	Compound not on scope of accreditation and analyzed with a one-point calibration

Sample/Sample Receipt Qualifiers and Notes:

J	Value reported below limit of quantitation (LOQ).
L	LCS recovery was out of method acceptance limits.
U	Analyte was not detected and is reported as less than the limit of detection (LOD). The LOD has been adjusted for any dilution or concentration of the sample.

BEACON ENVIRONMENTAL

2203A Commerce Road, Suite 1
Forest Hill, MD 21050 USA
1.410.838.8780

CERTIFICATE OF ANALYSIS

**University of Notre Dame**	**Site Name:** So-Cal Military Toxic Site	**Beacon Proposal:** 201201H01
Department of Biological Sciences	**Site Location:** Notspa, CA	**Lab Work Order:** 0005542
South Bend, IN 46556	**Project Manager:** Kristin Shrader-Frechette	**Reported:** 01/25/2021

Standard Traceability

2203A Commerce Road, Suite 1
Forest Hill, MD 21050 USA
1.410.838.8780

CERTIFICATE OF ANALYSIS

**University of Notre Dame**	**Site Name:** So-Cal Military Toxic Site	**Beacon Proposal:** 201201H01
Department of Biological Sciences	**Site Location:** Notspa, CA	**Lab Work Order:** 0005542
South Bend, IN 46556	**Project Manager:** Kristin Shrader-Frechette	**Reported:** 01/25/2021

Stock Standard Certificates of Analysis

2203A Commerce Road, Suite 1
Forest Hill, MD 21050 USA
1.410.838.8780

BEACON ENVIRONMENTAL

CERTIFICATE OF ANALYSIS

**University of Notre Dame**	**Site Name:** So-Cal Military Toxic Site	**Beacon Proposal:** 201201H01
Department of Biological Sciences	**Site Location:** Notspa, CA	**Lab Work Order:** 0005542
South Bend, IN 46556	**Project Manager:** Kristin Shrader-Frechette	**Reported:** 01/25/2021

Vendor: Absolute Standards, Inc. **Lab Standard No.:** 1800015

Lot No.: 080117

Absolute Standards, Inc.
800-368-1131
www.absolutestandards.com

Certified Reference Material CRM

ISO 17034 Accredited
Scopes: http://AbsoluteStandards.com

CERTIFIED WEIGHT REPORT

**Part Number:**	19167	**Solvent(s):**	**Lot#**
Lot Number:	112514	Methanol	DK793
Description:	CLP - Instrument Performance Check Solution<br>p-Bromofluorobenzene		
Expiration Date:	112520		
Recommended Storage:	Refrigerate (4 °C)		
Nominal Concentration (µg/mL):	2500		
NIST Test ID#:	732/245790	5E-05	Balance Uncertainty
Weight(s) shown below were combined and diluted to (mL):	100.0	0.006	Flask Uncertainty

Formulated By:	Paul Barron	112514 DATE
Reviewed By:	Pedro L. Rentas	112514 DATE

Compound	RM#	Lot Number	Nominal Conc (µg/mL)	Purity (%)	Uncertainty Purity	Target Weight(g)	Actual Weight(g)	Actual Conc (µg/mL)	Expanded Uncertainty (+/-) (µg/mL)	CAS#	OSHA PEL (TWA)	LD50
1. p-Bromofluorobenzene	48	01127COV	2500	99	0.2	0.25254	0.25282	2502.8	10.2	460-00-4	N/A	orl-rat 2700mg/kg

SDS Information (Solvent Safety Info. On Attached pg.)

Method GC6MSD-1: Column: Vocol (60m X 0.25mm ID X 1.5µm film thickness).Temp. 1=35°C (10min.), Temp. 2=200°C (8.75 min.), Rate=4°C/min., Injector Temp.=200°C, Detector Temp.=220°C. Solvent vent time = 6 min. Analysis performed by Candice Warren.

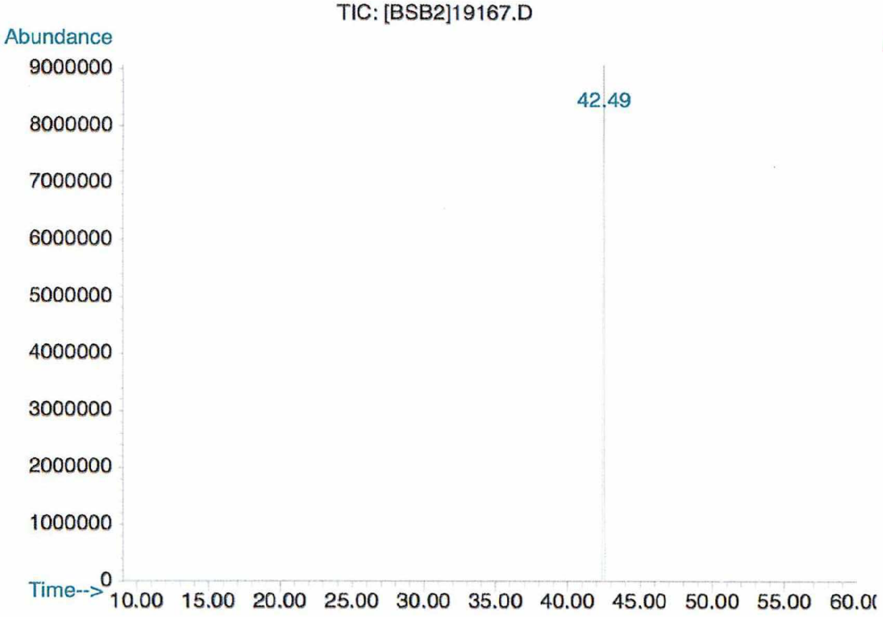

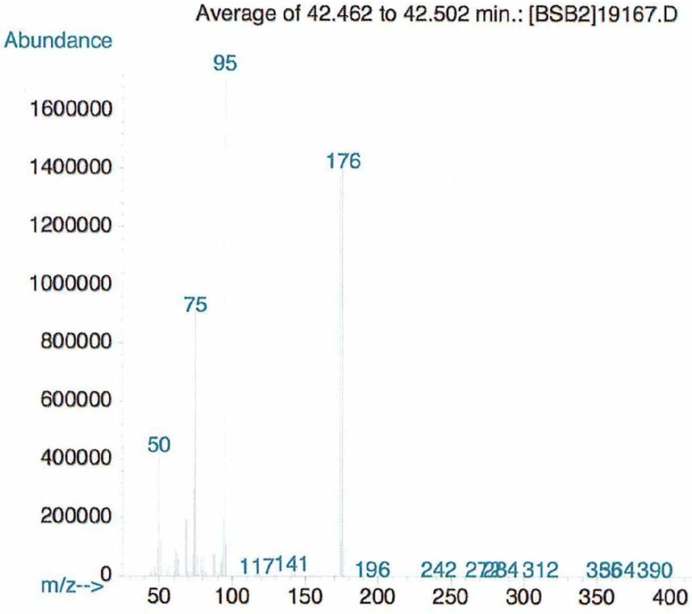

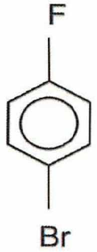

- The certified value is the concentration calculated from gravimetric and volumetric measurements unless otherwise stated.
- Standards are prepared gravimetrically using balances that are calibrated with weights traceable to NIST (see above).
- Standards are certified (+/-) 0.5% of the stated value, unless otherwise stated.
- All Standards, after opening ampule, should be stored with caps tight and under appropriate laboratory conditions.
- Uncertainty Reference: Taylor, B.N. and Kuyat, C.E., "Guidelines for Evaluating and Expressing the Uncertainty of NIST Measurement Result," NIST Technical Note 1297, U.S. Government Printing Office, Washington, DC, (1994).

Absolute Standards, Inc.
800-368-1131
www.absolutestandards.com

Certified Reference Material CRM

ISO 17034 Accredited
Scopes: http://AbsoluteStandards.com

Run 30, "P19167 L112514 [2500µg/mL in MeOH]"

Run Length: 59.99 min, 35996 points at 10 points/second.
Created: Wed, Nov 26, 2014 at 3:27:40 PM.
Sampled: Sequence "112514-GC1", Method "GC1-M7".
Analyzed using Method "GC1-M7".

Comments
GC1-M7 Analysis by Candice Warren
Column ID SPB-Vocol 105 meter X 0.53mm X 3.0µm film thickness
Flow rates: Total flow = 150mL/min., Helium (carrier) = 10mL/min.,
Helium (make-up) = 40mL/min., Hydrogen (make-up) = 100mL/min.,
Oven Profile: Temp. 1 = 35°C (Time 1 = 10 min.), Temp 2 = 200°C (Time 2 = 8.75 min.),
Rate = 4°C/min., Total run time = 60 min. Injector temp. = 200°C, PID Temp. = 200°C.
ELCD Signal = Edaq Channel 1 PID Signal = Edaq Channel 2
Standard injection = 0.5µL, Range=4 Purge Valve = 8 min

Name	PID RT (min.)
p-Bromofluorobenzene	36.19

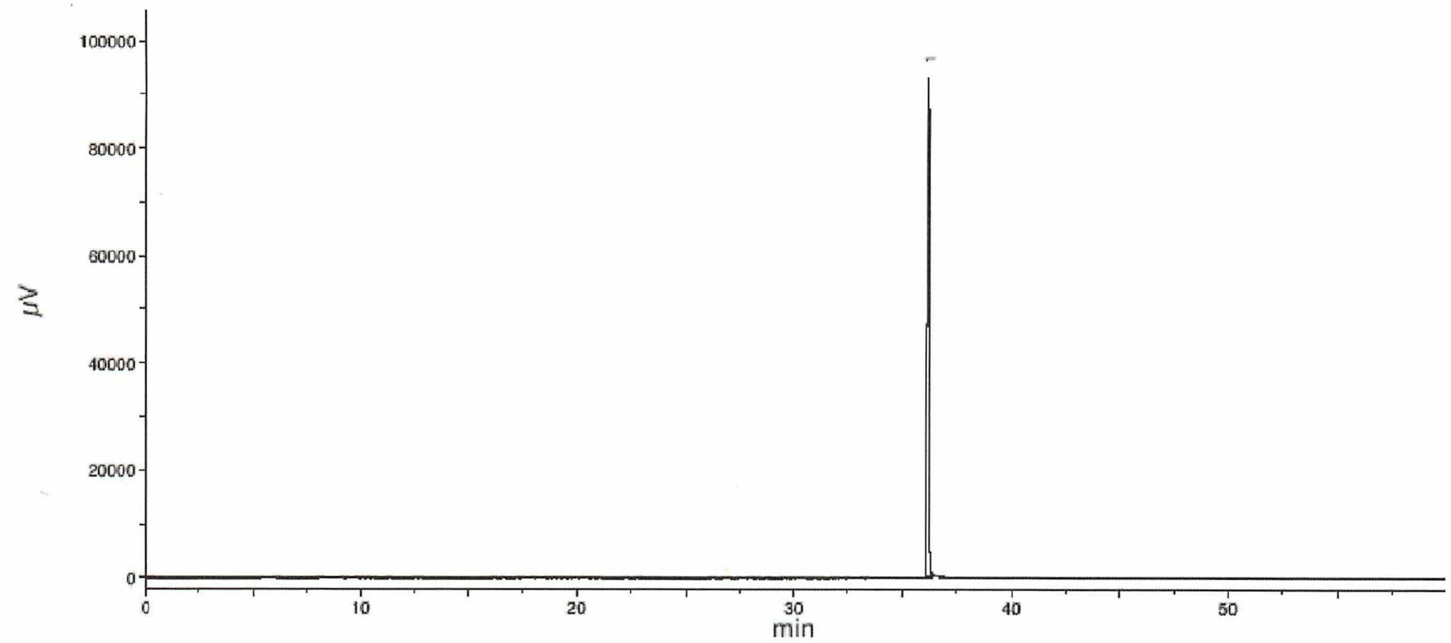

2203A Commerce Road, Suite 1
Forest Hill, MD 21050 USA
1.410.838.8780

CERTIFICATE OF ANALYSIS

**University of Notre Dame**	**Site Name:**	So-Cal Military Toxic Site	**Beacon Proposal:**	201201H01
Department of Biological Sciences	**Site Location:**	Notspa, CA	**Lab Work Order:**	0005542
South Bend, IN 46556	**Project Manager:**	Kristin Shrader-Frechette	**Reported:**	01/25/2021

Vendor: Absolute Standards, Inc. **Lab Standard No.:** 1900018

Lot No.: 112916

Absolute Standards, Inc.
800-368-1131
www.absolutestandards.com

Certified Reference Material CRM

ANAB ISO 17034 Accredited
AR-1539 Certificate Number
https://Absolutestandards.com

CERTIFIED WEIGHT REPORT

**Part Number:**	97270			
**Lot Number:**	112916			
**Description:**	8260 VOC 2nd Source Calibration Check			
	84 components			
**Expiration Date:**	112921		**Solvent(s):**	**Lot#**
**Recommended Storage:**	Freezer (0 °C)		Methanol	DP303Q9
**Nominal Concentration (µg/mL):**	100			
**NIST Test ID#:**	822-275872-11	5E-05	Balance Uncertainty	
Volume(s) shown below were combined and diluted to (mL): 100.0		0.001	Flask Uncertainty	

Formulated By:	Paul Barron	112916 DATE
Reviewed By:	Pedro L. Rentas	112916 DATE

SDS Information (Solvent Safety Info. On Attached pg.)

Compound	Part Number	Lot Number	Dil. Factor	Initial Vol. (mL)	Uncertainty Pipette	Initial Conc.(ug/mL)	Final Conc.(ug/mL)	Expanded Uncertainty (+/-) (µg/mL)	CAS#	OSHA PEL (TWA)	LD50
1. Bromodichloromethane	93538	112116	0.05	5.00	0.025	2000.1	100.0	1.5	75-27-4	N/A	orl-rat 916mg/kg
2. Dibromochloromethane	93538	112116	0.05	5.00	0.025	2000.2	100.0	1.5	124-48-1	N/A	orl-rat 848mg/kg
3. cis-1,2-Dichloroethene	93538	112116	0.05	5.00	0.025	2000.3	100.0	1.5	156-59-2	N/A	N/A
4. trans-1,2-Dichloroethene	93538	112116	0.05	5.00	0.025	2000.9	100.0	1.5	156-60-5	N/A	orl-rat 1235mg/kg
5. Methylene chloride	93538	112116	0.05	5.00	0.025	2000.2	100.0	1.5	75-09-2	500 ppm	orl-rat 2136mg/kg
6. 1,1-Dichloroethene	93538	112116	0.05	5.00	0.025	2000.2	100.0	1.1	75-35-4	1 ppm (4mg/m3/8H)	orl-rat 200mg/kg
7. Bromochloromethane	93538	112116	0.05	5.00	0.025	2000.5	100.0	1.5	74-97-5	200 ppm (1050mg/m3/8H)	orl-rat 5000mg/kg
8. Bromoform	93538	112116	0.05	5.00	0.025	2001.2	100.0	1.5	75-25-2	0.5 ppm (5mg/m3) (skin)	orl-rat 933mg/kg
9. Carbon tetrachloride	93538	112116	0.05	5.00	0.025	2000.7	100.0	1.5	56-23-5	2 ppm (12.6mg/m3/8H)	orl-rat 2350mg/kg
10. Chloroform	93538	112116	0.05	5.00	0.025	2000.1	100.0	1.5	67-66-3	50 ppm (240mg/m3) (CL)	orl-rat 908mg/kg
11. Dibromomethane	93538	112116	0.05	5.00	0.025	2000.9	100.0	1.5	74-95-3	N/A	orl-rat 108mg/kg
12. 1,1-Dichloroethane	93538	112116	0.05	5.00	0.025	2000.3	100.0	1.5	75-34-3	100 ppm	orl-rat 725mg/kg
13. 2,2-Dichloropropane	93538	112116	0.05	5.00	0.025	2000.9	100.0	1.5	594-20-7	N/A	N/A
14. Tetrachloroethene	93538	112116	0.05	5.00	0.025	2000.3	100.0	1.5	127-18-4	25 ppm (170mg/m3/8H)(final)	orl-rat 2629mg/kg
15. 1,1,1-Trichloroethane	93538	112116	0.05	5.00	0.025	2000.0	100.0	1.5	71-55-6	350 ppm (1900mg/m3/8H)	orl-rat 10300mg/kg
16. 1,2-Dibromo-3-chloropropane	93538	112116	0.05	5.00	0.025	2000.0	100.0	1.5	96-12-8	0.001 ppm	orl-rat 170mg/kg
17. 1,2-Dibromoethane	93538	112116	0.05	5.00	0.025	1999.9	100.0	1.5	106-93-4	20 ppm (8H)	orl-rat 108mg/kg
18. 1,2-Dichloroethane	93538	112116	0.05	5.00	0.025	2000.2	100.0	1.5	107-06-2	50 ppm (8H)	orl-rat 670mg/kg
19. 1,2-Dichloropropane	93538	112116	0.05	5.00	0.025	1999.9	100.0	1.5	78-87-5	75 ppm (350mg/m3/8H)	orl-rat 1947mg/kg
20. 1,3-Dichloropropane	93538	112116	0.05	5.00	0.025	2000.1	100.0	1.5	142-28-9	N/A	unr-mus 3600mg/kg
21. 1,1-Dichloropropene	93538	112116	0.05	5.00	0.025	2000.1	100.0	1.5	563-58-6	N/A	N/A
22. cis-1,3-Dichloropropene	93538	112116	0.05	5.00	0.025	2000.0	100.0	1.5	10061-01-5	N/A	N/A
23. trans-1,3-Dichloropropene	93538	112116	0.05	5.00	0.025	2000.1	100.0	1.5	10061-02-6	N/A	N/A
24. Hexachloro-1,3-butadiene	93538	112116	0.05	5.00	0.025	2000.2	100.0	1.5	87-68-3	0.02 ppm (0.24mg/m3/8H)	orl-rat 82mg/kg
25. 1,1,1,2-Tetrachloroethane	93538	112116	0.05	5.00	0.025	2000.4	100.0	1.5	630-20-6	N/A	orl-rat 670mg/kg
26. 1,1,2,2-Tetrachloroethane	93538	112116	0.05	5.00	0.025	2000.0	100.0	1.5	79-34-5	5 ppm (35mg/m3/9H)(skin)	orl-rat 800mg/kg
27. 1,1,2-Trichloroethane	93538	112116	0.05	5.00	0.025	2000.2	100.0	1.5	79-00-5	10 ppm (45mg/m3/8H)(skin)	orl-rat 836mg/kg
28. Trichloroethene	93538	112116	0.05	5.00	0.025	2000.2	100.0	1.5	79-01-6	50 ppm (270mg/m3/8H)	orl-mus 2402mg/kg
29. 1,2,3-Trichloropropane	93538	112116	0.05	5.00	0.025	2000.1	100.0	1.5	96-18-4	10 ppm (60mg/m3/8H)	orl-rat 149.6mg/kg
30. Benzene	93538	112116	0.05	5.00	0.025	2000.0	100.0	1.1	71-43-2	1 ppm	orl-rat 4894mg/kg
31. Bromobenzene	93538	112116	0.05	5.00	0.025	2000.2	100.0	1.1	108-86-1	N/A	orl-rat 2699mg/kg
32. n-Butyl benzene	93538	112116	0.05	5.00	0.025	2000.9	100.0	1.1	104-51-8	N/A	N/A
33. Ethyl benzene	93538	112116	0.05	5.00	0.025	2000.0	100.0	1.1	100-41-4	100 ppm (435mg/m3/8H)	orl-rat >2000mg/kg
34. p-Isopropyl toluene	93538	112116	0.05	5.00	0.025	2000.3	100.0	1.1	99-87-6	N/A	orl-rat 4750mg/kg
35. Naphthalene	93538	112116	0.05	5.00	0.025	2000.1	100.0	1.1	91-20-3	10 ppm (50mg/m3/8H)	orl-rat 490mg/kg
36. Toluene	93538	112116	0.05	5.00	0.025	2000.1	100.0	1.1	108-88-3	200 ppm	orl-rat 5000mg/kg
37. 1,2,3-Trichlorobenzene	93538	112116	0.05	5.00	0.025	2001.1	100.0	1.1	87-61-6	N/A	ipr-mus 1390mg/kg
38. 1,2,4-Trichlorobenzene	93538	112116	0.05	5.00	0.025	2000.7	100.0	1.1	120-82-1	5 ppm (CL) (40mg/m3)	orl-rat 756mg/kg
39. 1,2,4-Trimethylbenzene	93538	112116	0.05	5.00	0.025	2000.6	100.0	1.1	95-63-6	N/A	orl-rat 5g/kg
40. 1,3,5-Trimethylbenzene	93538	112116	0.05	5.00	0.025	2000.4	100.0	1.1	108-67-8	N/A	N/A
41. Styrene	93538	112116	0.05	5.00	0.025	2001.4	100.1	1.1	100-42-5	100 ppm	orl-rat 5000mg/kg
42. tert-Butyl benzene	93538	112116	0.05	5.00	0.025	2000.2	100.0	1.1	98-06-6	N/A	N/A
43. sec-Butyl benzene	93538	112116	0.05	5.00	0.025	2000.4	100.0	1.1	135-98-8	N/A	orl-rat 2240mg/kg
44. Chlorobenzene	93538	112116	0.05	5.00	0.025	2000.6	100.0	1.1	108-90-7	75 ppm (350mg/m3/8H)	orl-rat 2290mg/kg
45. 2-Chlorotoluene	93538	112116	0.05	5.00	0.025	2000.1	100.0	1.1	95-49-8	50 ppm (250mg/m3/8H)	orl-rat 3900mg/kg
46. 4-Chlorotoluene	93538	112116	0.05	5.00	0.025	2000.3	100.0	1.1	106-43-4	N/A	orl-rat 2100mg/kg
47. 1,2-Dichlorobenzene	93538	112116	0.05	5.00	0.025	2000.6	100.0	1.1	95-50-1	50 ppm (300mg/m3) (CL)	orl-rat 500mg/kg
48. 1,3-Dichlorobenzene	93538	112116	0.05	5.00	0.025	2000.5	100.0	1.1	541-73-1	N/A	ipr-mus 1062mg/kg
49. 1,4-Dichlorobenzene	93538	112116	0.05	5.00	0.025	2000.3	100.0	1.1	106-46-7	75 ppm (450mg/m3/8H)	orl-rat 500mg/kg
50. Isopropylbenzene	93538	112116	0.05	5.00	0.025	2000.6	100.0	1.1	98-82-8	50 ppm (245mg/m3/8H)	orl-rat 1400mg/kg
51. n-Propylbenzene	93538	112116	0.05	5.00	0.025	2000.4	100.0	1.1	103-65-1	N/A	orl-rat 6040mg/kg
52. o-Xylene	93538	112116	0.05	5.00	0.025	2000.0	100.0	1.1	95-47-6	100 ppm (435mg/m3/8H)	ipr-mus 1364mg/kg
53. m-Xylene	93538	112116	0.05	5.00	0.025	1000.1	**50.0**	0.7	108-38-3	100 ppm (435mg/m3/8H)	orl-rat 5g/kg
54. p-Xylene	93538	112116	0.05	5.00	0.025	1001.1	**50.1**	0.7	106-42-3	100 ppm (435mg/m3/8H)	orl-rat 5g/kg
55. Carbon disulphide	97269	102412	0.005	0.50	0.002	20013.3	100.1	1.0	75-15-0	4 ppm (12mg/m3) (skin)	orl-rat 1200mg/kg
56. 1,4-Dioxane	97269	102412	0.005	0.50	0.002	20006.8	100.0	1.0	123-91-1	25 ppm (90mg/m3/8H)(skin)	orl-mus 5700mg/kg
[illegible]xachloroethane	97269	102412	0.005	0.50	0.002	20003.5	100.0	1.0	67-72-1	1 ppm (10mg/m3/8H)(skin)	orl-gpg 4970mg/kg
[illegible]thyl tert-butyl ether (MTBE)	97269	102412	0.005	0.50	0.002	20022.8	100.1	1.0	1634-04-4	N/A	orl-rat 4g/kg
[illegible]Methylnaphthalene	97269	102412	0.005	0.50	0.002	20001.6	100.0	1.0	91-57-6	N/A	orl-rat 1630mg/kg
[illegible],2-Trichloro-1,2,2-trifluoroethane	97269	102412	0.005	0.50	0.002	20025.7	100.1	1.0	76-13-1	1000 ppm (7600mg/m3/8H)	orl-rat 43g/kg
[illegible]Pentane	97235	120114	0.05	5.00	0.025	2002.6	100.1	1.1	109-66-0	600 ppm(1800mg/m3/8H)	ivn-mus 446mg/kg
[illegible]Hexane	97235	120114	0.05	5.00	0.025	2002.6	100.1	1.1	110-54-3	50 ppm(180mg/m3/8H)	orl-rat 28710mg/kg
[illegible]Heptane	97235	120114	0.05	5.00	0.025	2003.1	100.1	1.1	142-82-5	400 ppm(1600mg/m3/8H)	ivn-mus 222mg/kg
[illegible]Octane	97235	120114	0.05	5.00	0.025	2001.6	100.1	1.1	111-65-9	300 ppm(1450mg/m3/8H)	N/A
[illegible]Nonane	97235	120114	0.05	5.00	0.025	2000.7	100.0	1.1	111-84-2	200 ppm (1050mg/m3/8H)	ivn-mus 218mg/kg
[illegible]Decane	97235	120114	0.05	5.00	0.025	2001.8	100.1	1.1	124-18-5	N/A	N/A

Page 90 of 329

Absolute Standards, Inc.
800-368-1131
www.absolutestandards.com

Certified Reference Material CRM

ANAB ISO 17034 Accredited
AR-1539 Certificate Number
https://Absolutestandards.com

67.	n-Undecane	97235	120114	0.05	5.00	0.025	2003.6	100.2	1.1	1120-21-4	N/A	ivn-mus 517mg/kg
68.	n-Dodecane	97235	120114	0.05	5.00	0.025	2000.6	100.0	1.1	112-40-3	N/A	N/A
69.	n-Tridecane	97235	120114	0.05	5.00	0.025	2000.2	100.0	1.1	629-50-5	N/A	ivn-mus 1161mg/kg
70.	n-Tetradecane	97235	120114	0.05	5.00	0.025	2001.5	100.1	1.1	629-59-4	N/A	N/A
71.	n-Pentadecane	97235	120114	0.05	5.00	0.025	2001.3	100.1	1.1	629-62-9	N/A	ivn-mus 3494mg/kg
72.	Bromomethane	30058	103116	0.05	5.00	0.025	2004.4	100.2	1.1	74-83-9	5 ppm (20mg/m3/8H) (skin)	orl-rat 214mg/kg
73.	Chloroethane	30058	103116	0.05	5.00	0.025	2003.4	100.2	1.1	75-00-3	1000 ppm (2600mg/m3/8H)	N/A
74.	Chloromethane	30058	103116	0.05	5.00	0.025	2003.4	100.2	1.1	74-87-3	100 ppm	orl-rat 1800mg/kg
75.	Dichlorodifluoromethane	30058	103116	0.05	5.00	0.025	2000.7	100.0	1.1	75-71-8	1000 ppm (4950mg/m3/8H)	N/A
76.	Trichlorofluoromethane	30058	103116	0.05	5.00	0.025	2001.7	100.1	1.1	75-69-4	1000 ppm (5600mg/m3/8H)	ipr-mus 1743mg/kg
77.	Vinyl chloride	30058	103116	0.05	5.00	0.025	2010.4	100.5	1.1	75-01-4	N/A	N/A
78.	4-Methyl-2-pentanone (MIBK)	82442	112816	0.00	0.50	0.002	20003.5	100.0	1.0	108-10-1	100 ppm (410mg/m3/8H)	orl-rat 2080mg/kg
79.	p-Bromofluorobenzene	20002	112816	0.05	5.00	0.025	2000.5	100.0	1.1	460-00-4	N/A	orl-rat 2700mg/kg
80.	1,2-Dichloroethane-d4	20002	112816	0.05	5.00	0.025	2001.8	100.1	1.1	17060-07-0	N/A	orl-mus 625mg/kg
81.	Toluene-d8	20002	112816	0.05	5.00	0.025	2002.1	100.1	1.1	2037-26-5	200 ppm	orl-rat 5000mg/kg
82.	Chlorobenzene-d5	22013	112816	0.10	10.00	0.006	2001.9	**200.2**	0.8	3114-55-4	N/A	orl-rat 1110mg/kg
83.	1,4-Dichlorobenzene-d4	22013	112816	0.10	10.00	0.006	2002.1	**200.2**	0.9	3855-82-1	N/A	orl-rat 500mg/kg
84.	Fluorobenzene	22013	112816	0.10	10.00	0.006	2000.5	**200.0**	0.9	462-06-6	N/A	orl-rat 4399mg/kg

- The certified value is the concentration calculated from gravimetric and volumetric measurements unless otherwise stated.
- Standards are prepared gravimetrically using balances that are calibrated with weights traceable to NIST (see above).
- Standards are certifed (+/-) 0.5% of the stated value, unless otherwise stated.
- All Standards, after opening ampule, should be stored with caps tight and under appropriate laboratory conditions.
- Uncertainty Reference: Taylor, B.N. and Kuyat, C.E., "Guidelines for Evaluating and Expressing the Uncertainty of NIST Measurement Result," NIST Technical Note 1297, U.S. Government Printing Office, Washington, DC, (1994).

2203A Commerce Road, Suite 1
Forest Hill, MD 21050 USA
1.410.838.8780

CERTIFICATE OF ANALYSIS

**University of Notre Dame**	**Site Name:** So-Cal Military Toxic Site	**Beacon Proposal:** 201201H01
Department of Biological Sciences	**Site Location:** Notspa, CA	**Lab Work Order:** 0005542
South Bend, IN 46556	**Project Manager:** Kristin Shrader-Frechette	**Reported:** 01/25/2021

Vendor: Absolute Standards, Inc. **Lab Standard No.:** 1900019

Lot No.: 041218

Absolute Standards, Inc.
800-368-1131
www.absolutestandards.com

Analytical Reference Material ARM

ISO 9001 QS Registered
ISO 17025-34-35-43 Accredited
Scopes: http://AbsoluteStandards.com

CERTIFIED WEIGHT REPORT

**Part Number:**	92026
Lot Number:	041218
Description:	EPA Method 8260B Internal & Surrogate Standard
	7 components
Expiration Date:	041223
Recommended Storage:	Refrigerate (4 °C)
Nominal Concentration (µg/mL):	2000
NIST Test ID#:	2506734D

**Solvent(s):**	**Lot#**
Methanol	DS435

5E-05 Balance Uncertainty
0.001 Flask Uncertainty

Weight(s) shown below were combined and diluted to (mL): 100.0

Formulated By:	Jason Criscio
041218	DATE
Reviewed By:	Pedro L. Rentas
041218	DATE

SDS Information (Solvent Safety Info. On Attached pg.)

	Compound	RM#	Lot Number	Nominal Conc (µg/mL)	Purity (%)	Uncertainty Purity	Target Weight(g)	Actual Weight(g)	Actual Conc (µg/mL)	Expanded Uncertainty (+/-) (µg/mL)	CAS#	OSHA PEL (TWA)	LD50
1.	Chlorobenzene-d5	69	PR-23926/062912CB1	2000	99.7	0.2	0.20062	0.20085	2002.3	8.1	3114-55-4	N/A	orl-rat 1110mg/kg
2.	1,4-Dichlorobenzene-d4	118	PR-18488/07267CB1	2000	98	0.2	0.20410	0.20429	2001.8	8.2	3855-82-1	N/A	orl-rat 500mg/kg
3.	Fluorobenzene	186	16006LH	2000	99	0.2	0.20204	0.20220	2001.6	8.1	462-06-6	N/A	orl-rat 4399mg/kg
4.	p-Bromofluorobenzene	48	01127COV	2000	99	0.2	0.20204	0.20221	2001.7	8.1	460-00-4	N/A	orl-rat 2700mg/kg
5.	1,4-Difluorobenzene	155	13105AO	2000	99	0.2	0.20204	0.20224	2002.0	8.1	540-36-3	N/A	N/A
6.	1,2-Dichloroethane-d4	137	V288P25	2000	99.6	0.2	0.20083	0.20095	2001.2	8.1	17060-07-0	N/A	orl-mus 625mg/kg
7.	Toluene-d8	282	PR-27981/120116TL1	2000	99.5	0.2	0.20103	0.20120	2001.7	8.1	2037-26-5	200 ppm	orl-rat 5000mg/kg

- **The certified value is the concentration calculated from gravimetric and volumetric measurements unless otherwise stated.**
- **Standards are prepared gravimetrically using balances that are calibrated with weights traceable to NIST (see above).**
- **Standards are certifed (+/-) 0.5% of the stated value, unless otherwise stated.**
- **All Standards, after opening ampule, should be stored with caps tight and under appropriate laboratory conditions.**
- **Uncertainty Reference: Taylor, B.N. and Kuyat, C.E., "Guidelines for Evaluating and Expressing the Uncertainty of NIST Measurement Result," NIST Technical Note 1297, U.S. Government Printing Office, Washington, DC, (1994).**

Comments
GC5-M1 Analysis by Candice Warren
Column ID SPB-Vocol 105 meter X 0.53mm X 3.0µm film thickness
Flow rates: Total flow=290mL/min., Helium (carrier)=10mL/min.,
Helium(make-up)=10mL/min., Hydogen(make-up)=40mL/min., Air(make-up)=230mL/min.
Oven Profile: Temp. 1=35°C (Time 1=10 min.), Temp 2=200°C (Time 2=8.75 min.).
Rate = 4°C/min., Total run time=60 min. Injector temp.=200°C, FID Temp.=200°C.
FID Signal = Edaq Channel 1
Standard injection = 0.5µL, Range=3

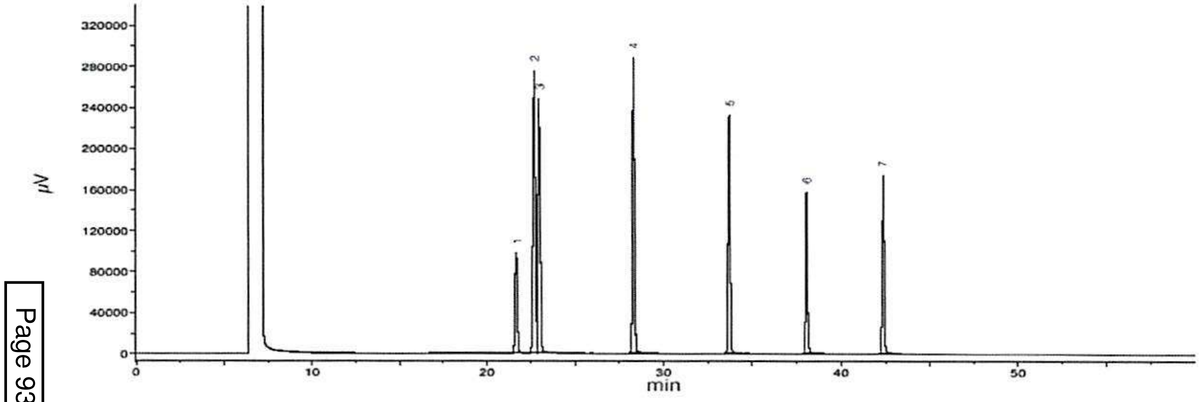

Name	FID (min.)
1,2-Dichloroethane-d4	21.67
Fluorobenzene	22.70
1,4-Difluorobenzene	22.98
Toluene-d8	28.30
Chlorobenzene-d5	33.69
p-Bromofluorobenzene	38.06
1,4-Dichlorobenzene-d4	42.36

2203A Commerce Road, Suite 1
Forest Hill, MD 21050 USA
1.410.838.8780

CERTIFICATE OF ANALYSIS

**University of Notre Dame**	**Site Name:** So-Cal Military Toxic Site	**Beacon Proposal:** 201201H01
Department of Biological Sciences	**Site Location:** Notspa, CA	**Lab Work Order:** 0005542
South Bend, IN 46556	**Project Manager:** Kristin Shrader-Frechette	**Reported:** 01/25/2021

Vendor: Absolute Standards, Inc. **Lab Standard No.:** 1900037

Lot No.: 112916

Absolute Standards, Inc.
800-368-1131
www.absolutestandards.com

Certified Reference Material CRM

ANAB ISO 17034 Accredited
AR-1539 Certificate Number
https://Absolutestandards.com

CERTIFIED WEIGHT REPORT

**Part Number:**	97270		
**Lot Number:**	112916		
**Description:**	8260 VOC 2nd Source Calibration Check		
	84 components		
**Expiration Date:**	112921	**Solvent(s):** Methanol	**Lot#** DP303Q9
**Recommended Storage:**	Freezer (0 °C)		
**Nominal Concentration (µg/mL):**	100		
**NIST Test ID#:**	822-275872-11	5E-05	Balance Uncertainty
Volume(s) shown below were combined and diluted to (mL):	100.0	0.001	Flask Uncertainty

Formulated By:	Paul Barron	112916 DATE
Reviewed By:	Pedro L. Rentas	112916 DATE

SDS Information (Solvent Safety Info. On Attached pg.)

	Compound	Part Number	Lot Number	Dil. Factor	Initial Vol. (mL)	Uncertainty Pipette	Initial Conc.(ug/mL)	Final Conc.(ug/mL)	Expanded Uncertainty (+/-) (µg/mL)	CAS#	OSHA PEL (TWA)	LD50
1.	Bromodichloromethane	93538	112116	0.05	5.00	0.025	2000.1	100.0	1.5	75-27-4	N/A	orl-rat 916mg/kg
2.	Dibromochloromethane	93538	112116	0.05	5.00	0.025	2000.2	100.0	1.5	124-48-1	N/A	orl-rat 848mg/kg
3.	cis-1,2-Dichloroethene	93538	112116	0.05	5.00	0.025	2000.3	100.0	1.5	156-59-2	N/A	N/A
4.	trans-1,2-Dichloroethene	93538	112116	0.05	5.00	0.025	2000.9	100.0	1.5	156-60-5	N/A	orl-rat 1235mg/kg
5.	Methylene chloride	93538	112116	0.05	5.00	0.025	2000.2	100.0	1.5	75-09-2	500 ppm	orl-rat 2136mg/kg
6.	1,1-Dichloroethene	93538	112116	0.05	5.00	0.025	2000.2	100.0	1.1	75-35-4	1 ppm (4mg/m3/8H)	orl-rat 200mg/kg
7.	Bromochloromethane	93538	112116	0.05	5.00	0.025	2000.5	100.0	1.5	74-97-5	200 ppm (1050mg/m3/8H)	orl-rat 5000mg/kg
8.	Bromoform	93538	112116	0.05	5.00	0.025	2001.2	100.0	1.5	75-25-2	0.5 ppm (5mg/m3) (skin)	orl-rat 933mg/kg
9.	Carbon tetrachloride	93538	112116	0.05	5.00	0.025	2000.7	100.0	1.5	56-23-5	2 ppm (12.6mg/m3/8H)	orl-rat 2350mg/kg
10.	Chloroform	93538	112116	0.05	5.00	0.025	2000.1	100.0	1.5	67-66-3	50 ppm (240mg/m3) (CL)	orl-rat 908mg/kg
11.	Dibromomethane	93538	112116	0.05	5.00	0.025	2000.9	100.0	1.5	74-95-3	N/A	orl-rat 108mg/kg
12.	1,1-Dichloroethane	93538	112116	0.05	5.00	0.025	2000.3	100.0	1.5	75-34-3	100 ppm	orl-rat 725mg/kg
13.	2,2-Dichloropropane	93538	112116	0.05	5.00	0.025	2000.9	100.0	1.5	594-20-7	N/A	N/A
14.	Tetrachloroethene	93538	112116	0.05	5.00	0.025	2000.3	100.0	1.5	127-18-4	25 ppm (170mg/m3/8H)(final)	orl-rat 2629mg/kg
15.	1,1,1-Trichloroethane	93538	112116	0.05	5.00	0.025	2000.0	100.0	1.5	71-55-6	350 ppm (1900mg/m3/8H)	orl-rat 10300mg/kg
16.	1,2-Dibromo-3-chloropropane	93538	112116	0.05	5.00	0.025	2000.0	100.0	1.5	96-12-8	0.001 ppm	orl-rat 170mg/kg
17.	1,2-Dibromoethane	93538	112116	0.05	5.00	0.025	1999.9	100.0	1.5	106-93-4	20 ppm (8H)	orl-rat 108mg/kg
18.	1,2-Dichloroethane	93538	112116	0.05	5.00	0.025	2000.2	100.0	1.5	107-06-2	50 ppm (8H)	orl-rat 670mg/kg
19.	1,2-Dichloropropane	93538	112116	0.05	5.00	0.025	1999.9	100.0	1.5	78-87-5	75 ppm (350mg/m3/8H)	orl-rat 1947mg/kg
20.	1,3-Dichloropropane	93538	112116	0.05	5.00	0.025	2000.1	100.0	1.5	142-28-9	N/A	unr-mus 3600mg/kg
21.	1,1-Dichloropropene	93538	112116	0.05	5.00	0.025	2000.1	100.0	1.5	563-58-6	N/A	N/A
22.	cis-1,3-Dichloropropene	93538	112116	0.05	5.00	0.025	2000.0	100.0	1.5	10061-01-5	N/A	N/A
23.	trans-1,3-Dichloropropene	93538	112116	0.05	5.00	0.025	2000.1	100.0	1.5	10061-02-6	N/A	N/A
24.	Hexachloro-1,3-butadiene	93538	112116	0.05	5.00	0.025	2000.2	100.0	1.5	87-68-3	0.02 ppm (0.24mg/m3/8H)	orl-rat 82mg/kg
25.	1,1,1,2-Tetrachloroethane	93538	112116	0.05	5.00	0.025	2000.4	100.0	1.5	630-20-6	N/A	orl-rat 670mg/kg
26.	1,1,2,2-Tetrachloroethane	93538	112116	0.05	5.00	0.025	2000.0	100.0	1.5	79-34-5	5 ppm (35mg/m3/9H)(skin)	orl-rat 800mg/kg
27.	1,1,2-Trichloroethane	93538	112116	0.05	5.00	0.025	2000.2	100.0	1.5	79-00-5	10 ppm (45mg/m3/8H)(skin)	orl-rat 836mg/kg
28.	Trichloroethene	93538	112116	0.05	5.00	0.025	2000.2	100.0	1.5	79-01-6	50 ppm (270mg/m3/8H)	orl-mus 2402mg/kg
29.	1,2,3-Trichloropropane	93538	112116	0.05	5.00	0.025	2000.1	100.0	1.5	96-18-4	10 ppm (60mg/m3/8H)	orl-rat 149.6mg/kg
30.	Benzene	93538	112116	0.05	5.00	0.025	2000.0	100.0	1.1	71-43-2	1 ppm	orl-rat 4894mg/kg
31.	Bromobenzene	93538	112116	0.05	5.00	0.025	2000.2	100.0	1.1	108-86-1	N/A	orl-rat 2699mg/kg
32.	n-Butyl benzene	93538	112116	0.05	5.00	0.025	2000.9	100.0	1.1	104-51-8	N/A	N/A
33.	Ethyl benzene	93538	112116	0.05	5.00	0.025	2000.0	100.0	1.1	100-41-4	100 ppm (435mg/m3/8H)	orl-rat >2000mg/kg
34.	p-Isopropyl toluene	93538	112116	0.05	5.00	0.025	2000.3	100.0	1.1	99-87-6	N/A	orl-rat 4750mg/kg
35.	Naphthalene	93538	112116	0.05	5.00	0.025	2000.1	100.0	1.1	91-20-3	10 ppm (50mg/m3/8H)	orl-rat 490mg/kg
36.	Toluene	93538	112116	0.05	5.00	0.025	2000.1	100.0	1.1	108-88-3	200 ppm	orl-rat 5000mg/kg
37.	1,2,3-Trichlorobenzene	93538	112116	0.05	5.00	0.025	2001.1	100.0	1.1	87-61-6	N/A	ipr-mus 1390mg/kg
38.	1,2,4-Trichlorobenzene	93538	112116	0.05	5.00	0.025	2000.7	100.0	1.1	120-82-1	5 ppm (CL) (40mg/m3)	orl-rat 756mg/kg
39.	1,2,4-Trimethylbenzene	93538	112116	0.05	5.00	0.025	2000.6	100.0	1.1	95-63-6	N/A	orl-rat 5g/kg
40.	1,3,5-Trimethylbenzene	93538	112116	0.05	5.00	0.025	2000.4	100.0	1.1	108-67-8	N/A	N/A
41.	Styrene	93538	112116	0.05	5.00	0.025	2001.4	100.1	1.1	100-42-5	100 ppm	orl-rat 5000mg/kg
42.	tert-Butyl benzene	93538	112116	0.05	5.00	0.025	2000.2	100.0	1.1	98-06-6	N/A	N/A
43.	sec-Butyl benzene	93538	112116	0.05	5.00	0.025	2000.4	100.0	1.1	135-98-8	N/A	orl-rat 2240mg/kg
44.	Chlorobenzene	93538	112116	0.05	5.00	0.025	2000.6	100.0	1.1	108-90-7	75 ppm (350mg/m3/8H)	orl-rat 2290mg/kg
45.	2-Chlorotoluene	93538	112116	0.05	5.00	0.025	2000.1	100.0	1.1	95-49-8	50 ppm (250mg/m3/8H)	orl-rat 3900mg/kg
46.	4-Chlorotoluene	93538	112116	0.05	5.00	0.025	2000.3	100.0	1.1	106-43-4	N/A	orl-rat 2100mg/kg
47.	1,2-Dichlorobenzene	93538	112116	0.05	5.00	0.025	2000.6	100.0	1.1	95-50-1	50 ppm (300mg/m3) (CL)	orl-rat 500mg/kg
48.	1,3-Dichlorobenzene	93538	112116	0.05	5.00	0.025	2000.5	100.0	1.1	541-73-1	N/A	ipr-mus 1062mg/kg
49.	1,4-Dichlorobenzene	93538	112116	0.05	5.00	0.025	2000.3	100.0	1.1	106-46-7	75 ppm (450mg/m3/8H)	orl-rat 500mg/kg
50.	Isopropylbenzene	93538	112116	0.05	5.00	0.025	2000.6	100.0	1.1	98-82-8	50 ppm (245mg/m3/8H)	orl-rat 1400mg/kg
51.	n-Propylbenzene	93538	112116	0.05	5.00	0.025	2000.4	100.0	1.1	103-65-1	N/A	orl-rat 6040mg/kg
52.	o-Xylene	93538	112116	0.05	5.00	0.025	2000.0	100.0	1.1	95-47-6	100 ppm (435mg/m3/8H)	ipr-mus 1364mg/kg
53.	m-Xylene	93538	112116	0.05	5.00	0.025	1000.1	**50.0**	0.7	108-38-3	100 ppm (435mg/m3/8H)	orl-rat 5g/kg
54.	p-Xylene	93538	112116	0.05	5.00	0.025	1001.1	**50.1**	0.7	106-42-3	100 ppm (435mg/m3/8H)	orl-rat 5g/kg
55.	Carbon disulphide	97269	102412	0.005	0.50	0.002	20013.3	100.1	1.0	75-15-0	4 ppm (12mg/m3) (skin)	orl-rat 1200mg/kg
56.	1,4-Dioxane	97269	102412	0.005	0.50	0.002	20006.8	100.0	1.0	123-91-1	25 ppm (90mg/m3/8H)(skin)	orl-mus 5700mg/kg
[illegible]	[illegible]exachloroethane	97269	102412	0.005	0.50	0.002	20003.5	100.0	1.0	67-72-1	1 ppm (10mg/m3/8H)(skin)	orl-gpg 4970mg/kg
[illegible]	[illegible]ethyl tert-butyl ether (MTBE)	97269	102412	0.005	0.50	0.002	20022.8	100.1	1.0	1634-04-4	N/A	orl-rat 4g/kg
[illegible]	[illegible]Methylnaphthalene	97269	102412	0.005	0.50	0.002	20001.6	100.0	1.0	91-57-6	N/A	orl-rat 1630mg/kg
[illegible]	[illegible],2-Trichloro-1,2,2-trifluoroethane	97269	102412	0.005	0.50	0.002	20025.7	100.1	1.0	76-13-1	1000 ppm (7600mg/m3/8H)	orl-rat 43g/kg
[illegible]	[illegible]Pentane	97235	120114	0.05	5.00	0.025	2002.6	100.1	1.1	109-66-0	600 ppm(1800mg/m3/8H)	ivn-mus 446mg/kg
[illegible]	[illegible]Hexane	97235	120114	0.05	5.00	0.025	2002.6	100.1	1.1	110-54-3	50 ppm(180mg/m3/8H)	orl-rat 28710mg/kg
[illegible]	[illegible]Heptane	97235	120114	0.05	5.00	0.025	2003.1	100.1	1.1	142-82-5	400 ppm(1600mg/m3/8H)	ivn-mus 222mg/kg
[illegible]	[illegible]Octane	97235	120114	0.05	5.00	0.025	2001.6	100.1	1.1	111-65-9	300 ppm(1450mg/m3/8H)	N/A
[illegible]	[illegible]Nonane	97235	120114	0.05	5.00	0.025	2000.7	100.0	1.1	111-84-2	200 ppm (1050mg/m3/8H)	ivn-mus 218mg/kg
[illegible]	[illegible]Decane	97235	120114	0.05	5.00	0.025	2001.8	100.1	1.1	124-18-5	N/A	N/A

Absolute Standards, Inc.
800-368-1131
www.absolutestandards.com

Certified Reference Material CRM

ANAB ISO 17034 Accredited
AR-1539 Certificate Number
https://Absolutestandards.com

67.	n-Undecane	97235	120114	0.05	5.00	0.025	2003.6	100.2	1.1	1120-21-4	N/A	ivn-mus 517mg/kg
68.	n-Dodecane	97235	120114	0.05	5.00	0.025	2000.6	100.0	1.1	112-40-3	N/A	N/A
69.	n-Tridecane	97235	120114	0.05	5.00	0.025	2000.2	100.0	1.1	629-50-5	N/A	ivn-mus 1161mg/kg
70.	n-Tetradecane	97235	120114	0.05	5.00	0.025	2001.5	100.1	1.1	629-59-4	N/A	N/A
71.	n-Pentadecane	97235	120114	0.05	5.00	0.025	2001.3	100.1	1.1	629-62-9	N/A	ivn-mus 3494mg/kg
72.	Bromomethane	30058	103116	0.05	5.00	0.025	2004.4	100.2	1.1	74-83-9	5 ppm (20mg/m3/8H) (skin)	orl-rat 214mg/kg
73.	Chloroethane	30058	103116	0.05	5.00	0.025	2003.4	100.2	1.1	75-00-3	1000 ppm (2600mg/m3/8H)	N/A
74.	Chloromethane	30058	103116	0.05	5.00	0.025	2003.4	100.2	1.1	74-87-3	100 ppm	orl-rat 1800mg/kg
75.	Dichlorodifluoromethane	30058	103116	0.05	5.00	0.025	2000.7	100.0	1.1	75-71-8	1000 ppm (4950mg/m3/8H)	N/A
76.	Trichlorofluoromethane	30058	103116	0.05	5.00	0.025	2001.7	100.1	1.1	75-69-4	1000 ppm (5600mg/m3/8H)	ipr-mus 1743mg/kg
77.	Vinyl chloride	30058	103116	0.05	5.00	0.025	2010.4	100.5	1.1	75-01-4	N/A	N/A
78.	4-Methyl-2-pentanone (MIBK)	82442	112816	0.00	0.50	0.002	20003.5	100.0	1.0	108-10-1	100 ppm (410mg/m3/8H)	orl-rat 2080mg/kg
79.	p-Bromofluorobenzene	20002	112816	0.05	5.00	0.025	2000.5	100.0	1.1	460-00-4	N/A	orl-rat 2700mg/kg
80.	1,2-Dichloroethane-d4	20002	112816	0.05	5.00	0.025	2001.8	100.1	1.1	17060-07-0	N/A	orl-mus 625mg/kg
81.	Toluene-d8	20002	112816	0.05	5.00	0.025	2002.1	100.1	1.1	2037-26-5	200 ppm	orl-rat 5000mg/kg
82.	Chlorobenzene-d5	22013	112816	0.10	10.00	0.006	2001.9	**200.2**	0.8	3114-55-4	N/A	orl-rat 1110mg/kg
83.	1,4-Dichlorobenzene-d4	22013	112816	0.10	10.00	0.006	2002.1	**200.2**	0.9	3855-82-1	N/A	orl-rat 500mg/kg
84.	Fluorobenzene	22013	112816	0.10	10.00	0.006	2000.5	**200.0**	0.9	462-06-6	N/A	orl-rat 4399mg/kg

- **The certified value is the concentration calculated from gravimetric and volumetric measurements unless otherwise stated.**
- **Standards are prepared gravimetrically using balances that are calibrated with weights traceable to NIST (see above).**
- **Standards are certifed (+/-) 0.5% of the stated value, unless otherwise stated.**
- **All Standards, after opening ampule, should be stored with caps tight and under appropriate laboratory conditions.**
- **Uncertainty Reference: Taylor, B.N. and Kuyat, C.E., "Guidelines for Evaluating and Expressing the Uncertainty of NIST Measurement Result," NIST Technical Note 1297, U.S. Government Printing Office, Washington, DC, (1994).**

2203A Commerce Road, Suite 1
Forest Hill, MD 21050 USA
1.410.838.8780

CERTIFICATE OF ANALYSIS

**University of Notre Dame**		**Site Name:**	So-Cal Military Toxic Site	**Beacon Proposal:**	201201H01
Department of Biological Sciences		**Site Location:**	Notspa, CA	**Lab Work Order:**	0005542
South Bend, IN 46556		**Project Manager:**	Kristin Shrader-Frechette	**Reported:**	01/25/2021

Vendor: Absolute Standards, Inc. **Lab Standard No.:** 1900054
Lot No.: 061019

Absolute Standards, Inc.
800-368-1131
www.absolutestandards.com

Certified Reference Material CRM

ISO 17034 Accredited
Scopes: http://AbsoluteStandards.com

CERTIFIED WEIGHT REPORT

Part Number: 64090
Lot Number: 061019
Description: VOC Calibration Level 1 (800 ug/mL)
42 components
Expiration Date: 061022
Recommended Storage: Freezer (0 °C)
Nominal Concentration (µg/mL): Varied
NIST Test ID#: 6UTB

Solvent: Methanol
Lot# DU230-USQ10

5E-05 Balance Uncertainty
0.002 Flask Uncertainty

Volume(s) shown below were combined and diluted to (mL): 20.0

Formulated By:	Gabriel Helland	061019	DATE
Reviewed By:	Pedro L. Rentas	061019	DATE

RED = Surrogate; BLUE = Internal

SDS Information (Solvent Safety Info. On Attached pg.)

	Compound	Part Number	Lot Number	Dilution Factor	Initial Vol. (mL)	Uncertainty Pipette (mL)	Initial Conc.(µg/mL)	Final Conc.(µg/mL)	Expanded Uncertainty (+/-) µg/mL	CAS#	OSHA PEL (TWA)	LD50
1.	Benzene	64088	060719	0.200	4.00	0.017	4001.1	800.8	7.5	71-43-2	1 ppm	orl-rat 4894mg/kg
2.	Carbon tetrachloride	64088	060719	0.200	4.00	0.017	4000.8	800.7	7.5	56-23-5	2 ppm (12.6mg/m3/8H)	orl-rat 2350mg/kg
3.	Chlorobenzene	64088	060719	0.200	4.00	0.017	4000.7	800.7	7.5	108-90-7	75 ppm (350mg/m3/8H)	orl-rat 2290mg/kg
4.	Chloroform	64088	060719	0.200	4.00	0.017	4000.7	800.7	7.5	67-66-3	50 ppm (240mg/m3) (CL)	orl-rat 908mg/kg
5.	1,2-Dibromoethane	64088	060719	0.200	4.00	0.017	4000.8	800.7	7.5	106-93-4	20 ppm (8H)	orl-rat 108mg/kg
6.	1,2-Dichlorobenzene	64088	060719	0.200	4.00	0.017	4001.3	800.8	7.5	95-50-1	50 ppm (300mg/m3) (CL)	orl-rat 500mg/kg
7.	1,3-Dichlorobenzene	64088	060719	0.200	4.00	0.017	4001.3	800.8	7.6	541-73-1	N/A	ipr-mus 1062mg/kg
8.	1,4-Dichlorobenzene	64088	060719	0.200	4.00	0.017	4000.6	800.7	7.5	106-46-7	75 ppm (450mg/m3/8H)	orl-rat 500mg/kg
9.	1,1-Dichloroethane	64088	060719	0.200	4.00	0.017	4001.0	800.8	7.5	75-34-3	100 ppm	orl-rat 725mg/kg
10.	1,2-Dichloroethane	64088	060719	0.200	4.00	0.017	4001.0	800.8	7.5	107-06-2	50 ppm (8H)	orl-rat 670mg/kg
11.	1,1-Dichloroethene	64088	060719	0.200	4.00	0.017	4001.3	800.8	7.5	75-35-4	1 ppm (4mg/m3/8H)	orl-rat 200mg/kg
12.	cis-1,2-Dichloroethene	64088	060719	0.200	4.00	0.017	4001.3	800.8	7.5	156-59-2	N/A	N/A
13.	trans-1,2-Dichloroethene	64088	060719	0.200	4.00	0.017	4001.3	800.8	7.6	156-60-5	N/A	orl-rat 1235mg/kg
14.	1,4-Dioxane	64088	060719	0.200	4.00	0.017	4000.3	800.6	7.5	123-91-1	25 ppm (90mg/m3/8H)(skin)	orl-mus 5700mg/kg
15.	Ethyl benzene	64088	060719	0.200	4.00	0.017	4001.4	800.9	7.5	100-41-4	100 ppm (435mg/m3/8H)	orl-rat >2000mg/kg
16.	Isopropylbenzene	64088	060719	0.200	4.00	0.017	4001.3	800.8	7.5	98-82-8	50 ppm (245mg/m3/8H)	orl-rat 1400mg/kg
17.	Methyl tert-butyl ether (MTBE)	64088	060719	0.200	4.00	0.017	4000.4	800.7	7.5	1634-04-4	N/A	orl-rat 4g/kg
18.	2-Methylnaphthalene	64088	060719	0.200	4.00	0.017	4000.4	800.7	7.6	91-57-6	N/A	orl-rat 1630mg/kg
19.	Naphthalene	64088	060719	0.200	4.00	0.017	4000.3	800.6	7.5	91-20-3	10 ppm (50mg/m3/8H)	orl-rat 490mg/kg
20.	1,1,1,2-Tetrachloroethane	64088	060719	0.200	4.00	0.017	4000.5	800.7	7.5	630-20-6	N/A	orl-rat 670mg/kg
21.	1,1,2,2-Tetrachloroethane	64088	060719	0.200	4.00	0.017	4000.5	800.7	7.5	79-34-5	5 ppm (35mg/m3/9H)(skin)	orl-rat 800mg/kg
22.	Tetrachloroethene	64088	060719	0.200	4.00	0.017	4000.8	800.7	7.5	127-18-4	25 ppm (170mg/m3/8H)(final)	orl-rat 2629mg/kg
23.	Toluene	64088	060719	0.200	4.00	0.017	4001.4	800.9	7.5	108-88-3	200 ppm	orl-rat 5000mg/kg
24.	1,2,3-Trichlorobenzene	64088	060719	0.200	4.00	0.017	4000.4	800.7	7.5	87-61-6	N/A	ipr-mus 1390mg/kg
25.	1,2,4-Trichlorobenzene	64088	060719	0.200	4.00	0.017	4000.7	800.7	7.5	120-82-1	5 ppm (CL) (40mg/m3)	orl-rat 756mg/kg
26.	1,1,1-Trichloroethane	64088	060719	0.200	4.00	0.017	4000.8	800.7	7.5	71-55-6	350 ppm (1900mg/m3/8H)	orl-rat 10300mg/kg
27.	1,1,2-Trichloroethane	64088	060719	0.200	4.00	0.017	4001.2	800.8	7.6	79-00-5	10 ppm (45mg/m3/8H)(skin)	orl-rat 836mg/kg
28.	Trichloroethene	64088	060719	0.200	4.00	0.017	4000.8	800.7	7.5	79-01-6	50 ppm (270mg/m3/8H)	orl-mus 2402mg/kg
29.	1,2,3-Trichloropropane	64088	060719	0.200	4.00	0.017	4000.3	800.6	7.5	96-18-4	10 ppm (60mg/m3/8H)	orl-rat 149.6mg/kg
30.	1,1,2-Trichlorotrifluoroethane	64088	060719	0.200	4.00	0.017	4001.3	800.8	7.5	76-13-1	1000 ppm (7600mg/m3/8H)	orl-rat 43g/kg
31.	1,2,4-Trimethylbenzene	64088	060719	0.200	4.00	0.017	4000.3	800.7	7.6	95-63-6	N/A	orl-rat 5g/kg
32.	1,3,5-Trimethylbenzene	64088	060719	0.200	4.00	0.017	4000.3	800.7	7.6	108-67-8	N/A	orl-rat 5000mg/kg
33.	o-Xylene	64088	060719	0.200	4.00	0.017	4000.6	800.7	7.5	95-47-6	100 ppm (435mg/m3/8H)	ipr-mus 1364mg/kg
34.	m-Xylene	64088	060719	0.200	4.00	0.017	2000.4	**400.4**	3.8	108-38-3	100 ppm (435mg/m3/8H)	orl-rat 5g/kg
35.	p-Xylene	64088	060719	0.200	4.00	0.017	2001.2	**400.5**	3.8	106-42-3	100 ppm (435mg/m3/8H)	orl-rat 5g/kg
36.	Vinyl chloride	33591	052119	0.040	0.80	0.004	20030.1	801.8	8.7	75-01-4	N/A	N/A
37.	p-Bromofluorobenzene	64088	060719	0.200	4.00	0.017	4001.3	800.8	7.5	460-00-4	N/A	orl-rat 2700mg/kg
38.	1,2-Dichloroethane-d4	64088	060719	0.200	4.00	0.017	4000.7	800.7	7.5	17060-07-0	N/A	orl-mus 625mg/kg
39.	Toluene-d8	64088	060719	0.200	4.00	0.017	4000.6	800.7	7.5	2037-26-5	200 ppm	orl-rat 5000mg/kg
40.	Chlorobenzene-d5	22013	022818	0.100	2.00	0.017	2000.6	200.2	3.5	3114-55-4	N/A	orl-rat 1110mg/kg
41.	1,4-Dichlorobenzene-d4	22013	022818	0.100	2.00	0.017	2000.7	200.2	3.5	3855-82-1	N/A	orl-rat 500mg/kg
42.	Fluorobenzene	22013	022818	0.100	2.00	0.017	2001.0	200.2	3.5	462-06-6	N/A	orl-rat 4399mg/kg

- The certified value is the concentration calculated from gravimetric and volumetric measurements unless otherwise stated.
- Standards are prepared gravimetrically using balances that are calibrated with weights traceable to NIST (see above).
- Standards are certifed (+/-) 0.5% of the stated value, unless otherwise stated.
- All Standards, after opening ampule, should be stored with caps tight and under appropriate laboratory conditions.
- Uncertainty Reference: Taylor, B.N. and Kuyat, C.E., "Guidelines for Evaluating and Expressing the Uncertainty of NIST Measurement Result," NIST Technical Note 1297, U.S. Government Printing Office, Washington, DC, (1994).

BEACON ENVIRONMENTAL
2203A Commerce Road, Suite 1
Forest Hill, MD 21050 USA
1.410.838.8780

CERTIFICATE OF ANALYSIS

**University of Notre Dame**	**Site Name:** So-Cal Military Toxic Site	**Beacon Proposal:** 201201H01
Department of Biological Sciences	**Site Location:** Notspa, CA	**Lab Work Order:** 0005542
South Bend, IN 46556	**Project Manager:** Kristin Shrader-Frechette	**Reported:** 01/25/2021

Vendor: Absolute Standards, Inc. **Lab Standard No.:** 1900055

Lot No.: 061019

Absolute Standards, Inc.
800-368-1131
www.absolutestandards.com

Certified Reference Material CRM

ISO 17034 Accredited
Scopes: http://AbsoluteStandards.com

CERTIFIED WEIGHT REPORT

**Part Number:**	64091
**Lot Number:**	061019
**Description:**	VOC Calibration Level 2 (1200 ug/mL)
	42 components
**Expiration Date:**	061022
**Recommended Storage:**	Freezer (0 °C)
**Nominal Concentration (µg/mL):**	Varied
**NIST Test ID#:**	6UTB

Solvent: Methanol **Lot#** DU230-USQ10

5E-05 Balance Uncertainty
0.002 Flask Uncertainty

Volume(s) shown below were combined and diluted to (mL): 20.0

Formulated By:	Gabriel Helland	061019 DATE
Reviewed By:	Pedro L. Rentas	061019 DATE

RED = Surrogate; BLUE = Internal

	Compound	Part Number	Lot Number	Dilution Factor	Initial Vol. (mL)	Uncertainty Pipette (mL)	Initial Conc.(µg/mL)	Final Conc.(µg/mL)	Expanded Uncertainty (+/-) µg/mL	CAS#	SDS Information (Solvent Safety Info. On Attached pg.) OSHA PEL (TWA)	LD50
1.	Benzene	64088	060719	0.300	6.00	0.042	4001.1	1201.2	17.6	71-43-2	1 ppm	orl-rat 4894mg/kg
2.	Carbon tetrachloride	64088	060719	0.300	6.00	0.042	4000.8	1201.1	17.6	56-23-5	2 ppm (12.6mg/m3/8H)	orl-rat 2350mg/kg
3.	Chlorobenzene	64088	060719	0.300	6.00	0.042	4000.7	1201.1	17.6	108-90-7	75 ppm (350mg/m3/8H)	orl-rat 2290mg/kg
4.	Chloroform	64088	060719	0.300	6.00	0.042	4000.7	1201.1	17.6	67-66-3	50 ppm (240mg/m3) (CL)	orl-rat 908mg/kg
5.	1,2-Dibromoethane	64088	060719	0.300	6.00	0.042	4000.8	1201.1	17.6	106-93-4	20 ppm (8H)	orl-rat 108mg/kg
6.	1,2-Dichlorobenzene	64088	060719	0.300	6.00	0.042	4001.3	1201.3	17.6	95-50-1	50 ppm (300mg/m3) (CL)	orl-rat 500mg/kg
7.	1,3-Dichlorobenzene	64088	060719	0.300	6.00	0.042	4001.3	1201.3	17.6	541-73-1	N/A	ipr-mus 1062mg/kg
8.	1,4-Dichlorobenzene	64088	060719	0.300	6.00	0.042	4000.6	1201.1	17.6	106-46-7	75 ppm (450mg/m3/8H)	orl-rat 500mg/kg
9.	1,1-Dichloroethane	64088	060719	0.300	6.00	0.042	4001.0	1201.2	17.6	75-34-3	100 ppm	orl-rat 725mg/kg
10.	1,2-Dichloroethane	64088	060719	0.300	6.00	0.042	4001.0	1201.2	17.6	107-06-2	50 ppm (8H)	orl-rat 670mg/kg
11.	1,1-Dichloroethene	64088	060719	0.300	6.00	0.042	4001.3	1201.3	17.6	75-35-4	1 ppm (4mg/m3/8H)	orl-rat 200mg/kg
12.	cis-1,2-Dichloroethene	64088	060719	0.300	6.00	0.042	4001.3	1201.3	17.6	156-59-2	N/A	N/A
13.	trans-1,2-Dichloroethene	64088	060719	0.300	6.00	0.042	4001.3	1201.3	17.6	156-60-5	N/A	orl-rat 1235mg/kg
14.	1,4-Dioxane	64088	060719	0.300	6.00	0.042	4000.3	1201.0	17.6	123-91-1	25 ppm (90mg/m3/8H)(skin)	orl-mus 5700mg/kg
15.	Ethyl benzene	64088	060719	0.300	6.00	0.042	4001.4	1201.3	17.6	100-41-4	100 ppm (435mg/m3/8H)	orl-rat >2000mg/kg
16.	Isopropylbenzene	64088	060719	0.300	6.00	0.042	4001.3	1201.3	17.6	98-82-8	50 ppm (245mg/m3/8H)	orl-rat 1400mg/kg
17.	Methyl tert-butyl ether (MTBE)	64088	060719	0.300	6.00	0.042	4000.4	1201.0	17.6	1634-04-4	N/A	orl-rat 4g/kg
18.	2-Methylnaphthalene	64088	060719	0.300	6.00	0.042	4000.4	1201.0	17.6	91-57-6	N/A	orl-rat 1630mg/kg
19.	Naphthalene	64088	060719	0.300	6.00	0.042	4000.3	1201.0	17.6	91-20-3	10 ppm (50mg/m3/8H)	orl-rat 490mg/kg
20.	1,1,1,2-Tetrachloroethane	64088	060719	0.300	6.00	0.042	4000.5	1201.0	17.6	630-20-6	N/A	orl-rat 670mg/kg
21.	1,1,2,2-Tetrachloroethane	64088	060719	0.300	6.00	0.042	4000.5	1201.0	17.6	79-34-5	5 ppm (35mg/m3/9H)(skin)	orl-rat 800mg/kg
22.	Tetrachloroethene	64088	060719	0.300	6.00	0.042	4000.8	1201.1	17.6	127-18-4	25 ppm (170mg/m3/8H)(final)	orl-rat 2629mg/kg
23.	Toluene	64088	060719	0.300	6.00	0.042	4001.4	1201.3	17.6	108-88-3	200 ppm	orl-rat 5000mg/kg
24.	1,2,3-Trichlorobenzene	64088	060719	0.300	6.00	0.042	4000.4	1201.0	17.6	87-61-6	N/A	ipr-mus 1390mg/kg
25.	1,2,4-Trichlorobenzene	64088	060719	0.300	6.00	0.042	4000.7	1201.1	17.6	120-82-1	5 ppm (CL) (40mg/m3)	orl-rat 756mg/kg
26.	1,1,1-Trichloroethane	64088	060719	0.300	6.00	0.042	4000.8	1201.1	17.6	71-55-6	350 ppm (1900mg/m3/8H)	orl-rat 10300mg/kg
27.	1,1,2-Trichloroethane	64088	060719	0.300	6.00	0.042	4001.2	1201.2	17.6	79-00-5	10 ppm (45mg/m3/8H)(skin)	orl-rat 836mg/kg
28.	Trichloroethene	64088	060719	0.300	6.00	0.042	4000.8	1201.1	17.6	79-01-6	50 ppm (270mg/m3/8H)	orl-mus 2402mg/kg
29.	1,2,3-Trichloropropane	64088	060719	0.300	6.00	0.042	4000.3	1201.0	17.6	96-18-4	10 ppm (60mg/m3/8H)	orl-rat 149.6mg/kg
30.	1,1,2-Trichlorotrifluoroethane	64088	060719	0.300	6.00	0.042	4001.3	1201.3	17.6	76-13-1	1000 ppm (7600mg/m3/8H)	orl-rat 43g/kg
31.	1,2,4-Trimethylbenzene	64088	060719	0.300	6.00	0.042	4000.3	1201.0	17.6	95-63-6	N/A	orl-rat 5g/kg
32.	1,3,5-Trimethylbenzene	64088	060719	0.300	6.00	0.042	4000.3	1201.0	17.6	108-67-8	N/A	orl-rat 5000mg/kg
33.	o-Xylene	64088	060719	0.300	6.00	0.042	4000.6	1201.1	17.6	95-47-6	100 ppm (435mg/m3/8H)	ipr-mus 1364mg/kg
34.	m-Xylene	64088	060719	0.300	6.00	0.042	2000.4	**600.6**	8.8	108-38-3	100 ppm (435mg/m3/8H)	orl-rat 5g/kg
35.	p-Xylene	64088	060719	0.300	6.00	0.042	2001.2	**600.8**	8.8	106-42-3	100 ppm (435mg/m3/8H)	orl-rat 5g/kg
36.	Vinyl chloride	33591	052119	0.060	1.20	0.017	20030.1	1202.7	34.4	75-01-4	N/A	N/A
37.	p-Bromofluorobenzene	64088	060719	0.300	6.00	0.042	4001.3	1201.3	17.6	460-00-4	N/A	orl-rat 2700mg/kg
38.	1,2-Dichloroethane-d4	64088	060719	0.300	6.00	0.042	4000.7	1201.1	17.6	17060-07-0	N/A	orl-mus 625mg/kg
39.	Toluene-d8	64088	060719	0.300	6.00	0.042	4000.6	1201.0	17.6	2037-26-5	200 ppm	orl-rat 5000mg/kg
40.	Chlorobenzene-d5	22013	022818	0.100	2.00	0.017	2000.6	200.2	3.5	3114-55-4	N/A	orl-rat 1110mg/kg
41.	1,4-Dichlorobenzene-d4	22013	022818	0.100	2.00	0.017	2000.7	200.2	3.5	3855-82-1	N/A	orl-rat 500mg/kg
42.	Fluorobenzene	22013	022818	0.100	2.00	0.017	2001.0	200.2	3.5	462-06-6	N/A	orl-rat 4399mg/kg

- The certified value is the concentration calculated from gravimetric and volumetric measurements unless otherwise stated.
- Standards are prepared gravimetrically using balances that are calibrated with weights traceable to NIST (see above).
- Standards are certifed (+/-) 0.5% of the stated value, unless otherwise stated.
- All Standards, after opening ampule, should be stored with caps tight and under appropriate laboratory conditions.
- Uncertainty Reference: Taylor, B.N. and Kuyat, C.E., "Guidelines for Evaluating and Expressing the Uncertainty of NIST Measurement Result," NIST Technical Note 1297, U.S. Government Printing Office, Washington, DC, (1994).

2203A Commerce Road, Suite 1
Forest Hill, MD 21050 USA
1.410.838.8780

CERTIFICATE OF ANALYSIS

**University of Notre Dame**	**Site Name:** So-Cal Military Toxic Site	**Beacon Proposal:** 201201H01
Department of Biological Sciences	**Site Location:** Notspa, CA	**Lab Work Order:** 0005542
South Bend, IN 46556	**Project Manager:** Kristin Shrader-Frechette	**Reported:** 01/25/2021

Vendor: Absolute Standards, Inc. **Lab Standard No.:** 1900056

Lot No.: 061019

Absolute Standards, Inc.
800-368-1131
www.absolutestandards.com

Certified Reference Material CRM

ISO 17034 Accredited
Scopes: http://AbsoluteStandards.com

CERTIFIED WEIGHT REPORT

**Part Number:**	64092			
**Lot Number:**	061019			
**Description:**	VOC Calibration Level 3 (1600 ug/mL)			
	42 components			
**Expiration Date:**	061022		**Solvent:**	**Lot#**
**Recommended Storage:**	Freezer (0 °C)		Methanol	DU230-USQ10
**Nominal Concentration (µg/mL):**	Varied			
**NIST Test ID#:**	6UTB	5E-05	Balance Uncertainty	
Volume(s) shown below were combined and diluted to (mL):	20.0	0.002	Flask Uncertainty	

Gabriel Helland (signature)		061019
Formulated By:	Gabriel Helland	DATE
Pedro Rentas (signature)		061019
Reviewed By:	Pedro L. Rentas	DATE

RED = Surrogate; BLUE = Internal

SDS Information (Solvent Safety Info. On Attached pg.)

	Compound	Part Number	Lot Number	Dilution Factor	Initial Vol. (mL)	Uncertainty Pipette (mL)	Initial Conc.(µg/mL)	Final Conc.(µg/mL)	Expanded Uncertainty (+/-) µg/mL	CAS#	OSHA PEL (TWA)	LD50
1.	Benzene	64088	060719	0.400	8.00	0.042	4001.1	1601.6	18.1	71-43-2	1 ppm	orl-rat 4894mg/kg
2.	Carbon tetrachloride	64088	060719	0.400	8.00	0.042	4000.8	1601.5	18.1	56-23-5	2 ppm (12.6mg/m3/8H)	orl-rat 2350mg/kg
3.	Chlorobenzene	64088	060719	0.400	8.00	0.042	4000.7	1601.4	18.1	108-90-7	75 ppm (350mg/m3/8H)	orl-rat 2290mg/kg
4.	Chloroform	64088	060719	0.400	8.00	0.042	4000.7	1601.4	18.1	67-66-3	50 ppm (240mg/m3) (CL)	orl-rat 908mg/kg
5.	1,2-Dibromoethane	64088	060719	0.400	8.00	0.042	4000.8	1601.5	18.1	106-93-4	20 ppm (8H)	orl-rat 108mg/kg
6.	1,2-Dichlorobenzene	64088	060719	0.400	8.00	0.042	4001.3	1601.7	18.1	95-50-1	50 ppm (300mg/m3) (CL)	orl-rat 500mg/kg
7.	1,3-Dichlorobenzene	64088	060719	0.400	8.00	0.042	4001.3	1601.7	18.1	541-73-1	N/A	ipr-mus 1062mg/kg
8.	1,4-Dichlorobenzene	64088	060719	0.400	8.00	0.042	4000.6	1601.4	18.1	106-46-7	75 ppm (450mg/m3/8H)	orl-rat 500mg/kg
9.	1,1-Dichloroethane	64088	060719	0.400	8.00	0.042	4001.0	1601.6	18.1	75-34-3	100 ppm	orl-rat 725mg/kg
10.	1,2-Dichloroethane	64088	060719	0.400	8.00	0.042	4001.0	1601.6	18.1	107-06-2	50 ppm (8H)	orl-rat 670mg/kg
11.	1,1-Dichloroethene	64088	060719	0.400	8.00	0.042	4001.3	1601.7	18.1	75-35-4	1 ppm (4mg/m3/8H)	orl-rat 200mg/kg
12.	cis-1,2-Dichloroethene	64088	060719	0.400	8.00	0.042	4001.3	1601.7	18.1	156-59-2	N/A	N/A
13.	trans-1,2-Dichloroethene	64088	060719	0.400	8.00	0.042	4001.3	1601.7	18.1	156-60-5	N/A	orl-rat 1235mg/kg
14.	1,4-Dioxane	64088	060719	0.400	8.00	0.042	4000.3	1601.3	18.1	123-91-1	25 ppm (90mg/m3/8H)(skin)	orl-mus 5700mg/kg
15.	Ethyl benzene	64088	060719	0.400	8.00	0.042	4001.4	1601.7	18.1	100-41-4	100 ppm (435mg/m3/8H)	orl-rat >2000mg/kg
16.	Isopropylbenzene	64088	060719	0.400	8.00	0.042	4001.3	1601.7	18.1	98-82-8	50 ppm (245mg/m3/8H)	orl-rat 1400mg/kg
17.	Methyl tert-butyl ether (MTBE)	64088	060719	0.400	8.00	0.042	4000.4	1601.3	18.1	1634-04-4	N/A	orl-rat 4g/kg
18.	2-Methylnaphthalene	64088	060719	0.400	8.00	0.042	4000.4	1601.4	18.1	91-57-6	N/A	orl-rat 1630mg/kg
19.	Naphthalene	64088	060719	0.400	8.00	0.042	4000.3	1601.3	18.1	91-20-3	10 ppm (50mg/m3/8H)	orl-rat 490mg/kg
20.	1,1,1,2-Tetrachloroethane	64088	060719	0.400	8.00	0.042	4000.5	1601.4	18.1	630-20-6	N/A	orl-rat 670mg/kg
21.	1,1,2,2-Tetrachloroethane	64088	060719	0.400	8.00	0.042	4000.5	1601.4	18.1	79-34-5	5 ppm (35mg/m3/9H)(skin)	orl-rat 800mg/kg
22.	Tetrachloroethene	64088	060719	0.400	8.00	0.042	4000.8	1601.5	18.1	127-18-4	25 ppm (170mg/m3/8H)(final)	orl-rat 2629mg/kg
23.	Toluene	64088	060719	0.400	8.00	0.042	4001.4	1601.7	18.1	108-88-3	200 ppm	orl-rat 5000mg/kg
24.	1,2,3-Trichlorobenzene	64088	060719	0.400	8.00	0.042	4000.4	1601.3	18.1	87-61-6	N/A	ipr-mus 1390mg/kg
25.	1,2,4-Trichlorobenzene	64088	060719	0.400	8.00	0.042	4000.7	1601.4	18.1	120-82-1	5 ppm (CL) (40mg/m3)	orl-rat 756mg/kg
26.	1,1,1-Trichloroethane	64088	060719	0.400	8.00	0.042	4000.8	1601.5	18.1	71-55-6	350 ppm (1900mg/m3/8H)	orl-rat 10300mg/kg
27.	1,1,2-Trichloroethane	64088	060719	0.400	8.00	0.042	4001.2	1601.6	18.1	79-00-5	10 ppm (45mg/m3/8H)(skin)	orl-rat 836mg/kg
28.	Trichloroethene	64088	060719	0.400	8.00	0.042	4000.8	1601.5	18.1	79-01-6	50 ppm (270mg/m3/8H)	orl-mus 2402mg/kg
29.	1,2,3-Trichloropropane	64088	060719	0.400	8.00	0.042	4000.3	1601.3	18.1	96-18-4	10 ppm (60mg/m3/8H)	orl-rat 149.6mg/kg
30.	1,1,2-Trichlorotrifluoroethane	64088	060719	0.400	8.00	0.042	4001.3	1601.7	18.1	76-13-1	1000 ppm (7600mg/m3/8H)	orl-rat 43g/kg
31.	1,2,4-Trimethylbenzene	64088	060719	0.400	8.00	0.042	4000.3	1601.3	18.1	95-63-6	N/A	orl-rat 5g/kg
32.	1,3,5-Trimethylbenzene	64088	060719	0.400	8.00	0.042	4000.3	1601.3	18.1	108-67-8	N/A	orl-rat 5000mg/kg
33.	o-Xylene	64088	060719	0.400	8.00	0.042	4000.6	1601.4	18.1	95-47-6	100 ppm (435mg/m3/8H)	ipr-mus 1364mg/kg
34.	m-Xylene	64088	060719	0.400	8.00	0.042	2000.4	**800.8**	9.0	108-38-3	100 ppm (435mg/m3/8H)	orl-rat 5g/kg
35.	p-Xylene	64088	060719	0.400	8.00	0.042	2001.2	**801.1**	9.1	106-42-3	100 ppm (435mg/m3/8H)	orl-rat 5g/kg
36.	Vinyl chloride	33591	052119	0.080	1.60	0.017	20030.1	1603.6	34.7	75-01-4	N/A	N/A
37.	p-Bromofluorobenzene	64088	060719	0.400	8.00	0.042	4001.3	1601.7	18.1	460-00-4	N/A	orl-rat 2700mg/kg
38.	1,2-Dichloroethane-d4	64088	060719	0.400	8.00	0.042	4000.7	1601.4	18.1	17060-07-0	N/A	orl-mus 625mg/kg
39.	Toluene-d8	64088	060719	0.400	8.00	0.042	4000.6	1601.4	18.1	2037-26-5	200 ppm	orl-rat 5000mg/kg
40.	Chlorobenzene-d5	22013	022818	0.100	2.00	0.017	2000.6	200.2	3.5	3114-55-4	N/A	orl-rat 1110mg/kg
41.	1,4-Dichlorobenzene-d4	22013	022818	0.100	2.00	0.017	2000.7	200.2	3.5	3855-82-1	N/A	orl-rat 500mg/kg
42.	Fluorobenzene	22013	022818	0.100	2.00	0.017	2001.0	200.2	3.5	462-06-6	N/A	orl-rat 4399mg/kg

- The certified value is the concentration calculated from gravimetric and volumetric measurements unless otherwise stated.
- Standards are prepared gravimetrically using balances that are calibrated with weights traceable to NIST (see above).
- Standards are certifed (+/-) 0.5% of the stated value, unless otherwise stated.
- All Standards, after opening ampule, should be stored with caps tight and under appropriate laboratory conditions.
- Uncertainty Reference: Taylor, B.N. and Kuyat, C.E., "Guidelines for Evaluating and Expressing the Uncertainty of NIST Measurement Result," NIST Technical Note 1297, U.S. Government Printing Office, Washington, DC, (1994).

BEACON ENVIRONMENTAL

2203A Commerce Road, Suite 1
Forest Hill, MD 21050 USA
1.410.838.8780

CERTIFICATE OF ANALYSIS

**University of Notre Dame**	**Site Name:** So-Cal Military Toxic Site	**Beacon Proposal:** 201201H01
Department of Biological Sciences	**Site Location:** Notspa, CA	**Lab Work Order:** 0005542
South Bend, IN 46556	**Project Manager:** Kristin Shrader-Frechette	**Reported:** 01/25/2021

Vendor: Absolute Standards, Inc. **Lab Standard No.:** 1900057

Lot No.: 061019

Absolute Standards, Inc.
800-368-1131
www.absolutestandards.com

Certified Reference Material CRM

ISO 17034 Accredited
Scopes: http://AbsoluteStandards.com

CERTIFIED WEIGHT REPORT

Part Number: 64093
Lot Number: 061019
Description: VOC Calibration Level 4 (2000 ug/mL)
42 components
Expiration Date: 061022 — **Solvent:** Methanol — **Lot#** DU230-USQ10
Recommended Storage: Freezer (0 °C)
Nominal Concentration (µg/mL): Varied
NIST Test ID#: 6UTB — 5E-05 Balance Uncertainty
Volume(s) shown below were combined and diluted to (mL): 20.0 — 0.002 Flask Uncertainty

Gabriel Helland		061019
Formulated By:	Gabriel Helland	DATE
Pedro L. Rentas		061019
Reviewed By:	Pedro L. Rentas	DATE

RED = Surrogate; BLUE = Internal

SDS Information (Solvent Safety Info. On Attached pg.)

	Compound	Part Number	Lot Number	Dilution Factor	Initial Vol. (mL)	Uncertainty Pipette (mL)	Initial Conc.(µg/mL)	Final Conc.(µg/mL)	Expanded Uncertainty (+/-) µg/mL	CAS#	OSHA PEL (TWA)	LD50
1.	Benzene	64088	060719	0.500	10.00	0.042	4001.1	2002.0	18.7	71-43-2	1 ppm	orl-rat 4894mg/kg
2.	Carbon tetrachloride	64088	060719	0.500	10.00	0.042	4000.8	2001.9	18.7	56-23-5	2 ppm (12.6mg/m3/8H)	orl-rat 2350mg/kg
3.	Chlorobenzene	64088	060719	0.500	10.00	0.042	4000.7	2001.8	18.7	108-90-7	75 ppm (350mg/m3/8H)	orl-rat 2290mg/kg
4.	Chloroform	64088	060719	0.500	10.00	0.042	4000.7	2001.8	18.7	67-66-3	50 ppm (240mg/m3) (CL)	orl-rat 908mg/kg
5.	1,2-Dibromoethane	64088	060719	0.500	10.00	0.042	4000.8	2001.8	18.7	106-93-4	20 ppm (8H)	orl-rat 108mg/kg
6.	1,2-Dichlorobenzene	64088	060719	0.500	10.00	0.042	4001.3	2002.1	18.7	95-50-1	50 ppm (300mg/m3) (CL)	orl-rat 500mg/kg
7.	1,3-Dichlorobenzene	64088	060719	0.500	10.00	0.042	4001.3	2002.1	18.8	541-73-1	N/A	ipr-mus 1062mg/kg
8.	1,4-Dichlorobenzene	64088	060719	0.500	10.00	0.042	4000.6	2001.8	18.7	106-46-7	75 ppm (450mg/m3/8H)	orl-rat 500mg/kg
9.	1,1-Dichloroethane	64088	060719	0.500	10.00	0.042	4001.0	2002.0	18.7	75-34-3	100 ppm	orl-rat 725mg/kg
10.	1,2-Dichloroethane	64088	060719	0.500	10.00	0.042	4001.0	2002.0	18.7	107-06-2	50 ppm (8H)	orl-rat 670mg/kg
11.	1,1-Dichloroethene	64088	060719	0.500	10.00	0.042	4001.3	2002.1	18.7	75-35-4	1 ppm (4mg/m3/8H)	orl-rat 200mg/kg
12.	cis-1,2-Dichloroethene	64088	060719	0.500	10.00	0.042	4001.3	2002.1	18.7	156-59-2	N/A	N/A
13.	trans-1,2-Dichloroethene	64088	060719	0.500	10.00	0.042	4001.3	2002.1	18.8	156-60-5	N/A	orl-rat 1235mg/kg
14.	1,4-Dioxane	64088	060719	0.500	10.00	0.042	4000.3	2001.6	18.7	123-91-1	25 ppm (90mg/m3/8H)(skin)	orl-mus 5700mg/kg
15.	Ethyl benzene	64088	060719	0.500	10.00	0.042	4001.4	2002.2	18.7	100-41-4	100 ppm (435mg/m3/8H)	orl-rat >2000mg/kg
16.	Isopropylbenzene	64088	060719	0.500	10.00	0.042	4001.3	2002.1	18.7	98-82-8	50 ppm (245mg/m3/8H)	orl-rat 1400mg/kg
17.	Methyl tert-butyl ether (MTBE)	64088	060719	0.500	10.00	0.042	4000.4	2001.7	18.7	1634-04-4	N/A	orl-rat 4g/kg
18.	2-Methylnaphthalene	64088	060719	0.500	10.00	0.042	4000.4	2001.7	18.8	91-57-6	N/A	orl-rat 1630mg/kg
19.	Naphthalene	64088	060719	0.500	10.00	0.042	4000.3	2001.6	18.7	91-20-3	10 ppm (50mg/m3/8H)	orl-rat 490mg/kg
20.	1,1,1,2-Tetrachloroethane	64088	060719	0.500	10.00	0.042	4000.5	2001.7	18.7	630-20-6	N/A	orl-rat 670mg/kg
21.	1,1,2,2-Tetrachloroethane	64088	060719	0.500	10.00	0.042	4000.5	2001.7	18.7	79-34-5	5 ppm (35mg/m3/9H)(skin)	orl-rat 800mg/kg
22.	Tetrachloroethene	64088	060719	0.500	10.00	0.042	4000.8	2001.8	18.7	127-18-4	25 ppm (170mg/m3/8H)(final)	orl-rat 2629mg/kg
23.	Toluene	64088	060719	0.500	10.00	0.042	4001.4	2002.2	18.7	108-88-3	200 ppm	orl-rat 5000mg/kg
24.	1,2,3-Trichlorobenzene	64088	060719	0.500	10.00	0.042	4000.4	2001.7	18.7	87-61-6	N/A	ipr-mus 1390mg/kg
25.	1,2,4-Trichlorobenzene	64088	060719	0.500	10.00	0.042	4000.7	2001.8	18.7	120-82-1	5 ppm (CL) (40mg/m3)	orl-rat 756mg/kg
26.	1,1,1-Trichloroethane	64088	060719	0.500	10.00	0.042	4000.8	2001.8	18.7	71-55-6	350 ppm (1900mg/m3/8H)	orl-rat 10300mg/kg
27.	1,1,2-Trichloroethane	64088	060719	0.500	10.00	0.042	4001.2	2002.0	18.8	79-00-5	10 ppm (45mg/m3/8H)(skin)	orl-rat 836mg/kg
28.	Trichloroethene	64088	060719	0.500	10.00	0.042	4000.8	2001.9	18.7	79-01-6	50 ppm (270mg/m3/8H)	orl-mus 2402mg/kg
29.	1,2,3-Trichloropropane	64088	060719	0.500	10.00	0.042	4000.3	2001.6	18.7	96-18-4	10 ppm (60mg/m3/8H)	orl-rat 149.6mg/kg
30.	1,1,2-Trichlorotrifluoroethane	64088	060719	0.500	10.00	0.042	4001.3	2002.1	18.7	76-13-1	1000 ppm (7600mg/m3/8H)	orl-rat 43g/kg
31.	1,2,4-Trimethylbenzene	64088	060719	0.500	10.00	0.042	4000.3	2001.6	18.8	95-63-6	N/A	orl-rat 5g/kg
32.	1,3,5-Trimethylbenzene	64088	060719	0.500	10.00	0.042	4000.3	2001.6	18.8	108-67-8	N/A	orl-rat 5000mg/kg
33.	o-Xylene	64088	060719	0.500	10.00	0.042	4000.6	2001.8	18.7	95-47-6	100 ppm (435mg/m3/8H)	ipr-mus 1364mg/kg
34.	m-Xylene	64088	060719	0.500	10.00	0.042	2000.4	**1000.9**	9.4	108-38-3	100 ppm (435mg/m3/8H)	orl-rat 5g/kg
35.	p-Xylene	64088	060719	0.500	10.00	0.042	2001.2	**1001.3**	9.4	106-42-3	100 ppm (435mg/m3/8H)	orl-rat 5g/kg
36.	Vinyl chloride	33591	052119	0.100	2.00	0.017	20030.1	2004.5	35.0	75-01-4	N/A	N/A
37.	p-Bromofluorobenzene	64088	060719	0.500	10.00	0.042	4001.3	2002.1	18.7	460-00-4	N/A	orl-rat 2700mg/kg
38.	1,2-Dichloroethane-d4	64088	060719	0.500	10.00	0.042	4000.7	2001.8	18.7	17060-07-0	N/A	orl-mus 625mg/kg
39.	Toluene-d8	64088	060719	0.500	10.00	0.042	4000.6	2001.7	18.7	2037-26-5	200 ppm	orl-rat 5000mg/kg
40.	Chlorobenzene-d5	22013	022818	0.100	2.00	0.017	2000.6	200.2	3.5	3114-55-4	N/A	orl-rat 1110mg/kg
41.	1,4-Dichlorobenzene-d4	22013	022818	0.100	2.00	0.017	2000.7	200.2	3.5	3855-82-1	N/A	orl-rat 500mg/kg
42.	Fluorobenzene	22013	022818	0.100	2.00	0.017	2001.0	200.2	3.5	462-06-6	N/A	orl-rat 4399mg/kg

- The certified value is the concentration calculated from gravimetric and volumetric measurements unless otherwise stated.
- Standards are prepared gravimetrically using balances that are calibrated with weights traceable to NIST (see above).
- Standards are certifed (+/-) 0.5% of the stated value, unless otherwise stated.
- All Standards, after opening ampule, should be stored with caps tight and under appropriate laboratory conditions.
- Uncertainty Reference: Taylor, B.N. and Kuyat, C.E., "Guidelines for Evaluating and Expressing the Uncertainty of NIST Measurement Result," NIST Technical Note 1297, U.S. Government Printing Office, Washington, DC, (1994).

2203A Commerce Road, Suite 1
Forest Hill, MD 21050 USA
1.410.838.8780

CERTIFICATE OF ANALYSIS

**University of Notre Dame**	**Site Name:** So-Cal Military Toxic Site	**Beacon Proposal:** 201201H01
Department of Biological Sciences	**Site Location:** Notspa, CA	**Lab Work Order:** 0005542
South Bend, IN 46556	**Project Manager:** Kristin Shrader-Frechette	**Reported:** 01/25/2021

Vendor: Absolute Standards, Inc.   **Lab Standard No.:** 2000089

Lot No.: 021820

Absolute Standards, Inc.
800-368-1131
www.absolutestandards.com

Certified Reference Material CRM

ANAB ISO 17034 Accredited
AR-1539 Certificate Number
https://Absolutestandards.com

CERTIFIED WEIGHT REPORT

Part Number: 99080
Lot Number: 021820
Description: 8260 VOC Primary Calibration Level 0.5
84 components
Expiration Date: 021823 — **Solvent(s):** Methanol — **Lot#** DV182-USQ12
Recommended Storage: Freezer (0 °C)
Nominal Concentration (µg/mL): 5
NIST Test ID#: 6UTB — 5E-05 Balance Uncertainty
Volume(s) shown below were combined and diluted to (mL): 200.0 — 0.058 Flask Uncertainty

Gabriel Helland (signature)		021820
Formulated By:	Gabriel Helland	DATE
Pedro L. Rentas (signature)		021820
Reviewed By:	Pedro L. Rentas	DATE

	Compound	Part Number	Lot Number	Dil. Factor	Initial Vol. (mL)	Uncertainty Pipette (mL)	Initial Conc.(ug/mL)	Final Conc.(ug/mL)	Expanded Uncertainty (+/-) (µg/mL)	SDS Information (Solvent Safety Info. On Attached pg.) CAS#	OSHA PEL (TWA)	LD50
1.	Bromodichloromethane	93538	043019	0.0025	0.50	0.004	1999.8	5.0	1.2	75-27-4	N/A	orl-rat 916mg/kg
2.	Dibromochloromethane	93538	043019	0.0025	0.50	0.004	1999.8	5.0	1.2	124-48-1	N/A	orl-rat 848mg/kg
3.	cis-1,2-Dichloroethene	93538	043019	0.0025	0.50	0.004	1999.9	5.0	1.2	156-59-2	N/A	N/A
4.	trans-1,2-Dichloroethene	93538	043019	0.0025	0.50	0.004	1999.8	5.0	1.2	156-60-5	N/A	orl-rat 1235mg/kg
5.	Methylene chloride	93538	043019	0.0025	0.50	0.004	1999.9	5.0	1.2	75-09-2	500 ppm	orl-rat 820mg/kg
6.	1,1-Dichloroethene	93538	043019	0.0025	0.50	0.004	2000.3	5.0	1.2	75-35-4	1 ppm (4mg/m3/8H)	orl-rat 200mg/kg
7.	Bromochloromethane	93538	043019	0.0025	0.50	0.004	2000.0	5.0	1.2	74-97-5	200 ppm (1050mg/m3/8H)	orl-rat 5000mg/kg
8.	Bromoform	93538	043019	0.0025	0.50	0.004	2000.0	5.0	1.2	75-25-2	0.5 ppm (5mg/m3) (skin)	orl-rat 933mg/kg
9.	Carbon tetrachloride	93538	043019	0.0025	0.50	0.004	2000.0	5.0	1.2	56-23-5	2 ppm (12.6mg/m3/8H)	orl-rat 2350mg/kg
10.	Chloroform	93538	043019	0.0025	0.50	0.004	2000.0	5.0	1.2	67-66-3	50 ppm (240mg/m3) (CL)	orl-rat 908mg/kg
11.	Dibromomethane	93538	043019	0.0025	0.50	0.004	2000.0	5.0	1.2	74-95-3	N/A	orl-rat 108mg/kg
12.	1,1-Dichloroethane	93538	043019	0.0025	0.50	0.004	1999.9	5.0	1.2	75-34-3	100 ppm	orl-rat 725mg/kg
13.	2,2-Dichloropropane	93538	043019	0.0025	0.50	0.004	2000.0	5.0	1.2	594-20-7	N/A	N/A
14.	Tetrachloroethene	93538	043019	0.0025	0.50	0.004	2000.0	5.0	1.2	127-18-4	25 ppm (170mg/m3/8H)(final)	orl-rat 2629mg/kg
15.	1,1,1-Trichloroethane	93538	043019	0.0025	0.50	0.004	1999.9	5.0	1.2	71-55-6	350 ppm (1900mg/m3/8H)	orl-rat 10300mg/kg
16.	1,2-Dibromo-3-chloropropane	93538	043019	0.0025	0.50	0.004	1999.8	5.0	1.2	96-12-8	0.001 ppm	orl-rat 170mg/kg
17.	1,2-Dibromoethane	93538	043019	0.0025	0.50	0.004	1999.9	5.0	1.2	106-93-4	20 ppm (8H)	orl-rat 108mg/kg
18.	1,2-Dichloroethane	93538	043019	0.0025	0.50	0.004	1999.9	5.0	1.2	107-06-2	50 ppm (8H)	orl-rat 670mg/kg
19.	1,2-Dichloropropane	93538	043019	0.0025	0.50	0.004	1999.9	5.0	1.2	78-87-5	75 ppm (350mg/m3/8H)	orl-rat 1947mg/kg
20.	1,3-Dichloropropane	93538	043019	0.0025	0.50	0.004	1999.8	5.0	1.2	142-28-9	N/A	unr-mus 3600mg/kg
21.	1,1-Dichloropropene	93538	043019	0.0025	0.50	0.004	1981.8	5.0	1.2	563-58-6	N/A	N/A
22.	cis-1,3-Dichloropropene	93538	043019	0.0025	0.50	0.004	1999.8	5.0	1.2	10061-01-5	N/A	N/A
23.	trans-1,3-Dichloropropene	93538	043019	0.0025	0.50	0.004	1999.8	5.0	1.2	10061-02-6	N/A	N/A
24.	Hexachloro-1,3-butadiene	93538	043019	0.0025	0.50	0.004	1999.8	5.0	1.2	87-68-3	0.02 ppm (0.24mg/m3/8H)	orl-rat 82mg/kg
25.	1,1,1,2-Tetrachloroethane	93538	043019	0.0025	0.50	0.004	1999.8	5.0	1.2	630-20-6	N/A	orl-rat 670mg/kg
26.	1,1,2,2-Tetrachloroethane	93538	043019	0.0025	0.50	0.004	1999.9	5.0	1.2	79-34-5	5 ppm (35mg/m3/8H)(skin)	orl-rat 800mg/kg
27.	1,1,2-Trichloroethane	93538	043019	0.0025	0.50	0.004	1999.8	5.0	1.2	79-00-5	10 ppm (45mg/m3/8H)(skin)	orl-rat 836mg/kg
28.	Trichloroethene	93538	043019	0.0025	0.50	0.004	1999.8	5.0	1.2	79-01-6	50 ppm (270mg/m3/8H)	orl-mus 2402mg/kg
29.	1,2,3-Trichloropropane	93538	043019	0.0025	0.50	0.004	1999.8	5.0	1.2	96-18-4	10 ppm (60mg/m3/8H)	orl-rat 149.8mg/kg
30.	Benzene	93538	043019	0.0025	0.50	0.004	2000.0	5.0	1.2	71-43-2	1 ppm	orl-rat 4894mg/kg
31.	Bromobenzene	93538	043019	0.0025	0.50	0.004	2000.2	5.0	1.2	108-86-1	N/A	orl-rat 2699mg/kg
32.	n-Butyl benzene	93538	043019	0.0025	0.50	0.004	2000.9	5.0	1.2	104-51-8	N/A	N/A
33.	Ethyl benzene	93538	043019	0.0025	0.50	0.004	2000.0	5.0	1.2	100-41-4	100 ppm (435mg/m3/8H)	orl-rat >2000mg/kg
34.	p-Isopropyl toluene	93538	043019	0.0025	0.50	0.004	2000.3	5.0	1.2	99-87-6	N/A	orl-rat 4750mg/kg
35.	Naphthalene	93538	043019	0.0025	0.50	0.004	2000.1	5.0	1.2	91-20-3	10 ppm (50mg/m3/8H)	orl-rat 490mg/kg
36.	Toluene	93538	043019	0.0025	0.50	0.004	2000.1	5.0	1.2	108-88-3	200 ppm	orl-rat 5000mg/kg
37.	1,2,3-Trichlorobenzene	93538	043019	0.0025	0.50	0.004	2001.1	5.0	1.2	87-61-6	N/A	ipr-mus 1390mg/kg
38.	1,2,4-Trichlorobenzene	93538	043019	0.0025	0.50	0.004	2000.7	5.0	1.2	120-82-1	5 ppm (CL) (40mg/m3)	orl-rat 756mg/kg
39.	1,2,4-Trimethylbenzene	93538	043019	0.0025	0.50	0.004	2000.6	5.0	1.2	95-63-6	N/A	orl-rat 5g/kg
40.	1,3,5-Trimethylbenzene	93538	043019	0.0025	0.50	0.004	2000.4	5.0	1.2	108-67-8	N/A	N/A
41.	Styrene	93538	043019	0.0025	0.50	0.004	2000.0	5.0	1.2	100-42-5	100 ppm	orl-rat 5000mg/kg
42.	tert-Butyl benzene	93538	043019	0.0025	0.50	0.004	2000.2	5.0	1.2	98-06-6	N/A	N/A
43.	sec-Butyl benzene	93538	043019	0.0025	0.50	0.004	2000.4	5.0	1.2	135-98-8	N/A	orl-rat 2240mg/kg
44.	Chlorobenzene	93538	043019	0.0025	0.50	0.004	2000.6	5.0	1.2	108-90-7	75 ppm (350mg/m3/8H)	orl-rat 2290mg/kg
45.	2-Chlorotoluene	93538	043019	0.0025	0.50	0.004	2000.1	5.0	1.2	95-49-8	50 ppm (250mg/m3/8H)	orl-rat 3900mg/kg
46.	4-Chlorotoluene	93538	043019	0.0025	0.50	0.004	2000.3	5.0	1.2	106-43-4	N/A	orl-rat 2100mg/kg
47.	1,2-Dichlorobenzene	93538	043019	0.0025	0.50	0.004	2000.6	5.0	1.2	95-50-1	50 ppm (300mg/m3) (CL)	orl-rat 500mg/kg
48.	1,3-Dichlorobenzene	93538	043019	0.0025	0.50	0.004	2000.5	5.0	1.2	541-73-1	N/A	ipr-mus 1062mg/kg
49.	1,4-Dichlorobenzene	93538	043019	0.0025	0.50	0.004	2000.3	5.0	1.2	106-46-7	75 ppm (450mg/m3/8H)	orl-rat 500mg/kg
50.	Isopropylbenzene	93538	043019	0.0025	0.50	0.004	2000.6	5.0	1.2	98-82-8	50 ppm (245mg/m3/8H)	orl-rat 1400mg/kg
51.	n-Propylbenzene	93539	043019	0.0025	0.50	0.004	2000.4	5.0	1.2	103-65-1	N/A	orl-rat 6040mg/kg
52.	o-Xylene	93538	043019	0.0025	0.50	0.004	2000.0	5.0	1.2	95-47-6	100 ppm (435mg/m3/8H)	ipr-mus 1364mg/kg
53.	m-Xylene	93538	043019	0.0025	0.50	0.004	1000.0	2.5	0.6	108-38-3	100 ppm (435mg/m3/8H)	orl-rat 5g/kg
54.	p-Xylene	93538	043019	0.0025	0.50	0.004	999.9	2.5	0.6	106-42-3	100 ppm (435mg/m3/8H)	orl-rat 5g/kg
55.	Carbon disulphide	97233	021720	0.0025	0.50	0.004	2000.6	5.0	1.2	75-15-0	4 ppm (12mg/m3) (skin)	orl-rat 1200mg/kg
56.	1,4-Dioxane	97233	021720	0.0025	0.50	0.004	2000.8	5.0	1.2	123-91-1	25 ppm (90mg/m3/8H)(skin)	orl-mus 5700mg/kg
57.	Hexachloroethane	97233	021720	0.0025	0.50	0.004	2000.7	5.0	1.2	67-72-1	1 ppm (10mg/m3/8H)(skin)	orl-gpg 4970mg/kg
58.	Methyl tert-butyl ether (MTBE)	97233	021720	0.0025	0.50	0.004	2000.7	5.0	1.2	1634-04-4	N/A	orl-rat 4g/kg
59.	2-Methylnaphthalene	97233	021720	0.0025	0.50	0.004	2000.4	5.0	1.2	91-57-6	N/A	orl-rat 1630mg/kg
60.	1,1,2-Trichlorotrifluoroethane	97233	021720	0.0025	0.50	0.004	2000.9	5.0	1.2	76-13-1	1000 ppm (7600mg/m3/8H)	orl-rat 43g/kg
61.	n-Pentane	97235	120114	0.0025	0.50	0.004	2002.6	5.0	1.2	109-66-0	600 ppm(1800mg/m3/8H)	ivn-mus 446mg/kg
62.	n-Hexane	97235	120114	0.0025	0.50	0.004	2002.6	5.0	1.2	110-54-3	50 ppm(180mg/m3/8H)	orl-rat 28710mg/kg
63.	n-Heptane	97235	120114	0.0025	0.50	0.004	2003.1	5.0	1.2	142-82-5	400 ppm(1600mg/m3/8H)	ivn-mus 222mg/kg
64.	n-Octane	97235	120114	0.0025	0.50	0.004	2001.6	5.0	1.2	111-65-9	300 ppm(1450mg/m3/8H)	N/A
65.	n-Nonane	97235	120114	0.0025	0.50	0.004	2000.7	5.0	1.2	111-84-2	200 ppm (1050mg/m3/8H)	ivn-mus 218mg/kg
66.	n-Decane	97235	120114	0.0025	0.50	0.004	2001.8	5.0	1.2	124-18-5	N/A	N/A
67.	n-Undecane	97235	120114	0.0025	0.50	0.004	2003.6	5.0	1.2	1120-21-4	N/A	ivn-mus 517mg/kg
68.	n-Dodecane	97235	120114	0.0025	0.50	0.004	2000.6	5.0	1.2	112-40-3	N/A	N/A
69.	n-Tridecane	97235	120114	0.0025	0.50	0.004	2000.2	5.0	1.2	629-50-5	N/A	ivn-mus 1161mg/kg
70.	n-Tetradecane	97235	120114	0.0025	0.50	0.004	2001.5	5.0	1.2	629-59-4	N/A	N/A
71.	n-Pentadecane	97235	120114	0.0025	0.50	0.004	2001.3	5.0	1.2	629-62-9	N/A	ivn-mus 3494mg/kg
72.	Bromomethane	30058	123019	0.0025	0.50	0.004	2004.8	5.0	1.2	74-83-9	5 ppm (20mg/m3/8H) (skin)	orl-rat 214mg/kg
73.	Chloroethane	30058	123019	0.0025	0.50	0.004	2002.5	5.0	1.2	75-00-3	1000 ppm (2600mg/m3/8H)	N/A
74.	Chloromethane	30058	123019	0.0025	0.50	0.004	2003.0	5.0	1.2	74-87-3	100 ppm	orl-rat 1800mg/kg
75.	Dichlorodifluoromethane	30058	123019	0.0025	0.50	0.004	2034.9	5.1	1.2	75-71-8	1000 ppm (4950mg/m3/8H)	N/A
76.	Trichlorofluoromethane	30058	123019	0.0025	0.50	0.004	2009.6	5.0	1.2	75-69-4	1000 ppm (5600mg/m3/8H)	ipr-mus 1743mg/kg
77.	Vinyl chloride	30058	123019	0.0025	0.50	0.004	2001.2	5.0	1.2	75-01-4	N/A	N/A
78.	4-Methyl-2-pentanone	97259	112818	0.0025	0.50	0.004	2000.1	5.0	1.2	108-10-1	100 ppm (410mg/m3/8H)	orl-rat 2080mg/kg
79.	p-Bromofluorobenzene	20002	020419	0.0025	0.50	0.004	2000.7	5.0	1.2	460-00-4	N/A	orl-rat 2700mg/kg
80.	1,2-Dichloroethane-d4	20002	020419	0.0025	0.50	0.004	2000.6	5.0	1.2	17060-07-0	N/A	orl-mus 625mg/kg
81.	Toluene-d8	20002	020419	0.0025	0.50	0.004	2000.4	5.0	1.2	2037-26-5	200 ppm	orl-rat 5000mg/kg
82.	Chlorobenzene-d5	22013	021720	0.10	20.00	0.003	2001.6	200.2	1.4	3114-55-4	N/A	orl-rat 1110mg/kg
83.	1,4-Dichlorobenzene-d4	22013	021720	0.10	20.00	0.003	2002.0	200.2	1.4	3855-82-1	N/A	orl-rat 500mg/kg
84.	Fluorobenzene	22013	021720	0.10	20.00	0.003	2002.1	200.2	1.4	462-06-6	N/A	orl-rat 4399mg/kg

- The certified value is the concentration calculated from gravimetric and volumetric measurements unless otherwise stated.
- Standards are prepared gravimetrically using balances that are calibrated with weights traceable to NIST (see above).
- Standards are certifed (+/-) 0.5% of the stated value, unless otherwise stated.
- All Standards, after opening ampule, should be stored with caps tight and under appropriate laboratory conditions.
- Uncertainty Reference: Taylor, B.N. and Kuyat, C.E., "Guidelines for Evaluating and Expressing the Uncertainty of NIST Measurement Result," NIST Technical Note 1297, U.S. Government Printing Office, Washington, DC, (1994).

BEACON ENVIRONMENTAL

2203A Commerce Road, Suite 1
Forest Hill, MD 21050 USA
1.410.838.8780

CERTIFICATE OF ANALYSIS

**University of Notre Dame**	**Site Name:**	So-Cal Military Toxic Site	**Beacon Proposal:**	201201H01
Department of Biological Sciences	**Site Location:**	Notspa, CA	**Lab Work Order:**	0005542
South Bend, IN 46556	**Project Manager:**	Kristin Shrader-Frechette	**Reported:**	01/25/2021

Vendor: Absolute Standards, Inc. **Lab Standard No.:** 2000090

Lot No.: 021820

Absolute Standards, Inc.
800-368-1131
www.absolutestandards.com

Certified Reference Material CRM

ANAB ISO 17034 Accredited
AR-1539 Certificate Number
https://Absolutestandards.com

CERTIFIED WEIGHT REPORT

Part Number: 97253
Lot Number: 021820
Description: 8260 VOC Primary Calibration Level 1
84 components
Expiration Date: 021823 — **Solvent(s):** Methanol — **Lot#** DV182-USQ12
Recommended Storage: Freezer (0 °C)
Nominal Concentration (µg/mL): 10
NIST Test ID#: 6UTB — 5E-05 Balance Uncertainty
Volume(s) shown below were combined and diluted to (mL): 100.0 — 0.012 Flask Uncertainty

Gabriel Helland		021820
Formulated By:	Gabriel Helland	DATE
Pedro Rentas		021820
Reviewed By:	Pedro L. Rentas	DATE

	Compound	Part Number	Lot Number	Dil. Factor	Initial Vol. (mL)	Uncertainty Pipette (mL)	Initial Conc.(ug/mL)	Final Conc.(ug/mL)	Expanded Uncertainty (+/-) (µg/mL)	SDS Information (Solvent Safety Info. On Attached pg.) CAS#	OSHA PEL (TWA)	LD50
1.	Bromodichloromethane	93538	043019	0.005	0.50	0.004	1999.8	10.0	0.5	75-27-4	N/A	orl-rat 916mg/kg
2.	Dibromochloromethane	93538	043019	0.005	0.50	0.004	1999.8	10.0	0.5	124-48-1	N/A	orl-rat 848mg/kg
3.	cis-1,2-Dichloroethene	93538	043019	0.005	0.50	0.004	1999.9	10.0	0.5	156-59-2	N/A	N/A
4.	trans-1,2-Dichloroethene	93538	043019	0.005	0.50	0.004	1999.8	10.0	0.5	156-60-5	N/A	orl-rat 1235mg/kg
5.	Methylene chloride	93538	043019	0.005	0.50	0.004	1999.9	10.0	0.5	75-09-2	500 ppm	orl-rat 820mg/kg
6.	1,1-Dichloroethene	93538	043019	0.005	0.50	0.004	2000.3	10.0	0.5	75-35-4	1 ppm (4mg/m3/8H)	orl-rat 200mg/kg
7.	Bromochloromethane	93538	043019	0.005	0.50	0.004	2000.0	10.0	0.5	74-97-5	200 ppm (1050mg/m3/8H)	orl-rat 5000mg/kg
8.	Bromoform	93538	043019	0.005	0.50	0.004	2000.0	10.0	0.5	75-25-2	0.5 ppm (5mg/m3) (skin)	orl-rat 933mg/kg
9.	Carbon tetrachloride	93538	043019	0.005	0.50	0.004	2000.0	10.0	0.5	56-23-5	2 ppm (12.6mg/m3/8H)	orl-rat 2350mg/kg
10.	Chloroform	93538	043019	0.005	0.50	0.004	2000.0	10.0	0.5	67-66-3	50 ppm (240mg/m3) (CL)	orl-rat 908mg/kg
11.	Dibromomethane	93538	043019	0.005	0.50	0.004	2000.0	10.0	0.5	74-95-3	N/A	orl-rat 108mg/kg
12.	1,1-Dichloroethane	93538	043019	0.005	0.50	0.004	1999.9	10.0	0.5	75-34-3	100 ppm	orl-rat 725mg/kg
13.	2,2-Dichloropropane	93538	043019	0.005	0.50	0.004	2000.0	10.0	0.5	594-20-7	N/A	N/A
14.	Tetrachloroethene	93538	043019	0.005	0.50	0.004	2000.0	10.0	0.5	127-18-4	25 ppm (170mg/m3/8H)(final)	orl-rat 2629mg/kg
15.	1,1,1-Trichloroethane	93538	043019	0.005	0.50	0.004	1999.9	10.0	0.5	71-55-6	350 ppm (1900mg/m3/8H)	orl-rat 10300mg/kg
16.	1,2-Dibromo-3-chloropropane	93538	043019	0.005	0.50	0.004	1999.8	10.0	0.5	96-12-8	0.001 ppm	orl-rat 170mg/kg
17.	1,2-Dibromoethane	93538	043019	0.005	0.50	0.004	1999.9	10.0	0.5	106-93-4	20 ppm (8H)	orl-rat 108mg/kg
18.	1,2-Dichloroethane	93538	043019	0.005	0.50	0.004	1999.9	10.0	0.5	107-06-2	50 ppm (8H)	orl-rat 670mg/kg
19.	1,2-Dichloropropane	93538	043019	0.005	0.50	0.004	1999.9	10.0	0.5	78-87-5	75 ppm (350mg/m3/8H)	orl-rat 1947mg/kg
20.	1,3-Dichloropropane	93538	043019	0.005	0.50	0.004	1999.8	10.0	0.5	142-28-9	N/A	unr-mus 3600mg/kg
21.	1,1-Dichloropropene	93538	043019	0.005	0.50	0.004	1981.8	9.9	0.5	563-58-6	N/A	N/A
22.	cis-1,3-Dichloropropene	93538	043019	0.005	0.50	0.004	1999.8	10.0	0.5	10061-01-5	N/A	N/A
23.	trans-1,3-Dichloropropene	93538	043019	0.005	0.50	0.004	1999.8	10.0	0.5	10061-02-6	N/A	N/A
24.	Hexachloro-1,3-butadiene	93538	043019	0.005	0.50	0.004	1999.8	10.0	0.5	87-68-3	0.02 ppm (0.24mg/m3/8H)	orl-rat 82mg/kg
25.	1,1,1,2-Tetrachloroethane	93538	043019	0.005	0.50	0.004	1999.8	10.0	0.5	630-20-6	N/A	orl-rat 670mg/kg
26.	1,1,2,2-Tetrachloroethane	93538	043019	0.005	0.50	0.004	1999.9	10.0	0.5	79-34-5	5 ppm (35mg/m3/9H)(skin)	orl-rat 800mg/kg
27.	1,1,2-Trichloroethane	93538	043019	0.005	0.50	0.004	1999.8	10.0	0.5	79-00-5	10 ppm (45mg/m3/8H)(skin)	orl-rat 836mg/kg
28.	Trichloroethene	93538	043019	0.005	0.50	0.004	1999.8	10.0	0.5	79-01-6	50 ppm (270mg/m3/8H)	orl-mus 2402mg/kg
29.	1,2,3-Trichloropropane	93538	043019	0.005	0.50	0.004	1999.8	10.0	0.5	96-18-4	10 ppm (60mg/m3/8H)	orl-rat 149.6mg/kg
30.	Benzene	93538	043019	0.005	0.50	0.004	2000.0	10.0	0.5	71-43-2	1 ppm	orl-rat 4894mg/kg
31.	Bromobenzene	93538	043019	0.005	0.50	0.004	2000.2	10.0	0.5	108-86-1	N/A	orl-rat 2699mg/kg
32.	n-Butyl benzene	93538	043019	0.005	0.50	0.004	2000.9	10.0	0.5	104-51-8	N/A	N/A
33.	Ethyl benzene	93538	043019	0.005	0.50	0.004	2000.0	10.0	0.5	100-41-4	100 ppm (435mg/m3/8H)	orl-rat >2000mg/kg
34.	p-Isopropyl toluene	93538	043019	0.005	0.50	0.004	2000.3	10.0	0.5	99-87-6	N/A	orl-rat 4750mg/kg
35.	Naphthalene	93538	043019	0.005	0.50	0.004	2000.1	10.0	0.5	91-20-3	10 ppm (50mg/m3/8H)	orl-rat 490mg/kg
36.	Toluene	93538	043019	0.005	0.50	0.004	2000.1	10.0	0.5	108-88-3	200 ppm	orl-rat 5000mg/kg
37.	1,2,3-Trichlorobenzene	93538	043019	0.005	0.50	0.004	2001.1	10.0	0.5	87-61-6	N/A	ipr-mus 1390mg/kg
38.	1,2,4-Trichlorobenzene	93538	043019	0.005	0.50	0.004	2000.7	10.0	0.5	120-82-1	5 ppm (CL) (40mg/m3)	orl-rat 756mg/kg
39.	1,2,4-Trimethylbenzene	93538	043019	0.005	0.50	0.004	2000.6	10.0	0.5	95-63-6	N/A	orl-rat 5g/kg
40.	1,3,5-Trimethylbenzene	93538	043019	0.005	0.50	0.004	2000.4	10.0	0.5	108-67-8	N/A	N/A
41.	Styrene	93538	043019	0.005	0.50	0.004	2000.0	10.0	0.5	100-42-5	100 ppm	orl-rat 5000mg/kg
42.	tert-Butyl benzene	93538	043019	0.005	0.50	0.004	2000.2	10.0	0.5	98-06-6	N/A	N/A
43.	sec-Butyl benzene	93538	043019	0.005	0.50	0.004	2000.4	10.0	0.5	135-98-8	N/A	orl-rat 2240mg/kg
44.	Chlorobenzene	93538	043019	0.005	0.50	0.004	2000.6	10.0	0.5	108-90-7	75 ppm (350mg/m3/8H)	orl-rat 2290mg/kg
45.	2-Chlorotoluene	93538	043019	0.005	0.50	0.004	2000.1	10.0	0.5	95-49-8	50 ppm (250mg/m3/8H)	orl-rat 3900mg/kg
46.	4-Chlorotoluene	93538	043019	0.005	0.50	0.004	2000.3	10.0	0.5	106-43-4	N/A	orl-rat 2100mg/kg
47.	1,2-Dichlorobenzene	93538	043019	0.005	0.50	0.004	2000.6	10.0	0.5	95-50-1	50 ppm (300mg/m3) (CL)	orl-rat 500mg/kg
48.	1,3-Dichlorobenzene	93538	043019	0.005	0.50	0.004	2000.5	10.0	0.5	541-73-1	N/A	ipr-mus 1062mg/kg
49.	1,4-Dichlorobenzene	93538	043019	0.005	0.50	0.004	2000.3	10.0	0.5	106-46-7	75 ppm (450mg/m3/8H)	orl-rat 500mg/kg
50.	Isopropylbenzene	93538	043019	0.005	0.50	0.004	2000.6	10.0	0.5	98-82-8	50 ppm (245mg/m3/8H)	orl-rat 1400mg/kg
51.	n-Propylbenzene	93538	043019	0.005	0.50	0.004	2000.4	10.0	0.5	103-65-1	N/A	orl-rat 6040mg/kg
52.	o-Xylene	93538	043019	0.005	0.50	0.004	2000.0	10.0	0.5	95-47-6	100 ppm (435mg/m3/8H)	ipr-mus 1364mg/kg
53.	m-Xylene	93538	043019	0.005	0.50	0.004	1000.0	**5.0**	0.3	108-38-3	100 ppm (435mg/m3/8H)	orl-rat 5g/kg
54.	p-Xylene	93538	043019	0.005	0.50	0.004	999.9	**5.0**	0.3	106-42-3	100 ppm (435mg/m3/8H)	orl-rat 5g/kg
55.	Carbon disulphide	97233	021720	0.005	0.50	0.004	2000.6	10.0	0.5	75-15-0	4 ppm (12mg/m3) (skin)	orl-rat 1200mg/kg
56.	1,4-Dioxane	97233	021720	0.005	0.50	0.004	2000.8	10.0	0.5	123-91-1	25 ppm (90mg/m3/8H)(skin)	orl-mus 5700mg/kg
57.	Hexachloroethane	97233	021720	0.005	0.50	0.004	2000.7	10.0	0.5	67-72-1	1 ppm (10mg/m3/8H)(skin)	orl-gpg 4970mg/kg
58.	Methyl tert-butyl ether (MTBE)	97233	021720	0.005	0.50	0.004	2000.7	10.0	0.5	1634-04-4	N/A	orl-rat 4g/kg
59.	2-Methylnaphthalene	97233	021720	0.005	0.50	0.004	2000.4	10.0	0.5	91-57-6	N/A	orl-rat 1630mg/kg
60.	1,1,2-Trichlorotrifluoroethane	97233	021720	0.005	0.50	0.004	2000.9	10.0	0.5	76-13-1	1000 ppm (7600mg/m3/8H)	orl-rat 43g/kg
61.	n-Pentane	97235	120114	0.005	0.50	0.004	2002.6	10.0	0.5	109-66-0	600 ppm(1800mg/m3/8H)	ivn-mus 446mg/kg
62.	n-Hexane	97235	120114	0.005	0.50	0.004	2002.6	10.0	0.5	110-54-3	50 ppm(180mg/m3/8H)	orl-rat 28710mg/kg
63.	n-Heptane	97235	120114	0.005	0.50	0.004	2003.1	10.0	0.5	142-82-5	400 ppm(1600mg/m3/8H)	ivn-mus 222mg/kg
64.	n-Octane	97235	120114	0.005	0.50	0.004	2001.6	10.0	0.5	111-65-9	300 ppm(1450mg/m3/8H)	N/A
65.	n-Nonane	97235	120114	0.005	0.50	0.004	2000.7	10.0	0.5	111-84-2	200 ppm (1050mg/m3/8H)	ivn-mus 218mg/kg
66.	n-Decane	97235	120114	0.005	0.50	0.004	2001.8	10.0	0.5	124-18-5	N/A	N/A
67.	n-Undecane	97235	120114	0.005	0.50	0.004	2003.6	10.0	0.5	1120-21-4	N/A	ivn-mus 517mg/kg
68.	n-Dodecane	97235	120114	0.005	0.50	0.004	2000.6	10.0	0.5	112-40-3	N/A	N/A
69.	n-Tridecane	97235	120114	0.005	0.50	0.004	2000.2	10.0	0.5	629-50-5	N/A	ivn-mus 1161mg/kg
70.	n-Tetradecane	97235	120114	0.005	0.50	0.004	2001.5	10.0	0.5	629-59-4	N/A	N/A
71.	n-Pentadecane	97235	120114	0.005	0.50	0.004	2001.3	10.0	0.5	629-62-9	N/A	ivn-mus 3494mg/kg
72.	Bromomethane	30058	123019	0.005	0.50	0.004	2004.8	10.0	0.5	74-83-9	5 ppm (20mg/m3/8H) (skin)	orl-rat 214mg/kg
73.	Chloroethane	30058	123019	0.005	0.50	0.004	2002.5	10.0	0.5	75-00-3	1000 ppm (2600mg/m3/8H)	N/A
74.	Chloromethane	30058	123019	0.005	0.50	0.004	2003.0	10.0	0.5	74-87-3	100 ppm	orl-rat 1800mg/kg
75.	Dichlorodifluoromethane	30058	123019	0.005	0.50	0.004	2034.9	10.2	0.5	75-71-8	1000 ppm (4950mg/m3/8H)	N/A
76.	Trichlorofluoromethane	30058	123019	0.005	0.50	0.004	2009.6	10.0	0.5	75-69-4	1000 ppm (5600mg/m3/8H)	ipr-mus 1743mg/kg
77.	Vinyl chloride	30058	123019	0.005	0.50	0.004	2001.2	10.0	0.5	75-01-4	N/A	N/A
78.	4-Methyl-2-pentanone	97259	112816	0.005	0.50	0.004	2000.1	10.0	0.5	108-10-1	100 ppm (410mg/m3/8H)	orl-rat 2080mg/kg
79.	p-Bromofluorobenzene	20002	020419	0.005	0.50	0.004	2000.7	10.0	0.5	460-00-4	N/A	orl-rat 2700mg/kg
80.	1,2-Dichloroethane-d4	20002	020419	0.005	0.50	0.004	2000.6	10.0	0.5	17060-07-0	N/A	orl-mus 625mg/kg
81.	Toluene-d8	20002	020419	0.005	0.50	0.004	2000.4	10.0	0.5	2037-26-5	200 ppm	orl-rat 5000mg/kg
82.	Chlorobenzene-d5	22013	021720	0.10	10.00	0.042	2001.6	**200.1**	1.9	3114-55-4	N/A	orl-rat 1110mg/kg
83.	1,4-Dichlorobenzene-d4	22013	021720	0.10	10.00	0.042	2002.0	**200.2**	1.9	3855-82-1	N/A	orl-rat 500mg/kg
84.	Fluorobenzene	22013	021720	0.10	10.00	0.042	2002.1	**200.2**	1.9	462-06-6	N/A	orl-rat 4399mg/kg

- The certified value is the concentration calculated from gravimetric and volumetric measurements unless otherwise stated.
- Standards are prepared gravimetrically using balances that are calibrated with weights traceable to NIST (see above).
- Standards are certifed (+/-) 0.5% of the stated value, unless otherwise stated.
- All Standards, after opening ampule, should be stored with caps tight and under appropriate laboratory conditions.
- Uncertainty Reference: Taylor, B.N. and Kuyat, C.E., "Guidelines for Evaluating and Expressing the Uncertainty of NIST Measurement Result," NIST Technical Note 1297, U.S. Government Printing Office, Washington, DC, (1994).

2203A Commerce Road, Suite 1
Forest Hill, MD 21050 USA
1.410.838.8780

CERTIFICATE OF ANALYSIS

**University of Notre Dame**	**Site Name:** So-Cal Military Toxic Site	**Beacon Proposal:** 201201H01
Department of Biological Sciences	**Site Location:** Notspa, CA	**Lab Work Order:** 0005542
South Bend, IN 46556	**Project Manager:** Kristin Shrader-Frechette	**Reported:** 01/25/2021

Vendor: Absolute Standards, Inc. **Lab Standard No.:** 2000091

Lot No.: 021823

Absolute Standards, Inc.
800-368-1131
www.absolutestandards.com

Certified Reference Material CRM

ANAB ISO 17034 Accredited
AR-1539 Certificate Number
https://Absolutestandards.com

CERTIFIED WEIGHT REPORT

**Part Number:**	97254		
**Lot Number:**	021820		
**Description:**	8260 VOC Primary Calibration Level 2		
	84 components		
**Expiration Date:**	021823	**Solvent(s):**	**Lot#**
**Recommended Storage:**	Freezer (0 °C)	Methanol	DV182-USQ12
**Nominal Concentration (µg/mL):**	20		
**NIST Test ID#:**	6UTB	5E-05	Balance Uncertainty
Volume(s) shown below were combined and diluted to (mL):	100.0	0.012	Flask Uncertainty

Gabriel Helland (signature)		021820
Formulated By:	Gabriel Helland	DATE
(signature)		021820
Reviewed By:	Pedro L. Rentas	DATE

	Compound	Part Number	Lot Number	Dil. Factor	Initial Vol. (mL)	Uncertainty Pipette (mL)	Initial Conc.(ug/mL)	Final Conc.(ug/mL)	Expanded Uncertainty (+/-) (µg/mL)	SDS Information (Solvent Safety Info. On Attached pg.) CAS#	OSHA PEL (TWA)	LD50
1.	Bromodichloromethane	93538	043019	0.010	1.00	0.004	1999.8	20.0	0.5	75-27-4	N/A	orl-rat 916mg/kg
2.	Dibromochloromethane	93538	043019	0.010	1.00	0.004	1999.8	20.0	0.5	124-48-1	N/A	orl-rat 848mg/kg
3.	cis-1,2-Dichloroethene	93538	043019	0.010	1.00	0.004	1999.9	20.0	0.5	156-59-2	N/A	N/A
4.	trans-1,2-Dichloroethene	93538	043019	0.010	1.00	0.004	1999.8	20.0	0.5	156-60-5	N/A	orl-rat 1235mg/kg
5.	Methylene chloride	93538	043019	0.010	1.00	0.004	1999.9	20.0	0.5	75-09-2	500 ppm	orl-rat 820mg/kg
6.	1,1-Dichloroethene	93538	043019	0.010	1.00	0.004	2000.3	20.0	0.5	75-35-4	1 ppm (4mg/m3/8H)	orl-rat 200mg/kg
7.	Bromochloromethane	93538	043019	0.010	1.00	0.004	2000.0	20.0	0.5	74-97-5	200 ppm (1050mg/m3/8H)	orl-rat 5000mg/kg
8.	Bromoform	93538	043019	0.010	1.00	0.004	2000.0	20.0	0.5	75-25-2	0.5 ppm (5mg/m3) (skin)	orl-rat 933mg/kg
9.	Carbon tetrachloride	93538	043019	0.010	1.00	0.004	2000.0	20.0	0.5	56-23-5	2 ppm (12.6mg/m3/8H)	orl-rat 2350mg/kg
10.	Chloroform	93538	043019	0.010	1.00	0.004	2000.0	20.0	0.5	67-66-3	50 ppm (240mg/m3) (CL)	orl-rat 908mg/kg
11.	Dibromomethane	93538	043019	0.010	1.00	0.004	2000.0	20.0	0.5	74-95-3	N/A	orl-rat 108mg/kg
12.	1,1-Dichloroethane	93538	043019	0.010	1.00	0.004	1999.9	20.0	0.5	75-34-3	100 ppm	orl-rat 725mg/kg
13.	2,2-Dichloropropane	93538	043019	0.010	1.00	0.004	2000.0	20.0	0.5	594-20-7	N/A	N/A
14.	Tetrachloroethene	93538	043019	0.010	1.00	0.004	2000.0	20.0	0.5	127-18-4	25 ppm (170mg/m3/8H)(final)	orl-rat 2629mg/kg
15.	1,1,1-Trichloroethane	93538	043019	0.010	1.00	0.004	1999.9	20.0	0.5	71-55-6	350 ppm (1900mg/m3/8H)	orl-rat 10300mg/kg
16.	1,2-Dibromo-3-chloropropane	93538	043019	0.010	1.00	0.004	1999.8	20.0	0.5	96-12-8	0.001 ppm	orl-rat 170mg/kg
17.	1,2-Dibromoethane	93538	043019	0.010	1.00	0.004	1999.9	20.0	0.5	106-93-4	20 ppm (8H)	orl-rat 108mg/kg
18.	1,2-Dichloroethane	93538	043019	0.010	1.00	0.004	1999.9	20.0	0.5	107-06-2	50 ppm (8H)	orl-rat 670mg/kg
19.	1,2-Dichloropropane	93538	043019	0.010	1.00	0.004	1999.9	20.0	0.5	78-87-5	75 ppm (350mg/m3/8H)	orl-rat 1947mg/kg
20.	1,3-Dichloropropane	93538	043019	0.010	1.00	0.004	1999.8	20.0	0.5	142-28-9	N/A	unr-mus 3600mg/kg
21.	1,1-Dichloropropene	93538	043019	0.010	1.00	0.004	1981.8	19.8	0.5	563-58-6	N/A	N/A
22.	cis-1,3-Dichloropropene	93538	043019	0.010	1.00	0.004	1999.8	20.0	0.5	10061-01-5	N/A	N/A
23.	trans-1,3-Dichloropropene	93538	043019	0.010	1.00	0.004	1999.8	20.0	0.5	10061-02-6	N/A	N/A
24.	Hexachloro-1,3-butadiene	93538	043019	0.010	1.00	0.004	1999.8	20.0	0.5	87-68-3	0.02 ppm (0.24mg/m3/8H)	orl-rat 82mg/kg
25.	1,1,1,2-Tetrachloroethane	93538	043019	0.010	1.00	0.004	1999.8	20.0	0.5	630-20-6	N/A	orl-rat 670mg/kg
26.	1,1,2,2-Tetrachloroethane	93538	043019	0.010	1.00	0.004	1999.9	20.0	0.5	79-34-5	5 ppm (35mg/m3/9H)(skin)	orl-rat 800mg/kg
27.	1,1,2-Trichloroethane	93538	043019	0.010	1.00	0.004	1999.8	20.0	0.5	79-00-5	10 ppm (45mg/m3/8H)(skin)	orl-rat 836mg/kg
28.	Trichloroethene	93538	043019	0.010	1.00	0.004	1999.8	20.0	0.5	79-01-6	50 ppm (270mg/m3/8H)	orl-mus 2402mg/kg
29.	1,2,3-Trichloropropane	93538	043019	0.010	1.00	0.004	1999.8	20.0	0.5	96-18-4	10 ppm (60mg/m3/8H)	orl-rat 149.6mg/kg
30.	Benzene	93538	043019	0.010	1.00	0.004	2000.0	20.0	0.5	71-43-2	1 ppm	orl-rat 4894mg/kg
31.	Bromobenzene	93538	043019	0.010	1.00	0.004	2000.2	20.0	0.5	108-86-1	N/A	orl-rat 2699mg/kg
32.	n-Butyl benzene	93538	043019	0.010	1.00	0.004	2000.9	20.0	0.5	104-51-8	N/A	N/A
33.	Ethyl benzene	93538	043019	0.010	1.00	0.004	2000.0	20.0	0.5	100-41-4	100 ppm (435mg/m3/8H)	orl-rat >2000mg/kg
34.	p-Isopropyl toluene	93538	043019	0.010	1.00	0.004	2000.3	20.0	0.5	99-87-6	N/A	orl-rat 4750mg/kg
35.	Naphthalene	93538	043019	0.010	1.00	0.004	2000.1	20.0	0.5	91-20-3	10 ppm (50mg/m3/8H)	orl-rat 490mg/kg
36.	Toluene	93538	043019	0.010	1.00	0.004	2000.1	20.0	0.5	108-88-3	200 ppm	orl-rat 5000mg/kg
37.	1,2,3-Trichlorobenzene	93538	043019	0.010	1.00	0.004	2001.1	20.0	0.5	87-61-6	N/A	ipr-mus 1390mg/kg
38.	1,2,4-Trichlorobenzene	93538	043019	0.010	1.00	0.004	2000.7	20.0	0.5	120-82-1	5 ppm (CL) (40mg/m3)	orl-rat 756mg/kg
39.	1,2,4-Trimethylbenzene	93538	043019	0.010	1.00	0.004	2000.6	20.0	0.5	95-63-6	N/A	orl-rat 5g/kg
40.	1,3,5-Trimethylbenzene	93538	043019	0.010	1.00	0.004	2000.4	20.0	0.5	108-67-8	N/A	N/A
41.	Styrene	93538	043019	0.010	1.00	0.004	2000.0	20.0	0.5	100-42-5	100 ppm	orl-rat 5000mg/kg
42.	tert-Butyl benzene	93538	043019	0.010	1.00	0.004	2000.2	20.0	0.5	98-06-6	N/A	N/A
43.	sec-Butyl benzene	93538	043019	0.010	1.00	0.004	2000.4	20.0	0.5	135-98-8	N/A	orl-rat 2240mg/kg
44.	Chlorobenzene	93538	043019	0.010	1.00	0.004	2000.6	20.0	0.5	108-90-7	75 ppm (350mg/m3/8H)	orl-rat 2290mg/kg
45.	2-Chlorotoluene	93538	043019	0.010	1.00	0.004	2000.1	20.0	0.5	95-49-8	50 ppm (250mg/m3/8H)	orl-rat 3900mg/kg
46.	4-Chlorotoluene	93538	043019	0.010	1.00	0.004	2000.3	20.0	0.5	106-43-4	N/A	orl-rat 2100mg/kg
47.	1,2-Dichlorobenzene	93538	043019	0.010	1.00	0.004	2000.6	20.0	0.5	95-50-1	50 ppm (300mg/m3) (CL)	orl-rat 500mg/kg
48.	1,3-Dichlorobenzene	93538	043019	0.010	1.00	0.004	2000.5	20.0	0.5	541-73-1	N/A	ipr-mus 1062mg/kg
49.	1,4-Dichlorobenzene	93538	043019	0.010	1.00	0.004	2000.3	20.0	0.5	106-46-7	75 ppm (450mg/m3/8H)	orl-rat 500mg/kg
50.	Isopropylbenzene	93538	043019	0.010	1.00	0.004	2000.6	20.0	0.5	98-82-8	50 ppm (245mg/m3/8H)	orl-rat 1400mg/kg
51.	n-Propylbenzene	93538	043019	0.010	1.00	0.004	2000.4	20.0	0.5	103-65-1	N/A	orl-rat 6040mg/kg
52.	o-Xylene	93538	043019	0.010	1.00	0.004	2000.0	20.0	0.5	95-47-6	100 ppm (435mg/m3/8H)	ipr-mus 1364mg/kg
53.	m-Xylene	93538	043019	0.010	1.00	0.004	1000.0	**10.0**	0.3	108-38-3	100 ppm (435mg/m3/8H)	orl-rat 5g/kg
54.	p-Xylene	93538	043019	0.010	1.00	0.004	999.9	**10.0**	0.3	106-42-3	100 ppm (435mg/m3/8H)	orl-rat 5g/kg
55.	Carbon disulphide	97233	021720	0.010	1.00	0.004	2000.6	20.0	0.5	75-15-0	4 ppm (12mg/m3) (skin)	orl-rat 1200mg/kg
56.	1,4-Dioxane	97233	021720	0.010	1.00	0.004	2000.8	20.0	0.5	123-91-1	25 ppm (90mg/m3/8H)(skin)	orl-mus 5700mg/kg
57.	Hexachloroethane	97233	021720	0.010	1.00	0.004	2000.7	20.0	0.5	67-72-1	1 ppm (10mg/m3/8H)(skin)	orl-gpg 4970mg/kg
58.	Methyl tert-butyl ether (MTBE)	97233	021720	0.010	1.00	0.004	2000.7	20.0	0.5	1634-04-4	N/A	orl-rat 4g/kg
59.	2-Methylnaphthalene	97233	021720	0.010	1.00	0.004	2000.4	20.0	0.5	91-57-6	N/A	orl-rat 1630mg/kg
60.	1,1,2-Trichlorotrifluoroethane	97233	021720	0.010	1.00	0.004	2000.9	20.0	0.5	76-13-1	1000 ppm (7600mg/m3/8H)	orl-rat 43g/kg
61.	n-Pentane	97235	120114	0.010	1.00	0.004	2002.6	20.0	0.5	109-66-0	600 ppm(1800mg/m3/8H)	ivn-mus 446mg/kg
62.	n-Hexane	97235	120114	0.010	1.00	0.004	2002.6	20.0	0.5	110-54-3	50 ppm(180mg/m3/8H)	orl-rat 28710mg/kg
63.	n-Heptane	97235	120114	0.010	1.00	0.004	2003.1	20.0	0.5	142-82-5	400 ppm(1600mg/m3/8H)	ivn-mus 222mg/kg
64.	n-Octane	97235	120114	0.010	1.00	0.004	2001.6	20.0	0.5	111-65-9	300 ppm(1450mg/m3/8H)	N/A
65.	n-Nonane	97235	120114	0.010	1.00	0.004	2000.7	20.0	0.5	111-84-2	200 ppm (1050mg/m3/8H)	ivn-mus 218mg/kg
66.	n-Decane	97235	120114	0.010	1.00	0.004	2001.8	20.0	0.5	124-18-5	N/A	N/A
67.	n-Undecane	97235	120114	0.010	1.00	0.004	2003.6	20.0	0.5	1120-21-4	N/A	ivn-mus 517mg/kg
68.	n-Dodecane	97235	120114	0.010	1.00	0.004	2000.6	20.0	0.5	112-40-3	N/A	N/A
69.	n-Tridecane	97235	120114	0.010	1.00	0.004	2000.2	20.0	0.5	629-50-5	N/A	ivn-mus 1161mg/kg
70.	n-Tetradecane	97235	120114	0.010	1.00	0.004	2001.5	20.0	0.5	629-59-4	N/A	N/A
71.	n-Pentadecane	97235	120114	0.010	1.00	0.004	2001.3	20.0	0.5	629-62-9	N/A	ivn-mus 3494mg/kg
72.	Bromomethane	30058	123019	0.010	1.00	0.004	2004.8	20.0	0.5	74-83-9	5 ppm (20mg/m3/8H) (skin)	orl-rat 214mg/kg
73.	Chloroethane	30058	123019	0.010	1.00	0.004	2002.5	20.0	0.5	75-00-3	1000 ppm (2600mg/m3/8H)	N/A
74.	Chloromethane	30058	123019	0.010	1.00	0.004	2003.0	20.0	0.5	74-87-3	100 ppm	orl-rat 1800mg/kg
75.	Dichlorodifluoromethane	30058	123019	0.010	1.00	0.004	2034.9	20.3	0.5	75-71-8	1000 ppm (4950mg/m3/8H)	N/A
76.	Trichlorofluoromethane	30058	123019	0.010	1.00	0.004	2009.6	20.1	0.5	75-69-4	1000 ppm (5600mg/m3/8H)	ipr-mus 1743mg/kg
77.	Vinyl chloride	30058	123019	0.010	1.00	0.004	2001.2	20.0	0.5	75-01-4	N/A	N/A
78.	4-Methyl-2-pentanone	97259	112816	0.010	1.00	0.004	2000.1	20.0	0.5	108-10-1	100 ppm (410mg/m3/8H)	orl-rat 2080mg/kg
79.	p-Bromofluorobenzene	20002	020419	0.010	1.00	0.004	2000.7	20.0	0.5	460-00-4	N/A	orl-rat 2700mg/kg
80.	1,2-Dichloroethane-d4	20002	020419	0.010	1.00	0.004	2000.6	20.0	0.5	17060-07-0	N/A	orl-mus 625mg/kg
81.	Toluene-d8	20002	020419	0.010	1.00	0.004	2000.4	20.0	0.5	2037-26-5	200 ppm	orl-rat 5000mg/kg
82.	Chlorobenzene-d5	22013	021720	0.10	10.00	0.042	2001.6	**200.1**	1.9	3114-55-4	N/A	orl-rat 1110mg/kg
83.	1,4-Dichlorobenzene-d4	22013	021720	0.10	10.00	0.042	2002.0	**200.2**	1.9	3855-82-1	N/A	orl-rat 500mg/kg
84.	Fluorobenzene	22013	021720	0.10	10.00	0.042	2002.1	**200.2**	1.9	462-06-6	N/A	orl-rat 4399mg/kg

- The certified value is the concentration calculated from gravimetric and volumetric measurements unless otherwise stated.
- Standards are prepared gravimetrically using balances that are calibrated with weights traceable to NIST (see above).
- Standards are certifed (+/-) 0.5% of the stated value, unless otherwise stated.
- All Standards, after opening ampule, should be stored with caps tight and under appropriate laboratory conditions.
- Uncertainty Reference: Taylor, B.N. and Kuyat, C.E., "Guidelines for Evaluating and Expressing the Uncertainty of NIST Measurement Result," NIST Technical Note 1297, U.S. Government Printing Office, Washington, DC, (1994).

BEACON ENVIRONMENTAL
2203A Commerce Road, Suite 1
Forest Hill, MD 21050 USA
1.410.838.8780

CERTIFICATE OF ANALYSIS

**University of Notre Dame**	**Site Name:** So-Cal Military Toxic Site	**Beacon Proposal:** 201201H01
Department of Biological Sciences	**Site Location:** Notspa, CA	**Lab Work Order:** 0005542
South Bend, IN 46556	**Project Manager:** Kristin Shrader-Frechette	**Reported:** 01/25/2021

Vendor: Absolute Standards, Inc. **Lab Standard No.:** 2000092
Lot No.: 021820

Absolute Standards, Inc.
800-368-1131
www.absolutestandards.com

Certified Reference Material CRM

ANAB ISO 17034 Accredited
AR-1539 Certificate Number
https://Absolutestandards.com

CERTIFIED WEIGHT REPORT

Part Number: 97255
Lot Number: 021820
Description: 8260 VOC Primary Calibration Level 3
84 components
Expiration Date: 021823
Recommended Storage: Freezer (0 °C)
Nominal Concentration (µg/mL): 50
NIST Test ID#: 6UTB
Volume(s) shown below were combined and diluted to (mL): 100.0

Solvent(s): Methanol **Lot#** DV182-USQ12

5E-05 Balance Uncertainty
0.012 Flask Uncertainty

Gabriel Helland (signature)			021820
Formulated By:	Gabriel Helland		DATE
(signature)			021820
Reviewed By:	Pedro L. Rentas		DATE

	Compound	Part Number	Lot Number	Dil. Factor	Initial Vol. (mL)	Uncertainty Pipette (mL)	Initial Conc.(ug/mL)	Final Conc.(ug/mL)	Expanded Uncertainty (+/-) (µg/mL)	SDS Information (Solvent Safety Info. On Attached pg.) CAS#	OSHA PEL (TWA)	LD50
1.	Bromodichloromethane	93538	043019	0.025	2.50	0.017	1999.8	50.0	0.9	75-27-4	N/A	orl-rat 916mg/kg
2.	Dibromochloromethane	93538	043019	0.025	2.50	0.017	1999.8	50.0	0.9	124-48-1	N/A	orl-rat 848mg/kg
3.	cis-1,2-Dichloroethene	93538	043019	0.025	2.50	0.017	1999.9	50.0	0.9	156-59-2	N/A	N/A
4.	trans-1,2-Dichloroethene	93538	043019	0.025	2.50	0.017	1999.8	50.0	0.9	156-60-5	N/A	orl-rat 1235mg/kg
5.	Methylene chloride	93538	043019	0.025	2.50	0.017	1999.9	50.0	0.9	75-09-2	500 ppm	orl-rat 820mg/kg
6.	1,1-Dichloroethene	93538	043019	0.025	2.50	0.017	2000.3	50.0	0.9	75-35-4	1 ppm (4mg/m3/8H)	orl-rat 200mg/kg
7.	Bromochloromethane	93538	043019	0.025	2.50	0.017	2000.0	50.0	0.9	74-97-5	200 ppm (1050mg/m3/8H)	orl-rat 5000mg/kg
8.	Bromoform	93538	043019	0.025	2.50	0.017	2000.0	50.0	0.9	75-25-2	0.5 ppm (5mg/m3) (skin)	orl-rat 933mg/kg
9.	Carbon tetrachloride	93538	043019	0.025	2.50	0.017	2000.0	50.0	0.9	56-23-5	2 ppm (12.6mg/m3/8H)	orl-rat 2350mg/kg
10.	Chloroform	93538	043019	0.025	2.50	0.017	2000.0	50.0	0.9	67-66-3	50 ppm (240mg/m3) (CL)	orl-rat 908mg/kg
11.	Dibromomethane	93538	043019	0.025	2.50	0.017	2000.0	50.0	0.9	74-95-3	N/A	orl-rat 108mg/kg
12.	1,1-Dichloroethane	93538	043019	0.025	2.50	0.017	1999.9	50.0	0.9	75-34-3	100 ppm	orl-rat 725mg/kg
13.	2,2-Dichloropropane	93538	043019	0.025	2.50	0.017	2000.0	50.0	0.9	594-20-7	N/A	N/A
14.	Tetrachloroethene	93538	043019	0.025	2.50	0.017	2000.0	50.0	0.9	127-18-4	25 ppm (170mg/m3/8H)(final)	orl-rat 2629mg/kg
15.	1,1,1-Trichloroethane	93538	043019	0.025	2.50	0.017	1999.9	50.0	0.9	71-55-6	350 ppm (1900mg/m3/8H)	orl-rat 10300mg/kg
16.	1,2-Dibromo-3-chloropropane	93538	043019	0.025	2.50	0.017	1999.8	50.0	0.9	96-12-8	0.001 ppm	orl-rat 170mg/kg
17.	1,2-Dibromoethane	93538	043019	0.025	2.50	0.017	1999.9	50.0	0.9	106-93-4	20 ppm (8H)	orl-rat 108mg/kg
18.	1,2-Dichloroethane	93538	043019	0.025	2.50	0.017	1999.9	50.0	0.9	107-06-2	50 ppm (8H)	orl-rat 670mg/kg
19.	1,2-Dichloropropane	93538	043019	0.025	2.50	0.017	1999.9	50.0	0.9	78-87-5	75 ppm (350mg/m3/8H)	orl-rat 1947mg/kg
20.	1,3-Dichloropropane	93538	043019	0.025	2.50	0.017	1999.8	50.0	0.9	142-28-9	N/A	unr-mus 3600mg/kg
21.	1,1-Dichloropropene	93538	043019	0.025	2.50	0.017	1981.8	49.5	1.0	563-58-6	N/A	N/A
22.	cis-1,3-Dichloropropene	93538	043019	0.025	2.50	0.017	1999.8	50.0	0.9	10061-01-5	N/A	N/A
23.	trans-1,3-Dichloropropene	93538	043019	0.025	2.50	0.017	1999.8	50.0	0.9	10061-02-6	N/A	N/A
24.	Hexachloro-1,3-butadiene	93538	043019	0.025	2.50	0.017	1999.8	50.0	0.9	87-68-3	0.02 ppm (0.24mg/m3/8H)	orl-rat 82mg/kg
25.	1,1,1,2-Tetrachloroethane	93538	043019	0.025	2.50	0.017	1999.8	50.0	0.9	630-20-6	N/A	orl-rat 670mg/kg
26.	1,1,2,2-Tetrachloroethane	93538	043019	0.025	2.50	0.017	1999.9	50.0	0.9	79-34-5	5 ppm (35mg/m3/9H)(skin)	orl-rat 800mg/kg
27.	1,1,2-Trichloroethane	93538	043019	0.025	2.50	0.017	1999.8	50.0	0.9	79-00-5	10 ppm (45mg/m3/8H)(skin)	orl-rat 836mg/kg
28.	Trichloroethene	93538	043019	0.025	2.50	0.017	1999.8	50.0	0.9	79-01-6	50 ppm (270mg/m3/8H)	orl-mus 2402mg/kg
29.	1,2,3-Trichloropropane	93538	043019	0.025	2.50	0.017	1999.8	50.0	0.9	96-18-4	10 ppm (60mg/m3/8H)	orl-rat 149.6mg/kg
30.	Benzene	93538	043019	0.025	2.50	0.017	2000.0	50.0	0.9	71-43-2	1 ppm	orl-rat 4894mg/kg
31.	Bromobenzene	93538	043019	0.025	2.50	0.017	2000.2	50.0	0.9	108-86-1	N/A	orl-rat 2699mg/kg
32.	n-Butyl benzene	93538	043019	0.025	2.50	0.017	2000.9	50.0	0.9	104-51-8	N/A	N/A
33.	Ethyl benzene	93538	043019	0.025	2.50	0.017	2000.0	50.0	0.9	100-41-4	100 ppm (435mg/m3/8H)	orl-rat >2000mg/kg
34.	p-Isopropyl toluene	93538	043019	0.025	2.50	0.017	2000.3	50.0	0.9	99-87-6	N/A	orl-rat 4750mg/kg
35.	Naphthalene	93538	043019	0.025	2.50	0.017	2000.1	50.0	0.9	91-20-3	10 ppm (50mg/m3/8H)	orl-rat 490mg/kg
36.	Toluene	93538	043019	0.025	2.50	0.017	2000.1	50.0	0.9	108-88-3	200 ppm	orl-rat 5000mg/kg
37.	1,2,3-Trichlorobenzene	93538	043019	0.025	2.50	0.017	2001.1	50.0	0.9	87-61-6	N/A	ipr-mus 1390mg/kg
38.	1,2,4-Trichlorobenzene	93538	043019	0.025	2.50	0.017	2000.7	50.0	0.9	120-82-1	5 ppm (CL) (40mg/m3)	orl-rat 756mg/kg
39.	1,2,4-Trimethylbenzene	93538	043019	0.025	2.50	0.017	2000.6	50.0	0.9	95-63-6	N/A	orl-rat 5g/kg
40.	1,3,5-Trimethylbenzene	93538	043019	0.025	2.50	0.017	2000.4	50.0	0.9	108-67-8	N/A	N/A
41.	Styrene	93538	043019	0.025	2.50	0.017	2000.0	50.0	0.9	100-42-5	100 ppm	orl-rat 5000mg/kg
42.	tert-Butyl benzene	93538	043019	0.025	2.50	0.017	2000.2	50.0	0.9	98-06-6	N/A	N/A
43.	sec-Butyl benzene	93538	043019	0.025	2.50	0.017	2000.4	50.0	0.9	135-98-8	N/A	orl-rat 2240mg/kg
44.	Chlorobenzene	93538	043019	0.025	2.50	0.017	2000.6	50.0	0.9	108-90-7	75 ppm (350mg/m3/8H)	orl-rat 2290mg/kg
45.	2-Chlorotoluene	93538	043019	0.025	2.50	0.017	2000.1	50.0	0.9	95-49-8	50 ppm (250mg/m3/8H)	orl-rat 3900mg/kg
46.	4-Chlorotoluene	93538	043019	0.025	2.50	0.017	2000.3	50.0	0.9	106-43-4	N/A	orl-rat 2100mg/kg
47.	1,2-Dichlorobenzene	93538	043019	0.025	2.50	0.017	2000.6	50.0	0.9	95-50-1	50 ppm (300mg/m3) (CL)	orl-rat 500mg/kg
48.	1,3-Dichlorobenzene	93538	043019	0.025	2.50	0.017	2000.5	50.0	0.9	541-73-1	N/A	ipr-mus 1062mg/kg
49.	1,4-Dichlorobenzene	93538	043019	0.025	2.50	0.017	2000.3	50.0	0.9	106-46-7	75 ppm (450mg/m3/8H)	orl-rat 500mg/kg
50.	Isopropylbenzene	93538	043019	0.025	2.50	0.017	2000.6	50.0	0.9	98-82-8	50 ppm (245mg/m3/8H)	orl-rat 1400mg/kg
51.	n-Propylbenzene	93538	043019	0.025	2.50	0.017	2000.4	50.0	0.9	103-65-1	N/A	orl-rat 6040mg/kg
52.	o-Xylene	93538	043019	0.025	2.50	0.017	2000.0	50.0	0.9	95-47-6	100 ppm (435mg/m3/8H)	ipr-mus 1364mg/kg
53.	m-Xylene	93538	043019	0.025	2.50	0.017	1000.0	**25.0**	0.5	108-38-3	100 ppm (435mg/m3/8H)	orl-rat 5g/kg
54.	p-Xylene	93538	043019	0.025	2.50	0.017	999.9	**25.0**	0.5	106-42-3	100 ppm (435mg/m3/8H)	orl-rat 5g/kg
55.	Carbon disulphide	97233	021720	0.025	2.50	0.017	2000.6	50.0	0.9	75-15-0	4 ppm (12mg/m3) (skin)	orl-rat 1200mg/kg
56.	1,4-Dioxane	97233	021720	0.025	2.50	0.017	2000.8	50.0	0.9	123-91-1	25 ppm (90mg/m3/8H)(skin)	orl-mus 5700mg/kg
57.	Hexachloroethane	97233	021720	0.025	2.50	0.017	2000.7	50.0	0.9	67-72-1	1 ppm (10mg/m3/8H)(skin)	orl-gpg 4970mg/kg
58.	Methyl tert-butyl ether (MTBE)	97233	021720	0.025	2.50	0.017	2000.7	50.0	0.9	1634-04-4	N/A	orl-rat 4g/kg
59.	2-Methylnaphthalene	97233	021720	0.025	2.50	0.017	2000.4	50.0	0.9	91-57-6	N/A	orl-rat 1630mg/kg
60.	1,1,2-Trichlorotrifluoroethane	97233	021720	0.025	2.50	0.017	2000.9	50.0	0.9	76-13-1	1000 ppm (7600mg/m3/8H)	orl-rat 43g/kg
61.	n-Pentane	97235	120114	0.025	2.50	0.017	2002.6	50.1	0.9	109-66-0	600 ppm(1800mg/m3/8H)	ivn-mus 446mg/kg
62.	n-Hexane	97235	120114	0.025	2.50	0.017	2002.6	50.1	0.9	110-54-3	50 ppm(180mg/m3/8H)	orl-rat 28710mg/kg
63.	n-Heptane	97235	120114	0.025	2.50	0.017	2003.1	50.1	0.9	142-82-5	400 ppm(1600mg/m3/8H)	ivn-mus 222mg/kg
64.	n-Octane	97235	120114	0.025	2.50	0.017	2001.6	50.0	0.9	111-65-9	300 ppm(1450mg/m3/8H)	N/A
65.	n-Nonane	97235	120114	0.025	2.50	0.017	2000.7	50.0	0.9	111-84-2	200 ppm (1050mg/m3/8H)	ivn-mus 218mg/kg
66.	n-Decane	97235	120114	0.025	2.50	0.017	2001.8	50.0	0.9	124-18-5	N/A	N/A
67.	n-Undecane	97235	120114	0.025	2.50	0.017	2003.6	50.1	0.9	1120-21-4	N/A	ivn-mus 517mg/kg
68.	n-Dodecane	97235	120114	0.025	2.50	0.017	2000.6	50.0	0.9	112-40-3	N/A	N/A
69.	n-Tridecane	97235	120114	0.025	2.50	0.017	2000.2	50.0	0.8	629-50-5	N/A	ivn-mus 1161mg/kg
70.	n-Tetradecane	97235	120114	0.025	2.50	0.017	2001.5	50.0	0.9	629-59-4	N/A	N/A
71.	n-Pentadecane	97235	120114	0.025	2.50	0.017	2001.3	50.0	0.9	629-62-9	N/A	ivn-mus 3494mg/kg
72.	Bromomethane	30058	123019	0.025	2.50	0.017	2004.8	50.1	0.9	74-83-9	5 ppm (20mg/m3/8H) (skin)	orl-rat 214mg/kg
73.	Chloroethane	30058	123019	0.025	2.50	0.017	2002.5	50.1	0.9	75-00-3	1000 ppm (2600mg/m3/8H)	N/A
74.	Chloromethane	30058	123019	0.025	2.50	0.017	2003.0	50.1	0.9	74-87-3	100 ppm	orl-rat 1800mg/kg
75.	Dichlorodifluoromethane	30058	123019	0.025	2.50	0.017	2034.9	50.9	0.9	75-71-8	1000 ppm (4950mg/m3/8H)	N/A
76.	Trichlorofluoromethane	30058	123019	0.025	2.50	0.017	2009.6	50.2	0.9	75-69-4	1000 ppm (5600mg/m3/8H)	ipr-mus 1743mg/kg
77.	Vinyl chloride	30058	123019	0.025	2.50	0.017	2001.2	50.0	0.8	75-01-4	N/A	N/A
78.	4-Methyl-2-pentanone	97259	112816	0.025	2.50	0.017	2000.1	50.0	0.9	108-10-1	100 ppm (410mg/m3/8H)	orl-rat 2080mg/kg
79.	p-Bromofluorobenzene	20002	020419	0.025	2.50	0.017	2000.7	50.0	0.8	460-00-4	N/A	orl-rat 2700mg/kg
80.	1,2-Dichloroethane-d4	20002	020419	0.025	2.50	0.017	2000.6	50.0	0.8	17060-07-0	N/A	orl-mus 625mg/kg
81.	Toluene-d8	20002	020419	0.025	2.50	0.017	2000.4	50.0	0.8	2037-26-5	200 ppm	orl-rat 5000mg/kg
82.	Chlorobenzene-d5	22013	021720	0.10	10.00	0.042	2001.6	**200.1**	1.9	3114-55-4	N/A	orl-rat 1110mg/kg
83.	1,4-Dichlorobenzene-d4	22013	021720	0.10	10.00	0.042	2002.0	**200.2**	1.9	3855-82-1	N/A	orl-rat 500mg/kg
84.	Fluorobenzene	22013	021720	0.10	10.00	0.042	2002.1	**200.2**	1.9	462-06-6	N/A	orl-rat 4399mg/kg

- The certified value is the concentration calculated from gravimetric and volumetric measurements unless otherwise stated.
- Standards are prepared gravimetrically using balances that are calibrated with weights traceable to NIST (see above).
- Standards are certifed (+/-) 0.5% of the stated value, unless otherwise stated.
- All Standards, after opening ampule, should be stored with caps tight and under appropriate laboratory conditions.
- Uncertainty Reference: Taylor, B.N. and Kuyat, C.E., "Guidelines for Evaluating and Expressing the Uncertainty of NIST Measurement Result," NIST Technical Note 1297, U.S. Government Printing Office, Washington, DC, (1994).

BEACON ENVIRONMENTAL
2203A Commerce Road, Suite 1
Forest Hill, MD 21050 USA
1.410.838.8780

CERTIFICATE OF ANALYSIS

**University of Notre Dame**	**Site Name:** So-Cal Military Toxic Site	**Beacon Proposal:** 201201H01
Department of Biological Sciences	**Site Location:** Notspa, CA	**Lab Work Order:** 0005542
South Bend, IN 46556	**Project Manager:** Kristin Shrader-Frechette	**Reported:** 01/25/2021

Vendor: Absolute Standards, Inc. **Lab Standard No.:** 2000093

Lot No.: 021820

Absolute Standards, Inc.
800-368-1131
www.absolutestandards.com

Certified Reference Material CRM

ANAB ISO 17034 Accredited
AR-1539 Certificate Number
https://Absolutestandards.com

CERTIFIED WEIGHT REPORT

Part Number: 97256
Lot Number: 021820
Description: 8260 VOC Primary Calibration Level 4
84 components
Expiration Date: 021823 **Solvent(s):** Methanol **Lot#** DV182-USQ12
Recommended Storage: Freezer (0 °C)
Nominal Concentration (µg/mL): 100
NIST Test ID#: 6UTB 5E-05 Balance Uncertainty
Volume(s) shown below were combined and diluted to (mL): 100.0 0.012 Flask Uncertainty

021820	Formulated By: Gabriel Helland	DATE
021820	Reviewed By: Pedro L. Rentas	DATE

SDS Information (Solvent Safety Info. On Attached pg.)

	Compound	Part Number	Lot Number	Dil. Factor	Initial Vol. (mL)	Uncertainty Pipette (mL)	Initial Conc.(ug/mL)	Final Conc.(ug/mL)	Expanded Uncertainty (+/-) (µg/mL)	CAS#	OSHA PEL (TWA)	LD50
1.	Bromodichloromethane	93538	043019	0.05	5.00	0.017	1999.8	100.0	1.1	75-27-4	N/A	orl-rat 916mg/kg
2.	Dibromochloromethane	93538	043019	0.05	5.00	0.017	1999.8	100.0	1.1	124-48-1	N/A	orl-rat 848mg/kg
3.	cis-1,2-Dichloroethene	93538	043019	0.05	5.00	0.017	1999.9	100.0	1.1	156-59-2	N/A	N/A
4.	trans-1,2-Dichloroethene	93538	043019	0.05	5.00	0.017	1999.8	100.0	1.1	156-60-5	N/A	orl-rat 1235mg/kg
5.	Methylene chloride	93538	043019	0.05	5.00	0.017	1999.9	100.0	1.1	75-09-2	500 ppm	orl-rat 820mg/kg
6.	1,1-Dichloroethene	93538	043019	0.05	5.00	0.017	2000.3	100.0	1.2	75-35-4	1 ppm (4mg/m3/8H)	orl-rat 200mg/kg
7.	Bromochloromethane	93538	043019	0.05	5.00	0.017	2000.0	100.0	1.1	74-97-5	200 ppm (1050mg/m3/8H)	orl-rat 5000mg/kg
8.	Bromoform	93538	043019	0.05	5.00	0.017	2000.0	100.0	1.1	75-25-2	0.5 ppm (5mg/m3) (skin)	orl-rat 933mg/kg
9.	Carbon tetrachloride	93538	043019	0.05	5.00	0.017	2000.0	100.0	1.1	56-23-5	2 ppm (12.6mg/m3/8H)	orl-rat 2350mg/kg
10.	Chloroform	93538	043019	0.05	5.00	0.017	2000.0	100.0	1.1	67-66-3	50 ppm (240mg/m3) (CL)	orl-rat 908mg/kg
11.	Dibromomethane	93538	043019	0.05	5.00	0.017	2000.0	100.0	1.1	74-95-3	N/A	orl-rat 108mg/kg
12.	1,1-Dichloroethane	93538	043019	0.05	5.00	0.017	1999.9	100.0	1.1	75-34-3	100 ppm	orl-rat 725mg/kg
13.	2,2-Dichloropropane	93538	043019	0.05	5.00	0.017	2000.0	100.0	1.1	594-20-7	N/A	N/A
14.	Tetrachloroethene	93538	043019	0.05	5.00	0.017	2000.0	100.0	1.1	127-18-4	25 ppm (170mg/m3/8H)(final)	orl-rat 2629mg/kg
15.	1,1,1-Trichloroethane	93538	043019	0.05	5.00	0.017	1999.9	100.0	1.1	71-55-6	350 ppm (1900mg/m3/8H)	orl-rat 10300mg/kg
16.	1,2-Dibromo-3-chloropropane	93538	043019	0.05	5.00	0.017	1999.8	100.0	1.1	96-12-8	0.001 ppm	orl-rat 170mg/kg
17.	1,2-Dibromoethane	93538	043019	0.05	5.00	0.017	1999.9	100.0	1.1	106-93-4	20 ppm (8H)	orl-rat 108mg/kg
18.	1,2-Dichloroethane	93538	043019	0.05	5.00	0.017	1999.9	100.0	1.1	107-06-2	50 ppm (8H)	orl-rat 670mg/kg
19.	1,2-Dichloropropane	93538	043019	0.05	5.00	0.017	1999.9	100.0	1.1	78-87-5	75 ppm (350mg/m3/8H)	orl-rat 1947mg/kg
20.	1,3-Dichloropropane	93538	043019	0.05	5.00	0.017	1999.8	100.0	1.1	142-28-9	N/A	unr-mus 3600mg/kg
21.	1,1-Dichloropropene	93538	043019	0.05	5.00	0.017	1981.8	99.1	1.5	563-58-6	N/A	N/A
22.	cis-1,3-Dichloropropene	93538	043019	0.05	5.00	0.017	1999.8	100.0	1.1	10061-01-5	N/A	N/A
23.	trans-1,3-Dichloropropene	93538	043019	0.05	5.00	0.017	1999.8	100.0	1.1	10061-02-6	N/A	N/A
24.	Hexachloro-1,3-butadiene	93538	043019	0.05	5.00	0.017	1999.8	100.0	1.1	87-68-3	0.02 ppm (0.24mg/m3/8H)	orl-rat 82mg/kg
25.	1,1,1,2-Tetrachloroethane	93538	043019	0.05	5.00	0.017	1999.8	100.0	1.1	630-20-6	N/A	orl-rat 670mg/kg
26.	1,1,2,2-Tetrachloroethane	93538	043019	0.05	5.00	0.017	1999.9	100.0	1.1	79-34-5	5 ppm (35mg/m3/9H)(skin)	orl-rat 800mg/kg
27.	1,1,2-Trichloroethane	93538	043019	0.05	5.00	0.017	1999.8	100.0	1.1	79-00-5	10 ppm (45mg/m3/8H)(skin)	orl-rat 836mg/kg
28.	Trichloroethene	93538	043019	0.05	5.00	0.017	1999.8	100.0	1.1	79-01-6	50 ppm (270mg/m3/8H)	orl-mus 2402mg/kg
29.	1,2,3-Trichloropropane	93538	043019	0.05	5.00	0.017	1999.8	100.0	1.1	96-18-4	10 ppm (60mg/m3/8H)	orl-rat 149.6mg/kg
30.	Benzene	93538	043019	0.05	5.00	0.017	2000.0	100.0	1.2	71-43-2	1 ppm	orl-rat 4894mg/kg
31.	Bromobenzene	93538	043019	0.05	5.00	0.017	2000.2	100.0	1.2	108-86-1	N/A	orl-rat 2699mg/kg
32.	n-Butyl benzene	93538	043019	0.05	5.00	0.017	2000.9	100.0	1.2	104-51-8	N/A	N/A
33.	Ethyl benzene	93538	043019	0.05	5.00	0.017	2000.0	100.0	1.2	100-41-4	100 ppm (435mg/m3/8H)	orl-rat >2000mg/kg
34.	p-Isopropyl toluene	93538	043019	0.05	5.00	0.017	2000.3	100.0	1.2	99-87-6	N/A	orl-rat 4750mg/kg
35.	Naphthalene	93538	043019	0.05	5.00	0.017	2000.1	100.0	1.2	91-20-3	10 ppm (50mg/m3/8H)	orl-rat 490mg/kg
36.	Toluene	93538	043019	0.05	5.00	0.017	2000.1	100.0	1.2	108-88-3	200 ppm	orl-rat 5000mg/kg
37.	1,2,3-Trichlorobenzene	93538	043019	0.05	5.00	0.017	2001.1	100.0	1.2	87-61-6	N/A	ipr-mus 1390mg/kg
38.	1,2,4-Trichlorobenzene	93538	043019	0.05	5.00	0.017	2000.7	100.0	1.2	120-82-1	5 ppm (CL) (40mg/m3)	orl-rat 756mg/kg
39.	1,2,4-Trimethylbenzene	93538	043019	0.05	5.00	0.017	2000.6	100.0	1.2	95-63-6	N/A	orl-rat 5g/kg
40.	1,3,5-Trimethylbenzene	93538	043019	0.05	5.00	0.017	2000.4	100.0	1.2	108-67-8	N/A	N/A
41.	Styrene	93538	043019	0.05	5.00	0.017	2000.0	100.0	1.2	100-42-5	100 ppm	orl-rat 5000mg/kg
42.	tert-Butyl benzene	93538	043019	0.05	5.00	0.017	2000.2	100.0	1.2	98-06-6	N/A	N/A
43.	sec-Butyl benzene	93538	043019	0.05	5.00	0.017	2000.4	100.0	1.2	135-98-8	N/A	orl-rat 2240mg/kg
44.	Chlorobenzene	93538	043019	0.05	5.00	0.017	2000.6	100.0	1.2	108-90-7	75 ppm (350mg/m3/8H)	orl-rat 2290mg/kg
45.	2-Chlorotoluene	93538	043019	0.05	5.00	0.017	2000.1	100.0	1.2	95-49-8	50 ppm (250mg/m3/8H)	orl-rat 3900mg/kg
46.	4-Chlorotoluene	93538	043019	0.05	5.00	0.017	2000.3	100.0	1.2	106-43-4	N/A	orl-rat 2100mg/kg
47.	1,2-Dichlorobenzene	93538	043019	0.05	5.00	0.017	2000.6	100.0	1.2	95-50-1	50 ppm (300mg/m3) (CL)	orl-rat 500mg/kg
48.	1,3-Dichlorobenzene	93538	043019	0.05	5.00	0.017	2000.5	100.0	1.2	541-73-1	N/A	ipr-mus 1062mg/kg
49.	1,4-Dichlorobenzene	93538	043019	0.05	5.00	0.017	2000.3	100.0	1.2	106-46-7	75 ppm (450mg/m3/8H)	orl-rat 500mg/kg
50.	Isopropylbenzene	93538	043019	0.05	5.00	0.017	2000.6	100.0	1.2	98-82-8	50 ppm (245mg/m3/8H)	orl-rat 1400mg/kg
51.	n-Propylbenzene	93538	043019	0.05	5.00	0.017	2000.4	100.0	1.2	103-65-1	N/A	orl-rat 6040mg/kg
52.	o-Xylene	93538	043019	0.05	5.00	0.017	2000.0	100.0	1.2	95-47-6	100 ppm (435mg/m3/8H)	ipr-mus 1364mg/kg
53.	m-Xylene	93538	043019	0.05	5.00	0.017	1000.0	**50.0**	0.6	108-38-3	100 ppm (435mg/m3/8H)	orl-rat 5g/kg
54.	p-Xylene	93538	043019	0.05	5.00	0.017	999.9	**50.0**	0.6	106-42-3	100 ppm (435mg/m3/8H)	orl-rat 5g/kg
55.	Carbon disulphide	97269	020720	0.005	0.50	0.004	20004.9	100.0	5.0	75-15-0	4 ppm (12mg/m3) (skin)	orl-rat 1200mg/kg
56.	1,4-Dioxane	97269	020720	0.005	0.50	0.004	20006.9	100.0	5.0	123-91-1	25 ppm (90mg/m3/8H)(skin)	orl-mus 5700mg/kg
57.	Hexachloroethane	97269	020720	0.005	0.50	0.004	20005.9	100.0	5.0	67-72-1	1 ppm (10mg/m3/8H)(skin)	orl-gpg 4970mg/kg
58.	Methyl tert-butyl ether (MTBE)	97269	020720	0.005	0.50	0.004	20005.9	100.0	5.0	1634-04-4	N/A	orl-rat 4g/kg
59.	2-Methylnaphthalene	97269	020720	0.005	0.50	0.004	20003.3	100.0	5.0	91-57-6	N/A	orl-rat 1630mg/kg
60.	1,1,2-Trichlorotrifluoroethane	97269	020720	0.005	0.50	0.004	20007.9	100.0	5.0	76-13-1	1000 ppm (7600mg/m3/8H)	orl-rat 43g/kg
61.	n-Pentane	97235	120114	0.05	5.00	0.017	2002.6	100.1	0.9	109-66-0	600 ppm(1800mg/m3/8H)	ivn-mus 446mg/kg
62.	n-Hexane	97235	120114	0.05	5.00	0.017	2002.6	100.1	0.9	110-54-3	50 ppm(180mg/m3/8H)	orl-rat 28710mg/kg
63.	n-Heptane	97235	120114	0.05	5.00	0.017	2003.1	100.2	0.9	142-82-5	400 ppm(1600mg/m3/8H)	ivn-mus 222mg/kg
64.	n-Octane	97235	120114	0.05	5.00	0.017	2001.6	100.1	0.9	111-65-9	300 ppm(1450mg/m3/8H)	N/A
65.	n-Nonane	97235	120114	0.05	5.00	0.017	2000.7	100.0	0.9	111-84-2	200 ppm (1050mg/m3/8H)	ivn-mus 218mg/kg
66.	n-Decane	97235	120114	0.05	5.00	0.017	2001.8	100.1	0.9	124-18-5	N/A	N/A
67.	n-Undecane	97235	120114	0.05	5.00	0.017	2003.6	100.2	0.9	1120-21-4	N/A	ivn-mus 517mg/kg
68.	n-Dodecane	97235	120114	0.05	5.00	0.017	2000.6	100.0	0.9	112-40-3	N/A	N/A
69.	n-Tridecane	97235	120114	0.05	5.00	0.017	2000.2	100.0	0.9	629-50-5	N/A	ivn-mus 1161mg/kg
70.	n-Tetradecane	97235	120114	0.05	5.00	0.017	2001.5	100.1	0.9	629-59-4	N/A	N/A
71.	n-Pentadecane	97235	120114	0.05	5.00	0.017	2001.3	100.1	0.9	629-62-9	N/A	ivn-mus 3494mg/kg
72.	Bromomethane	30058	123019	0.05	5.00	0.017	2004.8	100.2	0.9	74-83-9	5 ppm (20mg/m3/8H) (skin)	orl-rat 214mg/kg
73.	Chloroethane	30058	123019	0.05	5.00	0.017	2002.5	100.1	0.9	75-00-3	1000 ppm (2600mg/m3/8H)	N/A
74.	Chloromethane	30058	123019	0.05	5.00	0.017	2003.0	100.1	0.9	74-87-3	100 ppm	orl-rat 1800mg/kg
75.	Dichlorodifluoromethane	30058	123019	0.05	5.00	0.017	2034.9	101.7	0.9	75-71-8	1000 ppm (4950mg/m3/8H)	N/A
76.	Trichlorofluoromethane	30058	123019	0.05	5.00	0.017	2009.6	100.5	0.9	75-69-4	1000 ppm (5600mg/m3/8H)	ipr-mus 1743mg/kg
77.	Vinyl chloride	30058	123019	0.05	5.00	0.017	2001.2	100.1	0.9	75-01-4	N/A	N/A
78.	4-Methyl-2-pentanone (MIBK)	82442	112816	0.00	0.50	0.004	20003.5	100.0	5.0	108-10-1	100 ppm (410mg/m3/8H)	orl-rat 2080mg/kg
79.	p-Bromofluorobenzene	20002	020419	0.05	5.00	0.017	2000.7	100.0	0.9	460-00-4	N/A	orl-rat 2700mg/kg
80.	1,2-Dichloroethane-d4	20002	020419	0.05	5.00	0.017	2000.6	100.0	0.9	17060-07-0	N/A	orl-mus 625mg/kg
81.	Toluene-d8	20002	020419	0.05	5.00	0.017	2000.4	100.0	0.9	2037-26-5	200 ppm	orl-rat 5000mg/kg
82.	Chlorobenzene-d5	22013	021720	0.10	10.00	0.042	2001.6	**200.1**	1.9	3114-55-4	N/A	orl-rat 1110mg/kg
83.	1,4-Dichlorobenzene-d4	22013	021720	0.10	10.00	0.042	2002.0	**200.2**	1.9	3855-82-1	N/A	orl-rat 500mg/kg
84.	Fluorobenzene	22013	021720	0.10	10.00	0.042	2002.1	**200.2**	1.9	462-06-6	N/A	orl-rat 4399mg/kg

- The certified value is the concentration calculated from gravimetric and volumetric measurements unless otherwise stated.
- Standards are prepared gravimetrically using balances that are calibrated with weights traceable to NIST (see above).
- Standards are certifed (+/-) 0.5% of the stated value, unless otherwise stated.
- All Standards, after opening ampule, should be stored with caps tight and under appropriate laboratory conditions.
- Uncertainty Reference: Taylor, B.N. and Kuyat, C.E., "Guidelines for Evaluating and Expressing the Uncertainty of NIST Measurement Result," NIST Technical Note 1297, U.S. Government Printing Office, Washington, DC, (1994).

2203A Commerce Road, Suite 1
Forest Hill, MD 21050 USA
1.410.838.8780

CERTIFICATE OF ANALYSIS

**University of Notre Dame**	**Site Name:** So-Cal Military Toxic Site	**Beacon Proposal:** 201201H01
Department of Biological Sciences	**Site Location:** Notspa, CA	**Lab Work Order:** 0005542
South Bend, IN 46556	**Project Manager:** Kristin Shrader-Frechette	**Reported:** 01/25/2021

Vendor: Absolute Standards, Inc. **Lab Standard No.:** 2000094
Lot No.: 021820

Absolute Standards, Inc.
800-368-1131
www.absolutestandards.com

Certified Reference Material CRM

ANAB ISO 17034 Accredited
AR-1539 Certificate Number
https://Absolutestandards.com

CERTIFIED WEIGHT REPORT

Part Number: 97257
Lot Number: 021820
Description: 8260 VOC Primary Calibration Level 5
84 components
Expiration Date: 021823 **Solvent(s):** Methanol **Lot#** DV182-USQ12
Recommended Storage: Freezer (0 °C)
Nominal Concentration (µg/mL): 200
NIST Test ID#: 6UTB 5E-05 Balance Uncertainty
Volume(s) shown below were combined and diluted to (mL): 50.0 0.007 Flask Uncertainty

Gabriel Helland		021820
Formulated By:	Gabriel Helland	DATE
Pedro Rentas		021820
Reviewed By:	Pedro L. Rentas	DATE

	Compound	Part Number	Lot Number	Dil. Factor	Initial Vol. (mL)	Uncertainty Pipette (mL)	Initial Conc.(ug/mL)	Final Conc.(ug/mL)	Expanded Uncertainty (+/-) (µg/mL)	CAS#	SDS Information (Solvent Safety Info. On Attached pg.) OSHA PEL (TWA)	LD50
1.	Bromodichloromethane	93538	043019	0.10	5.00	0.017	1999.8	200.0	2.2	75-27-4	N/A	orl-rat 916mg/kg
2.	Dibromochloromethane	93538	043019	0.10	5.00	0.017	1999.8	200.0	2.2	124-48-1	N/A	orl-rat 848mg/kg
3.	cis-1,2-Dichloroethene	93538	043019	0.10	5.00	0.017	1999.9	200.0	2.2	156-59-2	N/A	N/A
4.	trans-1,2-Dichloroethene	93538	043019	0.10	5.00	0.017	1999.8	200.0	2.2	156-60-5	N/A	orl-rat 1235mg/kg
5.	Methylene chloride	93538	043019	0.10	5.00	0.017	1999.9	200.0	2.2	75-09-2	500 ppm	orl-rat 820mg/kg
6.	1,1-Dichloroethene	93538	043019	0.10	5.00	0.017	2000.3	200.0	2.4	75-35-4	1 ppm (4mg/m3/8H)	orl-rat 200mg/kg
7.	Bromochloromethane	93538	043019	0.10	5.00	0.017	2000.0	200.0	2.2	74-97-5	200 ppm (1050mg/m3/8H)	orl-rat 5000mg/kg
8.	Bromoform	93538	043019	0.10	5.00	0.017	2000.0	200.0	2.2	75-25-2	0.5 ppm (5mg/m3) (skin)	orl-rat 933mg/kg
9.	Carbon tetrachloride	93538	043019	0.10	5.00	0.017	2000.0	200.0	2.2	56-23-5	2 ppm (12.6mg/m3/8H)	orl-rat 2350mg/kg
10.	Chloroform	93538	043019	0.10	5.00	0.017	2000.0	200.0	2.1	67-66-3	50 ppm (240mg/m3) (CL)	orl-rat 908mg/kg
11.	Dibromomethane	93538	043019	0.10	5.00	0.017	2000.0	200.0	2.2	74-95-3	N/A	orl-rat 108mg/kg
12.	1,1-Dichloroethane	93538	043019	0.10	5.00	0.017	1999.9	200.0	2.2	75-34-3	100 ppm	orl-rat 725mg/kg
13.	2,2-Dichloropropane	93538	043019	0.10	5.00	0.017	2000.0	200.0	2.2	594-20-7	N/A	N/A
14.	Tetrachloroethene	93538	043019	0.10	5.00	0.017	2000.0	200.0	2.2	127-18-4	25 ppm (170mg/m3/8H)(final)	orl-rat 2629mg/kg
15.	1,1,1-Trichloroethane	93538	043019	0.10	5.00	0.017	1999.9	200.0	2.2	71-55-6	350 ppm (1900mg/m3/8H)	orl-rat 10300mg/kg
16.	1,2-Dibromo-3-chloropropane	93538	043019	0.10	5.00	0.017	1999.8	200.0	2.2	96-12-8	0.001 ppm	orl-rat 170mg/kg
17.	1,2-Dibromoethane	93538	043019	0.10	5.00	0.017	1999.9	200.0	2.2	106-93-4	20 ppm (8H)	orl-rat 108mg/kg
18.	1,2-Dichloroethane	93538	043019	0.10	5.00	0.017	1999.9	200.0	2.1	107-06-2	50 ppm (8H)	orl-rat 670mg/kg
19.	1,2-Dichloropropane	93538	043019	0.10	5.00	0.017	1999.9	200.0	2.2	78-87-5	75 ppm (350mg/m3/8H)	orl-rat 1947mg/kg
20.	1,3-Dichloropropane	93538	043019	0.10	5.00	0.017	1999.8	200.0	2.2	142-28-9	N/A	unr-mus 3600mg/kg
21.	1,1-Dichloropropene	93538	043019	0.10	5.00	0.017	1981.8	198.2	2.8	563-58-6	N/A	N/A
22.	cis-1,3-Dichloropropene	93538	043019	0.10	5.00	0.017	1999.8	200.0	2.2	10061-01-5	N/A	N/A
23.	trans-1,3-Dichloropropene	93538	043019	0.10	5.00	0.017	1999.8	200.0	2.2	10061-02-6	N/A	N/A
24.	Hexachloro-1,3-butadiene	93538	043019	0.10	5.00	0.017	1999.8	200.0	2.2	87-68-3	0.02 ppm (0.24mg/m3/8H)	orl-rat 82mg/kg
25.	1,1,1,2-Tetrachloroethane	93538	043019	0.10	5.00	0.017	1999.8	200.0	2.2	630-20-6	N/A	orl-rat 670mg/kg
26.	1,1,2,2-Tetrachloroethane	93538	043019	0.10	5.00	0.017	1999.9	200.0	2.2	79-34-5	5 ppm (35mg/m3/9H)(skin)	orl-rat 800mg/kg
27.	1,1,2-Trichloroethane	93538	043019	0.10	5.00	0.017	1999.8	200.0	2.2	79-00-5	10 ppm (45mg/m3/8H)(skin)	orl-rat 836mg/kg
28.	Trichloroethene	93538	043019	0.10	5.00	0.017	1999.8	200.0	2.2	79-01-6	50 ppm (270mg/m3/8H)	orl-mus 2402mg/kg
29.	1,2,3-Trichloropropane	93538	043019	0.10	5.00	0.017	1999.8	200.0	2.2	96-18-4	10 ppm (60mg/m3/8H)	orl-rat 149.6mg/kg
30.	Benzene	93538	043019	0.10	5.00	0.017	2000.0	200.0	2.4	71-43-2	1 ppm	orl-rat 4894mg/kg
31.	Bromobenzene	93538	043019	0.10	5.00	0.017	2000.2	200.0	2.4	108-86-1	N/A	orl-rat 2699mg/kg
32.	n-Butyl benzene	93538	043019	0.10	5.00	0.017	2000.9	200.1	2.4	104-51-8	N/A	N/A
33.	Ethyl benzene	93538	043019	0.10	5.00	0.017	2000.0	200.0	2.4	100-41-4	100 ppm (435mg/m3/8H)	orl-rat >2000mg/kg
34.	p-Isopropyl toluene	93538	043019	0.10	5.00	0.017	2000.3	200.0	2.4	99-87-6	N/A	orl-rat 4750mg/kg
35.	Naphthalene	93538	043019	0.10	5.00	0.017	2000.1	200.0	2.4	91-20-3	10 ppm (50mg/m3/8H)	orl-rat 490mg/kg
36.	Toluene	93538	043019	0.10	5.00	0.017	2000.1	200.0	2.4	108-88-3	200 ppm	orl-rat 5000mg/kg
37.	1,2,3-Trichlorobenzene	93538	043019	0.10	5.00	0.017	2001.1	200.1	2.4	87-61-6	N/A	ipr-mus 1390mg/kg
38.	1,2,4-Trichlorobenzene	93538	043019	0.10	5.00	0.017	2000.7	200.1	2.4	120-82-1	5 ppm (CL) (40mg/m3)	orl-rat 756mg/kg
39.	1,2,4-Trimethylbenzene	93538	043019	0.10	5.00	0.017	2000.6	200.1	2.4	95-63-6	N/A	orl-rat 5g/kg
40.	1,3,5-Trimethylbenzene	93538	043019	0.10	5.00	0.017	2000.4	200.1	2.4	108-67-8	N/A	N/A
41.	Styrene	93538	043019	0.10	5.00	0.017	2000.0	200.0	2.4	100-42-5	100 ppm	orl-rat 5000mg/kg
42.	tert-Butyl benzene	93538	043019	0.10	5.00	0.017	2000.2	200.0	2.4	98-06-6	N/A	N/A
43.	sec-Butyl benzene	93538	043019	0.10	5.00	0.017	2000.4	200.0	2.4	135-98-8	N/A	orl-rat 2240mg/kg
44.	Chlorobenzene	93538	043019	0.10	5.00	0.017	2000.6	200.1	2.4	108-90-7	75 ppm (350mg/m3/8H)	orl-rat 2290mg/kg
45.	2-Chlorotoluene	93538	043019	0.10	5.00	0.017	2000.1	200.0	2.4	95-49-8	50 ppm (250mg/m3/8H)	orl-rat 3900mg/kg
46.	4-Chlorotoluene	93538	043019	0.10	5.00	0.017	2000.3	200.0	2.4	106-43-4	N/A	orl-rat 2100mg/kg
47.	1,2-Dichlorobenzene	93538	043019	0.10	5.00	0.017	2000.6	200.1	2.4	95-50-1	50 ppm (300mg/m3) (CL)	orl-rat 500mg/kg
48.	1,3-Dichlorobenzene	93538	043019	0.10	5.00	0.017	2000.5	200.1	2.4	541-73-1	N/A	ipr-mus 1062mg/kg
49.	1,4-Dichlorobenzene	93538	043019	0.10	5.00	0.017	2000.3	200.0	2.4	106-46-7	75 ppm (450mg/m3/8H)	orl-rat 500mg/kg
50.	Isopropylbenzene	93538	043019	0.10	5.00	0.017	2000.6	200.1	2.4	98-82-8	50 ppm (245mg/m3/8H)	orl-rat 1400mg/kg
51.	n-Propylbenzene	93538	043019	0.10	5.00	0.017	2000.4	200.0	2.4	103-65-1	N/A	orl-rat 6040mg/kg
52.	o-Xylene	93538	043019	0.10	5.00	0.017	2000.0	200.0	2.4	95-47-6	100 ppm (435mg/m3/8H)	ipr-mus 1364mg/kg
53.	m-Xylene	93538	043019	0.10	5.00	0.017	1000.0	**100.0**	1.1	108-38-3	100 ppm (435mg/m3/8H)	orl-rat 5g/kg
54.	p-Xylene	93538	043019	0.10	5.00	0.017	999.9	**100.0**	1.1	106-42-3	100 ppm (435mg/m3/8H)	orl-rat 5g/kg
55.	Carbon disulphide	97269	020720	0.01	0.50	0.004	20004.9	200.1	6.3	75-15-0	4 ppm (12mg/m3) (skin)	orl-rat 1200mg/kg
56.	1,4-Dioxane	97269	020720	0.01	0.50	0.004	20006.9	200.1	6.3	123-91-1	25 ppm (90mg/m3/8H)(skin)	orl-mus 5700mg/kg
57.	Hexachloroethane	97269	020720	0.01	0.50	0.004	20005.9	200.1	6.3	67-72-1	1 ppm (10mg/m3/8H)(skin)	orl-gpg 4970mg/kg
58.	Methyl tert-butyl ether (MTBE)	97269	020720	0.01	0.50	0.004	20005.9	200.1	6.3	1634-04-4	N/A	orl-rat 4g/kg
59.	2-Methylnaphthalene	97269	020720	0.01	0.50	0.004	20003.3	200.0	6.3	91-57-6	N/A	orl-rat 1630mg/kg
60.	1,1,2-Trichlorotrifluoroethane	97269	020720	0.01	0.50	0.004	20007.9	200.1	6.3	76-13-1	1000 ppm (7600mg/m3/8H)	orl-rat 43g/kg
61.	n-Pentane	97235	120114	0.10	5.00	0.017	2002.6	200.3	1.7	109-66-0	600 ppm(1800mg/m3/8H)	ivn-mus 446mg/kg
62.	n-Hexane	97235	120114	0.10	5.00	0.017	2002.6	200.3	1.7	110-54-3	50 ppm(180mg/m3/8H)	orl-rat 28710mg/kg
63.	n-Heptane	97235	120114	0.10	5.00	0.017	2003.1	200.3	1.7	142-82-5	400 ppm(1600mg/m3/8H)	ivn-mus 222mg/kg
64.	n-Octane	97235	120114	0.10	5.00	0.017	2001.6	200.2	1.7	111-65-9	300 ppm(1450mg/m3/8H)	N/A
65.	n-Nonane	97235	120114	0.10	5.00	0.017	2000.7	200.1	1.7	111-84-2	200 ppm (1050mg/m3/8H)	ivn-mus 218mg/kg
66.	n-Decane	97235	120114	0.10	5.00	0.017	2001.8	200.2	1.7	124-18-5	N/A	N/A
67.	n-Undecane	97235	120114	0.10	5.00	0.017	2003.6	200.4	1.7	1120-21-4	N/A	ivn-mus 517mg/kg
68.	n-Dodecane	97235	120114	0.10	5.00	0.017	2000.6	200.1	1.7	112-40-3	N/A	N/A
69.	n-Tridecane	97235	120114	0.10	5.00	0.017	2000.2	200.0	1.7	629-50-5	N/A	ivn-mus 1161mg/kg
70.	n-Tetradecane	97235	120114	0.10	5.00	0.017	2001.5	200.2	1.7	629-59-4	N/A	N/A
71.	n-Pentadecane	97235	120114	0.10	5.00	0.017	2001.3	200.1	1.7	629-62-9	N/A	ivn-mus 3494mg/kg
72.	Bromomethane	30058	123019	0.10	5.00	0.017	2004.8	200.5	1.7	74-83-9	5 ppm (20mg/m3/8H) (skin)	orl-rat 214mg/kg
73.	Chloroethane	30058	123019	0.10	5.00	0.017	2002.5	200.3	1.7	75-00-3	1000 ppm (2600mg/m3/8H)	N/A
74.	Chloromethane	30058	123019	0.10	5.00	0.017	2003.0	200.3	1.7	74-87-3	100 ppm	orl-rat 1800mg/kg
75.	Dichlorodifluoromethane	30058	123019	0.10	5.00	0.017	2034.9	203.5	1.7	75-71-8	1000 ppm (4950mg/m3/8H)	N/A
76.	Trichlorofluoromethane	30058	123019	0.10	5.00	0.017	2009.6	201.0	1.7	75-69-4	1000 ppm (5600mg/m3/8H)	ipr-mus 1743mg/kg
77.	Vinyl chloride	30058	123019	0.10	5.00	0.017	2001.2	200.1	1.7	75-01-4	N/A	N/A
78.	4-Methyl-2-pentanone (MIBK)	82442	112816	0.01	0.50	0.004	20003.5	200.0	6.3	108-10-1	100 ppm (410mg/m3/8H)	orl-rat 2080mg/kg
79.	p-Bromofluorobenzene	20002	020419	0.10	5.00	0.017	2000.7	200.1	1.7	460-00-4	N/A	orl-rat 2700mg/kg
80.	1,2-Dichloroethane-d4	20002	020419	0.10	5.00	0.017	2000.6	200.1	1.7	17060-07-0	N/A	orl-mus 625mg/kg
81.	Toluene-d8	20002	020419	0.10	5.00	0.017	2000.4	200.1	1.7	2037-26-5	200 ppm	orl-rat 5000mg/kg
82.	Chlorobenzene-d5	22013	021720	0.10	5.00	0.017	2001.6	**200.2**	1.7	3114-55-4	N/A	orl-rat 1110mg/kg
83.	1,4-Dichlorobenzene-d4	22013	021720	0.10	5.00	0.017	2002.0	**200.2**	1.7	3855-82-1	N/A	orl-rat 500mg/kg
84.	Fluorobenzene	22013	021720	0.10	5.00	0.017	2002.1	**200.2**	1.7	462-06-6	N/A	orl-rat 4399mg/kg

- The certified value is the concentration calculated from gravimetric and volumetric measurements unless otherwise stated.
- Standards are prepared gravimetrically using balances that are calibrated with weights traceable to NIST (see above).
- Standards are certifed (+/-) 0.5% of the stated value, unless otherwise stated.
- All Standards, after opening ampule, should be stored with caps tight and under appropriate laboratory conditions.
- Uncertainty Reference: Taylor, B.N. and Kuyat, C.E., "Guidelines for Evaluating and Expressing the Uncertainty of NIST Measurement Result," NIST Technical Note 1297, U.S. Government Printing Office, Washington, DC, (1994).

2203A Commerce Road, Suite 1
Forest Hill, MD 21050 USA
1.410.838.8780

CERTIFICATE OF ANALYSIS

**University of Notre Dame**	**Site Name:** So-Cal Military Toxic Site	**Beacon Proposal:** 201201H01
Department of Biological Sciences	**Site Location:** Notspa, CA	**Lab Work Order:** 0005542
South Bend, IN 46556	**Project Manager:** Kristin Shrader-Frechette	**Reported:** 01/25/2021

Vendor: Absolute Standards, Inc. **Lab Standard No.:** 2000095

Lot No.: 021820

Absolute Standards, Inc.
800-368-1131
www.absolutestandards.com

Certified Reference Material CRM

ANAB ISO 17034 Accredited
AR-1539 Certificate Number
https://Absolutestandards.com

CERTIFIED WEIGHT REPORT

**Part Number:**	97258		
**Lot Number:**	021820		
**Description:**	8260 VOC Primary Calibration Level 6		
	84 components		
**Expiration Date:**	021823	**Solvent(s):** Methanol	**Lot#** DV182-USQ12
**Recommended Storage:**	Freezer (0 °C)		
**Nominal Concentration (µg/mL):**	400		
**NIST Test ID#:**	6UTB	5E-05	Balance Uncertainty
Volume(s) shown below were combined and diluted to (mL):	50.0	0.007	Flask Uncertainty

Gabriel Helland (signature)			021820
Formulated By:	Gabriel Helland		DATE
Pedro L. Rentas (signature)			021820
Reviewed By:	Pedro L. Rentas		DATE

SDS Information (Solvent Safety Info. On Attached pg.)

	Compound	Part Number	Lot Number	Dil. Factor	Initial Vol. (mL)	Uncertainty Pipette (mL)	Initial Conc.(ug/mL)	Final Conc.(ug/mL)	Expanded Uncertainty (+/-) (µg/mL)	CAS#	OSHA PEL (TWA)	LD50
1.	Bromodichloromethane	93538	043019	0.20	10.00	0.042	1999.8	400.0	4.6	75-27-4	N/A	orl-rat 916mg/kg
2.	Dibromochloromethane	93538	043019	0.20	10.00	0.042	1999.8	400.0	4.6	124-48-1	N/A	orl-rat 848mg/kg
3.	cis-1,2-Dichloroethene	93538	043019	0.20	10.00	0.042	1999.9	400.0	4.6	156-59-2	N/A	N/A
4.	trans-1,2-Dichloroethene	93538	043019	0.20	10.00	0.042	1999.8	400.0	4.6	156-60-5	N/A	orl-rat 1235mg/kg
5.	Methylene chloride	93538	043019	0.20	10.00	0.042	1999.9	400.0	4.6	75-09-2	500 ppm	orl-rat 820mg/kg
6.	1,1-Dichloroethene	93538	043019	0.20	10.00	0.042	2000.3	400.1	5.0	75-35-4	1 ppm (4mg/m3/8H)	orl-rat 200mg/kg
7.	Bromochloromethane	93538	043019	0.20	10.00	0.042	2000.0	400.0	4.6	74-97-5	200 ppm (1050mg/m3/8H)	orl-rat 5000mg/kg
8.	Bromoform	93538	043019	0.20	10.00	0.042	2000.0	400.0	4.6	75-25-2	0.5 ppm (5mg/m3) (skin)	orl-rat 933mg/kg
9.	Carbon tetrachloride	93538	043019	0.20	10.00	0.042	2000.0	400.0	4.6	56-23-5	2 ppm (12.6mg/m3/8H)	orl-rat 2350mg/kg
10.	Chloroform	93538	043019	0.20	10.00	0.042	2000.0	400.0	4.6	67-66-3	50 ppm (240mg/m3) (CL)	orl-rat 908mg/kg
11.	Dibromomethane	93538	043019	0.20	10.00	0.042	2000.0	400.0	4.6	74-95-3	N/A	orl-rat 108mg/kg
12.	1,1-Dichloroethane	93538	043019	0.20	10.00	0.042	1999.9	400.0	4.6	75-34-3	100 ppm	orl-rat 725mg/kg
13.	2,2-Dichloropropane	93538	043019	0.20	10.00	0.042	2000.0	400.0	4.6	594-20-7	N/A	N/A
14.	Tetrachloroethene	93538	043019	0.20	10.00	0.042	2000.0	400.0	4.6	127-18-4	25 ppm (170mg/m3/8H)(final)	orl-rat 2629mg/kg
15.	1,1,1-Trichloroethane	93538	043019	0.20	10.00	0.042	1999.9	400.0	4.6	71-55-6	350 ppm (1900mg/m3/8H)	orl-rat 10300mg/kg
16.	1,2-Dibromo-3-chloropropane	93538	043019	0.20	10.00	0.042	1999.8	400.0	4.6	96-12-8	0.001 ppm	orl-rat 170mg/kg
17.	1,2-Dibromoethane	93538	043019	0.20	10.00	0.042	1999.9	400.0	4.6	106-93-4	20 ppm (8H)	orl-rat 108mg/kg
18.	1,2-Dichloroethane	93538	043019	0.20	10.00	0.042	1999.9	400.0	4.6	107-06-2	50 ppm (8H)	orl-rat 670mg/kg
19.	1,2-Dichloropropane	93538	043019	0.20	10.00	0.042	1999.9	400.0	4.6	78-87-5	75 ppm (350mg/m3/8H)	orl-rat 1947mg/kg
20.	1,3-Dichloropropane	93538	043019	0.20	10.00	0.042	1999.8	400.0	4.6	142-28-9	N/A	unr-mus 3600mg/kg
21.	1,1-Dichloropropene	93538	043019	0.20	10.00	0.042	1981.8	396.4	5.9	563-58-6	N/A	N/A
22.	cis-1,3-Dichloropropene	93538	043019	0.20	10.00	0.042	1999.8	400.0	4.6	10061-01-5	N/A	N/A
23.	trans-1,3-Dichloropropene	93538	043019	0.20	10.00	0.042	1999.8	400.0	4.6	10061-02-6	N/A	N/A
24.	Hexachloro-1,3-butadiene	93538	043019	0.20	10.00	0.042	1999.8	400.0	4.6	87-68-3	0.02 ppm (0.24mg/m3/8H)	orl-rat 82mg/kg
25.	1,1,1,2-Tetrachloroethane	93538	043019	0.20	10.00	0.042	1999.8	400.0	4.6	630-20-6	N/A	orl-rat 670mg/kg
26.	1,1,2,2-Tetrachloroethane	93538	043019	0.20	10.00	0.042	1999.9	400.0	4.6	79-34-5	5 ppm (35mg/m3/9H)(skin)	orl-rat 800mg/kg
27.	1,1,2-Trichloroethane	93538	043019	0.20	10.00	0.042	1999.8	400.0	4.6	79-00-5	10 ppm (45mg/m3/8H)(skin)	orl-rat 836mg/kg
28.	Trichloroethene	93538	043019	0.20	10.00	0.042	1999.8	400.0	4.6	79-01-6	50 ppm (270mg/m3/8H)	orl-mus 2402mg/kg
29.	1,2,3-Trichloropropane	93538	043019	0.20	10.00	0.042	1999.8	400.0	4.6	96-18-4	10 ppm (60mg/m3/8H)	orl-rat 149.6mg/kg
30.	Benzene	93538	043019	0.20	10.00	0.042	2000.0	400.0	5.0	71-43-2	1 ppm	orl-rat 4894mg/kg
31.	Bromobenzene	93538	043019	0.20	10.00	0.042	2000.2	400.1	5.0	108-86-1	N/A	orl-rat 2699mg/kg
32.	n-Butyl benzene	93538	043019	0.20	10.00	0.042	2000.9	400.2	5.0	104-51-8	N/A	N/A
33.	Ethyl benzene	93538	043019	0.20	10.00	0.042	2000.0	400.0	5.0	100-41-4	100 ppm (435mg/m3/8H)	orl-rat >2000mg/kg
34.	p-Isopropyl toluene	93538	043019	0.20	10.00	0.042	2000.3	400.1	5.0	99-87-6	N/A	orl-rat 4750mg/kg
35.	Naphthalene	93538	043019	0.20	10.00	0.042	2000.1	400.0	5.0	91-20-3	10 ppm (50mg/m3/8H)	orl-rat 490mg/kg
36.	Toluene	93538	043019	0.20	10.00	0.042	2000.1	400.0	5.0	108-88-3	200 ppm	orl-rat 5000mg/kg
37.	1,2,3-Trichlorobenzene	93538	043019	0.20	10.00	0.042	2001.1	400.2	5.0	87-61-6	N/A	ipr-mus 1390mg/kg
38.	1,2,4-Trichlorobenzene	93538	043019	0.20	10.00	0.042	2000.7	400.2	5.0	120-82-1	5 ppm (CL) (40mg/m3)	orl-rat 756mg/kg
39.	1,2,4-Trimethylbenzene	93538	043019	0.20	10.00	0.042	2000.6	400.1	5.0	95-63-6	N/A	orl-rat 5g/kg
40.	1,3,5-Trimethylbenzene	93538	043019	0.20	10.00	0.042	2000.4	400.1	5.0	108-67-8	N/A	N/A
41.	Styrene	93538	043019	0.20	10.00	0.042	2000.0	400.0	5.0	100-42-5	100 ppm	orl-rat 5000mg/kg
42.	tert-Butyl benzene	93538	043019	0.20	10.00	0.042	2000.2	400.1	5.0	98-06-6	N/A	N/A
43.	sec-Butyl benzene	93538	043019	0.20	10.00	0.042	2000.4	400.1	5.0	135-98-8	N/A	orl-rat 2240mg/kg
44.	Chlorobenzene	93538	043019	0.20	10.00	0.042	2000.6	400.1	5.0	108-90-7	75 ppm (350mg/m3/8H)	orl-rat 2290mg/kg
45.	2-Chlorotoluene	93538	043019	0.20	10.00	0.042	2000.1	400.0	5.0	95-49-8	50 ppm (250mg/m3/8H)	orl-rat 3900mg/kg
46.	4-Chlorotoluene	93538	043019	0.20	10.00	0.042	2000.3	400.1	5.0	106-43-4	N/A	orl-rat 2100mg/kg
47.	1,2-Dichlorobenzene	93538	043019	0.20	10.00	0.042	2000.6	400.1	5.0	95-50-1	50 ppm (300mg/m3) (CL)	orl-rat 500mg/kg
48.	1,3-Dichlorobenzene	93538	043019	0.20	10.00	0.042	2000.5	400.1	5.0	541-73-1	N/A	ipr-mus 1062mg/kg
49.	1,4-Dichlorobenzene	93538	043019	0.20	10.00	0.042	2000.3	400.1	5.0	106-46-7	75 ppm (450mg/m3/8H)	orl-rat 500mg/kg
50.	Isopropylbenzene	93538	043019	0.20	10.00	0.042	2000.6	400.1	5.0	98-82-8	50 ppm (245mg/m3/8H)	orl-rat 1400mg/kg
51.	n-Propylbenzene	93538	043019	0.20	10.00	0.042	2000.4	400.1	5.0	103-65-1	N/A	orl-rat 6040mg/kg
52.	o-Xylene	93538	043019	0.20	10.00	0.042	2000.0	400.0	5.0	95-47-6	100 ppm (435mg/m3/8H)	ipr-mus 1364mg/kg
53.	m-Xylene	93538	043019	0.20	10.00	0.042	1000.0	**200.0**	2.3	108-38-3	100 ppm (435mg/m3/8H)	orl-rat 5g/kg
54.	p-Xylene	93538	043019	0.20	10.00	0.042	999.9	**200.0**	2.3	106-42-3	100 ppm (435mg/m3/8H)	orl-rat 5g/kg
55.	Carbon disulphide	97269	020720	0.02	1.00	0.004	20004.9	400.1	3.6	75-15-0	4 ppm (12mg/m3) (skin)	orl-rat 1200mg/kg
56.	1,4-Dioxane	97269	020720	0.02	1.00	0.004	20006.9	400.2	3.6	123-91-1	25 ppm (90mg/m3/8H)(skin)	orl-mus 5700mg/kg
57.	Hexachloroethane	97269	020720	0.02	1.00	0.004	20005.9	400.1	3.6	67-72-1	1 ppm (10mg/m3/8H)(skin)	orl-gpg 4970mg/kg
58.	Methyl tert-butyl ether (MTBE)	97269	020720	0.02	1.00	0.004	20005.9	400.1	3.6	1634-04-4	N/A	orl-rat 4g/kg
59.	2-Methylnaphthalene	97269	020720	0.02	1.00	0.004	20003.3	400.1	3.6	91-57-6	N/A	orl-rat 1630mg/kg
60.	1,1,2-Trichlorotrifluoroethane	97269	020720	0.02	1.00	0.004	20007.9	400.2	3.6	76-13-1	1000 ppm (7600mg/m3/8H)	orl-rat 43g/kg
61.	n-Pentane	97235	120114	0.20	10.00	0.042	2002.6	400.5	3.7	109-66-0	600 ppm(1800mg/m3/8H)	ivn-mus 446mg/kg
62.	n-Hexane	97235	120114	0.20	10.00	0.042	2002.6	400.5	3.7	110-54-3	50 ppm(180mg/m3/8H)	orl-rat 28710mg/kg
63.	n-Heptane	97235	120114	0.20	10.00	0.042	2003.1	400.6	3.8	142-82-5	400 ppm(1600mg/m3/8H)	ivn-mus 222mg/kg
64.	n-Octane	97235	120114	0.20	10.00	0.042	2001.6	400.3	3.7	111-65-9	300 ppm(1450mg/m3/8H)	N/A
65.	n-Nonane	97235	120114	0.20	10.00	0.042	2000.7	400.2	3.7	111-84-2	200 ppm (1050mg/m3/8H)	ivn-mus 218mg/kg
66.	n-Decane	97235	120114	0.20	10.00	0.042	2001.8	400.4	3.7	124-18-5	N/A	N/A
67.	n-Undecane	97235	120114	0.20	10.00	0.042	2003.6	400.7	3.8	1120-21-4	N/A	ivn-mus 517mg/kg
68.	n-Dodecane	97235	120114	0.20	10.00	0.042	2000.6	400.1	3.7	112-40-3	N/A	N/A
69.	n-Tridecane	97235	120114	0.20	10.00	0.042	2000.2	400.1	3.7	629-50-5	N/A	ivn-mus 1161mg/kg
70.	n-Tetradecane	97235	120114	0.20	10.00	0.042	2001.5	400.3	3.7	629-59-4	N/A	N/A
71.	n-Pentadecane	97235	120114	0.20	10.00	0.042	2001.3	400.3	3.7	629-62-9	N/A	ivn-mus 3494mg/kg
72.	Bromomethane	30058	123019	0.20	10.00	0.042	2004.8	401.0	3.7	74-83-9	5 ppm (20mg/m3/8H) (skin)	orl-rat 214mg/kg
73.	Chloroethane	30058	123019	0.20	10.00	0.042	2002.5	400.5	3.7	75-00-3	1000 ppm (2600mg/m3/8H)	N/A
74.	Chloromethane	30058	123019	0.20	10.00	0.042	2003.0	400.6	3.7	74-87-3	100 ppm	orl-rat 1800mg/kg
75.	Dichlorodifluoromethane	30058	123019	0.20	10.00	0.042	2034.9	407.0	3.8	75-71-8	1000 ppm (4950mg/m3/8H)	N/A
76.	Trichlorofluoromethane	30058	123019	0.20	10.00	0.042	2009.6	401.9	3.8	75-69-4	1000 ppm (5600mg/m3/8H)	ipr-mus 1743mg/kg
77.	Vinyl chloride	30058	123019	0.20	10.00	0.042	2001.2	400.3	3.7	75-01-4	N/A	N/A
78.	4-Methyl-2-pentanone (MIBK)	82442	103019	0.02	1.00	0.004	20002.0	400.1	3.6	108-10-1	100 ppm (410mg/m3/8H)	orl-rat 2080mg/kg
79.	p-Bromofluorobenzene	20002	020419	0.20	10.00	0.042	2000.7	400.2	3.7	460-00-4	N/A	orl-rat 2700mg/kg
80.	1,2-Dichloroethane-d4	20002	020419	0.20	10.00	0.042	2000.6	400.1	3.7	17060-07-0	N/A	orl-mus 625mg/kg
81.	Toluene-d8	20002	020419	0.20	10.00	0.042	2000.4	400.1	3.7	2037-26-5	200 ppm	orl-rat 5000mg/kg
82.	Chlorobenzene-d5	22013	021720	0.10	5.00	0.017	2001.6	**200.2**	1.6	3114-55-4	N/A	orl-rat 1110mg/kg
83.	1,4-Dichlorobenzene-d4	22013	021720	0.10	5.00	0.017	2002.0	**200.2**	1.6	3855-82-1	N/A	orl-rat 500mg/kg
84.	Fluorobenzene	22013	021720	0.10	5.00	0.017	2002.1	**200.2**	1.6	462-06-6	N/A	orl-rat 4399mg/kg

- The certified value is the concentration calculated from gravimetric and volumetric measurements unless otherwise stated.
- Standards are prepared gravimetrically using balances that are calibrated with weights traceable to NIST (see above).
- Standards are certifed (+/-) 0.5% of the stated value, unless otherwise stated.
- All Standards, after opening ampule, should be stored with caps tight and under appropriate laboratory conditions.
- Uncertainty Reference: Taylor, B.N. and Kuyat, C.E., "Guidelines for Evaluating and Expressing the Uncertainty of NIST Measurement Result," NIST Technical Note 1297, U.S. Government Printing Office, Washington, DC, (1994).

BEACON ENVIRONMENTAL
2203A Commerce Road, Suite 1
Forest Hill, MD 21050 USA
1.410.838.8780

CERTIFICATE OF ANALYSIS

**University of Notre Dame**	**Site Name:** So-Cal Military Toxic Site	**Beacon Proposal:** 201201H01
Department of Biological Sciences	**Site Location:** Notspa, CA	**Lab Work Order:** 0005542
South Bend, IN 46556	**Project Manager:** Kristin Shrader-Frechette	**Reported:** 01/25/2021

Vendor: Absolute Standards, Inc. **Lab Standard No.:** 2000348

Lot No.: 021820

Absolute Standards, Inc.
800-368-1131
www.absolutestandards.com

Certified Reference Material CRM

ANAB ISO 17034 Accredited
AR-1539 Certificate Number
https://Absolutestandards.com

CERTIFIED WEIGHT REPORT

Part Number: 97256
Lot Number: 021820
Description: 8260 VOC Primary Calibration Level 4
84 components
Expiration Date: 021823 — **Solvent(s):** Methanol — **Lot#** DV162-USQ12
Recommended Storage: Freezer (0 °C)
Nominal Concentration (µg/mL): 100
NIST Test ID#: 6UTB — 5E-05 Balance Uncertainty
Volume(s) shown below were combined and diluted to (mL): 100.0 — 0.012 Flask Uncertainty

Gabriel Helland (signature)		021820
Formulated By:	Gabriel Helland	DATE
Pedro L. Rentas (signature)		021820
Reviewed By:	Pedro L. Rentas	DATE

	Compound	Part Number	Lot Number	Dil. Factor	Initial Vol. (mL)	Uncertainty Pipette (mL)	Initial Conc. (ug/mL)	Final Conc. (ug/mL)	Expanded Uncertainty (+/-) (µg/mL)	SDS Information (Solvent Safety Info. On Attached pg.) CAS#	OSHA PEL (TWA)	LD50
1.	Bromodichloromethane	93538	043019	0.05	5.00	0.017	1999.8	100.0	1.1	75-27-4	N/A	orl-rat 916mg/kg
2.	Dibromochloromethane	93538	043019	0.05	5.00	0.017	1999.8	100.0	1.1	124-48-1	N/A	orl-rat 848mg/kg
3.	cis-1,2-Dichloroethene	93538	043019	0.05	5.00	0.017	1999.9	100.0	1.1	156-59-2	N/A	N/A
4.	trans-1,2-Dichloroethene	93538	043019	0.05	5.00	0.017	1999.8	100.0	1.1	156-60-5	N/A	orl-rat 1235mg/kg
5.	Methylene chloride	93538	043019	0.05	5.00	0.017	1999.9	100.0	1.1	75-09-2	500 ppm	orl-rat 820mg/kg
6.	1,1-Dichloroethene	93538	043019	0.05	5.00	0.017	2000.3	100.0	1.2	75-35-4	1 ppm (4mg/m3/8H)	orl-rat 200mg/kg
7.	Bromochloromethane	93538	043019	0.05	5.00	0.017	2000.0	100.0	1.1	74-97-5	200 ppm (1050mg/m3/8H)	orl-rat 5000mg/kg
8.	Bromoform	93538	043019	0.05	5.00	0.017	2000.0	100.0	1.1	75-25-2	0.5 ppm (5mg/m3) (skin)	orl-rat 933mg/kg
9.	Carbon tetrachloride	93538	043019	0.05	5.00	0.017	2000.0	100.0	1.1	56-23-5	2 ppm (12.6mg/m3/8H)	orl-rat 2350mg/kg
10.	Chloroform	93538	043019	0.05	5.00	0.017	2000.0	100.0	1.1	67-66-3	50 ppm (240mg/m3) (CL)	orl-rat 908mg/kg
11.	Dibromomethane	93538	043019	0.05	5.00	0.017	2000.0	100.0	1.1	74-95-3	N/A	orl-rat 108mg/kg
12.	1,1-Dichloroethane	93538	043019	0.05	5.00	0.017	1999.9	100.0	1.1	75-34-3	100 ppm	orl-rat 725mg/kg
13.	2,2-Dichloropropane	93538	043019	0.05	5.00	0.017	2000.0	100.0	1.1	594-20-7	N/A	N/A
14.	Tetrachloroethene	93538	043019	0.05	5.00	0.017	2000.0	100.0	1.1	127-18-4	25 ppm (170mg/m3/8H)(final)	orl-rat 2629mg/kg
15.	1,1,1-Trichloroethane	93538	043019	0.05	5.00	0.017	1999.9	100.0	1.1	71-55-6	350 ppm (1900mg/m3/8H)	orl-rat 10300mg/kg
16.	1,2-Dibromo-3-chloropropane	93538	043019	0.05	5.00	0.017	1999.8	100.0	1.1	96-12-8	0.001 ppm	orl-rat 170mg/kg
17.	1,2-Dibromoethane	93538	043019	0.05	5.00	0.017	1999.9	100.0	1.1	106-93-4	20 ppm (8H)	orl-rat 108mg/kg
18.	1,2-Dichloroethane	93538	043019	0.05	5.00	0.017	1999.9	100.0	1.1	107-06-2	50 ppm (8H)	orl-rat 670mg/kg
19.	1,2-Dichloropropane	93538	043019	0.05	5.00	0.017	1999.9	100.0	1.1	78-87-5	75 ppm (350mg/m3/8H)	orl-rat 1947mg/kg
20.	1,3-Dichloropropane	93538	043019	0.05	5.00	0.017	1999.8	100.0	1.1	142-28-9	N/A	unr-mus 3600mg/kg
21.	1,1-Dichloropropene	93538	043019	0.05	5.00	0.017	1981.8	99.1	1.5	563-58-6	N/A	N/A
22.	cis-1,3-Dichloropropene	93538	043019	0.05	5.00	0.017	1999.8	100.0	1.1	10061-01-5	N/A	N/A
23.	trans-1,3-Dichloropropene	93538	043019	0.05	5.00	0.017	1999.8	100.0	1.1	10061-02-6	N/A	N/A
24.	Hexachloro-1,3-butadiene	93538	043019	0.05	5.00	0.017	1999.8	100.0	1.1	87-68-3	0.02 ppm (0.24mg/m3/8H)	orl-rat 82mg/kg
25.	1,1,1,2-Tetrachloroethane	93538	043019	0.05	5.00	0.017	1999.8	100.0	1.1	630-20-6	N/A	orl-rat 670mg/kg
26.	1,1,2,2-Tetrachloroethane	93538	043019	0.05	5.00	0.017	1999.9	100.0	1.1	79-34-5	5 ppm (35mg/m3/8H)(skin)	orl-rat 800mg/kg
27.	1,1,2-Trichloroethane	93538	043019	0.05	5.00	0.017	1999.8	100.0	1.1	79-00-5	10 ppm (45mg/m3/8H)(skin)	orl-rat 836mg/kg
28.	Trichloroethene	93538	043019	0.05	5.00	0.017	1999.8	100.0	1.1	79-01-6	50 ppm (270mg/m3/8H)	orl-mus 2402mg/kg
29.	1,2,3-Trichloropropane	93538	043019	0.05	5.00	0.017	1999.8	100.0	1.1	96-18-4	10 ppm (60mg/m3/8H)	orl-rat 149.6mg/kg
30.	Benzene	93538	043019	0.05	5.00	0.017	2000.0	100.0	1.2	71-43-2	1 ppm	orl-rat 4894mg/kg
31.	Bromobenzene	93538	043019	0.05	5.00	0.017	2000.2	100.0	1.2	108-86-1	N/A	orl-rat 2699mg/kg
32.	n-Butyl benzene	93538	043019	0.05	5.00	0.017	2000.9	100.0	1.2	104-51-8	N/A	N/A
33.	Ethyl benzene	93538	043019	0.05	5.00	0.017	2000.0	100.0	1.2	100-41-4	100 ppm (435mg/m3/8H)	orl-rat >2000mg/kg
34.	p-Isopropyl toluene	93538	043019	0.05	5.00	0.017	2000.3	100.0	1.2	99-87-6	N/A	orl-rat 4750mg/kg
35.	Naphthalene	93538	043019	0.05	5.00	0.017	2000.1	100.0	1.2	91-20-3	10 ppm (50mg/m3/8H)	orl-rat 490mg/kg
36.	Toluene	93538	043019	0.05	5.00	0.017	2000.1	100.0	1.2	108-88-3	200 ppm	orl-rat 5000mg/kg
37.	1,2,3-Trichlorobenzene	93538	043019	0.05	5.00	0.017	2001.1	100.0	1.2	87-61-6	N/A	ipr-mus 1390mg/kg
38.	1,2,4-Trichlorobenzene	93538	043019	0.05	5.00	0.017	2000.7	100.0	1.2	120-82-1	5 ppm (CL) (40mg/m3)	orl-rat 756mg/kg
39.	1,2,4-Trimethylbenzene	93538	043019	0.05	5.00	0.017	2000.6	100.0	1.2	95-63-6	N/A	orl-rat 5g/kg
40.	1,3,5-Trimethylbenzene	93538	043019	0.05	5.00	0.017	2000.4	100.0	1.2	108-67-8	N/A	N/A
41.	Styrene	93538	043019	0.05	5.00	0.017	2000.0	100.0	1.2	100-42-5	100 ppm	orl-rat 5000mg/kg
42.	tert-Butyl benzene	93538	043019	0.05	5.00	0.017	2000.2	100.0	1.2	98-06-6	N/A	N/A
43.	sec-Butyl benzene	93538	043019	0.05	5.00	0.017	2000.4	100.0	1.2	135-98-8	N/A	orl-rat 2240mg/kg
44.	Chlorobenzene	93538	043019	0.05	5.00	0.017	2000.6	100.0	1.2	108-90-7	75 ppm (350mg/m3/8H)	orl-rat 2290mg/kg
45.	2-Chlorotoluene	93538	043019	0.05	5.00	0.017	2000.1	100.0	1.2	95-49-8	50 ppm (250mg/m3/8H)	orl-rat 3900mg/kg
46.	4-Chlorotoluene	93538	043019	0.05	5.00	0.017	2000.3	100.0	1.2	106-43-4	N/A	orl-rat 2100mg/kg
47.	1,2-Dichlorobenzene	93538	043019	0.05	5.00	0.017	2000.6	100.0	1.2	95-50-1	50 ppm (300mg/m3) (CL)	orl-rat 500mg/kg
48.	1,3-Dichlorobenzene	93538	043019	0.05	5.00	0.017	2000.5	100.0	1.2	541-73-1	N/A	ipr-mus 1062mg/kg
49.	1,4-Dichlorobenzene	93538	043019	0.05	5.00	0.017	2000.3	100.0	1.2	106-46-7	75 ppm (450mg/m3/8H)	orl-rat 500mg/kg
50.	Isopropylbenzene	93538	043019	0.05	5.00	0.017	2000.6	100.0	1.2	98-82-8	50 ppm (245mg/m3/8H)	orl-rat 1400mg/kg
51.	n-Propylbenzene	93538	043019	0.05	5.00	0.017	2000.4	100.0	1.2	103-65-1	N/A	orl-rat 6040mg/kg
52.	o-Xylene	93538	043019	0.05	5.00	0.017	2000.0	100.0	1.2	95-47-6	100 ppm (435mg/m3/8H)	ipr-mus 1364mg/kg
53.	m-Xylene	93538	043019	0.05	5.00	0.017	1000.0	**50.0**	0.6	108-38-3	100 ppm (435mg/m3/8H)	orl-rat 5g/kg
54.	p-Xylene	93538	043019	0.05	5.00	0.017	999.9	**50.0**	0.6	106-42-3	100 ppm (435mg/m3/8H)	orl-rat 5g/kg
55.	Carbon disulphide	97269	020720	0.005	0.50	0.004	20004.9	100.0	5.0	75-15-0	4 ppm (12mg/m3) (skin)	orl-rat 1200mg/kg
56.	1,4-Dioxane	97269	020720	0.005	0.50	0.004	20006.9	100.0	5.0	123-91-1	25 ppm (90mg/m3/8H)(skin)	orl-mus 5700mg/kg
57.	Hexachloroethane	97269	020720	0.005	0.50	0.004	20005.9	100.0	5.0	67-72-1	1 ppm (10mg/m3/8H)(skin)	orl-gpg 4970mg/kg
58.	Methyl tert-butyl ether (MTBE)	97269	020720	0.005	0.50	0.004	20005.9	100.0	5.0	1634-04-4	N/A	orl-rat 4g/kg
59.	2-Methylnaphthalene	97269	020720	0.005	0.50	0.004	20003.3	100.0	5.0	91-57-6	N/A	orl-rat 1630mg/kg
60.	1,1,2-Trichlorotrifluoroethane	97269	020720	0.005	0.50	0.004	20007.9	100.0	5.0	76-13-1	1000 ppm (7600mg/m3/8H)	orl-rat 43g/kg
61.	n-Pentane	97235	120114	0.05	5.00	0.017	2002.6	100.1	0.9	109-66-0	600 ppm(1800mg/m3/8H)	ivn-mus 446mg/kg
62.	n-Hexane	97235	120114	0.05	5.00	0.017	2002.6	100.1	0.9	110-54-3	50 ppm(180mg/m3/8H)	orl-rat 28710mg/kg
63.	n-Heptane	97235	120114	0.05	5.00	0.017	2003.1	100.2	0.9	142-82-5	400 ppm(1600mg/m3/8H)	ivn-mus 222mg/kg
64.	n-Octane	97235	120114	0.05	5.00	0.017	2001.6	100.1	0.9	111-65-9	300 ppm(1450mg/m3/8H)	N/A
65.	n-Nonane	97235	120114	0.05	5.00	0.017	2000.7	100.0	0.9	111-84-2	200 ppm (1050mg/m3/8H)	ivn-mus 218mg/kg
66.	n-Decane	97235	120114	0.05	5.00	0.017	2001.8	100.1	0.9	124-18-5	N/A	N/A
67.	n-Undecane	97235	120114	0.05	5.00	0.017	2003.6	100.2	0.9	1120-21-4	N/A	ivn-mus 517mg/kg
68.	n-Dodecane	97235	120114	0.05	5.00	0.017	2000.6	100.0	0.9	112-40-3	N/A	N/A
69.	n-Tridecane	97235	120114	0.05	5.00	0.017	2000.2	100.0	0.9	629-50-5	N/A	ivn-mus 1161mg/kg
70.	n-Tetradecane	97235	120114	0.05	5.00	0.017	2001.5	100.1	0.9	629-59-4	N/A	N/A
71.	n-Pentadecane	97235	120114	0.05	5.00	0.017	2001.3	100.1	0.9	629-62-9	N/A	ivn-mus 3494mg/kg
72.	Bromomethane	30058	123019	0.05	5.00	0.017	2004.8	100.2	0.9	74-83-9	5 ppm (20mg/m3/8H) (skin)	orl-rat 214mg/kg
73.	Chloroethane	30058	123019	0.05	5.00	0.017	2002.5	100.1	0.9	75-00-3	1000 ppm (2600mg/m3/8H)	N/A
74.	Chloromethane	30058	123019	0.05	5.00	0.017	2003.0	100.1	0.9	74-87-3	100 ppm	orl-rat 1800mg/kg
75.	Dichlorodifluoromethane	30058	123019	0.05	5.00	0.017	2034.9	101.7	0.9	75-71-8	1000 ppm (4950mg/m3/8H)	N/A
76.	Trichlorofluoromethane	30058	123019	0.05	5.00	0.017	2009.6	100.5	0.9	75-69-4	1000 ppm (5600mg/m3/8H)	ipr-mus 1743mg/kg
77.	Vinyl chloride	30058	123019	0.05	5.00	0.017	2001.2	100.1	0.9	75-01-4	N/A	N/A
78.	4-Methyl-2-pentanone (MIBK)	82442	112816	0.00	0.50	0.004	20003.5	100.0	5.0	108-10-1	100 ppm (410mg/m3/8H)	orl-rat 2080mg/kg
79.	p-Bromofluorobenzene	20002	020419	0.05	5.00	0.017	2000.7	100.0	0.9	460-00-4	N/A	orl-rat 2700mg/kg
80.	1,2-Dichloroethane-d4	20002	020419	0.05	5.00	0.017	2000.6	100.0	0.9	17060-07-0	N/A	orl-mus 625mg/kg
81.	Toluene-d8	20002	020419	0.05	5.00	0.017	2000.4	100.0	0.9	2037-26-5	200 ppm	orl-rat 5000mg/kg
82.	Chlorobenzene-d5	22013	021720	0.10	10.00	0.042	2001.6	**200.1**	1.9	3114-55-4	N/A	orl-rat 1110mg/kg
83.	1,4-Dichlorobenzene-d4	22013	021720	0.10	10.00	0.042	2002.0	**200.2**	1.9	3855-82-1	N/A	orl-rat 500mg/kg
84.	Fluorobenzene	22013	021720	0.10	10.00	0.042	2002.1	**200.2**	1.9	462-06-6	N/A	orl-rat 4399mg/kg

- The certified value is the concentration calculated from gravimetric and volumetric measurements unless otherwise stated.
- Standards are prepared gravimetrically using balances that are calibrated with weights traceable to NIST (see above).
- Standards are certifed (+/-) 0.5% of the stated value, unless otherwise stated.
- All Standards, after opening ampule, should be stored with caps tight and under appropriate laboratory conditions.
- Uncertainty Reference: Taylor, B.N. and Kuyat, C.E., "Guidelines for Evaluating and Expressing the Uncertainty of NIST Measurement Result," NIST Technical Note 1297, U.S. Government Printing Office, Washington, DC, (1994).

2203A Commerce Road, Suite 1
Forest Hill, MD 21050 USA
1.410.838.8780

CERTIFICATE OF ANALYSIS

**University of Notre Dame**	**Site Name:** So-Cal Military Toxic Site	**Beacon Proposal:** 201201H01
Department of Biological Sciences	**Site Location:** Notspa, CA	**Lab Work Order:** 0005542
South Bend, IN 46556	**Project Manager:** Kristin Shrader-Frechette	**Reported:** 01/25/2021

Working Standard Preparation Summary

BEACON ENVIRONMENTAL

2203A Commerce Road, Suite 1
Forest Hill, MD 21050 USA
1.410.838.8780

CERTIFICATE OF ANALYSIS

**University of Notre Dame**	**Site Name:** So-Cal Military Toxic Site	**Beacon Proposal:** 201201H01
Department of Biological Sciences	**Site Location:** Notspa, CA	**Lab Work Order:** 0005542
South Bend, IN 46556	**Project Manager:** Kristin Shrader-Frechette	**Reported:** 01/25/2021

Working Standard Preparation Summary

Parent Standard	Amount (ml)	FinalVolume (ml)	Prepared	Expires
**Calibration QC**				
Standard: 2000337 / 8260 Calibration Std, 5ng, Working			10/19/2020	02/18/2023
2000090	0.5000	1.00		
Standard: 2000338 / 8260 Calibration Std, 10ng, Working			10/19/2020	02/18/2023
2000091	0.5000	1.00		
Standard: 2000339 / 8260 Calibration Std, 25ng, Working			10/19/2020	02/18/2023
2000092	0.5000	1.00		
Standard: 2000340 / 8260 Calibration Std, 50ng, Working			10/19/2020	02/18/2023
2000093	0.5000	1.00		
Standard: 2000341 / 8260 Calibration Std, 100ng, Working			10/19/2020	02/18/2023
2000094	0.5000	1.00		
Standard: 2000342 / 8260 Calibration Std, 200ng, Working			10/19/2020	02/18/2023
2000095	0.5000	1.00		
Standard: 2000343 / 8260 Calibration Std, 2.5ng, Working			10/19/2020	02/18/2023
2000089	0.5000	1.00		
Standard: 2000344 / 8260 Calibration Std, 400ng, Working			10/19/2020	06/10/2022
1900054	0.5000	1.00		
Standard: 2000345 / 8260 Calibration Std, 600ng, Working			10/19/2020	06/10/2022
1900055	0.5000	1.00		
Standard: 2000346 / 8260 Calibration Std, 800ng, Working			10/19/2020	06/10/2022
1900056	0.5000	1.00		
Standard: 2000347 / 8260 Calibration Std, 1000ng, Working			10/19/2020	06/10/2022
1900057	0.5000	1.00		
**Sequence/Sample QC**				
Standard: 1900037 / LCSD Second Source Working Standard, 50ng			06/17/2019	11/29/2021
1900018	0.5000	1.00		
Standard: 2000349 / CCV, 50ng			10/19/2020	02/18/2023
2000093	0.5000	1.00		
Standard: 2000431 / Working ISTD/SURR, Sub-Stock			12/21/2020	12/28/2020
1900019	0.0500	1.00		

2203A Commerce Road, Suite 1
Forest Hill, MD 21050 USA
1.410.838.8780

CERTIFICATE OF ANALYSIS

**University of Notre Dame**	**Site Name:** So-Cal Military Toxic Site	**Beacon Proposal:** 201201H01
Department of Biological Sciences	**Site Location:** Notspa, CA	**Lab Work Order:** 0005542
South Bend, IN 46556	**Project Manager:** Kristin Shrader-Frechette	**Reported:** 01/25/2021

Standard: 2100001 / Working ISTD/SURR, Sub-Stock			01/04/2021	01/11/2021
1900019	0.0500	1.00		
Standard: 2100002 / Working BS ISTD/SURR, Sub-Stock			01/04/2021	01/11/2021
1900019	0.0500	2.00		

2203A Commerce Road, Suite 1
Forest Hill, MD 21050 USA
1.410.838.8780

BEACON ENVIRONMENTAL

CERTIFICATE OF ANALYSIS

**University of Notre Dame**		**Site Name:**	So-Cal Military Toxic Site	**Beacon Proposal:**	201201H01
Department of Biological Sciences		**Site Location:**	Notspa, CA	**Lab Work Order:**	0005542
South Bend, IN 46556		**Project Manager:**	Kristin Shrader-Frechette	**Reported:**	01/25/2021

Sample Purge Log

Laboratory: Beacon Environmental

Work Order: 0005542 **Matrix:** Indoor Air

BEACON ENVIRONMENTAL

☑ Quant File Saved to Job Folder
☑ Data Entered into LIMS

Analysis List:
A_TO-17 BES PSV (ug/m3)

Analysis Sequence

B21A006

Instrument: K System
Sequence Date: 01/06/2021

Calibration ID: BL00006

Lab Number	Sample Name	STD ID	ISTD ID	Client	Comments
B21A006-TUN1		1800015			
21A0007-BS1			2100002		
21A0007-BLK1			2100001		
B21A006-ICV1		1900037			
0005542-08	H		2100001	University of Notre Dame	
0005542-04	D		2100001	University of Notre Dame	
B21A006-CCV1		2000349			

Standard/Description		Prepared	PreparedBy	Expiration
1800015	BFB, Tuning Solution, 2500ug/ml	11/01/2019 13:17	Peter B. Kelly	08/01/2022 00:00
1900037	LCSD Second Source Working Standard, 50ng	06/17/2019 13:57	Peter B. Kelly	11/29/2021 00:00
2000348	8260 LCS/CCV Stock, 100ng	10/19/2020 09:56	Peter B. Kelly	02/19/2023 00:00
2000349	CCV, 50ng	10/19/2020 09:59	Peter B. Kelly	02/18/2023 00:00
2100001	Working ISTD/SURR, Sub-Stock	01/04/2021 08:02	Allison T. Felter	01/11/2021 00:00
2100002	Working BS ISTD/SURR, Sub-Stock	01/04/2021 08:02	Allison T. Felter	01/11/2021 00:00

Printed: 01/06/2021 7:05 am

BEACON ENVIRONMENTAL

☑ Quant File Saved to Job Folder
☑ Data Entered into LIMS

Analysis List:
A_TO-17 BES PSV (ug/m3)

Analysis Sequence

B21A005

Instrument: K System
Sequence Date: 01/05/2021

Calibration ID: BL00006

Lab Number	Sample Name	STD ID	ISTD ID	Client	Comments
B21A005-TUN1		1800015			
21A0006-BS1			2100002		
21A0006-BLK1			2100001		
B21A005-ICV1		1900037			
0005542-01	A		2100001	University of Notre Dame	
0005542-02	B		2100001	University of Notre Dame	
0005542-03	C		2100001	University of Notre Dame	
~~0005542-04~~	~~D~~		~~2100001~~	~~University of Notre Dame~~	
0005542-05	E		2100001	University of Notre Dame	
0005542-06	F		2100001	University of Notre Dame	
0005542-07	G		2100001	University of Notre Dame	
~~0005542-08~~	~~H~~		~~2100001~~	~~University of Notre Dame~~	1.6.21
0005542-09	I		2100001	University of Notre Dame	
0005542-10	J		2100001	University of Notre Dame	
0005542-11	J-DUP		2100001	University of Notre Dame	
0005542-12	K		2100001	University of Notre Dame	
B21A005-CCV1		2000349			

Printed: 01/05/2021 1:09 pm

Analysis List:
A_TO-17 BES PSV (ug/m3)

Analysis Sequence

B21A005

(Continued)

Instrument: K System

Sequence Date: 01/05/2021

Standard/Description		Prepared	PreparedBy	Expiration
1800015	BFB, Tuning Solution, 2500ug/ml	11-01-2019 13:17	Peter B. Kelly	08-01-2022 00:00
1900037	LCSD Second Source Working Standard, 50ng	06-17-2019 13:57	Peter B. Kelly	11-29-2021 00:00
2000348	8260 LCS/CCV Stock, 100ng	10-19-2020 09:56	Peter B. Kelly	02-19-2023 00:00
2000349	CCV, 50ng	10-19-2020 09:59	Peter B. Kelly	02-18-2023 00:00
2100001	Working ISTD/SURR, Sub-Stock	01-04-2021 08:02	Allison T. Felter	01-11-2021 00:00
2100002	Working BS ISTD/SURR, Sub-Stock	01-04-2021 08:02	Allison T. Felter	01-11-2021 00:00

Printed: 01/05/2021 1:09 pm

BEACON ENVIRONMENTAL

★ 12 Hour Tune Time

WORK ORDER: 0005542

Sample Purge Log

Client: University of Notre Dame
Project: So-Cal Military Toxic Site
Reporting Options: Level4
Sampler: Beacon Passive Sampler
TAT: 5 Day(s)
Analysis: TO-17 BES PSV (ug/m3)

PURGE TIME: One hour

Beacon Passive Sampler

Purge Gas Tank PSI @Start	Date/Time Purge Start	Initials	LabNumber	SampleID	ReceivedDate	TAT 5 Day(s) DueDate	Comments: (e.g. Wet, Discolored, Damaged, QA/QC Requirements)
800	01/05/2021 0710	AIF	0005542-01	A	1/4/2021	1/11/2021	
			0005542-02	B	1/4/2021	1/11/2021	
			0005542-03	C	1/4/2021	1/11/2021	
			0005542-04	D	1/4/2021	1/11/2021	
			0005542-05	E	1/4/2021	1/11/2021	
			0005542-06	F	1/4/2021	1/11/2021	
			0005542-07	G	1/4/2021	1/11/2021	
			0005542-08	H	1/4/2021	1/11/2021	
			0005542-09	I	1/4/2021	1/11/2021	
			0005542-10	J	1/4/2021	1/11/2021	
			0005542-11	J-DUP	1/4/2021	1/11/2021	
			0005542-12	K	1/4/2021	1/11/2021	
Stop:	01/05/2021 0810						

Sample Count: 12
